# Dereplicative Combination of HPLC/DAD/MS and 2D NMR to Identify Lichexanthone Isomers in Lichen Extracts

**DOI:** 10.1002/pca.70043

**Published:** 2025-12-08

**Authors:** Solenn Ferron, Marylène Chollet‐Krugler, Hermann Pinson, Rania Marzoug, Philippe Uriac, Françoise Lohézic‐Le Dévéhat

**Affiliations:** ^1^ CNRS, ISCR (Institut des Sciences Chimiques de Rennes)—UMR 6226, Univ Rennes Rennes France

**Keywords:** dereplication, HPLC/DAD/MS, *Lecanora alboflavida*, lichens, NMR, xanthones

## Abstract

**Introduction:**

Lichexanthones are the major xanthones found in lichens. They present a high degree of isomerism, which makes their identification tedious. Xanthones are known to occur in lichens according to chemosyndromes, and these compounds act as chemotaxonomic markers. Many lichens that produce xanthones are crustose lichens from *Lecanora* or *Pertusaria* genera, which often leads to small amounts of extracts being analyzed.

**Objective:**

We aimed to set up a method able to identify the right isomers of lichexanthones contained in the extract of a xanthone‐producing lichen. This method has to produce reliable results even without all the possible isomers at hand, as they are numerous and difficult to obtain.

**Material and Methods:**

The 16 norlichexanthones were obtained by a strategy combining isolation and synthesis. All of them were characterized by a full set of NMR experiments, highlighting key features, and an HPLC/DAD/MS method was developed. To exemplify the method, selected lichens were submitted to acetone microextraction and the extracts were analyzed by HPLC/DAD/MS and NMR.

**Results:**

All norlichexanthones were well separated by HPLC/DAD/MS, which enables their identification in the lichen extracts, provided that the retention time of all the isomers is known; ^13^C NMR is very informative about the position of chlorine in norlichexanthones but lacks sensitivity. 2D NMR provides a high level of structural information even on complex extracts.

**Conclusion:**

In addition to HPLC/DAD/MS, NMR can be used directly on a lichen extract to confirm the positions of the chlorine atoms on the lichexanthone scaffold, thanks to the HSQC experiment. Furthermore, the NOESY experiment gives the position of methylations for a comprehensive overview of the substitution patterns involved in the extract of a xanthone‐producing lichen, without requiring the entire series of the 64 derivatives.

## Abstract 2

Xanthones are secondary metabolites deriving from the *9H*‐xanthen‐9‐one scaffold. In lichens, lichexanthones are important chemotaxonomic markers to distinguish between morphologically related species. They can be chlorinated at Positions 2, 4, 5 and 7, and methylated at Positions 3 and 6, resulting in a high number of isomers. We created a library of the 16 non‐methylated derivatives (called norlichexanthones), developed an HPLC/DAD/MS method capable of separating them and elaborated a 2D NMR strategy to identify the isomers.

## 1 Introduction

Lichens are fungi that have developed a symbiotic way of life with a photobiont, which can be an alga, a cyanobacteria, or both. This symbiosis forms organisms that are extremely resistant to environmental constraints like UV exposition. One possible explanation for this resistance is their capacity to synthesize a wide range of secondary metabolites, which usually represent around 10% of their weight. These compounds belong mostly to compound classes specific to lichens, like depsides or depsidones. As they are taxonomic markers for species with very close morphology, they have been studied for a long time, mainly with TLC, even if HPTLC and HPLC methods have been developed [1]. More recently, MS/MS libraries of compounds have been deposited in the GNPS platform [2, 3, 4]. Despite these huge improvements, lichenochemistry mostly relies on TLC analyses, as it is widely used by lichenologists for taxonomic purposes.

Among lichen products, xanthones are not specific to lichens, but are also produced by plant, bacterial and fungal species [5]. The most common and representative family of xanthones in lichens is the lichexanthones. Their 1,3,6‐trihydroxy‐8‐methyl‐9*H*‐xanthen‐9‐one scaffold results from a specific biosynthetic pathway [6] achieved by a non‐reducing polyketide synthase (NR‐PKS) [7]. Post‐PKS modifications include chlorinations that can occur at Positions 2, 4, 5 and 7, as well as methylations at positions 3 and 6, leading to 64 possible structures presenting a high isomeric degree.

Lichexanthones are present in lichens according to chemosyndromes [8], and sometimes a high number of these compounds exists in a lichen extract. The co‐occurrence of these compounds can be explained by the successive chlorinations and methylations of norlichexanthone, which is the starting point for all these derivatives [9]. Despite the existence of standardized methods for lichen compound identification [10] and specific efforts regarding lichexanthones [11] it is still difficult to identify the lichexanthone derivatives in a lichen extract, because the discrimination between isomers requires either structurally informative methods, or the entire series of the compounds to compare their chromatographic behavior by co‐elution with the extract.

NMR is the method of choice for structural requirements, but it is efficient mostly on pure compounds. Here, we demonstrate the interest of very simple HSQC‐NMR observations to get the necessary structural information, combined with HPLC/DAD/MS dereplication. The 16 non‐methylated lichexanthones derivatives, also named norlichexanthones, were obtained either by synthesis or by lichen extraction and isolation. An HPLC/DAD/MS method was set up to separate those compounds and a comprehensive set of NMR experiments enabled us to identify the distinguishing features between the isomers. As a result, a method involving 2D NMR and HPLC/DAD/MS is presented which succeeded in unambiguously identifying the lichexanthone derivatives in lichen extracts, and the complementarity of these techniques is highlighted through the example of the lichen species *Lecanora alboflavida* , which was found to contain a great diversity of lichexanthone derivatives.

## 2 Experimental

### 2.1 General Experimental Procedures

Flash chromatography was realized on a Gilson PLC2050 system, on two types of columns. Normal phase separations were achieved on Si columns (PuriFlash Si‐HP 50 μm, Interchim), with a liquid injection and an elution with a gradient of cyclohexane and ethyl acetate. Reversed‐phase separations were achieved on C18 columns (Puriflash C18‐HP 50 μm, Interchim), with a dry‐load injection and a gradient of water and methanol.

Semi‐preparative HPLC purification was performed on a Shimadzu LC20AD system with a reversed‐phase Prevail C18‐Select column (Grace) (250 × 10 mm, 5 μm) and a gradient of A (0.1% formic acid in water) and B (0.1% formic acid in acetonitrile), eluting from 40% B to 100% B in 35 min.

### 2.2 Lichen Material

*Lecidella asema* var. *elaeochromoides* (Nyl.) Nimis & Tretiach (1996) (67/JYM04/2014) and *Myriolecis antiqua* (J.R. Laundon) Śliwa, Zhao Xin & Lumbsch (2015) (41/JYM24/2014) were collected on rocks by Jean‐Yves Monnat in March 2014 at la Pointe du Raz (Plogoff, 29 Finistère, France) and in Vernay (Acigné, 35 Ille‐et‐Vilaine, France), respectively.

*L. alboflavida* Taylor (1836) (93/PU/2022) and *Pyrrhospora quernea* (Dicks.) Körb (1855) (102/PU/2023) were collected on the bark of *Quercus* sp. by Philippe Uriac in the forest of Rennes (Liffré, 35 Ille‐et‐Vilaine, France) in June 2022 and in January 2023, respectively.

*Lecidella elaeochroma* (Ach.) M. Choisy (1950) (162/SF/2024) was collected on the bark of *Quercus* sp. by Solenn Ferron in L'Aubriais (Gahard, 35 Ille‐et‐Vilaine, France) in May 2024.

All vouchers are stored at the University of Rennes.

### 2.3 Preparation of the Xanthones

#### 2.3.1 Extraction and Isolation From Lichens (Compounds 7, 9, 12, and 16)

*L. alboflavida* thallus (0.5 g) was grounded in liquid N_2_ and extracted twice with 20 mL of acetone at 40°C under sonication for 30 min. After filtration and evaporation of acetone under vacuum, 44 mg of crude extract was obtained. The extract was dissolved in acetone and submitted to semi‐preparative HPLC, resulting in the isolation of 1.5, 14, and 2 mg of compounds **9**, **12**, and **16**, respectively (Figures S39–S43, S56–S58, and S73–S76).

*L. asema* var. *elaeochromoides* (0.45 g) was submitted to the same process to afford 40 mg of crude extract. Semi‐preparative HPLC separation of this extract gave 1 mg of compound **7** (Figures S30‐S33).

#### 2.3.2 Synthesis of Building Blocks 17–20

**5‐chloro‐orsellinic acid 17**: Orsellinic acid (500 mg, 3.0 mmol) was dissolved in 25 mL of anhydrous diethyl ether (Et_2_O). Sulfuryl chloride (289 μL, 3.6 mmol, 1.2 eq.) in 5 mL Et_2_O is added dropwise. The solution was stirred at room temperature for 2 h, then the reaction was quenched by the addition of 25 mL distilled water and extracted with Et_2_O. Compound **17** (570 mg, 2.8 mmol, 95%) was obtained as a mixture (79/21) with 3,5‐dichloro‐orsellinic acid and used without further purification (Figure S77).

**3‐chloro‐orsellinic acid 18**: Methyl‐4‐hydroxy‐6‐methyl‐2‐oxocyclohex‐3‐ene‐1‐carboxylate (1 g, 5.4 mmol) was dissolved in 5 mL acetic acid, and chlorine (0.77 g, 10.7 mmol, 2 eq.) in 10 mL acetic acid was added dropwise with stirring and ice‐cooling. The solution was stirred at room temperature for 0.5 h and at 60°C for 2.5 h, then the reaction was quenched by iced water and extracted with Et_2_O. Methyl‐3‐chloro‐orsellinate (750 mg, 3.5 mmol, 64%) was obtained as a white amorphous solid and was further stirred with benzyl bromide (0.9 mL, 7 mmol, 2 eq.) and potassium carbonate (2 g, 14 mmol, 4 eq.) in *N,N*‐dimethylformamide (5 mL) for 15 h. Benzyl bromide in excess was removed by evaporation then water was added. The solution was extracted with Et_2_O, washed with water, and dried on sodium sulfate to afford methyl‐2,4‐bisbenzyloxy‐3‐chloro‐orsellinate. This ester (800 mg, 2 mmol) was stirred for 6 h on a steam bath with potassium hydroxide (800 mg) in water (1 mL) and dimethylsulfoxide (15 mL). The reaction was quenched by water, extracted with Et_2_O, washed, dried on sodium sulfate, and evaporated to give 570 mg of the corresponding carboxylic acid. The acid (570 mg, 1.49 mmol) and 10% palladized charcoal (110 mg) were stirred in ethanol (15 mL) under hydrogen (3 bar, 48 h) to give 3‐chloro‐orsellinic acid **18** (180 mg, 0.89 mmol, Figure S78).

**3,5‐dichloro‐orsellinic acid 19**: Orsellinic acid (50 mg, 0.3 mmol) was dissolved in 0.4 mL of acetic acid. Sulfuryl chloride (50 μL, 0.6 mmol, 2 eq.) in 1 mL Et_2_O was added. The solution was stirred at room temperature for 4 h, then the reaction was quenched by the addition of 5 mL distilled water and extracted with Et_2_O. Compound **19** was obtained as a white amorphous solid (69 mg, 0.3 mmol, 98%, Figure S79) and used without further purification.

**Chlorophloroglucinol 20**: Phloroglucinol (1 g, 7.9 mmol) was dissolved in 35 mL of Et_2_O. Sulfuryl chloride (0.72 mL, 7.94 mmol, 1 eq.) in 10 mL Et_2_O was added dropwise. The solution was stirred at room temperature for 2 h, then the reaction was quenched by the addition of 30 mL distilled water and extracted with Et_2_O. Compound **20** was obtained (834 mg, 5.2 mmol, 65%, Figure S80) after purification by normal phase flash chromatography.

#### 2.3.3 Synthesis of Xanthones by Condensation

Orsellinic and phloroglucinol derivatives were dissolved in Eaton's reagent (7.7% P_2_O_5_ in methanesulfonic acid). The solution was stirred at room temperature for 4 days, quenched by iced water, and extracted with ethyl acetate.

**Norlichexanthone 1**: 0.5 g of orsellinic acid (2.97 mmol) and 0.375 g of phloroglucinol (2.97 mmol, 1 eq.) reacted with 30 mL of Eaton's reagent to afford 287 mg of compound **1** (1.11 mmol, 37%, Figures S1‐S5) after purification by reversed‐phase flash chromatography.

**2‐chloronorlichexanthone 2 and 4‐chloronorlichexanthone 3**: 350 mg of orsellinic acid (2.08 mmol) and 334 mg of chlorophloroglucinol **20** (2.08 mmol, 1 eq.) reacted with 22 mL of Eaton's reagent to afford 101 mg of compound **2** (0.35 mmol, 17%, Figures S6–S10) and 46 mg of compound **3** (0.16 mmol, 8%, Figures S11–S15) after purification by normal phase flash chromatography.

**5‐chloronorlichexanthone 4**: 80 mg of 3‐chloro‐orsellinic acid **18** (0.39 mmol) and 55 mg of phloroglucinol (0.43 mmol, 1.1 eq.) reacted with 8 mL of Eaton's reagent to afford 24 mg of compound **4** (0.07 mmol, 19%, Figures S16–S20) after purification by semi‐preparative HPLC.

**7‐chloronorlichexanthone 5**: 300 mg of 5‐chloro‐orsellinic acid **17** (1.48 mmol) and 205 mg of phloroglucinol (1.63 mmol, 1.1 eq.) reacted with 15 mL of Eaton's reagent to afford 109 mg of compound **5** (0.37 mmol, 25%, Figures S21–S24) after purification by reversed‐phase flash chromatography.

2**,7‐dichloronorlichexanthone 8**: 250 mg of 5‐chloro‐orsellinic acid **17** (1.23 mmol) and 218 mg of chlorophloroglucinol **20** (1.35 mmol, 1.1 eq.) reacted with 20 mL of Eaton's reagent to afford 48 mg of compound **8** (0.15 mmol, 12%, Figures S34–S38) after purification by reversed‐phase flash chromatography.

**5,7‐dichloronorlichexanthone 11**: 150 mg of 3,5‐dichloro‐orsellinic acid **19** (0.63 mmol) and 120 mg of phloroglucinol (1.63 mmol, 1.1 eq.) reacted with 15 mL of Eaton's reagent to afford 47 mg of compound **11** (0.14 mmol, 23%, Figures S49–S53) after purification by reversed‐phase flash chromatography.

#### 2.3.4 Synthesis of Xanthones by Chlorination (Compounds 6, 10, 13, 14, and 15)

**2,4‐dichloronorlichexanthone 6**: 100 mg of norlichexanthone **1** (0.39 mmol) was dissolved in THF (10 mL). Sulfuryl chloride (34 μL, 1.1 eq.) in 5 mL of Et_2_O was added dropwise. The solution was stirred for 2 h at room temperature, then quenched with 20 mL of water and extracted with ethyl acetate. Compound **6** (24 mg, 0.07 mmol, 17%, Figures S25–S29) was obtained after purification by semi‐preparative HPLC.

**4,7‐dichloronorlichexanthone 10 and 2,4,7‐trichloronorlichexanthone 13**: 80 mg of 7‐chloro‐norlichexanthone **5** (0.27 mmol) was dissolved in Et_2_O (8 mL) and THF (2 mL). Sulfuryl chloride (20 μL, 1.1 eq.) in 8 mL of Et_2_O was added dropwise. The solution was stirred for 2 h at room temperature, then quenched with 15 mL of water and extracted with ethyl acetate. Compounds **10** (22 mg, 0.07 mmol, 18%, Figures S44–S48) and **13** (7 mg, 0.02 mmol, 4%, Figures S59–S62) were obtained after purification by semi‐preparative HPLC.

**2,5,7‐trichloronorlichexanthone 14 and 4,5,7‐trichloronorlichexanthone 15**: 160 mg of 5,7‐dichloronorlichexanthone **11** (0.49 mmol) was dissolved in 15 mL Et_2_O and 5 mL THF. Sulfuryl chloride (20 μL, 1.1 eq.) in 20 mL of Et_2_O was added dropwise. The solution was stirred for 2 h at room temperature, then quenched with 50 mL of water and extracted with ethyl acetate. Compound **14** (4 mg, 0.01 mmol, 2%, Figures S63–S67) and compound **15** (33 mg, 0.09 mmol, 18%, Figures S68–S72) were obtained after purification by reversed‐phase flash chromatography followed by semi‐preparative HPLC.

### 2.4 Chromatographic and Spectroscopic Analyses

#### 2.4.1 HPLC/Dad/MS

HPLC/DAD/MS analyses were conducted on a Prominence Shimadzu LC‐20ad system equipped with an ADVION expression CMS mass spectrometer. The chromatography separation was achieved on a Phenomenex Kinetex C18 column (2.6 μm, 100 × 4.6 mm), with a gradient program optimized for non‐methylated xanthones using 0.1% formic acid in HPLC grade water (solvent A) and 0.1% formic acid in acetonitrile (solvent B) at a flow rate of 0.5 mL/min. The gradient was: 0 min: 40% B; 5 min: 40% B; 15 min: 44% B; 20 min: 44% B; 39 min: 64% B; 41 min: 100% B; 44 min: 100% B; 46 min: 40% B; 53 min: 40% B. Mass spectrometry detection was realized with an electrospray ionization (ESI) source in negative mode.

#### 2.4.2 NMR Experiments

All spectra were acquired in acetone‐*d*_6_ (Eurisotop). Experiments were performed either on a Bruker 500 MHz spectrometer with a TCI cryo‐probe, or on a Bruker 500 MHz equipped with a BBO probe, at the PRISM core facility (Rennes, France).

2D experiments included edited heteronuclear single quantum correlation (HSQCed), nuclear overhauser effect spectroscopy (NOESY), and heteronuclear multiple bond correlation (HMBC). Non‐uniform sampling (NUS) has been used for both HSQC and HMBC experiments, with NUS amounts of 25% and 50%, respectively.

### 2.5 Analytical Microextraction of Lichens

To provide extracts for the dereplication step, *L. alboflavida* , *M. antiqua* , *P. quernea* , and *L. elaeochroma* were submitted to microextractions. Small parts of these lichens (around 15 mm^2^) were ground in a 4‐mL tube and extracted with 2 mL of acetone at 40°C for 30 min under sonication. The solutions were then filtered and evaporated. 10 μL of a 3 mg/mL solution was injected to HPLC/DAD/MS analyses were performed on 10 µL of 3 mg/mL solutions of each lichen extract (Figures S117–S121). The residual extract was evaporated to dryness and then dissolved in acetone‐*d*_6_ for NMR acquisitions (Figures S81–S94).

## 3 Results and Discussion

### 3.1 Obtention of the 16 Non‐Methylated Lichexanthones

In lichens, norlichexanthone can be chlorinated at positions 2, 4, 5, and/or 7, leading to 16 derivating structures with the generic name of norlichexanthones. Additionally, these 16 norlichechexanthones can be methylated at Positions 3 and/or 6. The compounds are named lichexanthones when both of the positions are methylated. To make things easier, one of us (PU) introduced a convenient way to name them. The structure of lichexanthone is modeled by an “L,” preceded either by “O” if it is not methylated, or by the position(s) of the methyl group(s). Accordingly, it is followed by an “O” if it is not chlorinated, or by the position(s) of the chlorine atom(s).

**FIGURE 1:**
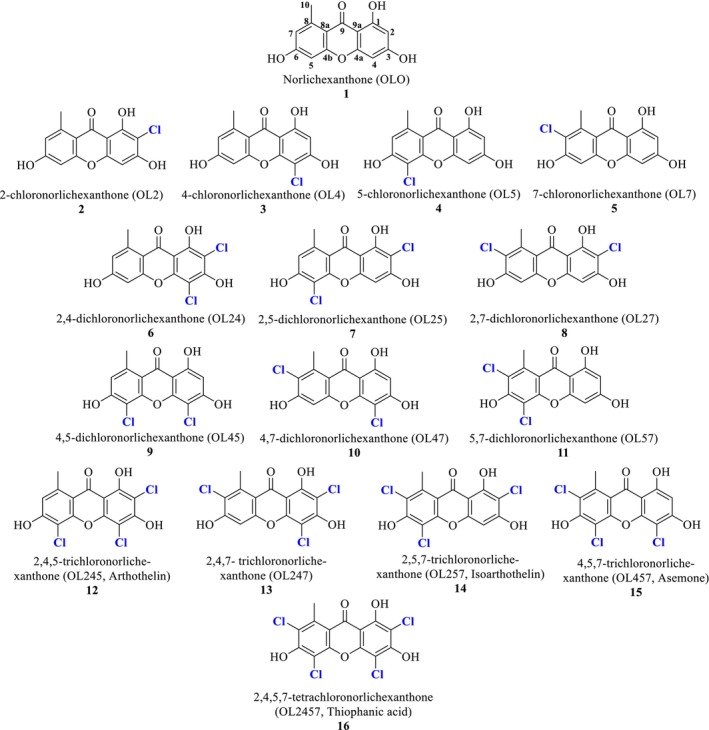
Structures, names, and synonymies of the compounds **1**–**16**.

The 16 norlichexanthones (compounds **1–16**, Figure 1) were obtained either by synthesis or by isolation from lichen extracts, in order to build an analytical database containing HPLC/DAD/MS data (retention times and m/z) and NMR data (proton and carbon chemical shifts).

Isolation from a lichen extract can be very efficient for some compounds as lichens often produce one or two compounds in large proportions, representing the huge majority of the extract. We could thus isolate compounds **12** and **16** from *L. alboflavida* , a corticolous lichen found in Brittany, but mostly described from the British Isles [12]. Besides those readily available compounds, isolation can also afford xanthones more difficult to obtain by synthesis. Compounds **7** and **9** were obtained in minute amounts from *L. asema* var. *elaeochromoides* and *L. alboflavida* , respectively.

**FIGURE 2:**
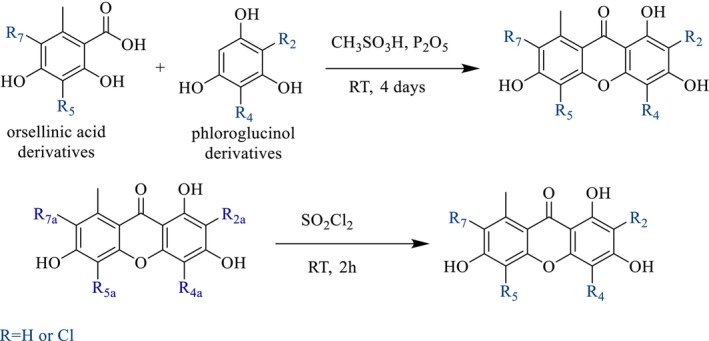
Two synthesis strategies.

As xanthones‐producing lichens are often crustose lichens, the biomass availability can be an issue to obtain minor compounds. We thus decided to synthesize most of them. Two main strategies were used: condensation of an orsellinic acid derivative with a phloroglucinol, which was chlorinated at selected positions, or chlorination of a norlichexanthone (Figure 2).

**FIGURE 3:**
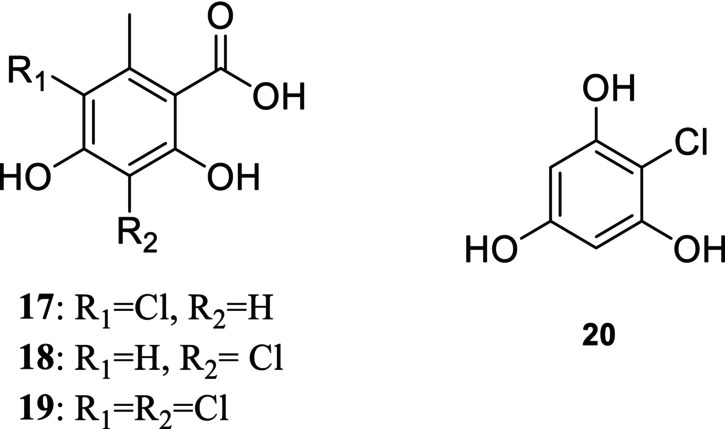
Structures of the compounds **17**–**20**.

The first strategy involved Eaton's reagent [13], and is commonly used for the condensation of salicylic acid with a phenol derivative [14, 15] as an improvement of the original GSS method developed by Grover, Shah and Shah [16]. In our case, orsellinic acid and its three chlorinated derivatives **17**–**19** reacted with phloroglucinol or one of its chlorinated derivatives **20** (Figure 3) [17].

Chlorination of orsellinic acid to obtain 5‐chloroorsellinic acid was very simply achieved by the addition of sulfuryl chloride, either in diethyl ether for monochlorination, or in acetic acid for dichlorination. Chlorination of orsellinic acid with sulfuryl chloride occurs at position 5 preferentially. 3‐Chloroorsellinic acid is therefore obtained by Cl_2_ chlorination of methyl‐4‐hydroxy‐6‐methyl‐2‐oxocyclohex‐3‐ene‐1‐carboxylate [18], followed by the protection of phenol moieties, demethylation and deprotection.

Condensation reactions are simple and give correct yields if orsellinic acid, phloroglucinol or both are not chlorinated. Compounds **1**, **2**, **3**, **4**, **5**, and **11** were obtained that way in good amounts. When both reagents are monochlorinated, yields are low or even zero: 12% for compound **8** and 0% for compounds **7**, **9**, and **10**.

Some compounds (**6**, **10**, **13**, **14**, and **15)** have been efficiently obtained by simple chlorination of either norlichexanthone **1** or a mono‐ or dichloronorlichexanthone by sulfuryl chloride. Chlorination reactions are efficient with a rapid conversion and few by‐products, and are typically useful to obtain trichloronorlichexanthones for which condensation leads to low yields.

These different strategies enabled us to obtain the complete series of the norlichexanthones.

### 3.2 HPLC/DAD/MS Analysis

With all the compounds at hand, a chromatographic separation method of the norlichexanthones has been set up. The 16 compounds were well separated thanks to their retention times (*Rt*) and m/z (Figures S97–S116).

Some of the compounds present a very similar retention time, for example, OL245 (*Rt* = 18.8 min) and OL27 (*Rt* = 19 min), but can be distinguished by their lowest *m/z* (359 and 325, respectively). All isomers are separated by at least 0.6 min, which rules out any risk of confusion. Retention times and *m/z* ratios are therefore both necessary to resolve overlaps that exist among the 16 compounds.

**FIGURE 4:**
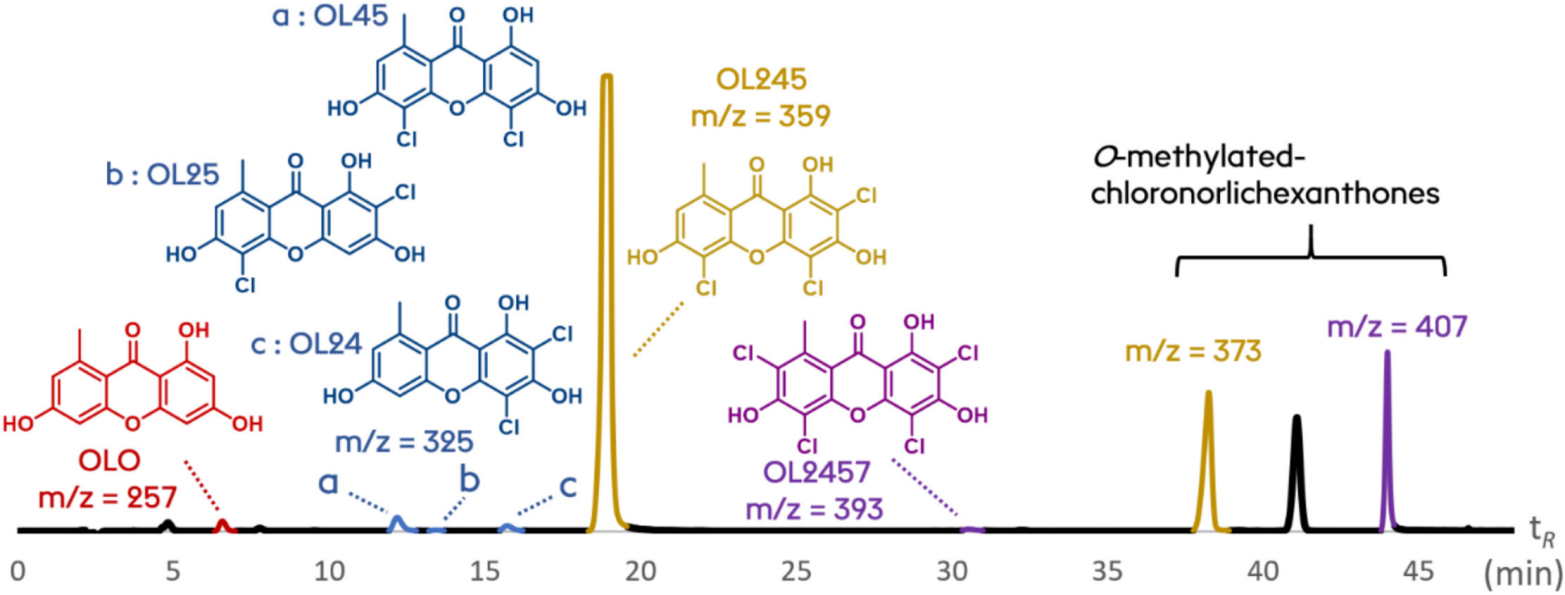
HPLC/DAD chromatogram of *Lecanora alboflavida* extract with MS annotation of the peaks.

This method allowed the dereplication of an extract of *L. alboflavida* , leading to the annotation of six norlichexanthones in the sample (Figure 4): norlichexanthone (OLO), 2,4‐dichloronorlichexanthone (OL24), 2,5‐dichloronorlichexanthone (OL25), 4,5‐dichloronorlichexanthone (OL45), 2,4,5‐trichloronorlichexanthone (OL245), and 2,4,5,7‐tetrachloro‐norlichexanthone (OL2457).

Arthothelin (OL245, 2,4,5‐trichloronorlichexanthone), which is, so far, the only compound described in *L. alboflavida* [19], is the major compound detected here. Methylated xanthones (*m/z* = 373 for a monomethyltrichloronorlichexanthone and *m/z* = 407 for a monomethyl‐tetrachloronorlichexanthone) are not annotated but are reasonably derived from the corresponding non‐methylated norlichexanthones; however, the position of the methyl group cannot be deduced from the HPLC/DAD/MS data. For example, the xanthone detected at *Rt* = 38.3 min and *m/z* 373 corresponds to a monomethyltrichloronorlichexanthone and should have its chlorines in positions 2, 4, and 5 regarding the major OL245, but the 3‐*O*‐ or 6‐*O*‐ position of the methyl group remains uncertain.

This method allows the detection and identification of minor compounds in the extract. The main drawback is the lack of structural information, as the compounds are only identified thanks to their retention time and *m/z*. Considering the number of isomers with the same exact mass, only compounds of known retention times can be identified with certainty, and very few labs in the world, if any, possess the 64 possible derivatives of the lichexanthones series. Moreover, any change in the chromatographic system leads to a new set up of the method with the need for all standards, as the compounds are very close to each other.

### 3.3 NMR Analysis

#### 3.3.1 NMR Database of Proton and Carbone Chemical Shifts

All compounds have been submitted to a comprehensive set of NMR analyses (Figures S1–S76). In previous studies [20, 21], Sundholm had reported ^13^C chemical shifts of many lichexanthones, including the entire series of the norlichexanthones and our results were in accordance with his reports. In addition, 2D experiments (HSQC, HMBC, and NOESY) allowed the unambiguous assignments of all ^1^H and ^13^C chemical shifts of the 16 compounds (Tables 1 and 2).

**TABLE 1:** ^13^C chemical shifts (125 MHz, in acetone‐*d*_6_) of norlichexanthones.

C↓	Compound															
	1	2	3	4	5	6	7	8	9	10	11	12	13	14	15	16
	OLO	OL2	OL4	OL5	OL7	OL24	OL25^b^	OL27	OL45	OL47	OL57	OL245	OL247	OL257	OL457	OL2457^a^
1	164.9	160.1	162.7	164.8	164.9	158.3	160.1^c^	160.1	162.6	162.7	164.8	158.1	157.9	159.9	162.5	156.4
2	98.7	102.9	99.1	99.3	99	103.7	103^c^	103.3	99.5	99.2	99.4	104.2	104	103.8	99.6	102.2
3	165.5	160.7	161.5	166	165.8	156.6	161	161.2	161.8	161.2	166	157	157.1	161.4	161.5	156.8
4	94	94.3	98.1	94.3	94	98.9	94.4	94.3	98.7	98.1	94.3	99.6	99.1	94.6	98.6	98.8
5	101.5	101.6	101.7	106.1	102.4	101.7	106.3	102.4	106.7	102.5	107.7	106.7	102.5	107.8	108.1	107.2
6	163.5	163.8	163.8	159.1	159.1	164.2	159.2	159.8	160.4	159.6	155.2	159.6	159.7	156.2	155.8	155.5
7	116.9	117.1	117.4	116.3	120.5	117.7	116.3	120.9	117.2	121.2	120.6	117	121.3	121.3	121.3	121
8	144.4	144.6	144.6	141.9	141	144.8	142.2	141.1	141.8	141.2	138.8	142.2	141.3	138.8	138.9	137.5
9	183	182.8	182.7	182.7	182.6	182.5	182.5	182.5	182.5	182.5	182.3	182.4	182.1	182.1	182.2	180.2
10	23.4	23.5	23.4	23.3	18.6	23.4	23	18.7	23.3	18.7	18.6	23.3	18.7	18.7	18.6	18.3
8a	112.6	112.3	112.3	113.4	113.2	112	113.3	112.8	112.7	112.8	113.8	112.9	112.6	112.9	113.2	111
4b	160.2	160.3	160.1	155.2	158	160	156.6	158.1	155.3	157.8	153.5	155	157.7	153.5	153.4	151.7
4a	158	155.7	153.1	157.8	157.8	151.2	155.5	155.5	153	152.9	157.4	151	150.9	155.1	152.6	149.1
9a	103.8	104	104.2	103.5	103.8	104.1	103.7	103.9	103.9	104.4	103.7	103.9	104	103.8	104.3	103.8

^a^ Recorded in DMSO‐*d*_6_.

^b^ Deduced from HMBC experiment.

^c^ Deduced from additional contribution of chlorinated positions.

**TABLE 2:** 1H chemical shifts (500 MHz, in acetone‐*d*_6_) of norlichexanthones.

H↓	Compound															
	1	2	3	4	5	6	7	8	9	10	11	12	13	14	15	16
	OLO	OL2	OL4	OL5	OL7	OL24	OL25	OL27	OL45	OL47	OL57	OL245	OL247	OL257	OL457	OL2457^a^
2	6.18 (d, *J* = 2.2 Hz, 1H)	—	6.38 (s, 1H)	6.23 (d, *J* = 2.2 Hz, 1H)	6.20 (d, *J* = 2.1 Hz, 1H)	—	—	—	6.44 (s, 1H)	6.40 (s, 1H)	6.26 (d, *J* = 2.2 Hz, 1H)	—	—	—	6.44 (s, 1H)	—
4	6.30 (d, *J* = 2.2 Hz, 1H)	6.52 (s, 1H)	—	6.43 (d, *J* = 2.2 Hz, 1H)	6.30 (d, *J* = 2.1 Hz, 1H)	—	6.69 (s, 1H)	6.52 (s, 1H)	—	—	6.45 (d, *J* = 2.2 Hz, 1H)	—	—	6.66 (s, 1H)	—	—
5	6.70 (s, 1H)	6.71 (s, 1H)	6.82 (d, *J* = 2.5 Hz, 1H)	—	6.91 (s, 1H)	6.80 (d, *J* = 2.5 Hz, 1H)	—	6.93 (s, 1H)	—	7.04 (s, 1H)	—	—	7.04 (s, 1H)	—	—	—
7	6.70 (s, 1H)	6.72 (s, 1H)	6.74 (dq, *J* = 2.5, 0.9 Hz, 1H)	6.89 (q, *J* = 0.9 Hz, 1H)	—	6.75 (dq, *J* = 2.5, 1.0 Hz, 1H)	6.94 (s, 1H)	—	6.94 (bs, 1H)	—	—	6.91 (s, 1H)	—	—	—	—
10	2.77 (s, 3H)	2.78 (s, 3H)	2.78 (d, *J* = 0.9 Hz, 3H)	2.76 (d, *J* = 0.9 Hz, 3H)	2.96 (s, 3H)	2.77 (d, *J* = 1.0 Hz, 3H)	2.79 (s, 3H)	2.96 (s, 3H)	2.78 (s, 1H)	2.97 (s, 3H)	2.97 (s, 3H)	2.75 (s, 3H)	2.94 (s, 3H)	2.95 (s, 3H)	2.95 (s, 3H)	2.69 (s, 3H)
1‐OH	13.45 (s, 1H)	14.21 (s, 1H)	13.43 (s, 1H)	13.24 (s, 1H)	13.28 (s, 1H)	14.20 (s, 1H)	14.00 (s, 1H)	14.02 (s, 1H)	13.26 (s, 1H)	13.28 (s, 1H)	13.10 (s, 1H)	13.91 (s, 1H)			13.04 (s, 1H)	13.52 (s, 1H)

^a^ Recorded in DMSO‐*d*_6_.

**FIGURE 5:**
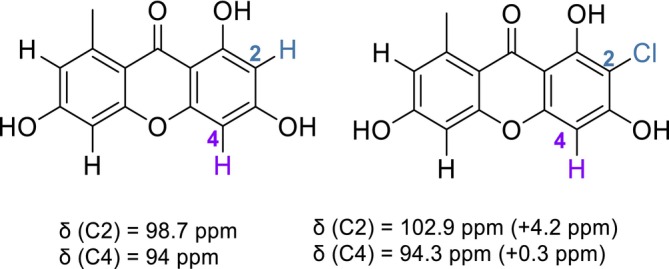
Influence of chlorination status of C‐2 on ^13^C chemical shift of C‐2 and C‐4.

In norlichexanthones, chlorine substituents have a strong deshielding effect on the carbon bearing it (+ 4–5 ppm), and a shielding effect on carbons in *ortho* (−5 ppm). However, it has nearly no effect on the carbon in *meta* position (Table 1). Chemical shifts of carbons at positions that can be occupied by either a chlorine or a hydrogen atom (i.e., Positions 2, 4, 5, and 7) are therefore not influenced by the chlorination status of each other. In the example of the 2‐chloronorlichexanthone **2**, the presence of the chlorine atom at position 2 has nearly no effect on the chemical shift of C‐4 but a deshielding effect on C‐2 (Figure 5).

#### 3.3.2 Discrimination of Isomers Using 2D NMR

**FIGURE 6:**
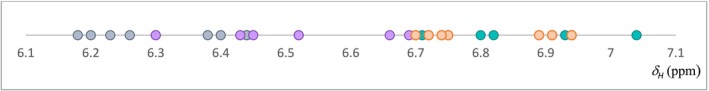
Chemical shifts of the aromatic protons (Positions 2, 4, 5, and 7) of the 16 norlichexanthones. The colors of the dots represent the positions of the protons (blue = Position 2, purple = Position 4, green = Position 5 and orange = Position 7).

The 16 molecules of the norlichexanthone series differ only by the chlorination status of positions 2, 4, 5, and 7. Therefore, to distinguish between them, we need to know whether each of these four aromatic positions is occupied by a chlorine or a hydrogen atom. Unfortunately, this distinction cannot be achieved by proton NMR analysis, because of the overlap of the positions (Figure 6).

^13^C chemical shifts give a high level of structural information, and in the context of chlorinated norlichexanthones, they are very informative about the position of chlorine atoms. Nevertheless, given the ^13^C NMR spectrum of a lichen extract containing chlorinated norlichexanthones, it could be tricky to determine the chlorination pattern of its xanthones, as the spectrum may be crowded. For example, a suitable ^13^C NMR spectrum of *L. alboflavida* extract gave 112 peaks (ns = 10,240; *t* = 9 h) on a 200 ppm 1D scale (Figure S82).

**FIGURE 7:**
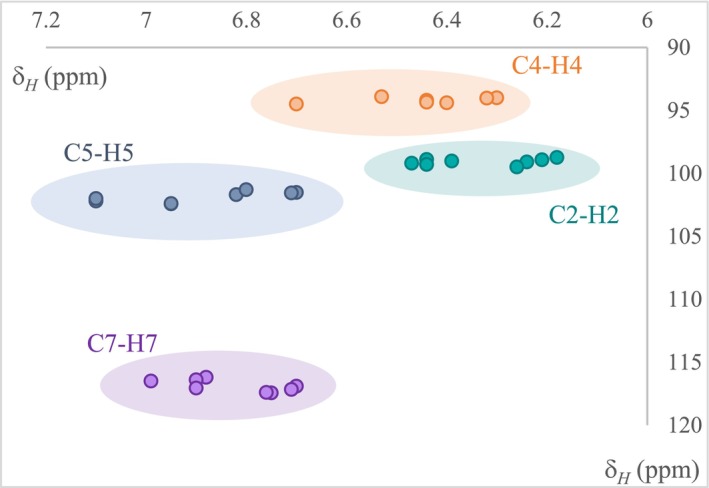
Plot of HSQC ^1^H/^13^C coordinates of the 15 norlichexanthones with at least one aromatic proton (^1^H between 6 and 7.2 ppm; ^13^C between 90 and 120 ppm). The colors of the dots represent the positions of the protons (blue = Position 2, purple = Position 4, green = Position 5 and orange = Position 7).

A convenient way to simplify the spectrum and save time while preserving the structural information is to acquire 2D NMR, and especially HSQC. For the example above of *L. alboflavida* extract, 63 peaks were obtained in 2D of 200 × 14 ppm in 2.3 h with 16 scans (Figure S83). HSQC was thus acquired for all compounds and coordinates of aromatic proton/carbon correlations were plotted on Figure 7.

Proton dimension would result in a strong overlap between protons of different positions (Figure 7), and even carbon could be slightly confusing, for example for C2 and C5 positions which can sometimes differ by not more than 1 or 2 ppm (Table 1). However, when HSQC is used, four consistent and non‐overlapping groups clearly appear according to the positions of the aromatic protons and carbons (Figure 7). Consequently, the positions which do not display an aromatic proton are chlorinated.

In *L. alboflavida* , the major norlichexanthone is arthothelin (OL245), which contains one aromatic proton at Position 7, and three chlorine atoms at Positions 2, 4, and 5. The 7‐position is easily highlighted on the HSQC spectrum of *L. alboflavida* extract (Figures S83 and S84), leading to the identification of arthothelin as the major norlichexanthone of the extract.

#### 3.3.3 NMR Dereplication of Lichen Extracts

**FIGURE 8:**
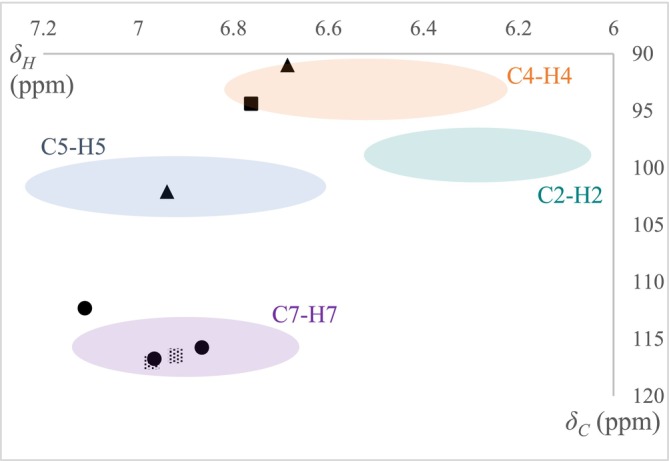
Plot of HSCQ coordinates of four lichen extracts (^1^H between 6 and 7.2 ppm; ^13^C between 90 and 120 ppm). ▲ *M. antiqua* , ░ *L. alboflavida* , ■ *P. quernea* , ● *L. elaeochroma* .

To confirm the validity of this concept and in addition to *L. alboflavida* extract, the extracts of three more lichen specimens from different species have been analyzed by HSQC‐NMR: *L. elaeochroma* , *M. antiqua* , and *P. quernea* (Figures 8, S83, S87, S90, and S93). These analyses led to the determination of the aromatic proton positions, which allowed subsequently to deduce the position of chlorine atoms in the xanthones of the extracts (Table 3).

**TABLE 3:** Interpretation table of HSQC analyses of the lichen extracts.

Lichen	Position of H	Position of Cl	Bibliographic data (references)
*M. antiqua*	45	**27**	OLO, OL2, OL7, **OL27**, **6L27**, and **36L27** [22]
*L. alboflavida*	7	**245**	**OL245** [19]
*P. quernea*	4	**257**	OL245 [19, 23], **OL257** [23, 24], OL2457 [19, 23, 24], and minor dechlorinated and/or methylated [23]
*L. elaeochroma*	7	**245**	**OL245**, **6L245**, 36L45 [19]

The information obtained may seem weak compared to the effective xanthone content of these lichens. For example, we showed previously (Figure 4) that *L. alboflavida* contains a series of norlichexanthone derivatives comprising norlichexanthone, 2,4‐, 2,5‐ and 4,5‐dichloronorlichexanthones, 2,4,5‐trichloronorlichexanthone and 2,4,5,7‐tetrachloro‐norlichexanthone. With HSQC, only the major chlorination pattern is highlighted, showing the three chlorinated Positions 2, 4, and 5. However, unlike HPLC retention times, which depend on the specific stationary phase of the column, NMR chemical shifts are independent of the spectrometer used; the norlichexanthone NMR database can therefore be used standalone, providing structural information that cannot be obtained by usual lichen extracts analyses like TLC or even HPLC/DAD/MS.

Likewise, in the three other extracts, only the major chlorination positions are well detected, and the consistency with the data provided by the literature is highlighted in bold in Table 3.

### 3.4 Combination of NMR and HPLC/MS Information to Enhance Dereplication

We have therefore demonstrated that HSQC NMR makes it possible to identify the major chlorination pattern of a lichen extract. Additionally, when methylation occurred, its position can also be determined, if the methoxy group is nearby a non‐chlorinated position, thanks to the NOESY experiment. NOESY is a proton‐proton homonuclear 2D NMR experiment that highlights a dipolar interaction between two nuclei, and thus a spatial proximity (from 2 to 5 Å of distance).

**FIGURE 9:**
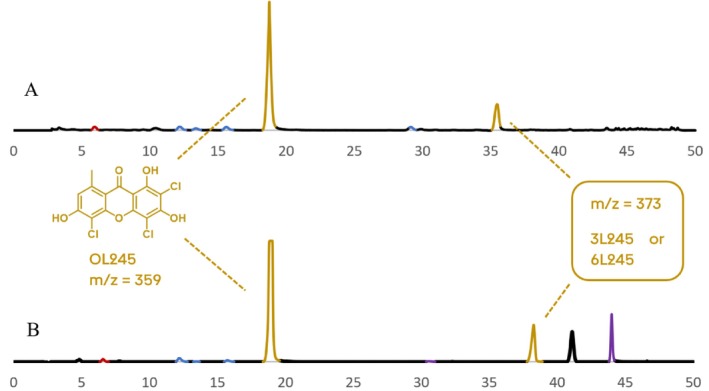
UV chromatogram (λ = 254 nm) of *L. elaeochroma* (A) and *L. alboflavida* (B) extracts.

*L. alboflavida* and *L. elaeochroma* have a very similar chlorination pattern with 2,4,5‐trichloronorlichexanthone as the major compound (Table 3), but differ in their methylated trichloronorlichexanthone which has different retention times (Figure 9).

In *L. elaeochroma* , the isomer described in the literature is the 6‐*O*‐methyl‐2,4,5‐trichloronorlichexanthone [19]. This can be easily confirmed by a NOESY experiment, which highlights the correlation between the aromatic H‐7 and the 6‐*O*‐methyl group (Figures S93 and S95). In *L. alboflavida* extract, the retention time is different (Figure 9). It also does not present any NOESY correlation between the aromatic proton and the methoxy proton, meaning that it contains the other isomer, 3‐*O*‐methyltrichloronorlichexanthone.

In addition, *L. alboflavida* presents a methylated derivative of thiophanic acid (OL2457). As this compound is fully chlorinated, there is no case in which there is an adjacent proton to the methyl group (Figure S96), and only HMBC correlations can distinguish between them.

HPLC/DAD/MS is able to separate efficiently all the norlichexanthone derivatives that can occur in a lichen extract, and the identification is possible provided that the retention times of all the compounds are known. Here we have shown that the major positions of chlorine in norlichexanthones can be determined without standard samples, with a very simple HSQC observation focused only on aromatic positions. In addition, for methylated compounds, the position of the methoxy group can be revealed thanks to NOESY correlations between this group and the adjacent proton.

2D NMR thus complements HPLC/DAD/MS by validating the position of chlorinations and extending identification to methylated derivatives, even without the complete series of 64 compounds being available (Table 4).

**TABLE 4:** Contribution of the various techniques used.

Technique	Variable	Information provided	Limitation	Application to *L. alboflavida* extract
Mass spectrometry	*m/z*	Number of chlorine atoms on the norlichexanthone	No information about the position of the chlorine atoms	OLO, 3 different dichloronorlichexanthones, one trichloronorlichexanthone, OL2457, one monomethylated trichloronorlichexanthone and one monomethylated tetrachloronorlichexanthone
Chromatographic separation	*Rt*	Isomer annotation	May vary according to the chromatographic equipment: only compounds of known *Rt* will be identify	OLO, OL24, OL25, OL45, OL245 and OL2457
HSQC NMR	H/C ^1^J correlation	H and Cl position on the norlichexanthone scaffold	Detects the major compounds only	OL245
NOESY NMR	H/H spatial correlation	OCH_3_ position (3 or 6)	No information if all the positions are chlorinated	3L245

In the frame of the lichen species chosen for this study, we also demonstrated the rich lichexanthone content of *L. alboflavida* : norlichexanthone, 2,4‐dichloronorlichexanthone, 2,5‐dichloronorlichexanthone, 4,5‐dichloronorlichexanthone, 2,4,5‐trichloronorlichexanthone (arthothelin), 2,4,5,7‐tetrachloronorlichexanthone (thiophanic acid), and 3‐*O*‐methylnorlichexanthone (thuringione), when only arthothelin was already described in this species.

## Supporting information


**Figure S1:** pca70043‐sup‐0001‐Supplementary_Material.pdf. ^1^H NMR spectrum (500 MHz) in acetone‐*d*_6_ of norlichexanthone **1**.
**Figure S2:** Jmod NMR spectrum (125 MHz) in acetone‐*d*_6_ of norlichexanthone **1**.
**Figure S3:** HSQC NMR spectrum (500/125 MHz) in acetone‐*d*_6_ of norlichexanthone **1**.
**Figure S4:** HMBC NMR spectrum (500/125 MHz) in acetone‐*d*_6_ of norlichexanthone **1**.
**Figure S5:** NOESY NMR spectrum (500 MHz) in acetone‐*d*_6_ of norlichexanthone **1**.
**Figure S6:** pca70043‐sup‐0001‐Supplementary_Material.pdf. ^1^H NMR spectrum (500 MHz) in acetone‐*d*_6_ of 2‐chloronorlichexanthone **2**.
**Figure S7:** Jmod NMR spectrum (125 MHz) in acetone‐*d*_6_ of 2‐chloronorlichexanthone **2**.
**Figure S8:** HSQC NMR spectrum (500/125 MHz) in acetone‐*d*_6_ of 2‐chloronorlichexanthone **2**.
**Figure S9:** HMBC NMR spectrum (500/125 MHz) in acetone‐*d*_6_ of 2‐chloronorlichexanthone **2**.
**Figure S10:** NOESY NMR spectrum (500 MHz) in acetone‐*d*_6_ of 2‐chloronorlichexanthone **2**.
**Figure S11:** pca70043‐sup‐0001‐Supplementary_Material.pdf. ^1^H NMR spectrum (500 MHz) in acetone‐*d*_6_ of 4‐chloronorlichexanthone **3**.
**Figure S12:** Jmod NMR spectrum (125 MHz) in acetone‐*d*_6_ of 4‐chloronorlichexanthone **3**.
**Figure S13:** HSQC NMR spectrum (500/125 MHz) in acetone‐*d*_6_ of 4‐chloronorlichexanthone **3**.
**Figure S14:** HMBC NMR spectrum (500/125 MHz) in acetone‐*d*_6_ of 4‐chloronorlichexanthone **3**.
**Figure S15:** NOESY NMR spectrum (500 MHz) in acetone‐*d*_6_ of 4‐chloronorlichexanthone **3**.
**Figure S16:** pca70043‐sup‐0001‐Supplementary_Material.pdf. ^1^H NMR spectrum (500 MHz) in acetone‐*d*_6_ of 5‐chloronorlichexanthone **4**.
**Figure S17:** Jmod NMR spectrum (125 MHz) in acetone‐*d*_6_ of 5‐chloronorlichexanthone **4**.
**Figure S18:** HSQC NMR spectrum (500/125 MHz) in acetone‐*d*_6_ of 5‐chloronorlichexanthone **4**.
**Figure S19:** HMBC NMR spectrum (500/125 MHz) in acetone‐*d*_6_ of 5‐chloronorlichexanthone **4**.
**Figure S20:** NOESY NMR spectrum (500 MHz) in acetone‐*d*_6_ of 5‐chloronorlichexanthone **4**.
**Figure S21:** pca70043‐sup‐0001‐Supplementary_Material.pdf. ^1^H NMR spectrum (500 MHz) in acetone‐*d*_6_ of 7‐chloronorlichexanthone **5**.
**Figure S22:** Jmod NMR spectrum (125 MHz) in acetone‐*d*_6_ of 7‐chloronorlichexanthone **5**.
**Figure S23:** HSQC NMR spectrum (500/125 MHz) in acetone‐*d*_6_ of 7‐chloronorlichexanthone **5**.
**Figure S24:** HMBC NMR spectrum (500/125 MHz) in acetone‐*d*_6_ of 7‐chloronorlichexanthone **5**.
**Figure S25:** pca70043‐sup‐0001‐Supplementary_Material.pdf. ^1^H NMR spectrum (500 MHz) in acetone‐*d*_6_ of 2,4‐dichloronorlichexanthone **6**.
**Figure S26:** Jmod NMR spectrum (125 MHz) in acetone‐*d*_6_ of 2,4‐dichloronorlichexanthone **6**.
**Figure S27:** HSQC NMR spectrum (500/125 MHz) in acetone‐*d*_6_ of 2,4‐dichloronorlichexanthone **6**.
**Figure S28:** HMBC NMR spectrum (500/125 MHz) in acetone‐*d*_6_ of 2,4‐dichloronorlichexanthone **6**.
**Figure S29:** NOESY NMR spectrum (500 MHz) in acetone‐*d*_6_ of 2,4‐dichloronorlichexanthone **6**.
**Figure S30:** pca70043‐sup‐0001‐Supplementary_Material.pdf. ^1^H NMR spectrum (500 MHz) in acetone‐*d*_6_ of 2,5‐dichloronorlichexanthone **7**.
**Figure S31:** HSQC NMR spectrum (500/125 MHz) in acetone‐*d*_6_ of 2,5‐dichloronorlichexanthone **7**.
**Figure S32:** HMBC NMR spectrum (500/125 MHz) in acetone‐*d*_6_ of 2,5‐dichloronorlichexanthone **7**.
**Figure S33:** NOESY NMR spectrum (500 MHz) in acetone‐*d*_6_ of 2,5‐dichloronorlichexanthone **7**.
**Figure S34:** pca70043‐sup‐0001‐Supplementary_Material.pdf. ^1^H NMR spectrum (500 MHz) in acetone‐*d*_6_ of 2,7‐dichloronorlichexanthone **8**.
**Figure S35:** Jmod NMR spectrum (125 MHz) in acetone‐*d*_6_ of 2,7‐dichloronorlichexanthone **8**.
**Figure S36:** HSQC NMR spectrum (500/125 MHz) in acetone‐*d*_6_ of 2,7‐dichloronorlichexanthone **8**.
**Figure S37:** HMBC NMR spectrum (500/125 MHz) in acetone‐*d*_6_ of 2,7‐dichloronorlichexanthone **8**.
**Figure S38:** NOESY NMR spectrum (500 MHz) in acetone‐*d*_6_ of 2,7‐dichloronorlichexanthone **8**.
**Figure S39:** pca70043‐sup‐0001‐Supplementary_Material.pdf. ^1^H NMR spectrum (500 MHz) in acetone‐*d*_6_ of 4,5‐dichloronorlichexanthone **9**.
**Figure S40:** Jmod NMR spectrum (125 MHz) in acetone‐*d*_6_ of 4,5‐dichloronorlichexanthone **9**.
**Figure S41:** HSQC NMR spectrum (500/125 MHz) in acetone‐*d*_6_ of 4,5‐dichloronorlichexanthone **9**.
**Figure S42:** HMBC NMR spectrum (500/125 MHz) in acetone‐*d*_6_ of 4,5‐dichloronorlichexanthone **9**.
**Figure S43:** NOESY NMR spectrum (500 MHz) in acetone‐*d*_6_ of 4,5‐dichloronorlichexanthone **9**.
**Figure S44:** pca70043‐sup‐0001‐Supplementary_Material.pdf. ^1^H NMR spectrum (500 MHz) in acetone‐*d*_6_ of 4,7‐dichloronorlichexanthone **10**.
**Figure S45:** Jmod NMR spectrum (125 MHz) in acetone‐*d*_6_ of 4,7‐dichloronorlichexanthone **10**.
**Figure S46:** HSQC NMR spectrum (500/125 MHz) in acetone‐*d*_6_ of 4,7‐dichloronorlichexanthone **10**.
**Figure S47:** HMBC NMR spectrum (500/125 MHz) in acetone‐*d*_6_ of 4,7‐dichloronorlichexanthone **10**.
**Figure S48:** NOESY NMR spectrum (500 MHz) in acetone‐*d*_6_ of 4,7‐dichloronorlichexanthone **10**.
**Figure S49:** pca70043‐sup‐0001‐Supplementary_Material.pdf. ^1^H NMR spectrum (500 MHz) in acetone‐*d*_6_ of 5,7‐dichloronorlichexanthone **11**.
**Figure S50:** Jmod NMR spectrum (125 MHz) in acetone‐*d*_6_ of 5,7‐dichloronorlichexanthone **11**.
**Figure S51:** HSQC NMR spectrum (500/125 MHz) in acetone‐*d*_6_ of 5,7‐dichloronorlichexanthone **11**.
**Figure S52:** HMBC NMR spectrum (500/125 MHz) in acetone‐*d*_6_ of 5,7‐dichloronorlichexanthone **11**.
**Figure S53:** NOESY NMR spectrum (500 MHz) in acetone‐*d*_6_ of 5,7‐dichloronorlichexanthone **11**.
**Figure S54:** pca70043‐sup‐0001‐Supplementary_Material.pdf. ^1^H NMR spectrum (500 MHz) in acetone‐*d*_6_ of 2,4,5‐trichloronorlichexanthone **12**.
**Figure S55:** Jmod NMR spectrum (125 MHz) in acetone‐*d*_6_ of 2,4,5‐trichloronorlichexanthone **12**.
**Figure S56:** HSQC NMR spectrum (500/125 MHz) in acetone‐*d*_6_ of 2,4,5‐trichloronorlichexanthone **12**.
**Figure S57:** HMBC NMR spectrum (500/125 MHz) in acetone‐*d*_6_ of 2,4,5‐trichloronorlichexanthone **12**.
**Figure S58:** NOESY NMR spectrum (500 MHz) in acetone‐*d*_6_ of 2,4,5‐trichloronorlichexanthone **12**.
**Figure S59:** pca70043‐sup‐0001‐Supplementary_Material.pdf. ^1^H NMR spectrum (500 MHz) in acetone‐*d*_6_ of 2,4,7‐trichloronorlichexanthone **13**.
**Figure S60:** Jmod NMR spectrum (125 MHz) in acetone‐*d*_6_ of 2,4,7‐trichloronorlichexanthone **13**.
**Figure S61:** HSQC NMR spectrum (500/125 MHz) in acetone‐*d*_6_ of 2,4,7‐trichloronorlichexanthone **13**.
**Figure S62:** HMBC NMR spectrum (500/125 MHz) in acetone‐*d*_6_ of 2,4,7‐trichloronorlichexanthone **13**.
**Figure S63:** pca70043‐sup‐0001‐Supplementary_Material.pdf. ^1^H NMR spectrum (500 MHz) in acetone‐*d*_6_ of 2,5,7‐trichloronorlichexanthone **14**.
**Figure S64:** Jmod NMR spectrum (125 MHz) in acetone‐*d*_6_ of 2,5,7‐trichloronorlichexanthone **14**.
**Figure S65:** HSQC NMR spectrum (500/125 MHz) in acetone‐*d*_6_ of 2,5,7‐trichloronorlichexanthone **14**.
**Figure S66:** HMBC NMR spectrum (500/125 MHz) in acetone‐*d*_6_ of 2,5,7‐trichloronorlichexanthone **14**.
**Figure S67:** NOESY NMR spectrum (500 MHz) in acetone‐*d*_6_ of 2,5,7‐trichloronorlichexanthone **14**.
**Figure S68:** pca70043‐sup‐0001‐Supplementary_Material.pdf. ^1^H NMR spectrum (500 MHz) in acetone‐*d*_6_ of 4,5,7‐trichloronorlichexanthone **15**.
**Figure S69:** Jmod NMR spectrum (125 MHz) in acetone‐*d*_6_ of 4,5,7‐trichloronorlichexanthone **15**.
**Figure S70:** HSQC NMR spectrum (500/125 MHz) in acetone‐*d*_6_ of 4,5,7‐trichloronorlichexanthone **15**.
**Figure S71:** HMBC NMR spectrum (500/125 MHz) in acetone‐*d*_6_ of 4,5,7‐trichloronorlichexanthone **15**.
**Figure S72:** NOESY NMR spectrum (500 MHz) in acetone‐*d*_6_ of 4,5,7‐trichloronorlichexanthone **15**.
**Figure S73:** pca70043‐sup‐0001‐Supplementary_Material.pdf. ^1^H NMR spectrum (500 MHz) in DMSO‐*d*_6_ of 2,4,5,7‐tetrachloronorlichexanthone **16**.
**Figure S74:** Jmod NMR spectrum (125 MHz) in DMSO‐*d*_6_ of 2,4,5,7‐tetrachloronorlichexanthone **16**.
**Figure S75:** HSQC NMR spectrum (500/125 MHz) in DMSO‐*d*_6_ of 2,4,5,7‐tetrachloronorlichexanthone **16**.
**Figure S76:** HMBC NMR spectrum (500/125 MHz) in DMSO‐*d*_6_ of 2,4,5,7‐tetrachloronorlichexanthone **16**.
**Figure S77:** pca70043‐sup‐0001‐Supplementary_Material.pdf. ^1^H NMR spectrum (500 MHz) in acetone‐*d*_6_ of 5‐chloroorsellinic acid **17**.
**Figure S78:** pca70043‐sup‐0001‐Supplementary_Material.pdf. ^1^H NMR spectrum (500 MHz) in acetone‐*d*_6_ of 3‐chloroorsellinic acid **18**.
**Figure S79:** pca70043‐sup‐0001‐Supplementary_Material.pdf. ^1^H NMR spectrum (500 MHz) in acetone‐*d*_6_ of 3,5‐dichloroorsellinic acid **19**.
**Figure S80:** pca70043‐sup‐0001‐Supplementary_Material.pdf. ^1^H NMR spectrum (500 MHz) in acetone‐*d*_6_ of chlorophloroglucinol **20**.
**Figure S81:** pca70043‐sup‐0001‐Supplementary_Material.pdf. ^1^H NMR spectrum (500 MHz) in acetone‐*d*_6_ of *Lecanora alboflavida* extract.
**Figure S82:** pca70043‐sup‐0001‐Supplementary_Material.pdf. ^13^C NMR spectrum (125 MHz) in acetone‐*d*_6_ of *Lecanora alboflavida* extract.
**Figure S83:** HSQC NMR spectrum (500/125 MHz) in acetone‐*d*_6_ of *Lecanora alboflavida* extract.
**Figure S84:** HSQC spectrum of *L. alboflavida* extract (^1^H between 6 and 7.2 ppm; 13C between 90 and 120 ppm).
**Figure S85:** NOESY NMR spectrum (500 MHz) in acetone‐*d*_6_ of *Lecanora alboflavida* extract.
**Figure S86:** pca70043‐sup‐0001‐Supplementary_Material.pdf. ^1^H NMR spectrum (500 MHz) in acetone‐*d*_6_ of *Myriolecis antiqua* extract.
**Figure S87:** HSQC NMR spectrum (500/125 MHz) in acetone‐*d*_6_ of *Myriolecis antiqua* extract.
**Figure S88:** NOESY NMR spectrum (500 MHz) in acetone‐*d*_6_ of *Myriolecis antiqua* extract.
**Figure S89:** pca70043‐sup‐0001‐Supplementary_Material.pdf. ^1^H NMR spectrum (500 MHz) in acetone‐*d*_6_ of *Pyrrhospora quernea* extract.
**Figure S90:** HSQC NMR spectrum (500/125 MHz) in acetone‐*d*_6_ of *Pyrrhospora quernea* extract.
**Figure S91:** NOESY NMR spectrum (500 MHz) in acetone‐*d*_6_ of *Pyrrhospora quernea* extract.
**Figure S92:** pca70043‐sup‐0001‐Supplementary_Material.pdf. ^1^H NMR spectrum (500 MHz) in acetone‐*d*_6_ of *Lecidella elaeochroma* extract.
**Figure S93:** HSQC NMR spectrum (500/125 MHz) in acetone‐*d*_6_ of *Lecidella elaeochroma* extract.
**Figure S94:** NOESY NMR spectrum (500 MHz) in acetone‐*d*_6_ of *Lecidella elaeochroma* extract.
**Figure S95:** Key NOESY correlation discriminating 3L245 from 6L245.
**Figure S96:** HMBC correlations (H ➔ C) that distinguish between 3L2457 and 6L2457.
**Figure S97:** HPLC/DAD chromatogram and extracted MS spectrum of norlichexanthone **1** (*Rt* = 6.6 min).
**Figure S98:** HPLC/DAD chromatogram and extracted MS spectrum of 2‐chloronorlichexanthone **2** (*Rt* = 10.5 min).
**Figure S99:** HPLC/DAD chromatogram and extracted MS spectrum of 4‐chloronorlichexanthone **3** (*Rt* = 9.4 min).
**Figure S100:** HPLC/DAD chromatogram and extracted MS spectrum of 5‐chloronorlichexanthone **4** (*Rt* = 8.6 min).
**Figure S101:** HPLC/DAD chromatogram and extracted MS spectrum of 7‐chloronorlichexanthone **5** (*Rt* = 12.8 min).
**Figure S102:** HPLC/DAD chromatogram and extracted MS spectrum of 2,4‐dichloronorlichexanthone **6** (*Rt* = 15.6 min).
**Figure S103:** HPLC/DAD chromatogram and extracted MS spectrum of 2,5‐dichloronorlichexanthone **7** (*Rt* = 13.4 min).
**Figure S104:** HPLC/DAD chromatogram and extracted MS spectrum of 2,7‐dichloronorlichexanthone **8** (*Rt* = 19.0 min).
**Figure S105:** HPLC/DAD chromatogram and extracted MS spectrum of 4,5‐dichloronorlichexanthone **9** (*Rt* = 12.1 min).
**Figure S106:** HPLC/DAD chromatogram and extracted MS spectrum of 4,7‐dichloronorlichexanthone **10** (*Rt* = 16.8 min).
**Figure S107:** HPLC/DAD chromatogram and extracted MS spectrum of 5,7‐dichloronorlichexanthone **11** (*Rt* = 17.4 min).
**Figure S108:** HPLC/DAD chromatogram and extracted MS spectrum of 2,4,5‐trichloronorlichexanthone **12** (*Rt* = 18.8 min).
**Figure S109:** HPLC/DAD chromatogram and extracted MS spectrum of 2,4,7‐trichloronorlichexanthone **13** (*Rt* = 26.1 min).
**Figure S110:** HPLC/DAD chromatogram and extracted MS spectrum of 2,5,7‐trichloronorlichexanthone **14** (*Rt* = 24.4 min).
**Figure S111:** HPLC/DAD chromatogram and extracted MS spectrum of 4,5,7‐trichloronorlichexanthone **15** (*Rt* = 22 min).
**Figure S112:** HPLC/DAD chromatogram and extracted MS spectrum of 2,4,5,7‐tetrachloronorlichexanthone **16** (*Rt* = 30.8 min).
**Figure S113:** Superimposition of the chromatograms of the four monochlorinated norlichexanthones (compounds **2**–**5**).
**Figure S114:** Superimposition of the chromatograms of the six dichlorinated norlichexanthones (compounds **6**–**11**).
**Figure S115:** Superimposition of the chromatograms of the four trichlorinated norlichexanthones (compounds **12**–**15**).
**Figure S116:** Separation of norlichexanthones along the lowest m/z ratio and retention time axes (*Rt* and *m/z*).
**Figure S117:** HPLC/DAD chromatogram of *L. asema* var. *elaeochromoides*.
**Figure S118:** HPLC/DAD chromatogram of *M. antiqua* .
**Figure S119:** HPLC/DAD chromatogram of *L. alboflavida* .
**Figure S120:** HPLC/DAD chromatogram of *P. quernea* .
**Figure S121:** HPLC/DAD chromatogram of *L. elaeochroma* .

## Data Availability

The data that supports the findings of this study are available in the Supporting Information of this article.

## References

[1] Analysis of Phenolic Products in Lichens for Identification and Taxonomy,” in Protocols in Lichenology (Springer, 2002), 281–295, 10.1007/978-3-642-56359-1_17.

[2] D. Olivier‐Jimenez , M. Chollet‐Krugler , D. Rondeau , et al., “A Database of High‐Resolution MS/MS Spectra for Lichen Metabolites,” Scientific Data 6, no. 1 (2019): 294, 10.1038/s41597-019-0305-1. 31780665 PMC6882832

[3] J. Bracegirdle , J. A. Elix , U. Mawalagedera , Y. H. Chooi , and C. Gueidan , “An Expanded Database of High‐Resolution MS/MS Spectra for Lichen‐Derived Natural Products,” Scientific Data 12, no. 1 (2025): 244, 10.1038/s41597-025-04488-w. 39934125 PMC11814408

[4] M. Wang , J. J. Carver , V. V. Phelan , et al., “Sharing and Community Curation of Mass Spectrometry Data With Global Natural Products Social Molecular Networking,” Nature Biotechnology 34, no. 8 (2016): 828–837, 10.1038/nbt.3597. PMC5321674 27504778

[5] K. S. Masters and S. Bräse , “Xanthones From Fungi, Lichens, and Bacteria: The Natural Products and Their Synthesis,” Chemical Reviews 112, no. 7 (2012): 3717–3776, 10.1021/cr100446h. 22617028

[6] P. Le Pogam and J. Boustie , “Xanthones of Lichen Source: A 2016 Update,” Molecules 21, no. 3 (2016): 294, 10.3390/molecules21030294. 26950106 PMC6273661

[7] R. A. Cacho , Y. H. Chooi , H. Zhou , and Y. Tang , “Complexity Generation in Fungal Polyketide Biosynthesis: A Spirocycle‐Forming P450 in the Concise Pathway to the Antifungal Drug Griseofulvin,” ACS Chemical Biology 8, no. 10 (2013): 2322–2330, 10.1021/cb400541z. 23978092 PMC3821396

[8] J. A. Elix , H. M. Chappell , and H. Jiang , “Four New Lichen Xanthones,” Bryologist 94, no. 3 (1991): 304, 10.2307/3243971.

[9] J. A. Elix and C. E. Crook , “The Joint Occurrence of Chloroxanthones in Lichens, and a Further Thirteen New Lichen Xanthones,” Bryologist 95, no. 1 (1992): 52, 10.2307/3243785.

[10] G. B. Feige , H. T. Lumbsch , S. Huneck , and J. A. Elix , “Identification of Lichen Substances by a Standardized High‐Performance Liquid Chromatographic Method,” Journal of Chromatography. A 646, no. 2 (1993): 417–427, 10.1016/0021-9673(93)83356-w.

[11] C. Leuckert and J. G. Knoph , “European Taxa of Saxicolous *Lecidella* Containing Chloroxanthones: Identification of Patterns Using Thin Layer Chromatography,” Lichenologist 24, no. 4 (1992): 383–397, 10.1017/S0024282992000501.

[12] J. Malíček , F. Berger , Z. Palice , and J. Vondrák , “Corticolous Sorediate *Lecanora* Species (Lecanoraceae, Ascomycota) Containing Atranorin in Europe,” Lichenologist 49, no. 5 (2017): 431–455, 10.1017/S002428291700038X.

[13] P. E. Eaton , G. R. Carlson , and J. T. Lee , “Phosphorus Pentoxide‐Methanesulfonic Acid. Convenient Alternative to Polyphosphoric Acid,” Journal of Organic Chemistry 38, no. 23 (1973): 4071–4073, 10.1021/jo00987a028.

[14] R. K. M. Pillai , P. Naiksatam , F. Johnson , et al., “Thermorubin II. 1,3‐Dihydroxy‐9H‐xanthones and 1,3‐Dihydroxy‐9H‐xanthenes. New Methods of Synthesis,” Journal of Organic Chemistry 51, no. 5 (1986): 717–723, 10.1021/jo00355a024.

[15] S. Moreau , M. Varache‐Lembège , S. Larrouture , et al., “(2‐Arylhydrazonomethyl)‐Substituted Xanthones as Antimycotics: Synthesis and Fungistatic Activity Against *Candida* Species,” European Journal of Medicinal Chemistry 37, no. 3 (2002): 237–253, 10.1016/S0223-5234(01)01332-0. 11900868

[16] P. K. Grover , G. D. Shah , and R. C. Shah , “Xanthones. Part IV. A New Synthesis of Hydroxyxanthones and Hydroxybenzophenones,” Journal of the Chemical Society (1955): 3982, 10.1039/jr9550003982.

[17] C. Tresse , M. François‐Heude , V. Servajean , et al., “Total Synthesis of Tiacumicin B: Study of the Challenging β‐Selective Glycosylations,” Chemistry–A European Journal 27, no. 16 (2021): 5230–5239, 10.1002/chem.202005102. 33433914

[18] L. Fitzpatrick and T. Sala , “Sargent MV. Further Total Syntheses of Chlorine‐Containing Lichen Xanthones,” Journal of the Chemical Society. Perkin Transactions (1980): 85, 10.1039/p19800000085.

[19] P. Cannon , J. Malíček , C. Ivanovich , et al., “Lecanorales: Lecanoraceae Including the Genera *Ameliella*, *Bryonora*, *Carbonea*, *Claurouxia*, *Clauzadeana*, *Glaucomaria*, *Japewia*, *Japewiella*, *Lecanora*, *Lecidella*, *Miriquidica*, *Myriolecis*, *Palicella*, *Protoparmeliopsis*, *Pyrrhospora* and *Traponora*. [“Revisions of British and Irish Lichens”],” 2022, 25.

[20] E. G. Sundholm , “Total Syntheses of Lichen Xanthones,” Tetrahedron 34, no. 5 (1978): 577–586, 10.1016/0040-4020(78)80055-6.

[21] E. G. Sundholm , K. Undheim , and O. Wennerström , “Syntheses and 13C NMR Spectra of Some 5‐Chloro‐Substituted Lichen Xanthones,” Acta Chemica Scandinavica 33b (1979): 475–482, 10.3891/acta.chem.scand.33b-0475.

[22] J. R. Laundon , “ *Lecanora antiqua* , a New Saxicolous Species From Great Britain, and the Nomenclature and Authorship of *L. albescens* , *L. conferta* and *L. muralis* ,” Lichenologist 42, no. 6 (2010): 631–636, 10.1017/S0024282910000393.

[23] J. A. Elix and T. Tønsberg , “Notes on the Chemistry of Some Lichens, Including Four Species of *Lepraria* ,” Graphis Scripta 16 (2004): 43–45.

[24] A. Zduńczyk and M. Kukwa , “A Revision of Sorediate Crustose Lichens Containing Usnic Acid and Chlorinated Xanthones in Poland,” Herzogia 27, no. 1 (2014): 13–40, 10.13158/heia.27.1.2014.13.

