# Supplementary material for: Dereplicative Combination of HPLC/DAD/MS and 2D NMR to Identify Lichexanthone Isomers in Lichen Extracts
Source: Phytochem Anal. 2025 Dec 8;37(2):330–43. doi: 10.1002/pca.70043 (PMC12961365; doi:10.1002/pca.70043)

Supplementary information

Dereplicative combination of HPLC/DAD/MS and 2D NMR to identify lichexanthone derivatives in lichen extracts

Solenn Ferron, Marylène Chollet-Krugler, Hermann Pinson, Rania Marzoug, Philippe Uriac, † Françoise Lohézic-Le Dévéhat†*

CNRS, ISCR (Institut des Sciences Chimiques de Rennes) - UMR 6226, Univ Rennes, 35000 Rennes, France.

†Contributed equally to this work

*Corresponding author (francoise.le-devehat@univ-rennes.fr)

Table of contents

1. NMR data of compounds **1-20** 7

Figure S1. ^{1}H NMR spectrum (500 MHz) in acetone- d_6 of norlichexanthone **1** 7

Figure S2. Jmod NMR spectrum (125 MHz) in acetone- d_6 of norlichexanthone **1** 8

Figure S3. HSQC NMR spectrum (500/125 MHz) in acetone- d_6 of norlichexanthone **1** 9

Figure S4. HMBC NMR spectrum (500/125 MHz) in acetone- d_6 of norlichexanthone **1** 10

Figure S5. NOESY NMR spectrum (500 MHz) in acetone- d_6 of norlichexanthone **1** 11

Figure S6. ^{1}H NMR spectrum (500 MHz) in acetone- d_6 of 2-chloronorlichexanthone **2** 12

Figure S7. Jmod NMR spectrum (125 MHz) in acetone- d_6 of 2-chloronorlichexanthone **2** 13

Figure S8. HSQC NMR spectrum (500/125 MHz) in acetone- d_6 of 2-chloronorlichexanthone **2** 14

Figure S9. HMBC NMR spectrum (500/125 MHz) in acetone- d_6 of 2-chloronorlichexanthone **2** 15

Figure S10. NOESY NMR spectrum (500 MHz) in acetone- d_6 of 2-chloronorlichexanthone **2** 16

Figure S11. ^{1}H NMR spectrum (500 MHz) in acetone- d_6 of 4-chloronorlichexanthone **3** 17

Figure S12. Jmod NMR spectrum (125 MHz) in acetone- d_6 of 4-chloronorlichexanthone **3** 18

Figure S13. HSQC NMR spectrum (500/125 MHz) in acetone- d_6 of 4-chloronorlichexanthone **3** 19

Figure S14. HMBC NMR spectrum (500/125 MHz) in acetone- d_6 of 4-chloronorlichexanthone **3** 20

Figure S15. NOESY NMR spectrum (500 MHz) in acetone- d_6 of 4-chloronorlichexanthone **3** 21

Figure S16. ^{1}H NMR spectrum (500 MHz) in acetone- d_6 of 5-chloronorlichexanthone **4** 22

Figure S17. Jmod NMR spectrum (125 MHz) in acetone- d_6 of 5-chloronorlichexanthone **4** 23

Figure S18. HSQC NMR spectrum (500/125 MHz) in acetone- d_6 of 5-chloronorlichexanthone **4** 24

Figure S19. HMBC NMR spectrum (500/125 MHz) in acetone- d_6 of 5-chloronorlichexanthone **4** 25

Figure S20. NOESY NMR spectrum (500 MHz) in acetone- d_6 of 5-chloronorlichexanthone **4** 26

Figure S21. ^{1}H NMR spectrum (500 MHz) in acetone- d_6 of 7-chloronorlichexanthone **5** 27

Figure S22. Jmod NMR spectrum (125 MHz) in acetone- d_6 of 7-chloronorlichexanthone **5** 28

Figure S23. HSQC NMR spectrum (500/125 MHz) in acetone- d_6 of 7-chloronorlichexanthone **5** 29

Figure S24. HMBC NMR spectrum (500/125 MHz) in acetone- d_6 of 7-chloronorlichexanthone **5** 30

Figure S25. ^{1}H NMR spectrum (500 MHz) in acetone- d_6 of 2,4-dichloronorlichexanthone **6** 31

Figure S26. Jmod NMR spectrum (125 MHz) in acetone- d_6 of 2,4-dichloronorlichexanthone **6** 32

Figure S27. HSQC NMR spectrum (500/125 MHz) in acetone- d_6 of 2,4-dichloronorlichexanthone **6** .. 33

Figure S28. HMBC NMR spectrum (500/125 MHz) in acetone- d_6 of 2,4-dichloronorlichexanthone **6**. 34

Figure S29. NOESY NMR spectrum (500 MHz) in acetone- d_6 of 2,4-dichloronorlichexanthone **6** 35

Figure S30. ^{1}H NMR spectrum (500 MHz) in acetone- d_6 of 2,5-dichloronorlichexanthone **7** 36

Figure S31. HSQC NMR spectrum (500/125 MHz) in acetone- d_6 of 2,5-dichloronorlichexanthone **7** .. 37

Figure S32. HMBC NMR spectrum (500/125 MHz) in acetone- d_6 of 2,5-dichloronorlichexanthone **7**. 38

Figure S33. NOESY NMR spectrum (500 MHz) in acetone- d_6 of 2,5-dichloronorlichexanthone **7** 39

Figure S34. ^{1}H NMR spectrum (500 MHz) in acetone- d_6 of 2,7-dichloronorlichexanthone **8** 40

Figure S35. Jmod NMR spectrum (125 MHz) in acetone- d_6 of 2,7-dichloronorlichexanthone **8** 41

Figure S36. HSQC NMR spectrum (500/125 MHz) in acetone- d_6 of 2,7-dichloronorlichexanthone **8** .. 42
Figure S37. HMBC NMR spectrum (500/125 MHz) in acetone- d_6 of 2,7-dichloronorlichexanthone **8** . 43
Figure S38. NOESY NMR spectrum (500 MHz) in acetone- d_6 of 2,7-dichloronorlichexanthone **8** 44
Figure S39. ^{1}H NMR spectrum (500 MHz) in acetone- d_6 of 4,5-dichloronorlichexanthone **9** 45
Figure S40. Jmod NMR spectrum (125 MHz) in acetone- d_6 of 4,5-dichloronorlichexanthone **9** 46
Figure S41. HSQC NMR spectrum (500/125 MHz) in acetone- d_6 of 4,5-dichloronorlichexanthone **9** .. 47
Figure S42. HMBC NMR spectrum (500/125 MHz) in acetone- d_6 of 4,5-dichloronorlichexanthone **9** . 48
Figure S43. NOESY NMR spectrum (500 MHz) in acetone- d_6 of 4,5-dichloronorlichexanthone **9** 49
Figure S44. ^{1}H NMR spectrum (500 MHz) in acetone- d_6 of 4,7-dichloronorlichexanthone **10** 50
Figure S45. Jmod NMR spectrum (125 MHz) in acetone- d_6 of 4,7-dichloronorlichexanthone **10** 51
Figure S46. HSQC NMR spectrum (500/125 MHz) in acetone- d_6 of 4,7-dichloronorlichexanthone **10** 52
Figure S47. HMBC NMR spectrum (500/125 MHz) in acetone- d_6 of 4,7-dichloronorlichexanthone **10** 53
Figure S48. NOESY NMR spectrum (500 MHz) in acetone- d_6 of 4,7-dichloronorlichexanthone **10** 54
Figure S49. ^{1}H NMR spectrum (500 MHz) in acetone- d_6 of 5,7-dichloronorlichexanthone **11** 55
Figure S50. Jmod NMR spectrum (125 MHz) in acetone- d_6 of 5,7-dichloronorlichexanthone **11** 56
Figure S51. HSQC NMR spectrum (500/125 MHz) in acetone- d_6 of 5,7-dichloronorlichexanthone **11** 57
Figure S52. HMBC NMR spectrum (500/125 MHz) in acetone- d_6 of 5,7-dichloronorlichexanthone **11** 58
Figure S53. NOESY NMR spectrum (500 MHz) in acetone- d_6 of 5,7-dichloronorlichexanthone **11** 59
Figure S54. ^{1}H NMR spectrum (500 MHz) in acetone- d_6 of 2,4,5-trichloronorlichexanthone **12** 60
Figure S55. Jmod NMR spectrum (125 MHz) in acetone- d_6 of 2,4,5-trichloronorlichexanthone **12** 61
Figure S56. HSQC NMR spectrum (500/125 MHz) in acetone- d_6 of 2,4,5-trichloronorlichexanthone **12** 62
Figure S57. HMBC NMR spectrum (500/125 MHz) in acetone- d_6 of 2,4,5-trichloronorlichexanthone **12** 63
Figure S58. NOESY NMR spectrum (500 MHz) in acetone- d_6 of 2,4,5-trichloronorlichexanthone **12** . 64
Figure S59. ^{1}H NMR spectrum (500 MHz) in acetone- d_6 of 2,4,7-trichloronorlichexanthone **13** 65
Figure S60. Jmod NMR spectrum (125 MHz) in acetone- d_6 of 2,4,7-trichloronorlichexanthone **13** 66
Figure S61. HSQC NMR spectrum (500/125 MHz) in acetone- d_6 of 2,4,7-trichloronorlichexanthone **13** 67
Figure S62. HMBC NMR spectrum (500/125 MHz) in acetone- d_6 of 2,4,7-trichloronorlichexanthone **13** 68
Figure S63. ^{1}H NMR spectrum (500 MHz) in acetone- d_6 of 2,5,7-trichloronorlichexanthone **14** 69
Figure S64. Jmod NMR spectrum (125 MHz) in acetone- d_6 of 2,5,7-trichloronorlichexanthone **14** 70
Figure S65. HSQC NMR spectrum (500/125 MHz) in acetone- d_6 of 2,5,7-trichloronorlichexanthone **14** 71
Figure S66. HMBC NMR spectrum (500/125 MHz) in acetone- d_6 of 2,5,7-trichloronorlichexanthone **14** 72
Figure S67. NOESY NMR spectrum (500 MHz) in acetone- d_6 of 2,5,7-trichloronorlichexanthone **14** . 73

Figure S68. ^{1}H NMR spectrum (500 MHz) in acetone- d_6 of 4,5,7-trichloronorlichexanthone **15**.......... 74
Figure S69. Jmod NMR spectrum (125 MHz) in acetone- d_6 of 4,5,7-trichloronorlichexanthone **15** 75
Figure S70. HSQC NMR spectrum (500/125 MHz) in acetone- d_6 of 4,5,7-trichloronorlichexanthone **15** 76
Figure S71. HMBC NMR spectrum (500/125 MHz) in acetone- d_6 of 4,5,7-trichloronorlichexanthone **15** 77
Figure S72. NOESY NMR spectrum (500 MHz) in acetone- d_6 of 4,5,7-trichloronorlichexanthone **15**. 78
Figure S73. ^{1}H NMR spectrum (500 MHz) in DMSO- d_6 of 2,4,5,7-tetrachloronorlichexanthone **16**.... 79
Figure S74. Jmod NMR spectrum (125 MHz) in DMSO - d_6 of 2,4,5,7-tetrachloronorlichexanthone **16** 80
Figure S75. HSQC NMR spectrum (500/125 MHz) in DMSO - d_6 of 2,4,5,7-tetrachloronorlichexanthone **16** 81
Figure S76. HMBC NMR spectrum (500/125 MHz) in DMSO - d_6 of 2,4,5,7-tetrachloronorlichexanthone **16** 82
Figure S77. ^{1}H NMR spectrum (500 MHz) in acetone- d_6 of 5-chloroorsellinic acid **17**........ 83
Figure S78. ^{1}H NMR spectrum (500 MHz) in acetone- d_6 of 3-chloroorsellinic acid **18**........ 84
Figure S79. ^{1}H NMR spectrum (500 MHz) in acetone- d_6 of 3,5-dichloroorsellinic acid **19**........ 85
Figure S80. ^{1}H NMR spectrum (500 MHz) in acetone- d_6 of chlorophloroglucinol **20**........ 86
2. NMR analysis of lichens extracts 87
Figure S81. ^{1}H NMR spectrum (500 MHz) in acetone- d_6 of *Lecanora alboflavida* extract 87
Figure S82. ^{13}C NMR spectrum (125 MHz) in acetone-d_6 of *Lecanora alboflavida* extract 88
Figure S83. HSQC NMR spectrum (500/125 MHz) in acetone- d_6 of *Lecanora alboflavida* extract...... 89
Figure S84. HSQC spectrum of *L. alboflavida* extract (^{1}H between 6 and 7.2 ppm; ^{13}C between 90 and 120 ppm). 90
Figure S85. NOESY NMR spectrum (500 MHz) in acetone- d_6 of *Lecanora alboflavida* extract 91
Figure S86. ^{1}H NMR spectrum (500 MHz) in acetone- d_6 of *Myriolecis antiqua* extract 92
Figure S87. HSQC NMR spectrum (500/125 MHz) in acetone- d_6 of *Myriolecis antiqua* extract.......... 93
Figure S88. NOESY NMR spectrum (500 MHz) in acetone- d_6 of *Myriolecis antiqua* extract 94
Figure S89. ^{1}H NMR spectrum (500 MHz) in acetone- d_6 of *Pyrrhospora quernea* extract 95
Figure S90. HSQC NMR spectrum (500/125 MHz) in acetone- d_6 of *Pyrrhospora quernea* extract...... 96
Figure S91. NOESY NMR spectrum (500 MHz) in acetone- d_6 of *Pyrrhospora quernea* extract 97
Figure S92. ^{1}H NMR spectrum (500 MHz) in acetone- d_6 of *Lecidella elaeochroma* extract 98
Figure S93. HSQC NMR spectrum (500/125 MHz) in acetone- d_6 of *Lecidella elaeochroma* extract.... 99
Figure S94. NOESY NMR spectrum (500 MHz) in acetone- d_6 of *Lecidella elaeochroma* extract...... 100
Figure S95. Key NOESY correlation discriminating 3L245 from 6L245........ 101
Figure S96. HMBC correlations (H→ C) that distinguish between 3L2457 and 6L2457 101
3. Chromatographic data of compounds **1-16** 102
Figure S97. HPLC/DAD chromatogram and extracted MS spectrum of norlichexanthone **1** (*Rt*= 6.6 min) 102

Figure S98. HPLC/DAD chromatogram and extracted MS spectrum of 2-chloronorlichexanthone **2** (*Rt* = 10.5 min) 102

Figure S99. HPLC/DAD chromatogram and extracted MS spectrum of 4-chloronorlichexanthone **3** (*Rt* = 9.4 min) 103

Figure S100. HPLC/DAD chromatogram and extracted MS spectrum of 5-chloronorlichexanthone **4** (*Rt* = 8.6 min) 103

Figure S101. HPLC/DAD chromatogram and extracted MS spectrum of 7-chloronorlichexanthone **5** (*Rt* = 12.8 min) 104

Figure S102. HPLC/DAD chromatogram and extracted MS spectrum of 2,4-dichloronorlichexanthone **6** (*Rt* = 15.6 min) 104

Figure S103. HPLC/DAD chromatogram and extracted MS spectrum of 2,5-dichloronorlichexanthone **7** (*Rt* = 13.4 min) 105

Figure S104. HPLC/DAD chromatogram and extracted MS spectrum of 2,7-dichloronorlichexanthone **8** (*Rt* = 19.0 min) 105

Figure S105. HPLC/DAD chromatogram and extracted MS spectrum of 4,5-dichloronorlichexanthone **9** (*Rt* = 12.1 min) 106

Figure S106. HPLC/DAD chromatogram and extracted MS spectrum of 4,7-dichloronorlichexanthone **10** (*Rt* = 16.8 min) 106

Figure S107. HPLC/DAD chromatogram and extracted MS spectrum of 5,7-dichloronorlichexanthone **11** (*Rt* = 17.4 min) 107

Figure S108. HPLC/DAD chromatogram and extracted MS spectrum of 2,4,5-trichloronorlichexanthone **12** (*Rt* = 18.8 min) 107

Figure S109. HPLC/DAD chromatogram and extracted MS spectrum of 2,4,7-trichloronorlichexanthone **13** (*Rt* = 26.1 min) 108

Figure S110. HPLC/DAD chromatogram and extracted MS spectrum of 2,5,7-trichloronorlichexanthone **14** (*Rt* = 24.4 min) 108

Figure S111. HPLC/DAD chromatogram and extracted MS spectrum of 4,5,7-trichloronorlichexanthone **15** (*Rt* = 22 min) 109

Figure S112. HPLC/DAD chromatogram and extracted MS spectrum of 2,4,5,7-tetrachloronorlichexanthone **16** (*Rt* = 30.8 min) 109

Figure S113. Superimposition of the chromatograms of the four monochlorinated norlichexanthones (compounds **2-5**) 110

Figure S114. Superimposition of the chromatograms of the six dichlorinated norlichexanthones (compounds **6-11**) 110

Figure S115. Superimposition of the chromatograms of the four trichlorinated norlichexanthones (compounds **12-15**) 110

Figure S116. Separation of norlichexanthones along the lowest m/z ratio and retention time axes (Rt and m/z) 111

4. HPLC/DAD chromatograms of lichens extracts 111

Figure S117. HPLC/DAD chromatogram of *L. asema* var. *elaeochromoides* 111

Figure S118. HPLC/DAD chromatogram of *M. antiqua* 112

Figure S119. HPLC/DAD chromatogram of *L. alboflavida* 112

Figure S120. HPLC/DAD chromatogram of *P. quernea* 112
Figure S121. HPLC/DAD chromatogram of *L. elaeochroma* 113

1. NMR data of compounds **1-20**

Figure S1. ^{1}H NMR spectrum (500 MHz) in acetone- d_6 of norlichexanthone **1**

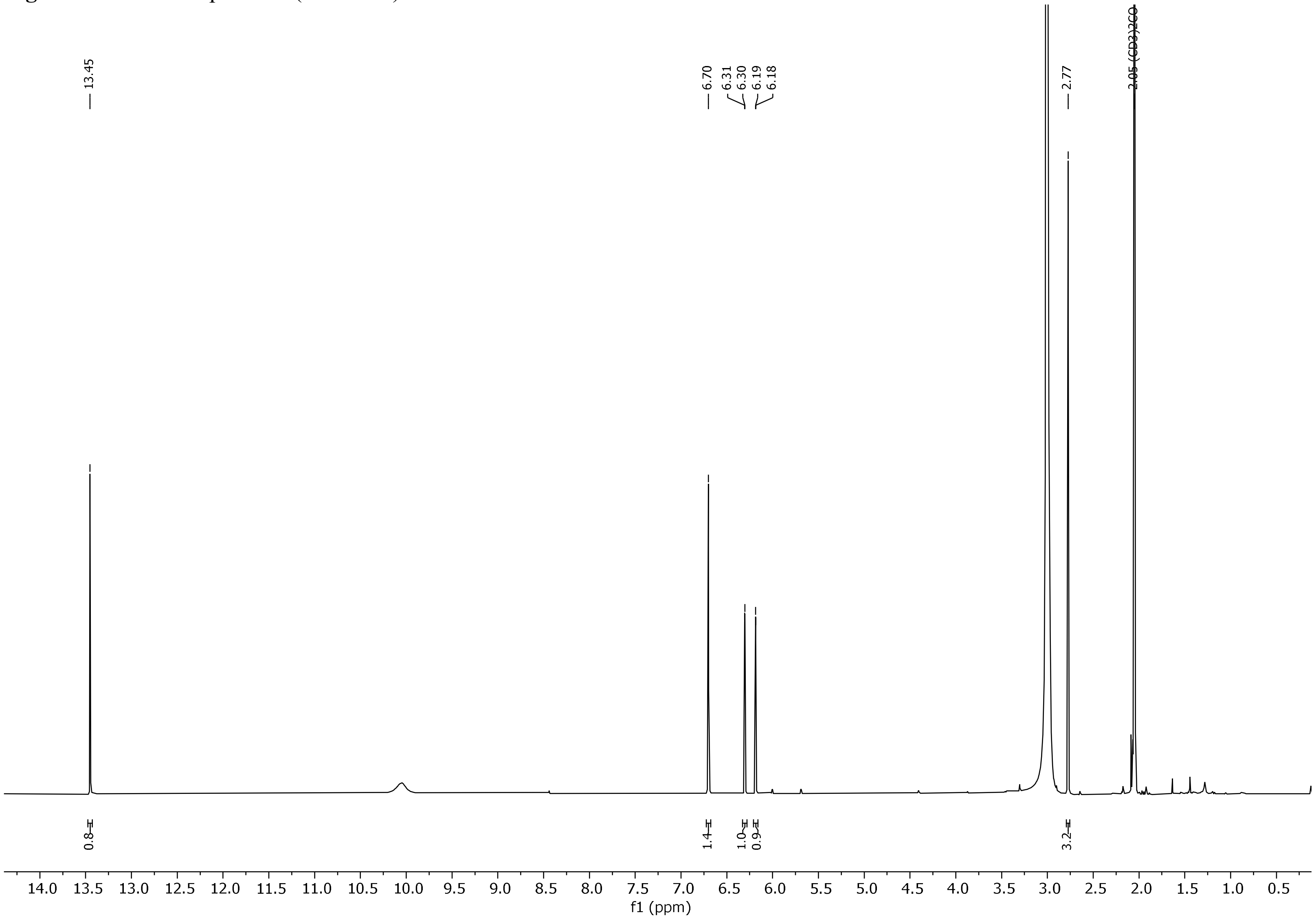

Figure S2. Jmod NMR spectrum (125 MHz) in acetone- d_6 of norlichexanthone **1**

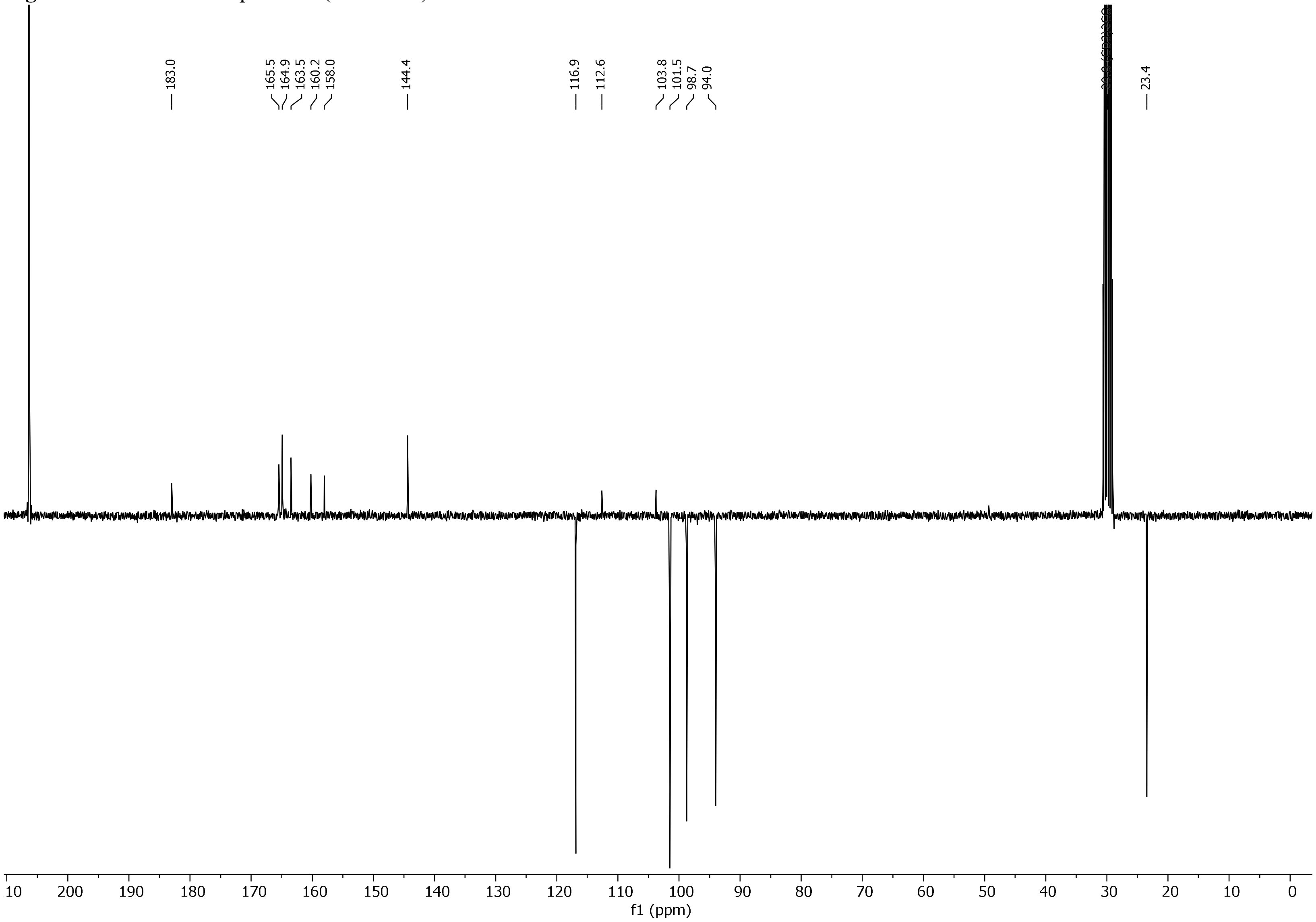

Figure S3. HSQC NMR spectrum (500/125 MHz) in acetone- d_6 of norlichexanthone **1**

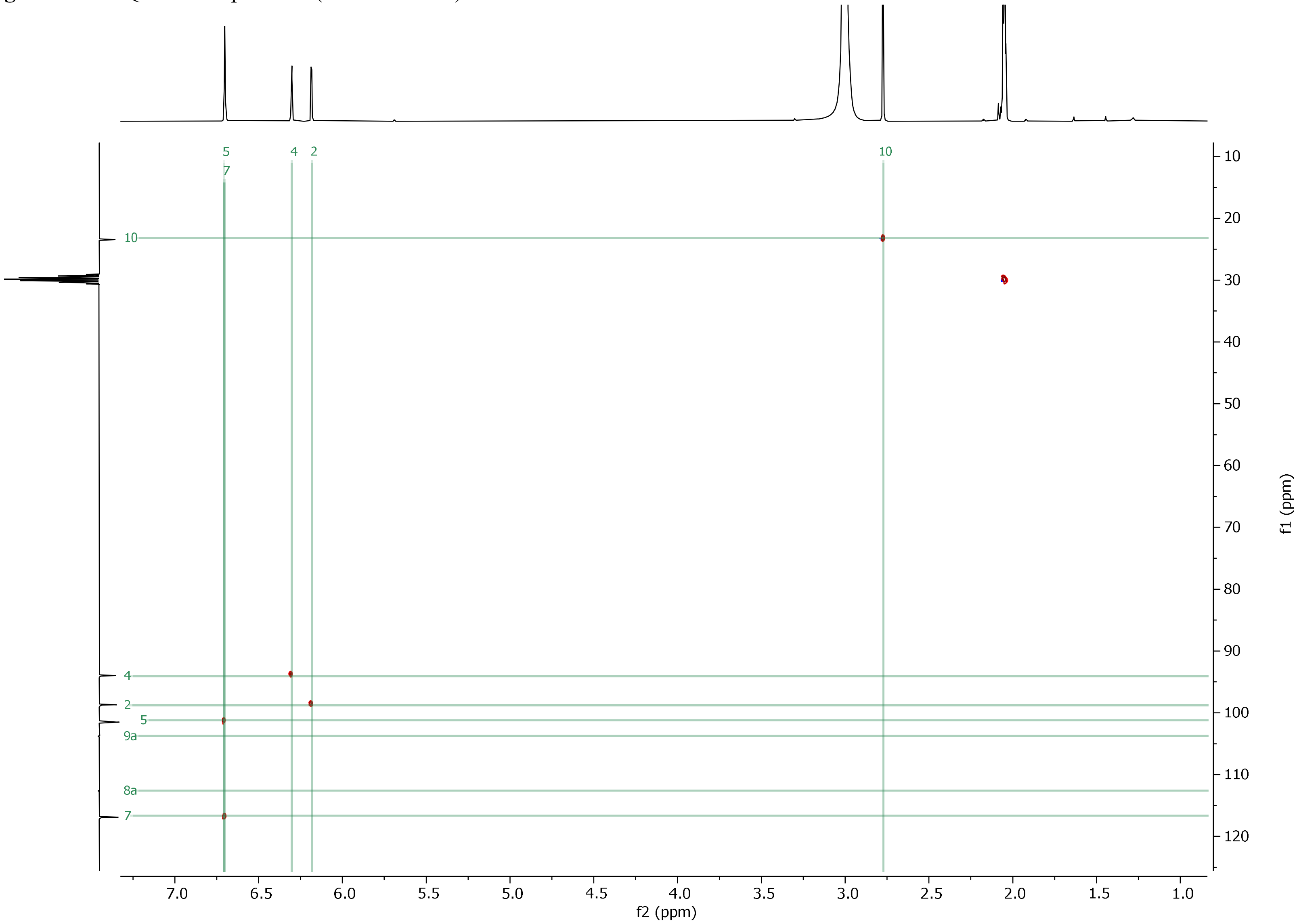

Figure S4. HMBC NMR spectrum (500/125 MHz) in acetone- d_6 of norlichexanthone **1**

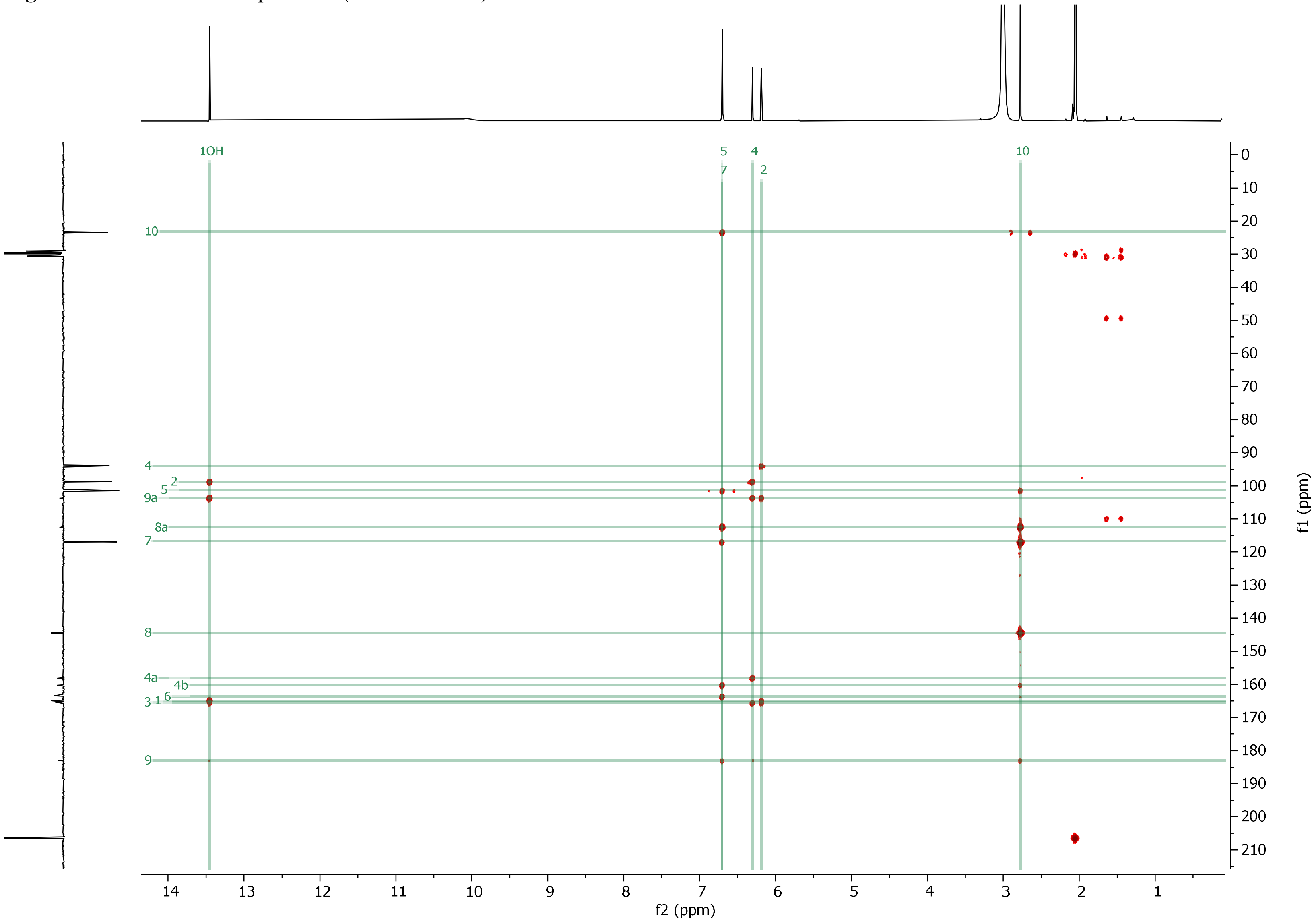

Figure S5. NOESY NMR spectrum (500 MHz) in acetone- d_6 of norlichexanthone **1**

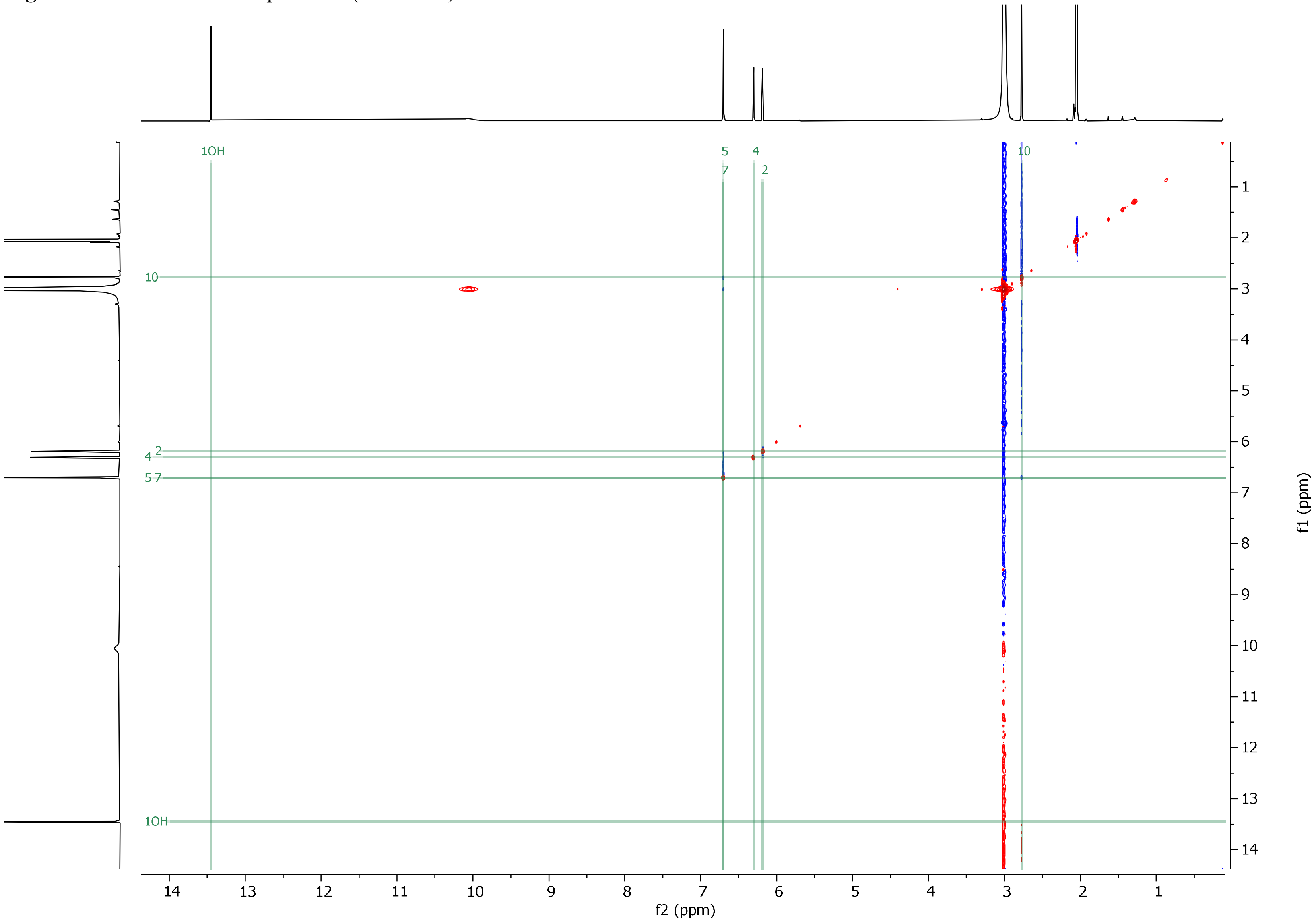

Figure S6. ^{1}H NMR spectrum (500 MHz) in acetone- d_6 of 2-chloronorlichexanthone **2**

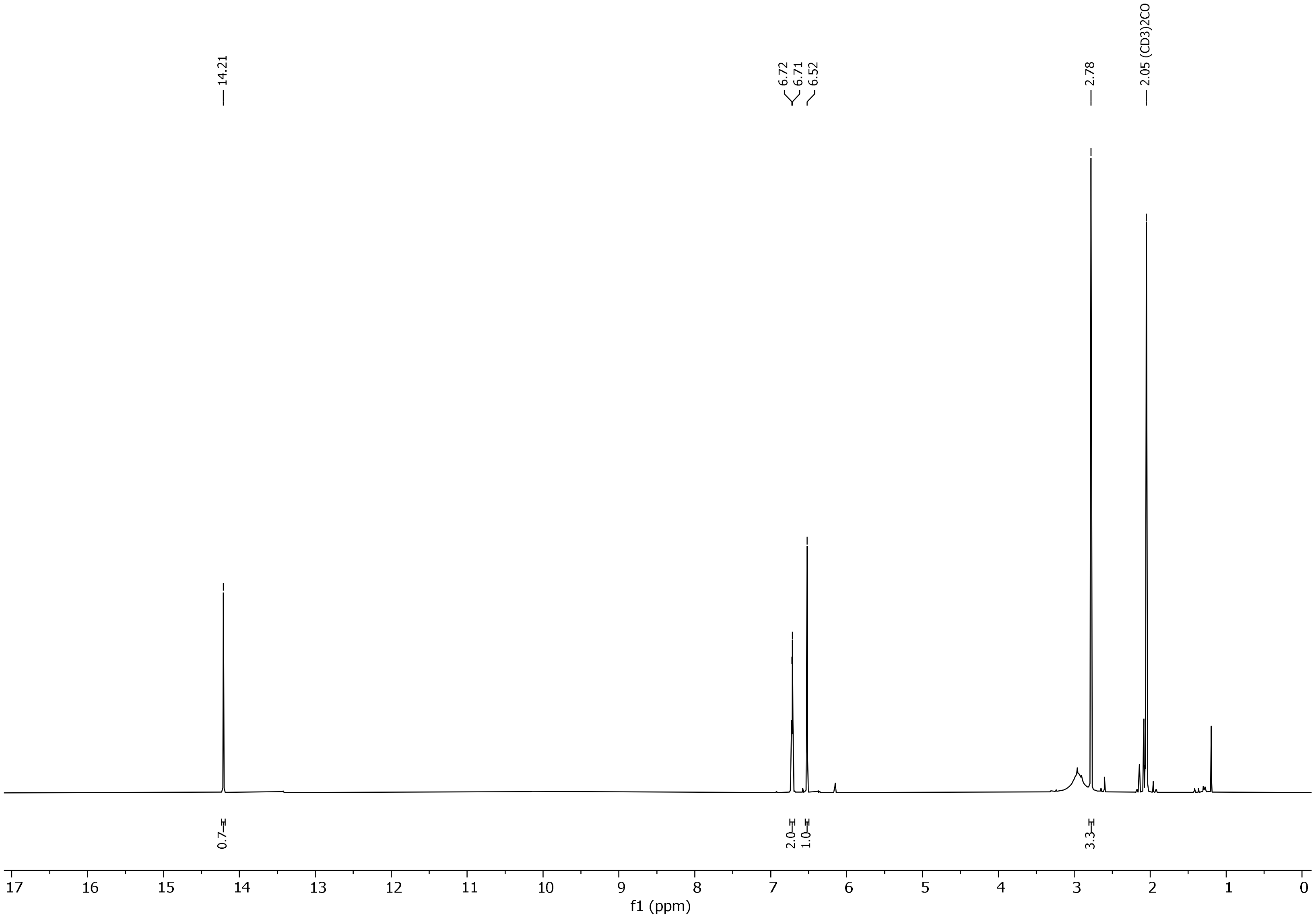

Figure S7. Jmod NMR spectrum (125 MHz) in acetone- d_6 of 2-chloronorlichexanthone **2**

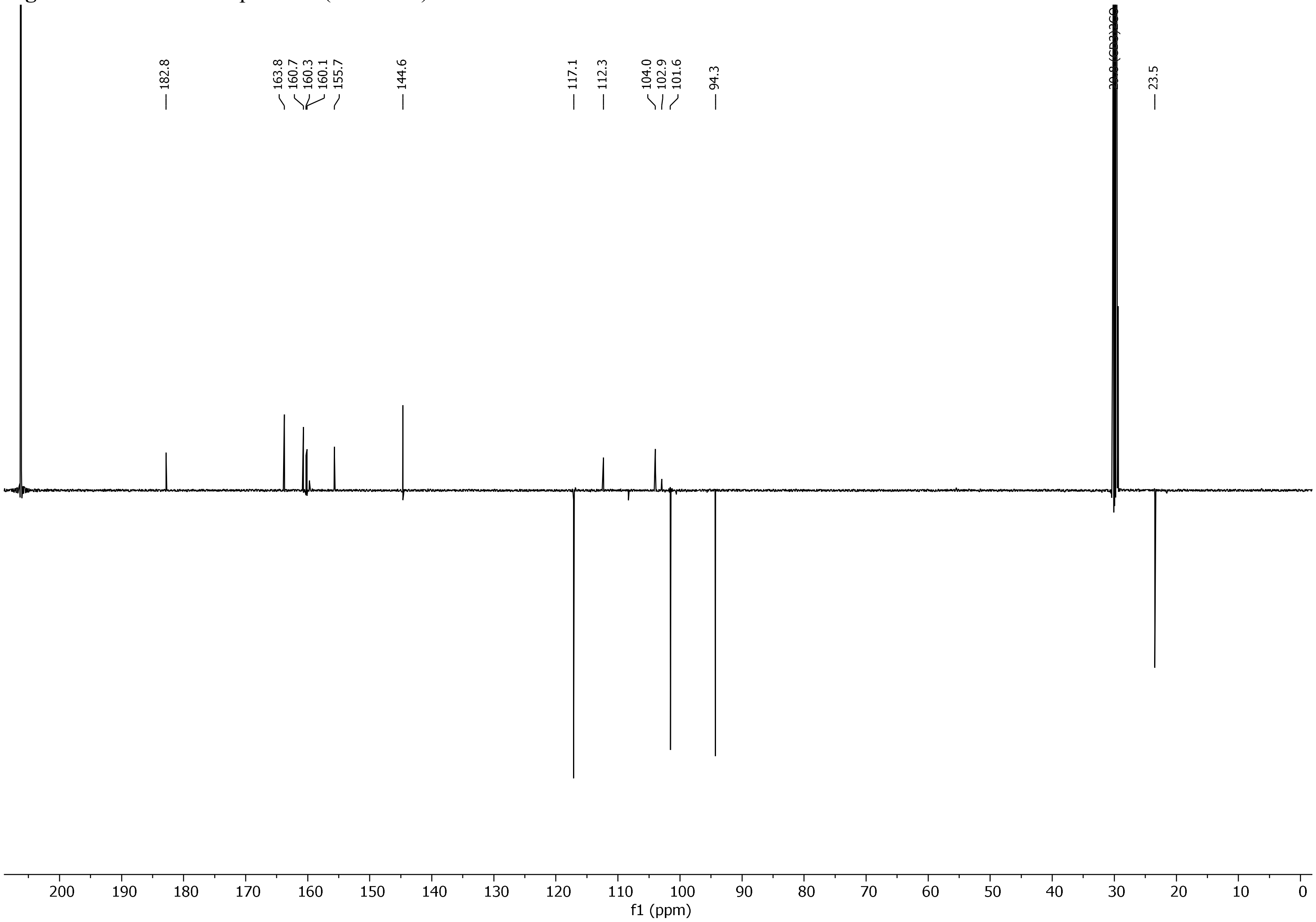

Figure S8. HSQC NMR spectrum (500/125 MHz) in acetone- d_6 of 2-chloronorlichexanthone **2**

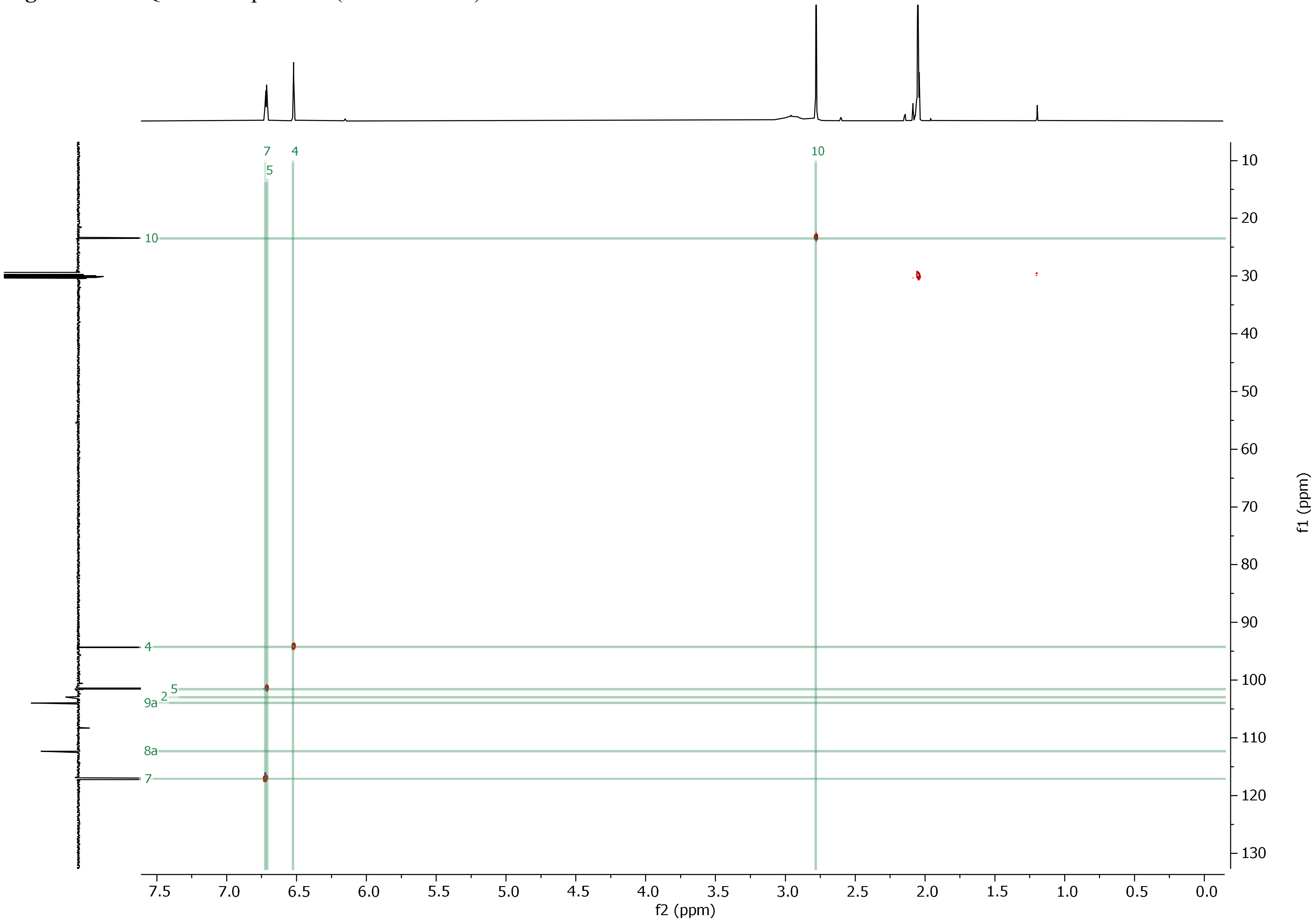

Figure S9. HMBC NMR spectrum (500/125 MHz) in acetone- d_6 of 2-chloronorlichexanthone **2**

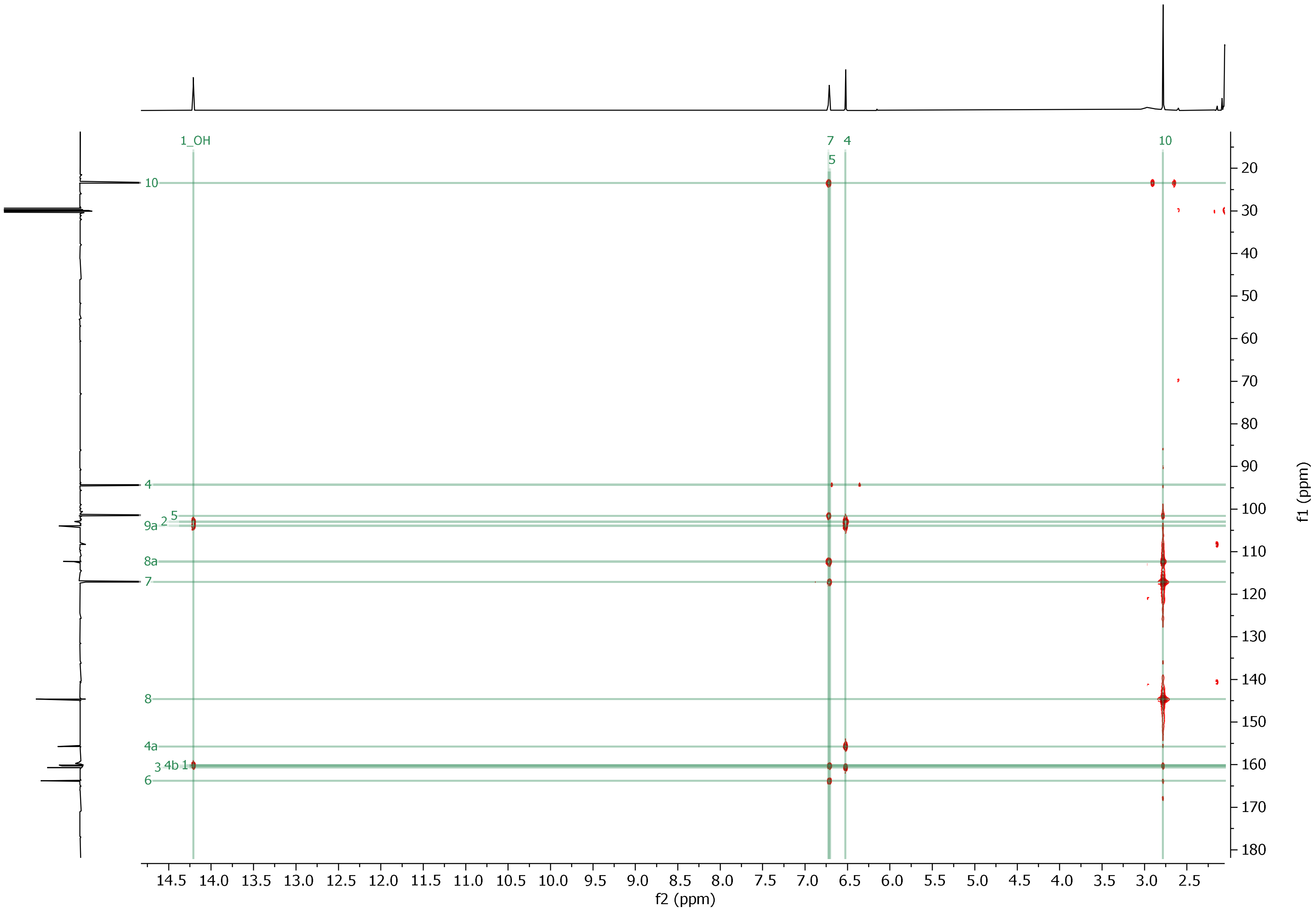

Figure S10. NOESY NMR spectrum (500 MHz) in acetone- d_6 of 2-chloronorlichexanthone **2**

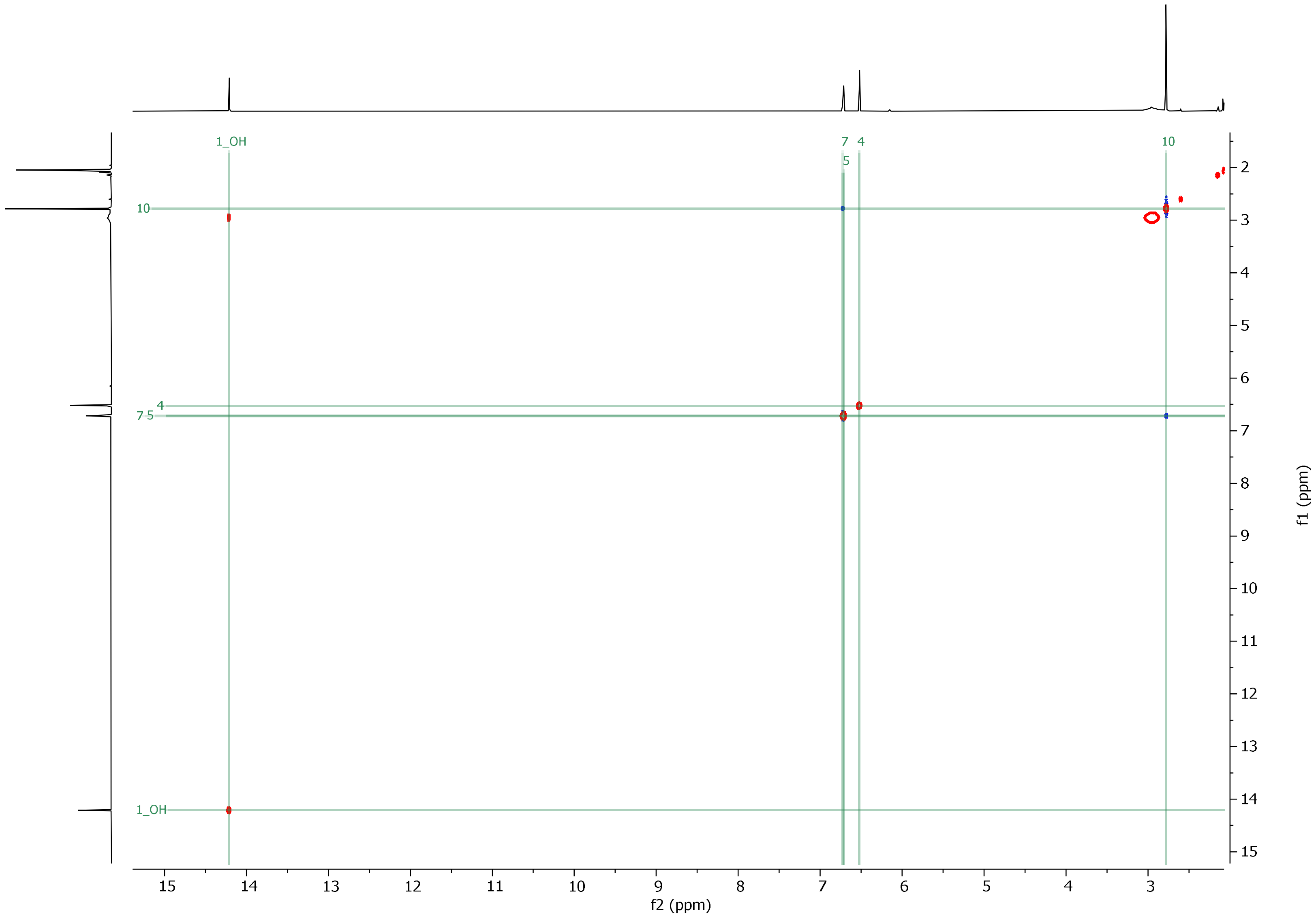

Figure S11. ^{1}H NMR spectrum (500 MHz) in acetone- d_6 of 4-chloronorlichexanthone **3**

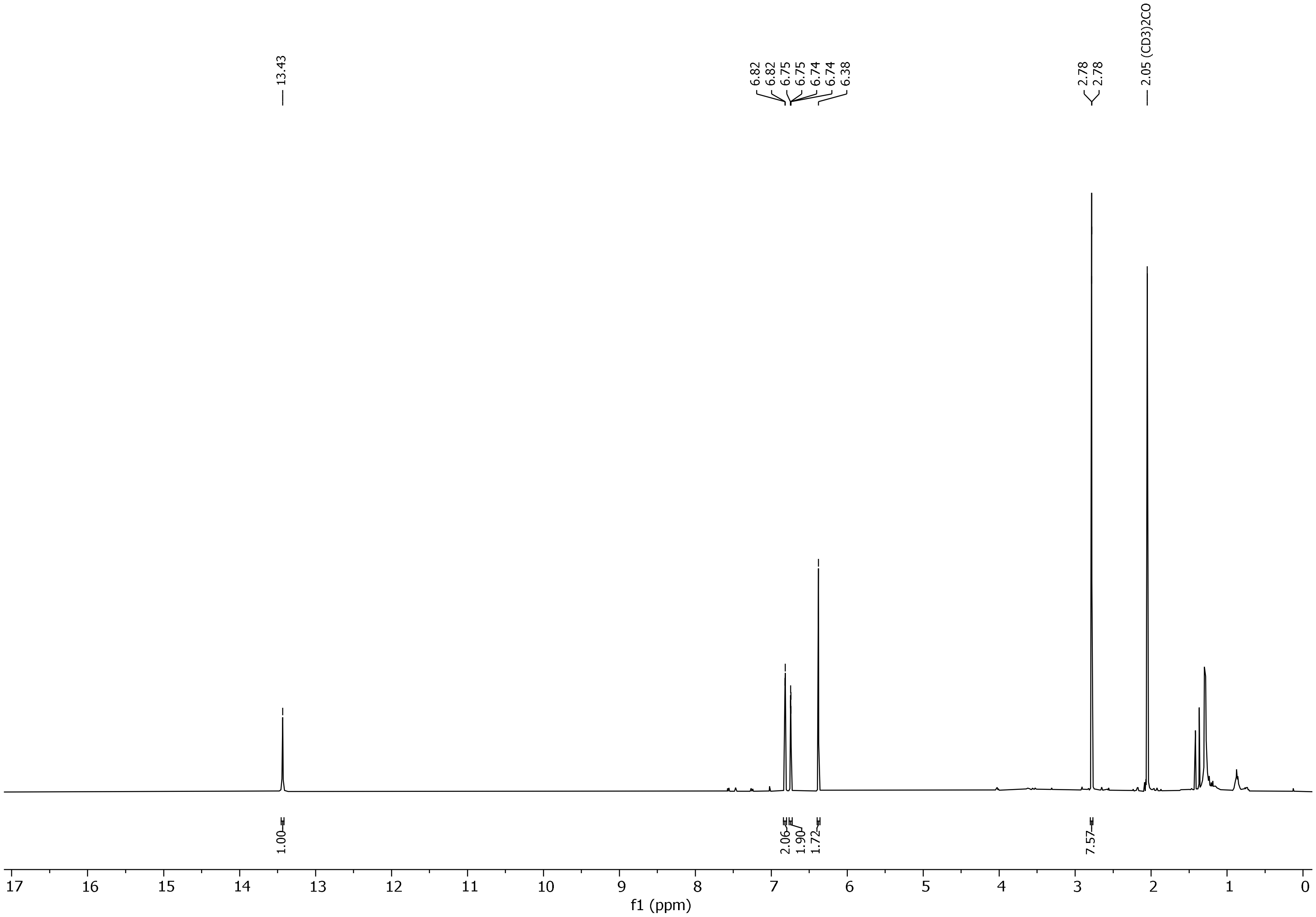

Figure S12. Jmod NMR spectrum (125 MHz) in acetone- d_6 of 4-chloronorlichexanthone **3**

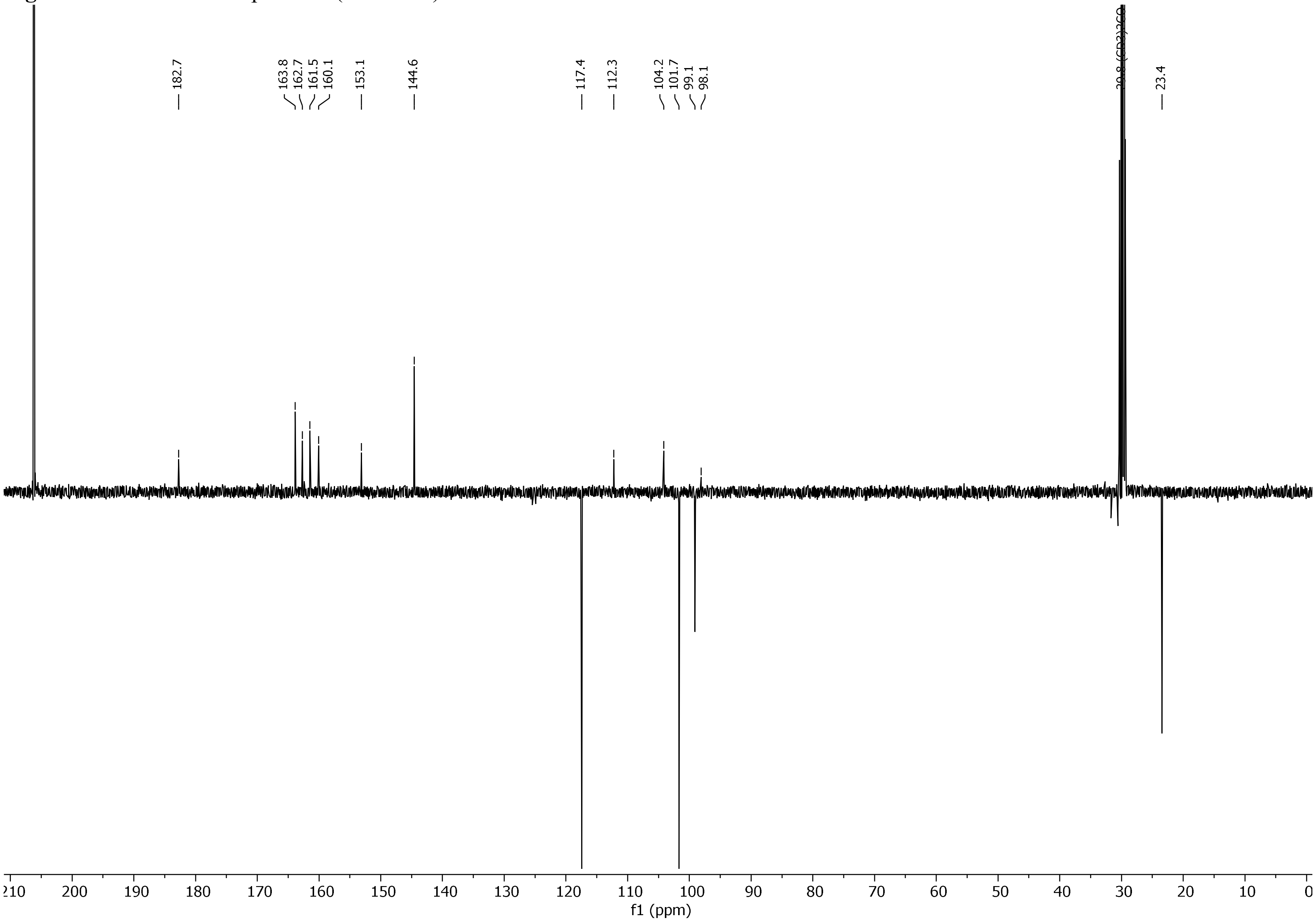

Figure S13. HSQC NMR spectrum (500/125 MHz) in acetone- d_6 of 4-chloronorlichexanthone **3**

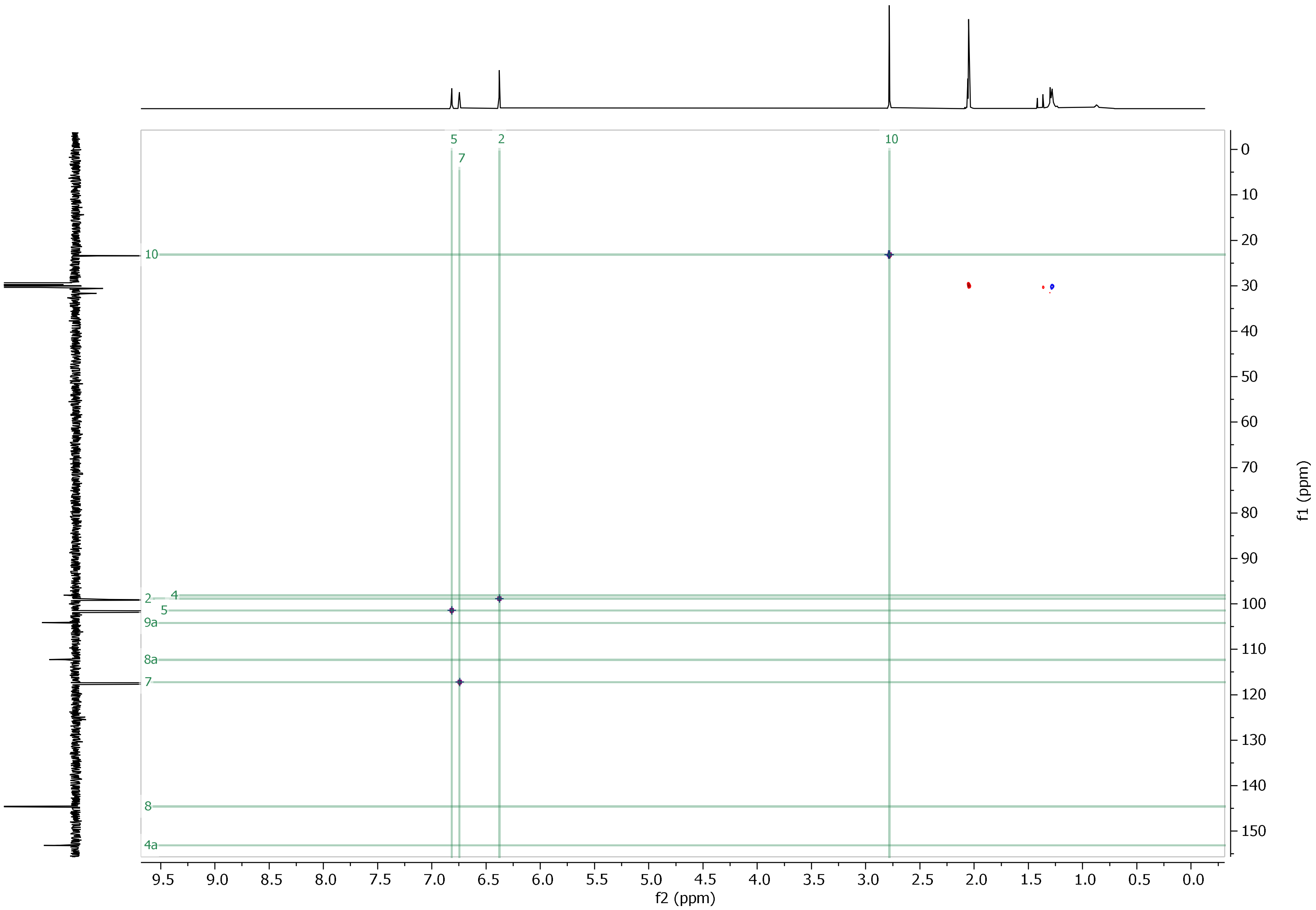

Figure S14. HMBC NMR spectrum (500/125 MHz) in acetone- d_6 of 4-chloronorlichexanthone **3**

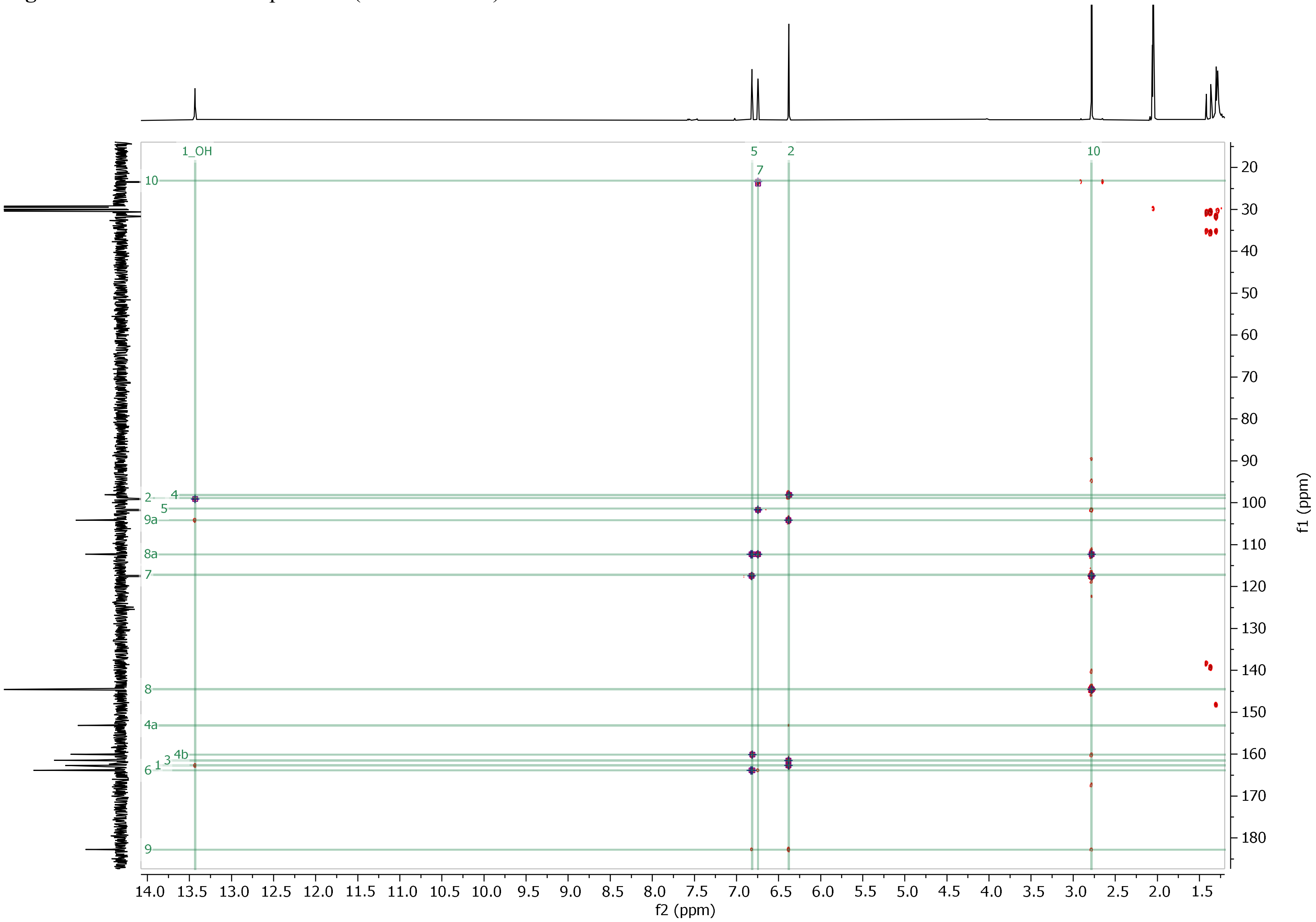

Figure S15. NOESY NMR spectrum (500 MHz) in acetone- d_6 of 4-chloronorlichexanthone **3**

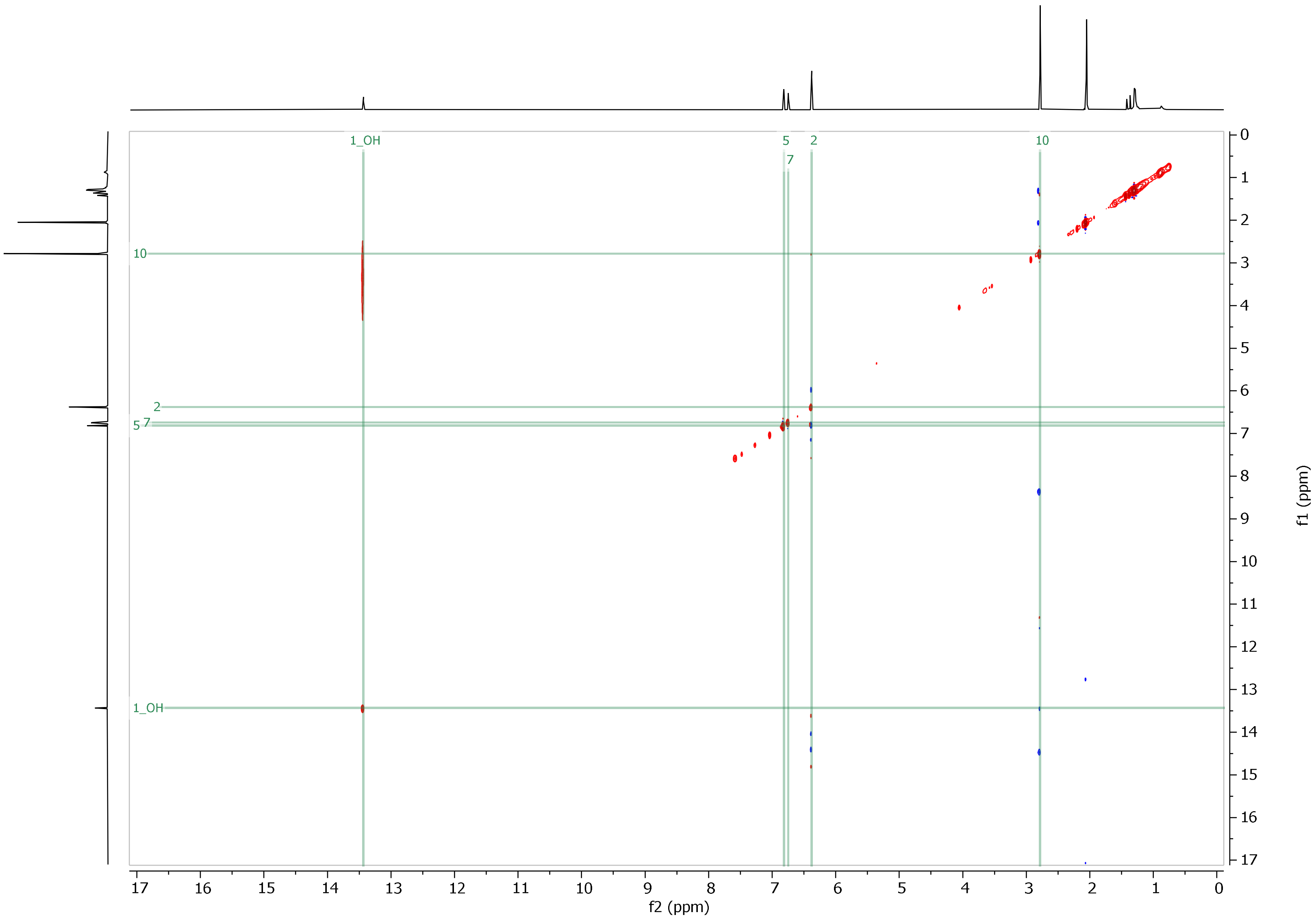

Figure S16. ^{1}H NMR spectrum (500 MHz) in acetone- d_6 of 5-chloronorlichexanthone **4**

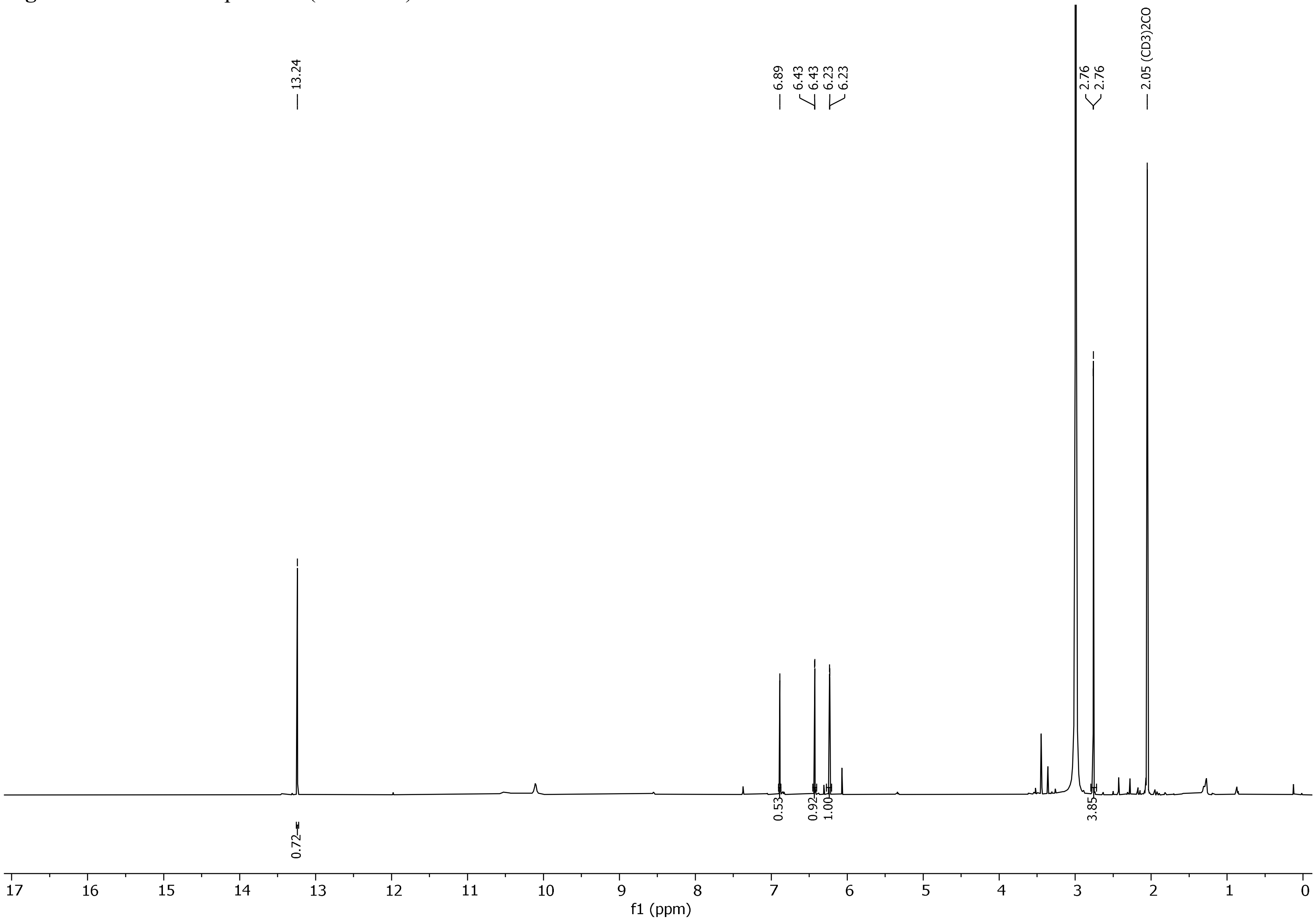

Figure S17. Jmod NMR spectrum (125 MHz) in acetone- d_6 of 5-chloronorlichexanthone **4**

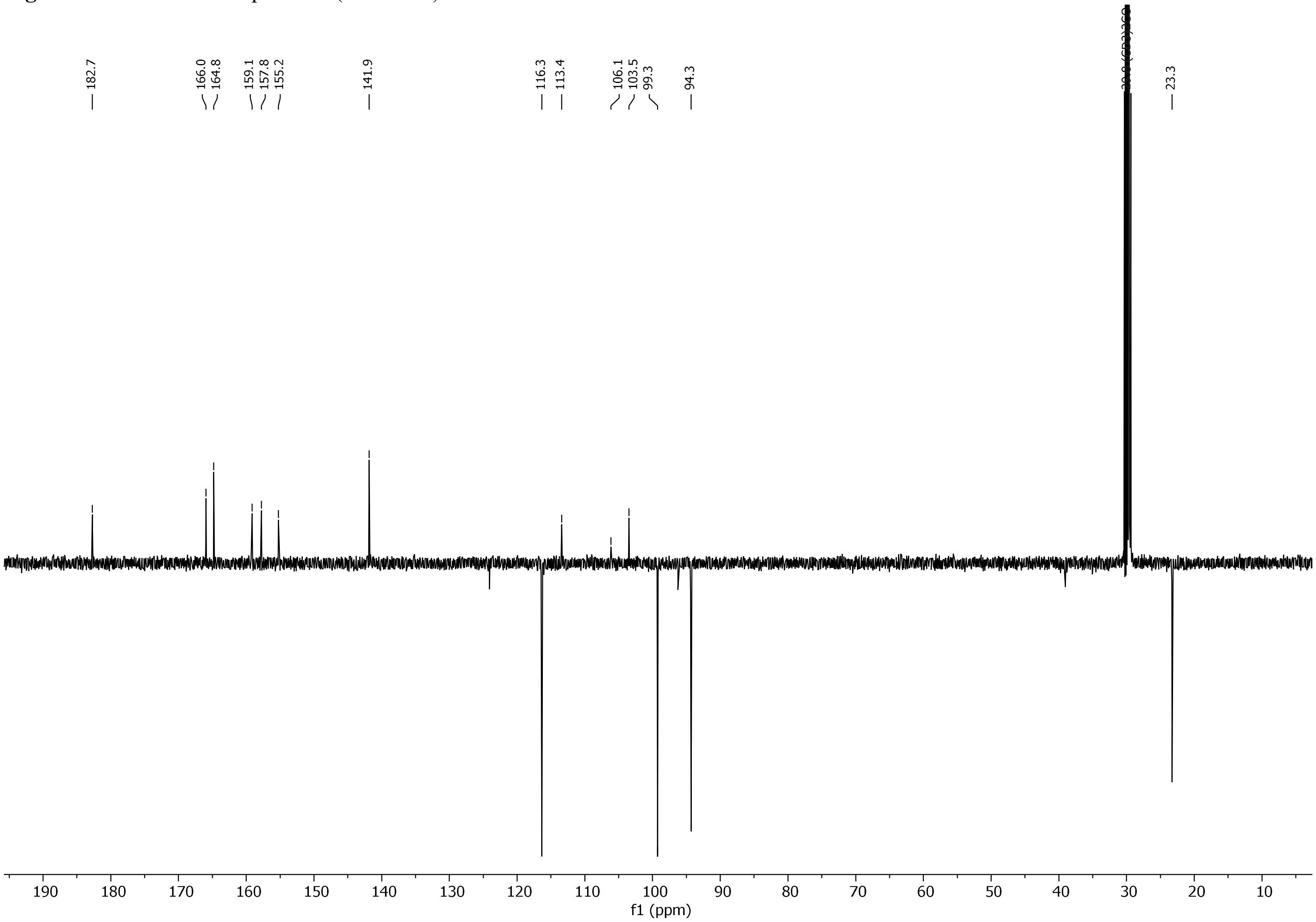

Figure S18. HSQC NMR spectrum (500/125 MHz) in acetone- d_6 of 5-chloronorlichexanthone **4**

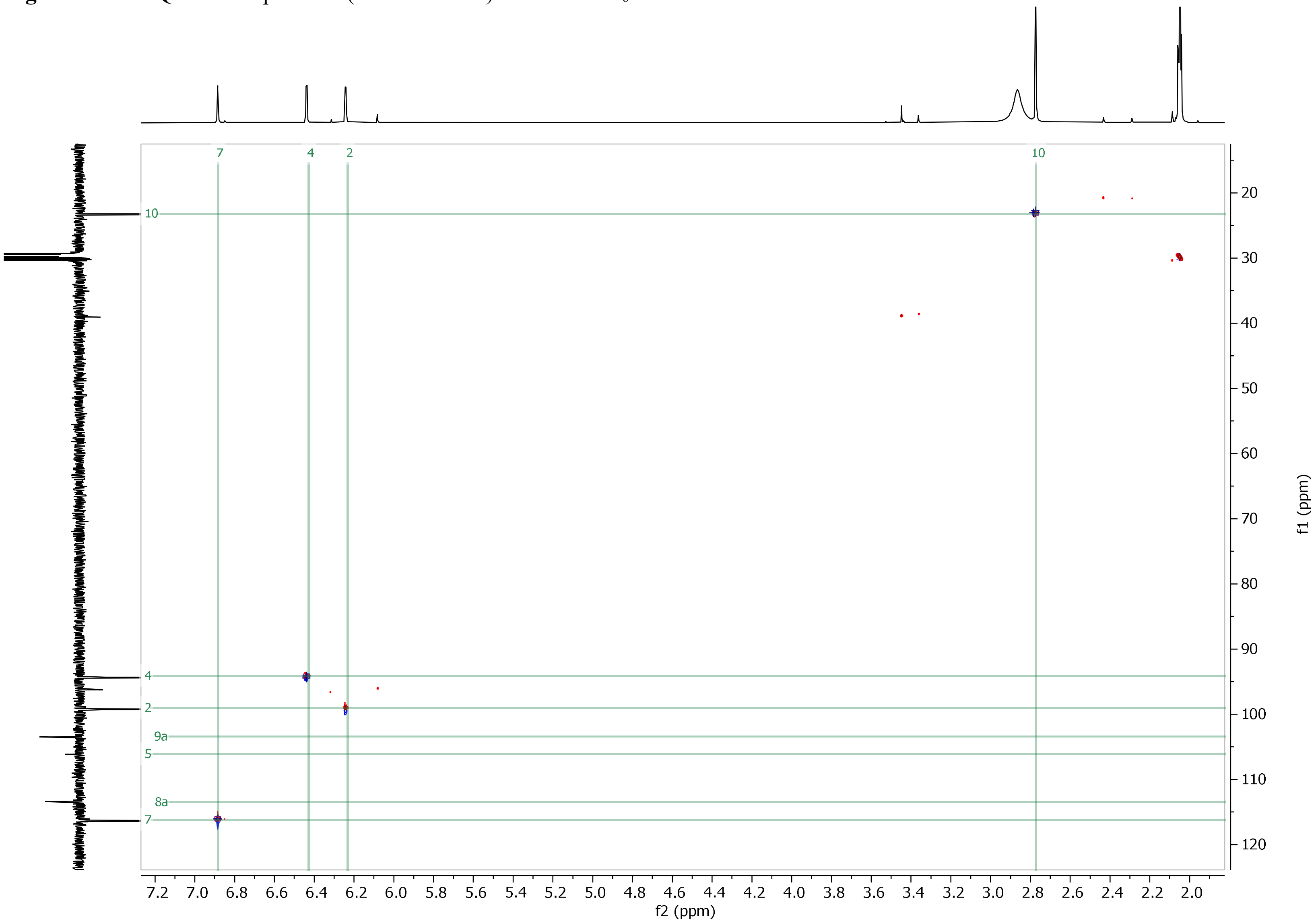

Figure S19. HMBC NMR spectrum (500/125 MHz) in acetone- d_6 of 5-chloronorlichexanthone **4**

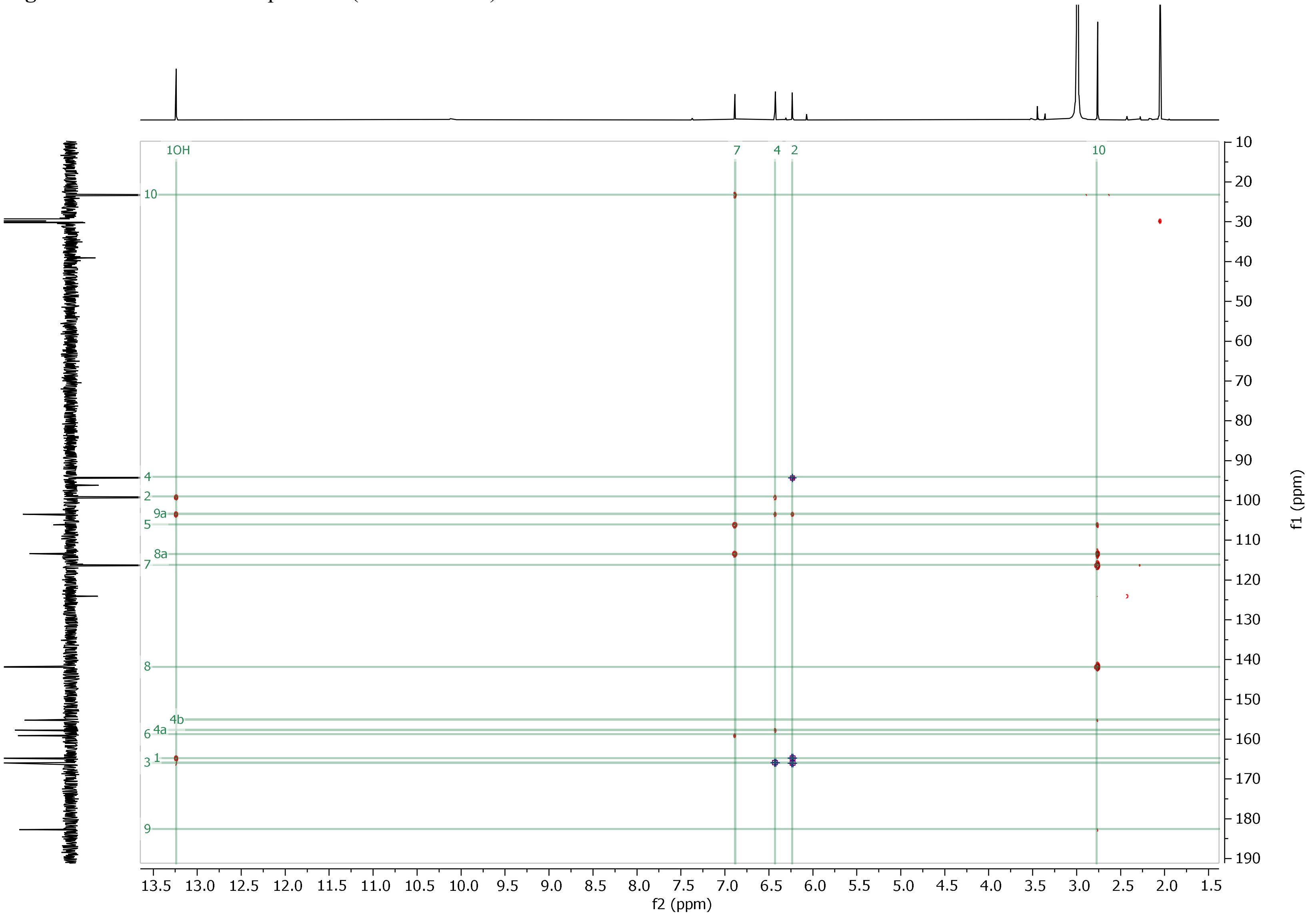

Figure S20. NOESY NMR spectrum (500 MHz) in acetone- d_6 of 5-chloronorlichexanthone **4**

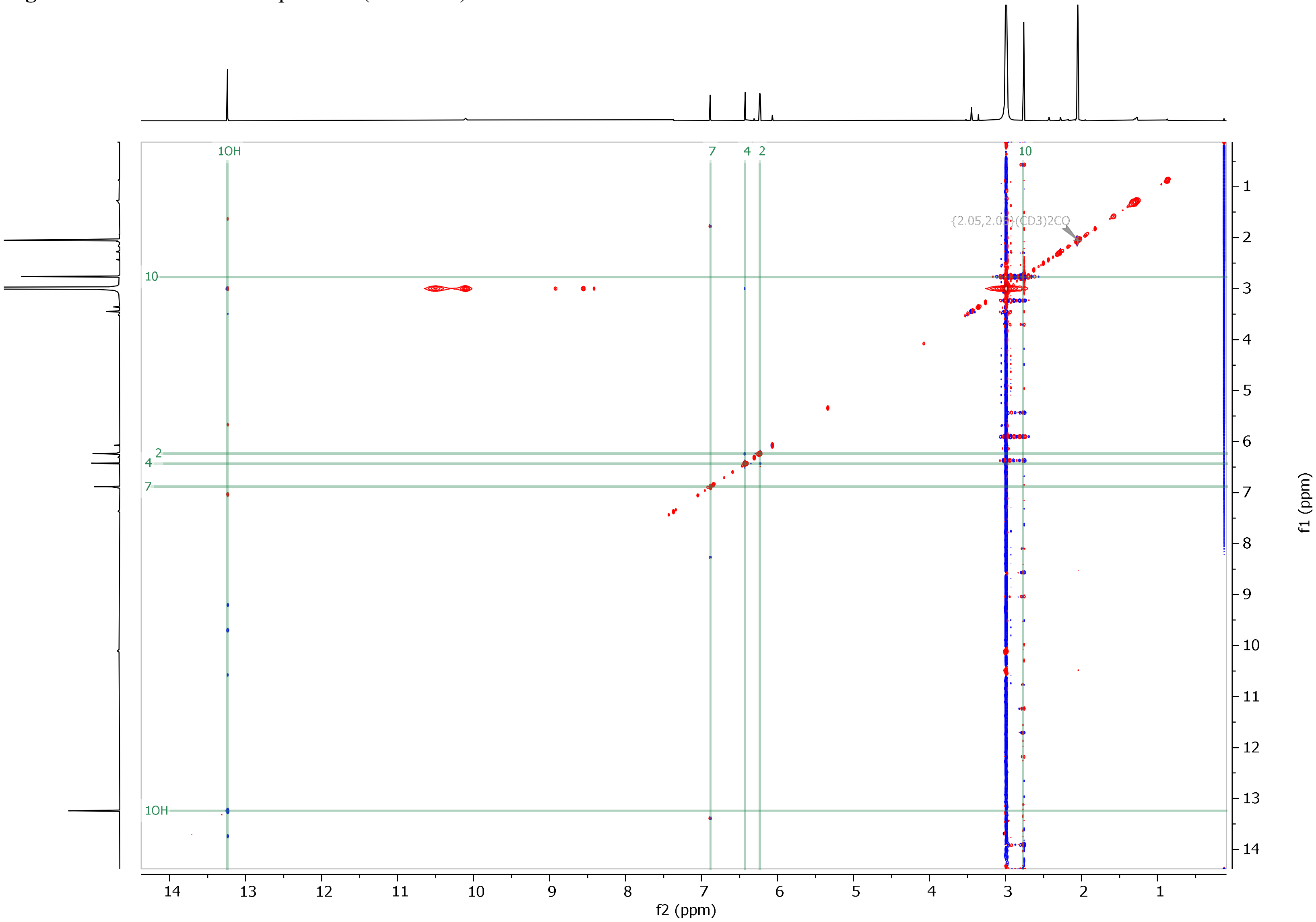

Figure S21. ^{1}H NMR spectrum (500 MHz) in acetone- d_6 of 7-chloronorlichexanthone **5**

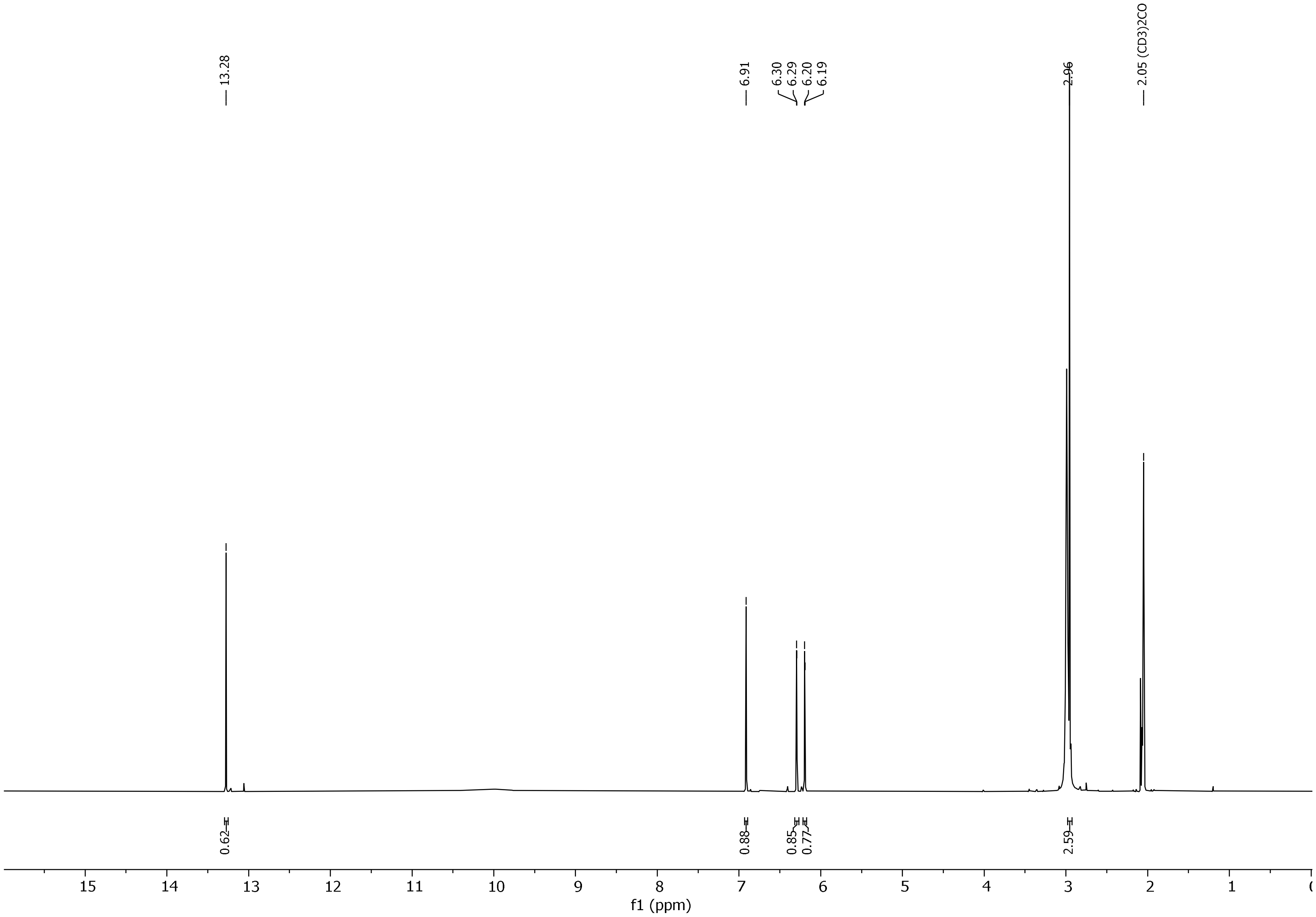

Figure S22. Jmod NMR spectrum (125 MHz) in acetone- d_6 of 7-chloronorlichexanthone **5**

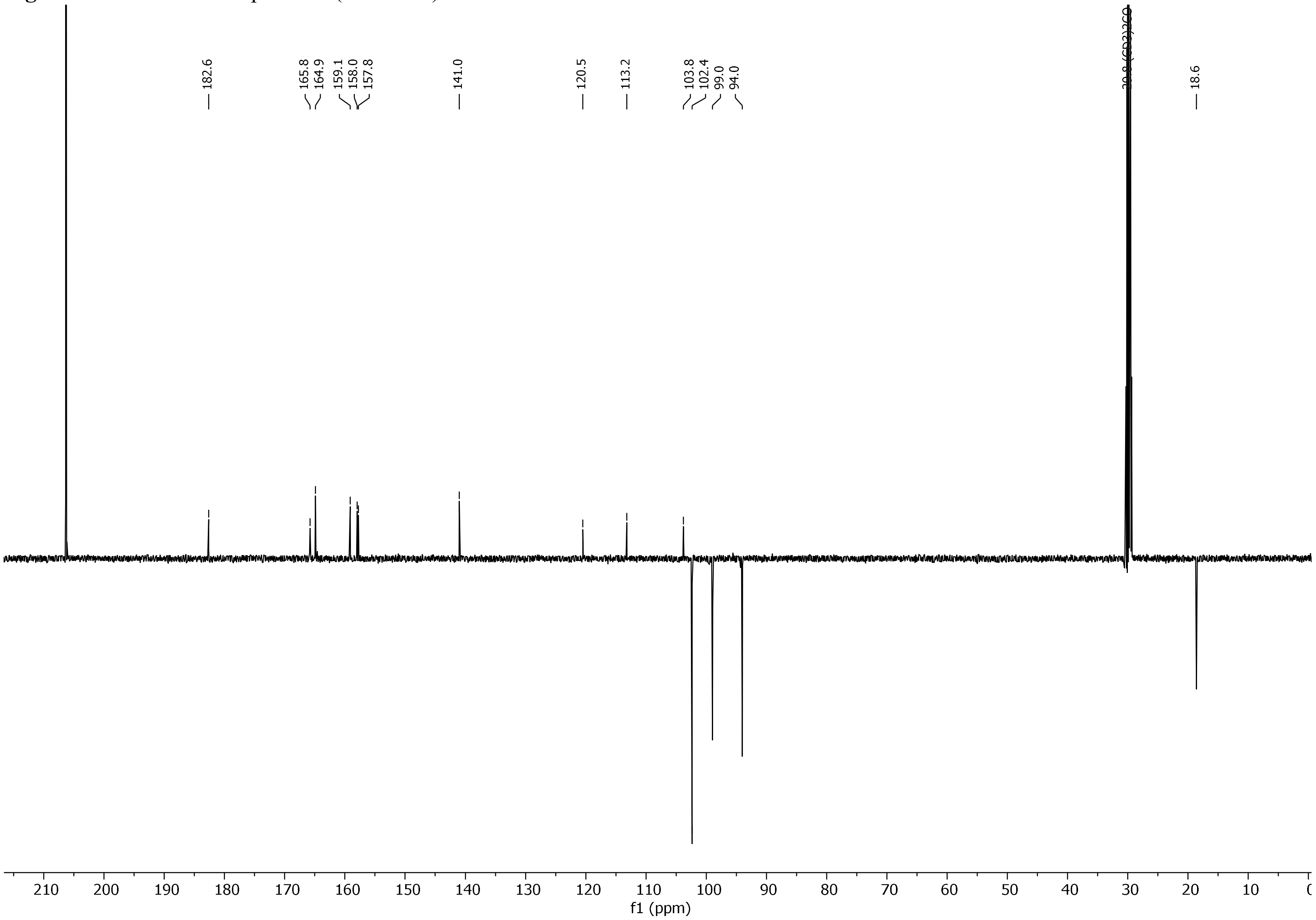

Figure S23. HSQC NMR spectrum (500/125 MHz) in acetone- d_6 of 7-chloronorlichexanthone **5**

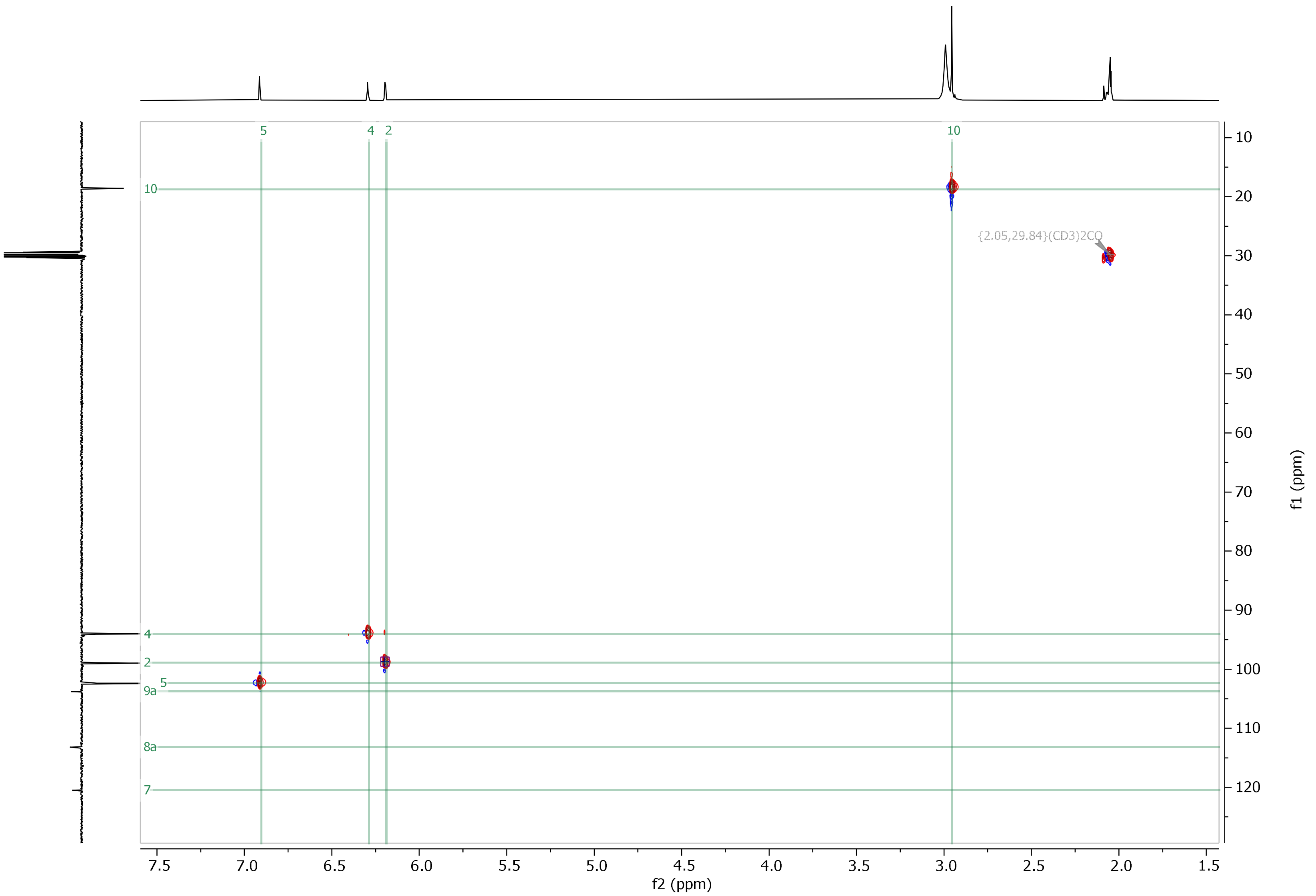

Figure S24. HMBC NMR spectrum (500/125 MHz) in acetone- d_6 of 7-chloronorlichexanthone **5**

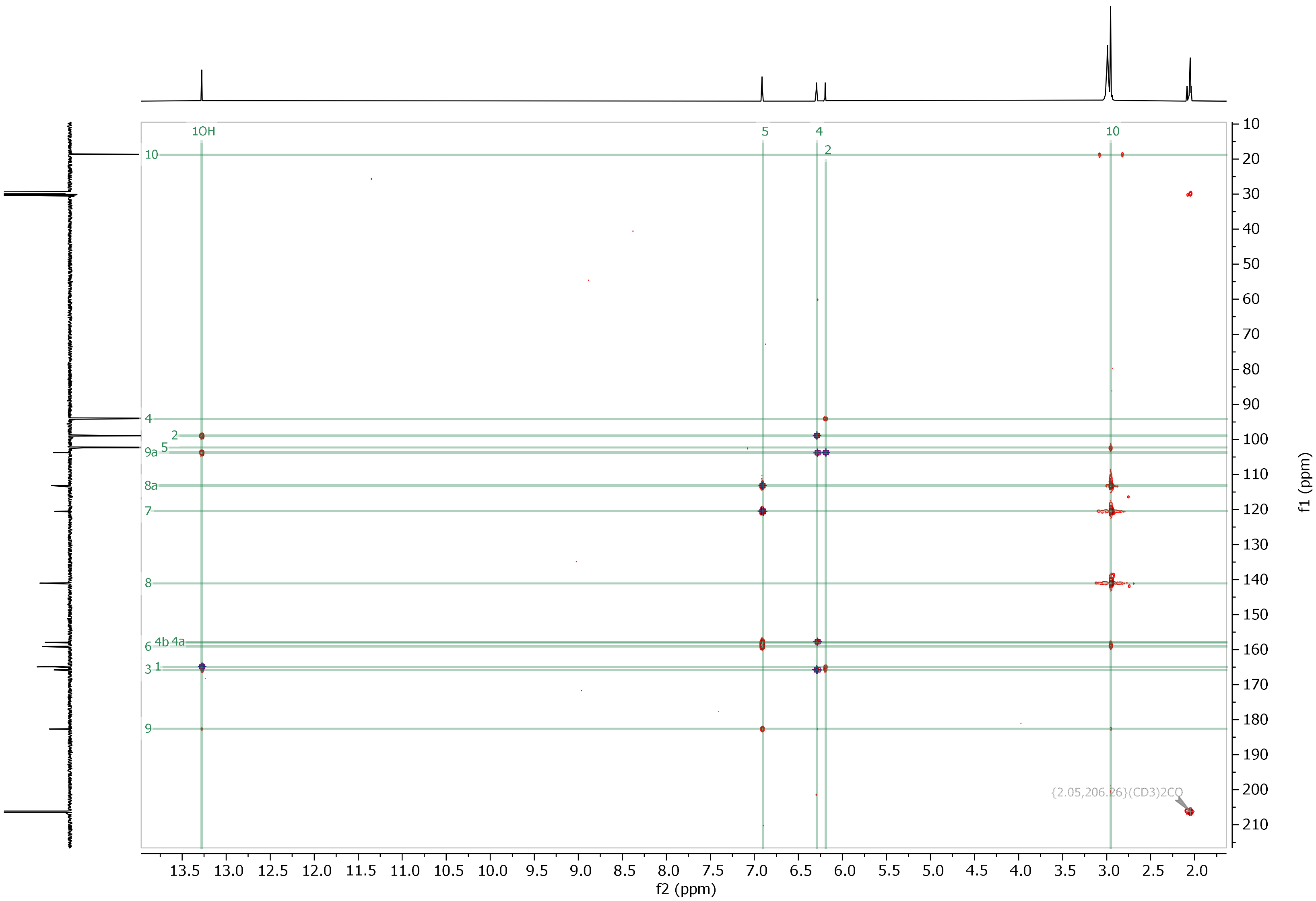

Figure S25. ^{1}H NMR spectrum (500 MHz) in acetone- d_6 of 2,4-dichloronorlichexanthone **6**

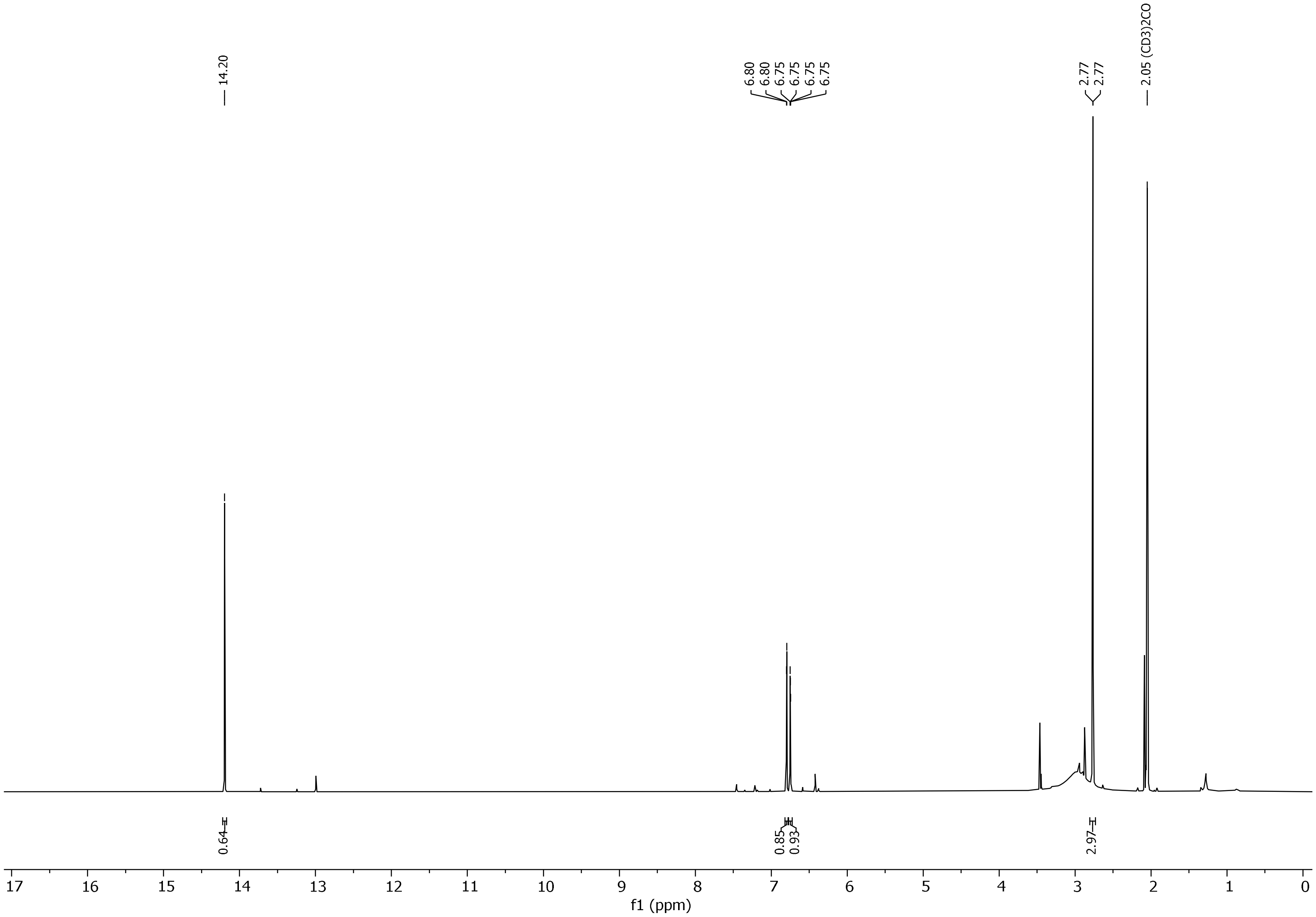

Figure S26. Jmod NMR spectrum (125 MHz) in acetone- d_6 of 2,4-dichloronorlichexanthone **6**

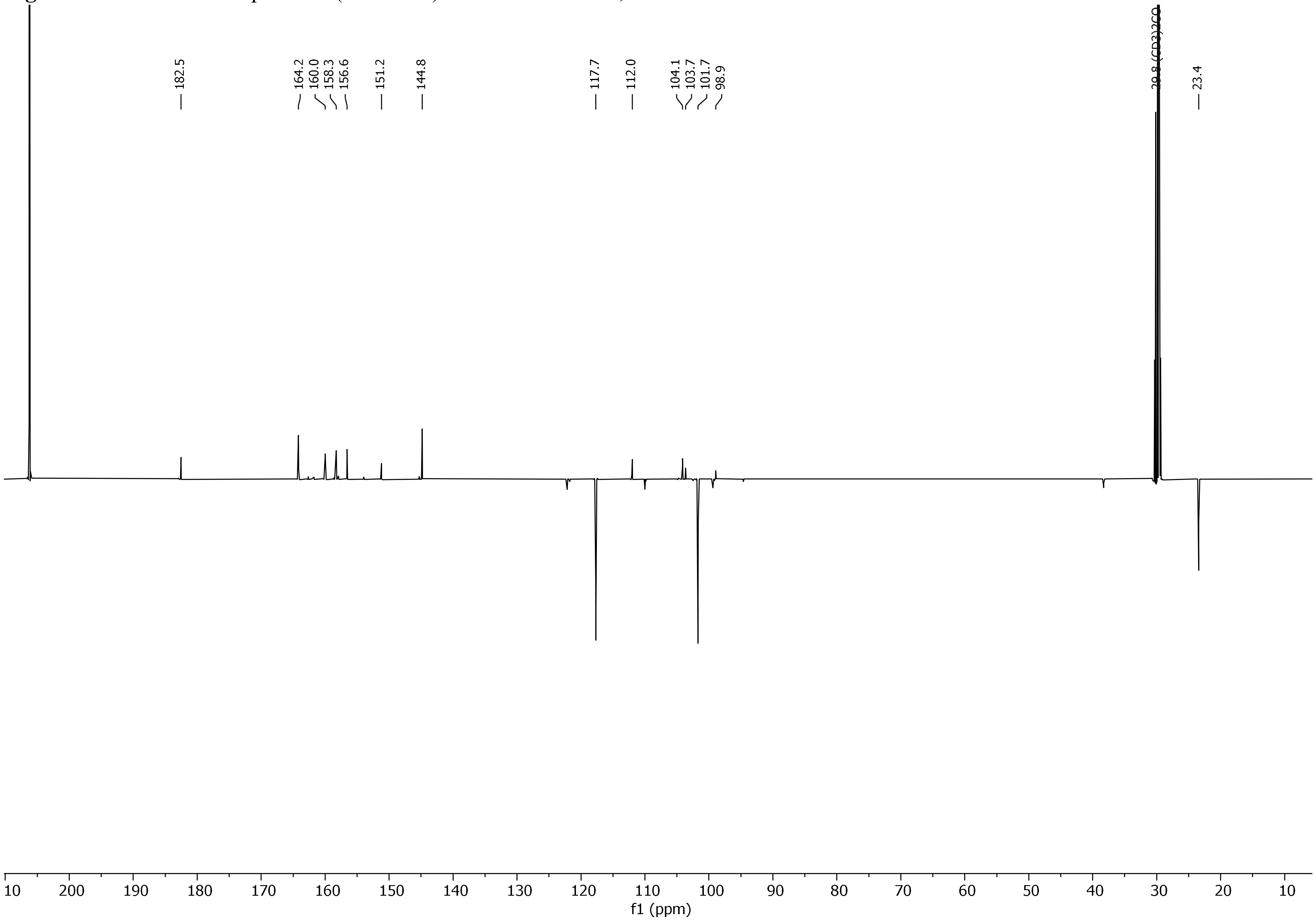

Figure S27. HSQC NMR spectrum (500/125 MHz) in acetone- d_6 of 2,4-dichloronorlichexanthone **6**

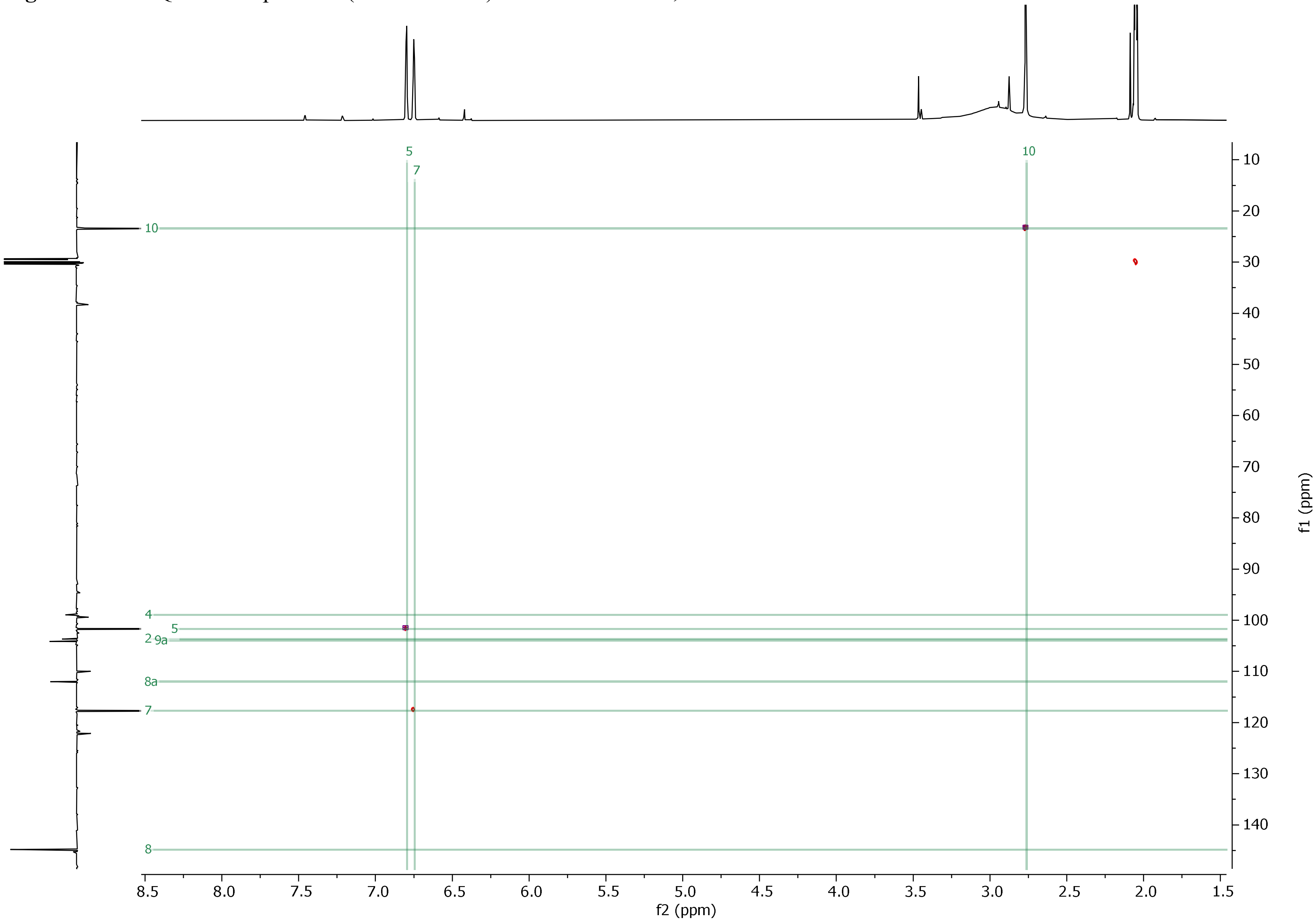

Figure S28. HMBC NMR spectrum (500/125 MHz) in acetone- d_6 of 2,4-dichloronorlichexanthone **6**

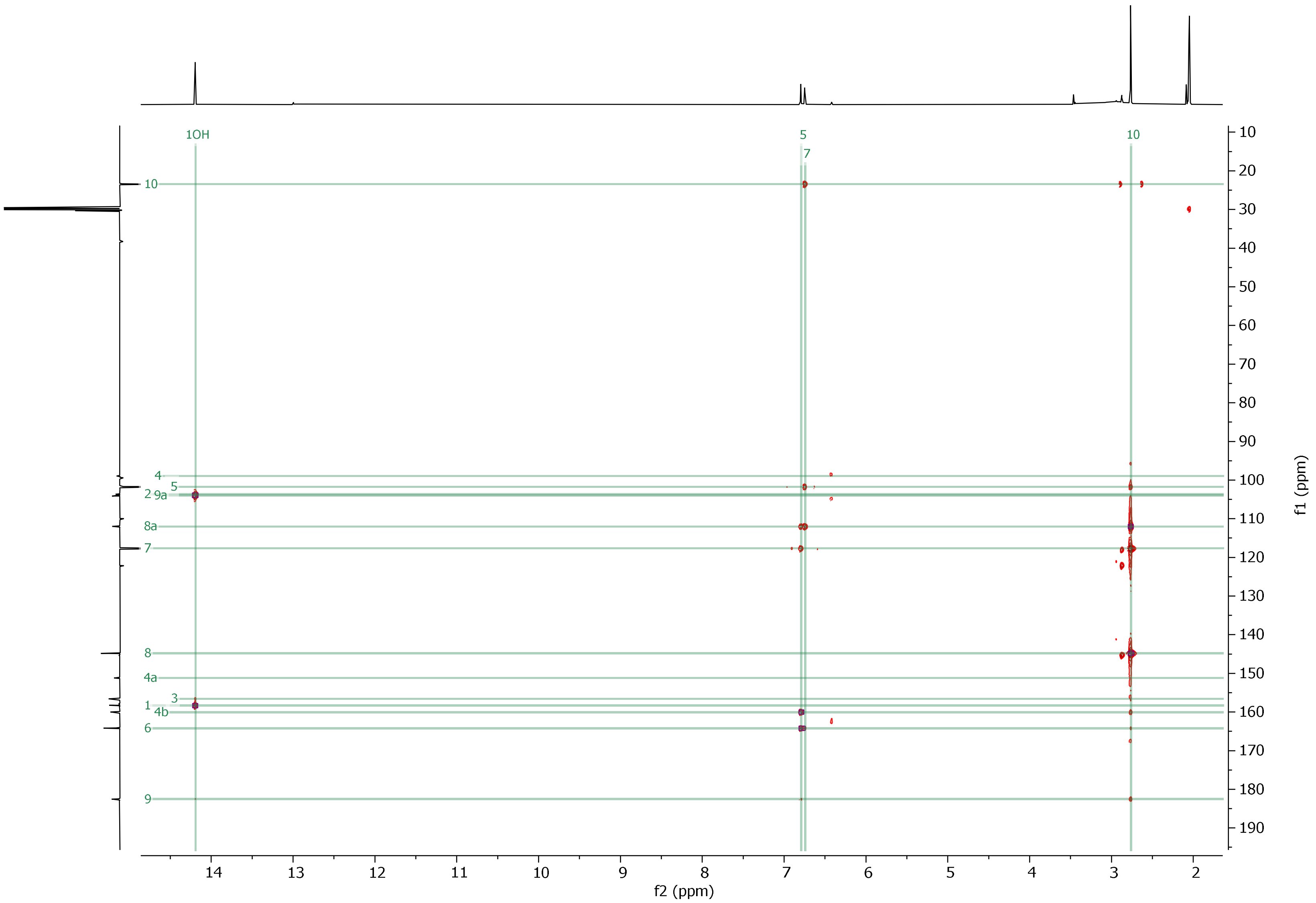

Figure S29. NOESY NMR spectrum (500 MHz) in acetone- d_6 of 2,4-dichloronorlichexanthone **6**

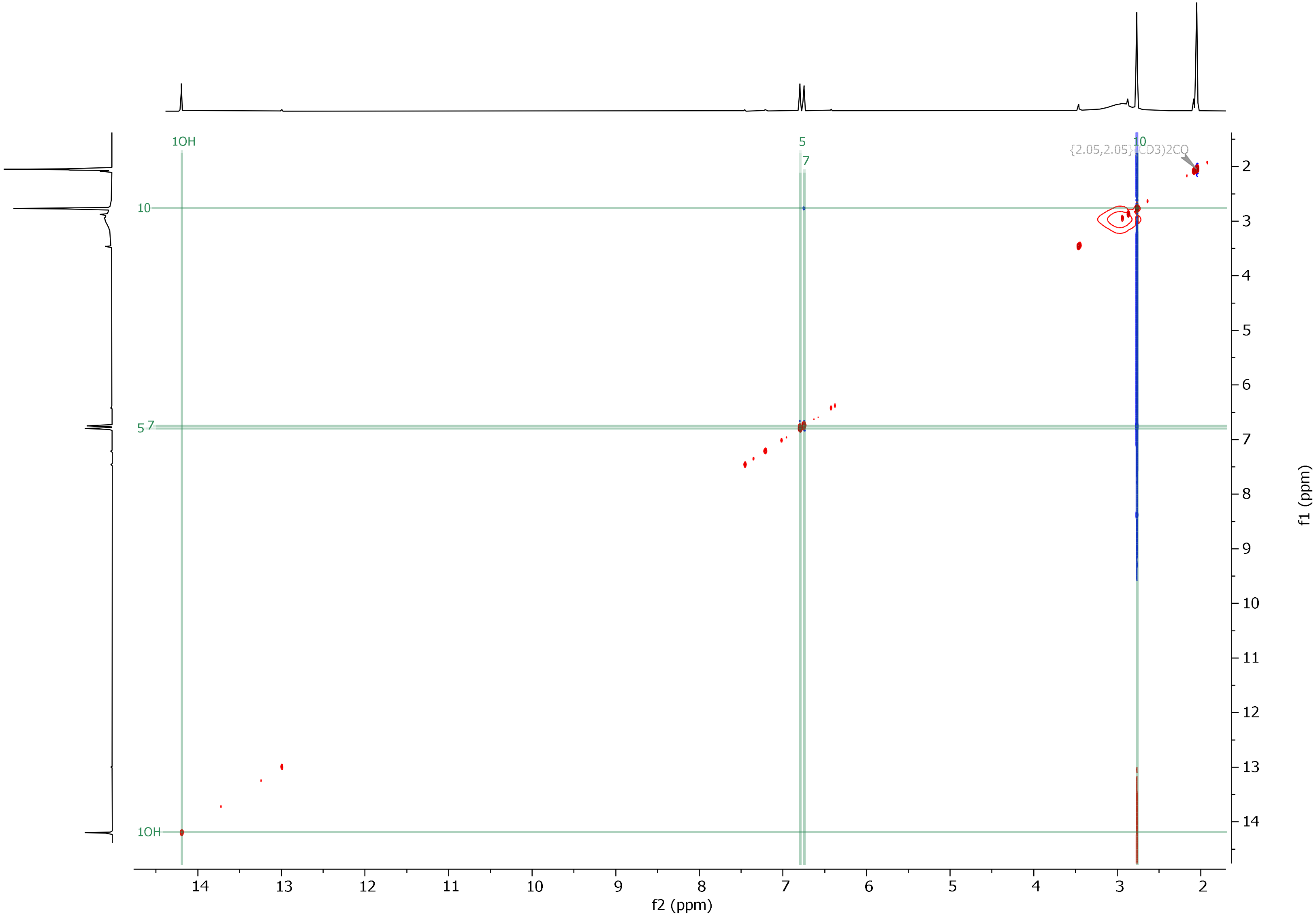

Figure S30. ^{1}H NMR spectrum (500 MHz) in acetone- d_6 of 2,5-dichloronorlichexanthone **7**

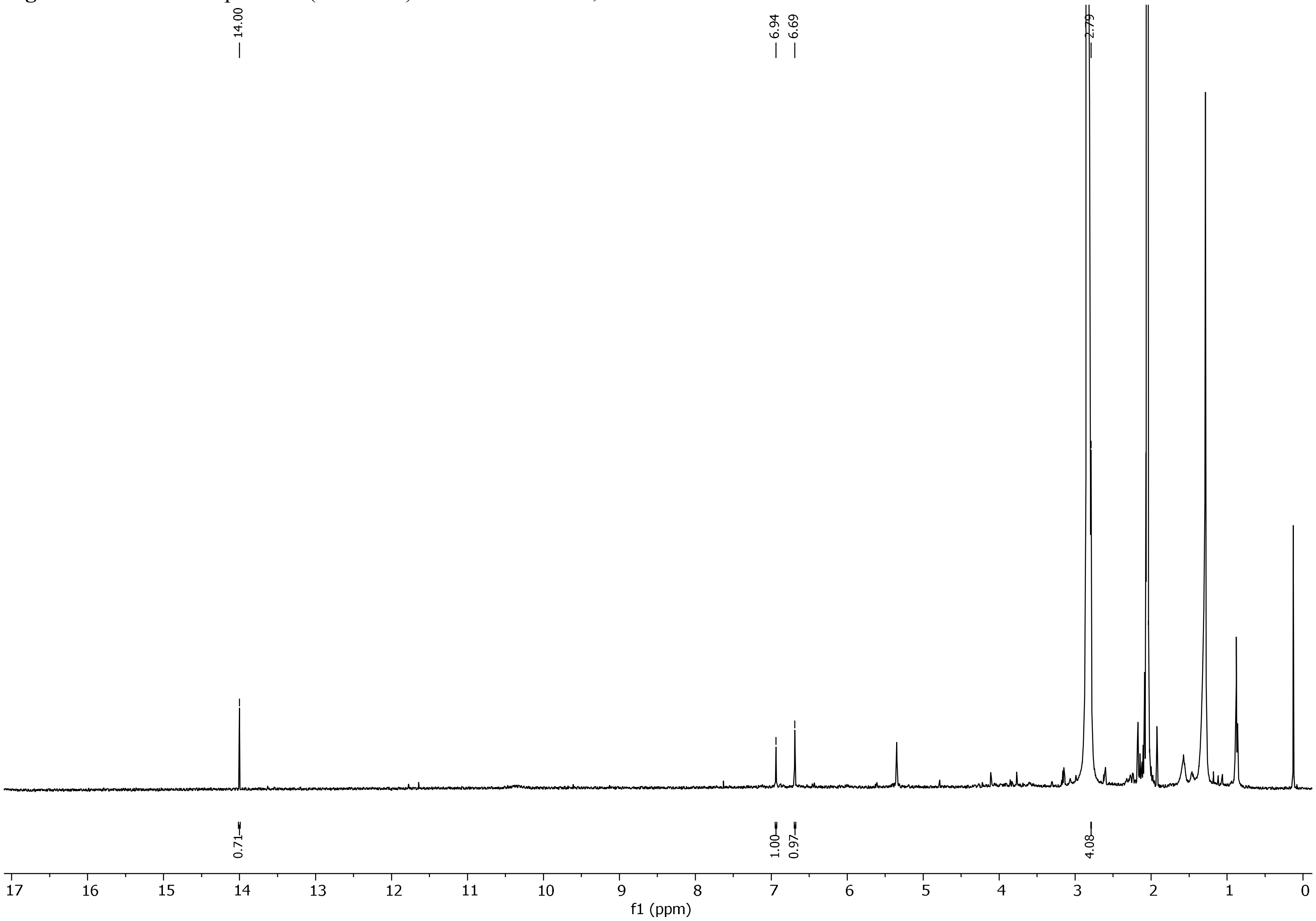

Figure S31. HSQC NMR spectrum (500/125 MHz) in acetone- d_6 of 2,5-dichloronorlichexanthone **7**

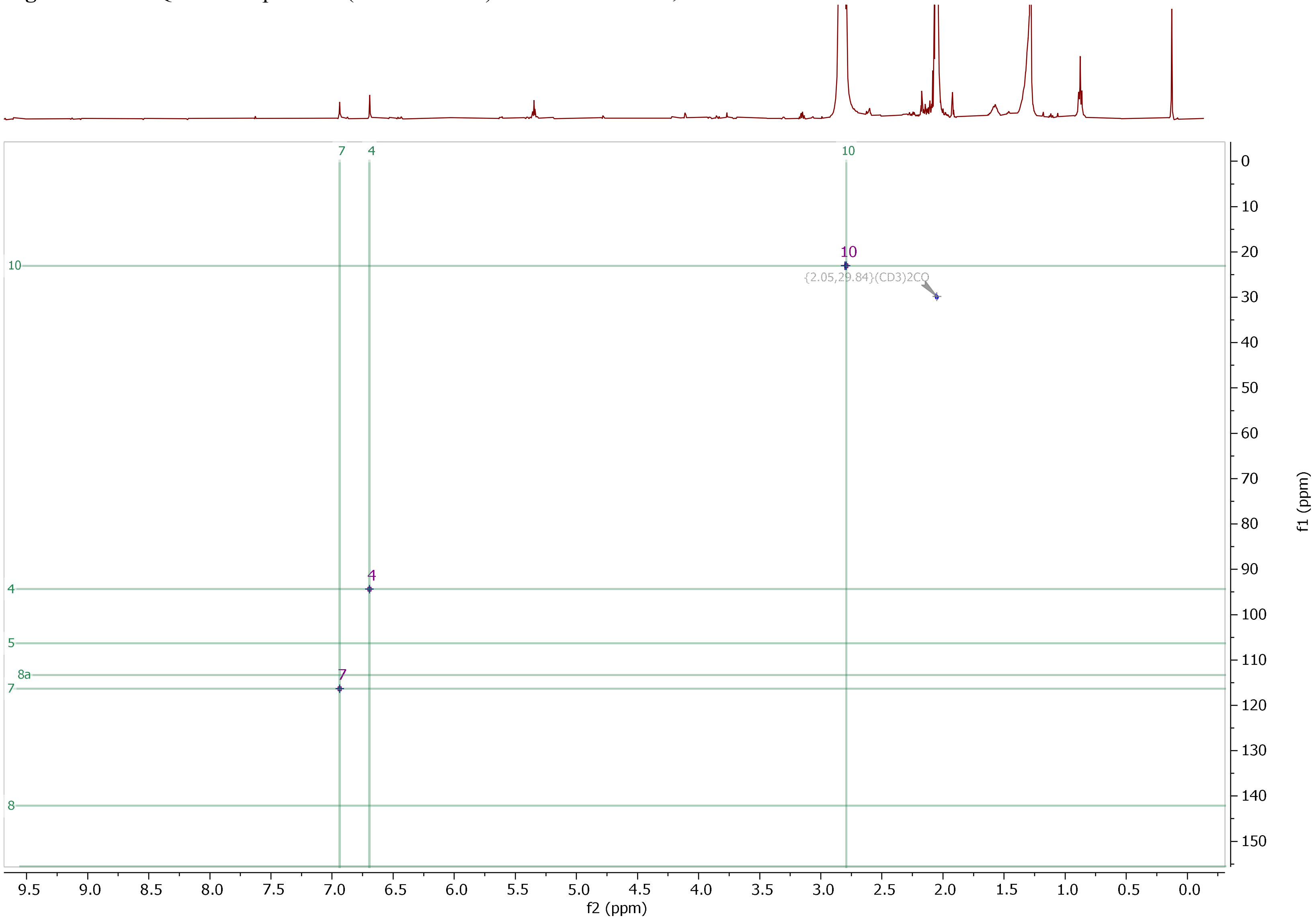

Figure S32. HMBC NMR spectrum (500/125 MHz) in acetone- d_6 of 2,5-dichloronorlichexanthone **7**

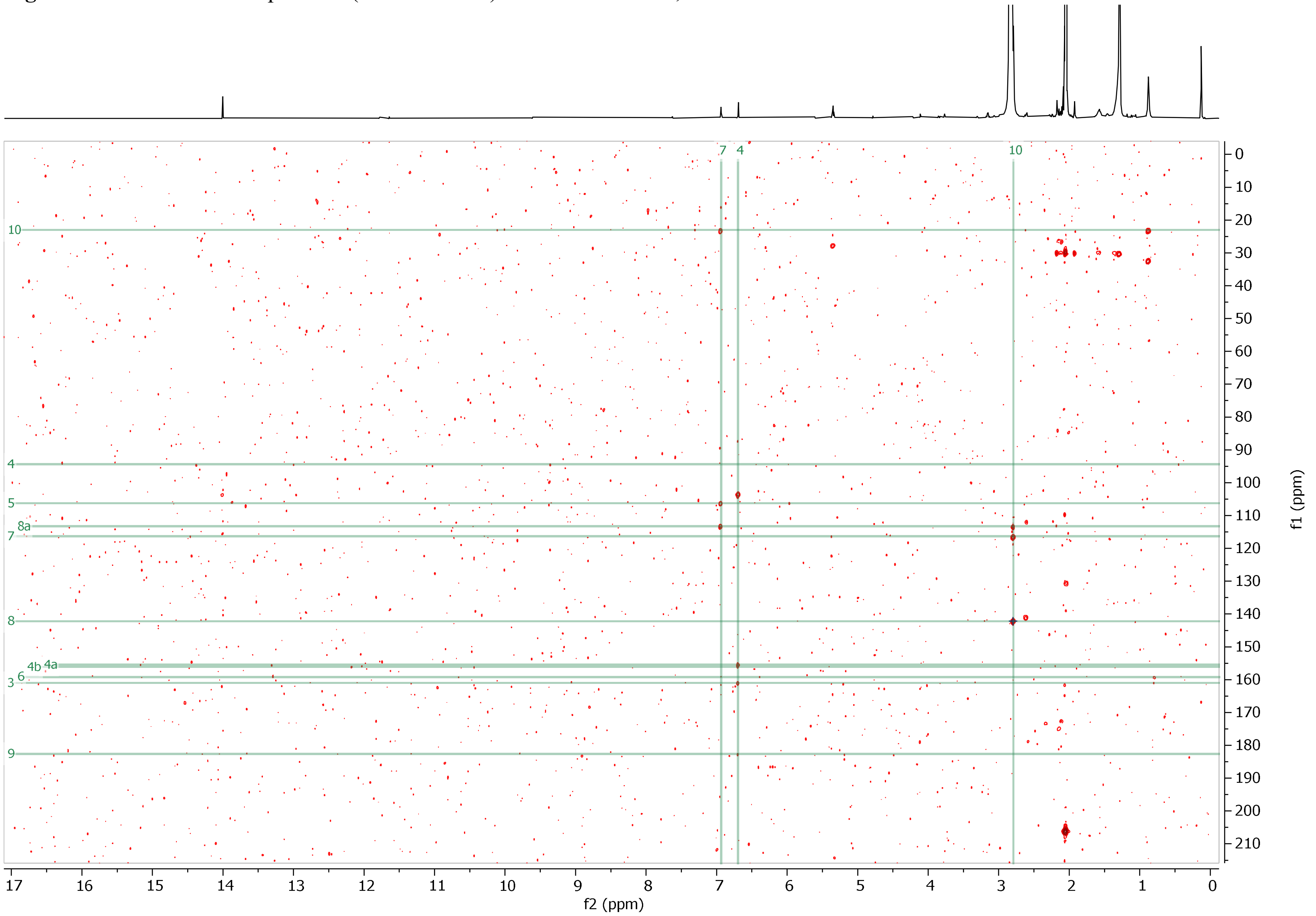

Figure S33. NOESY NMR spectrum (500 MHz) in acetone- d_6 of 2,5-dichloronorlichexanthone **7**

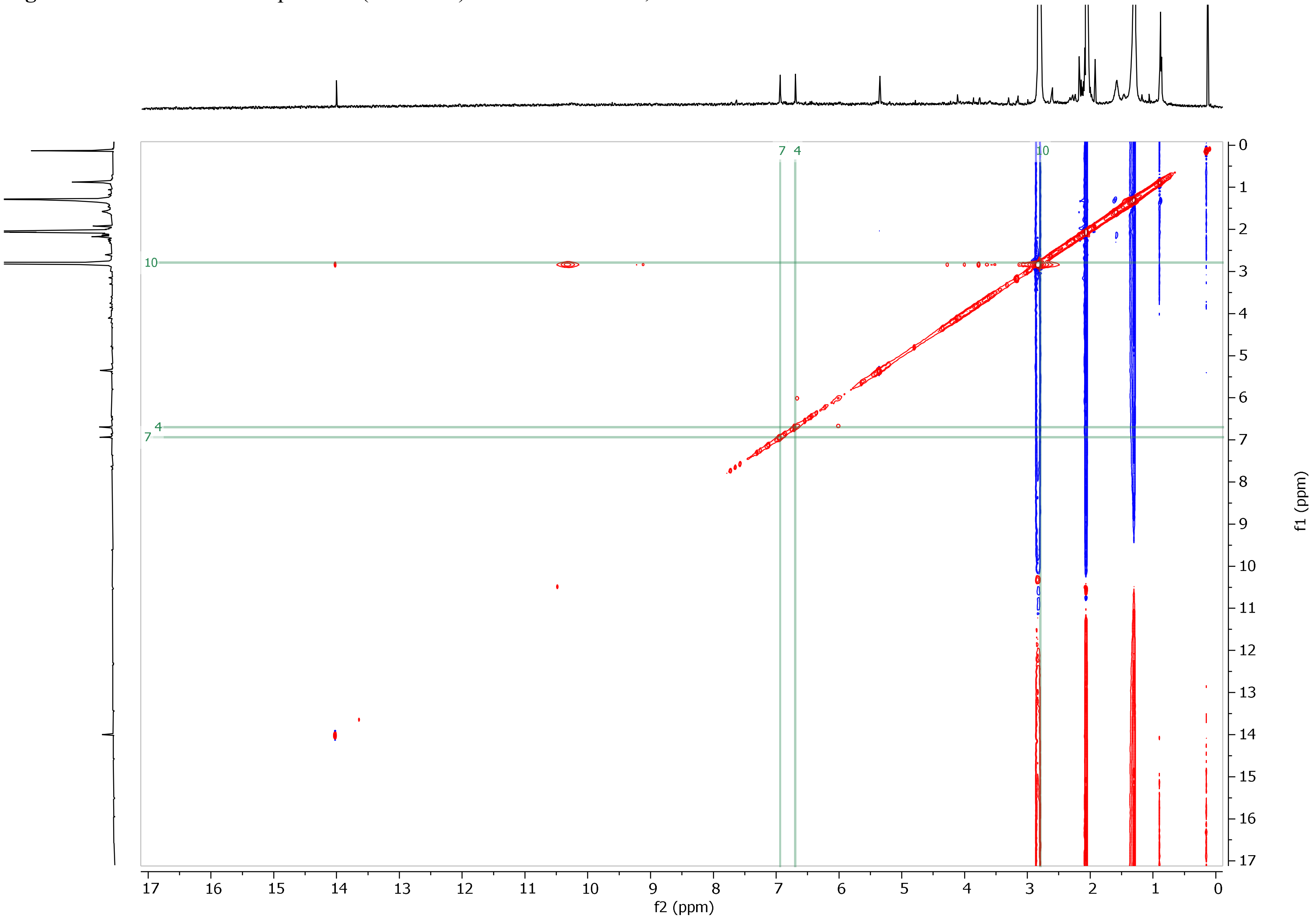

Figure S34. ^{1}H NMR spectrum (500 MHz) in acetone- d_6 of 2,7-dichloronorlichexanthone **8**

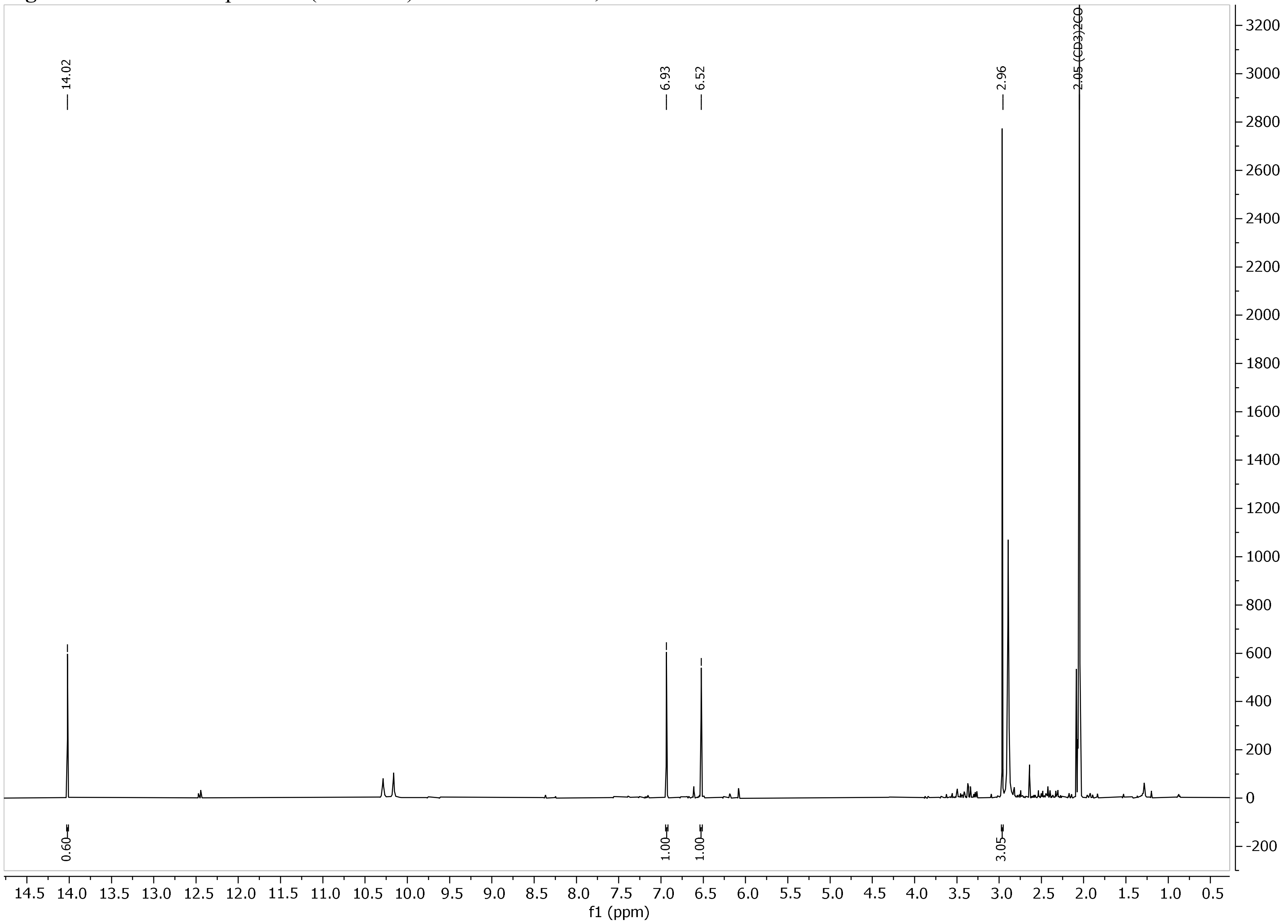

Figure S35. Jmod NMR spectrum (125 MHz) in acetone- d_6 of 2,7-dichloronorlichexanthone **8**

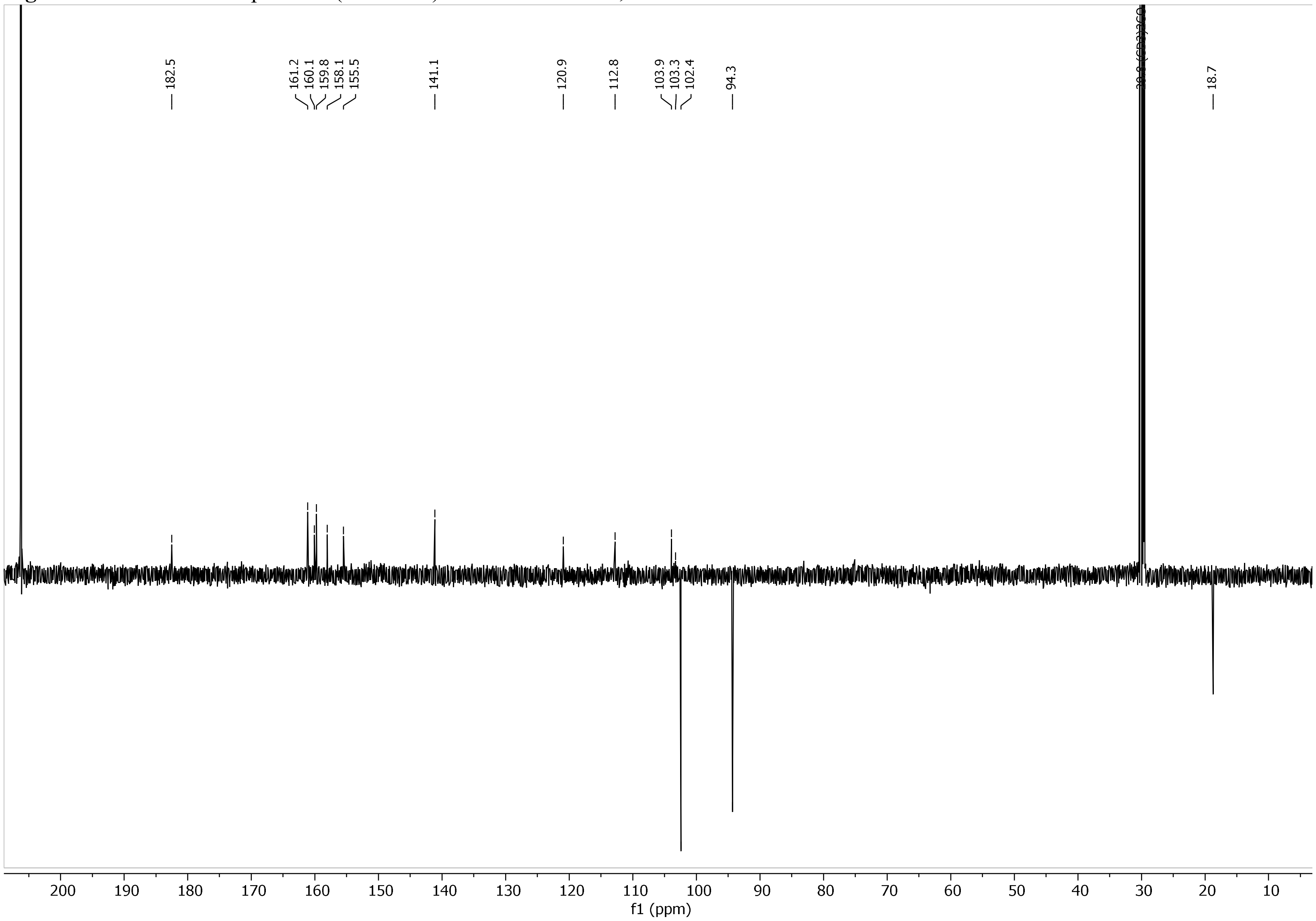

Figure S36. HSQC NMR spectrum (500/125 MHz) in acetone- d_6 of 2,7-dichloronorlichexanthone **8**

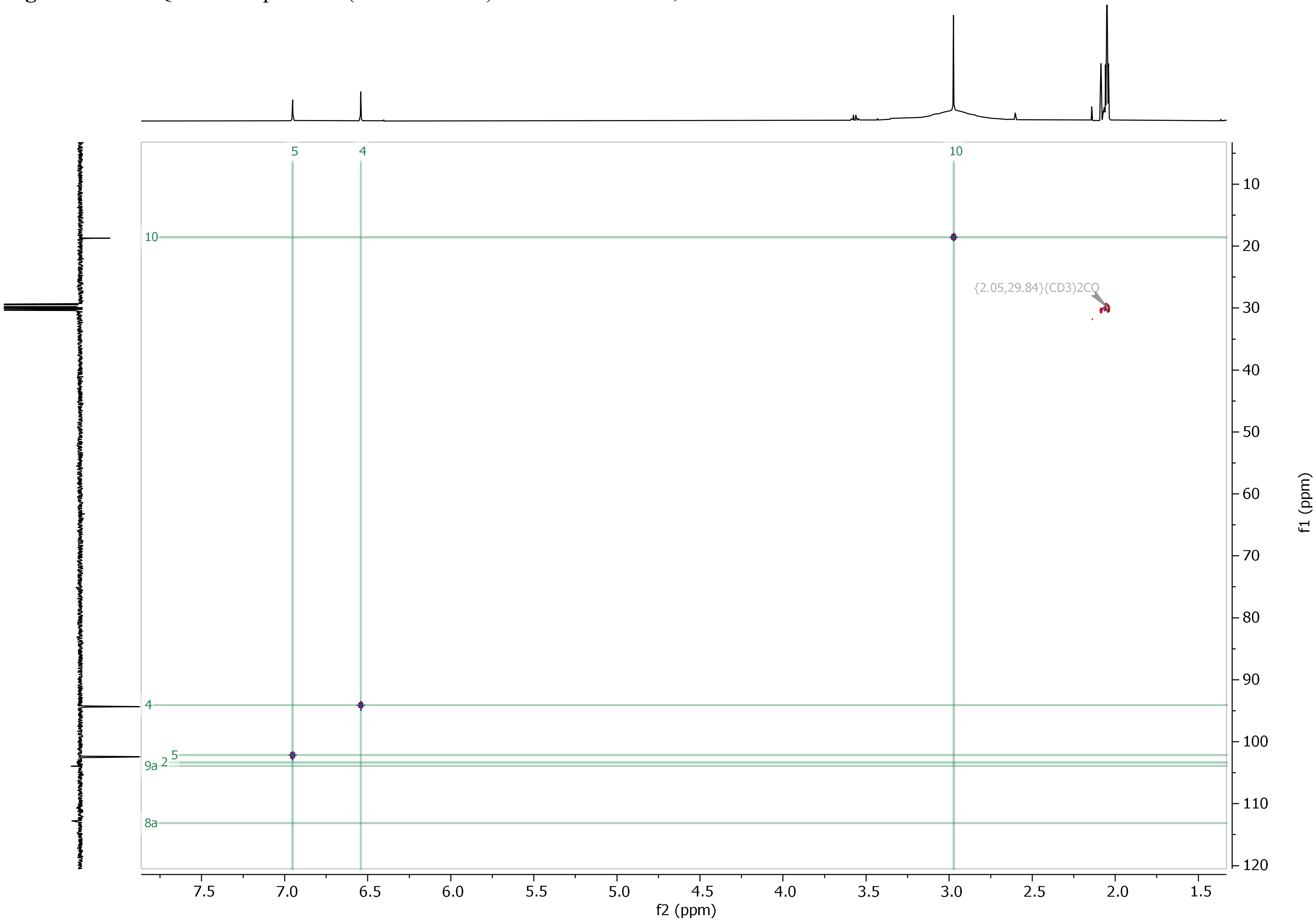

Figure S37. HMBC NMR spectrum (500/125 MHz) in acetone- d_6 of 2,7-dichloronorlichexanthone **8**

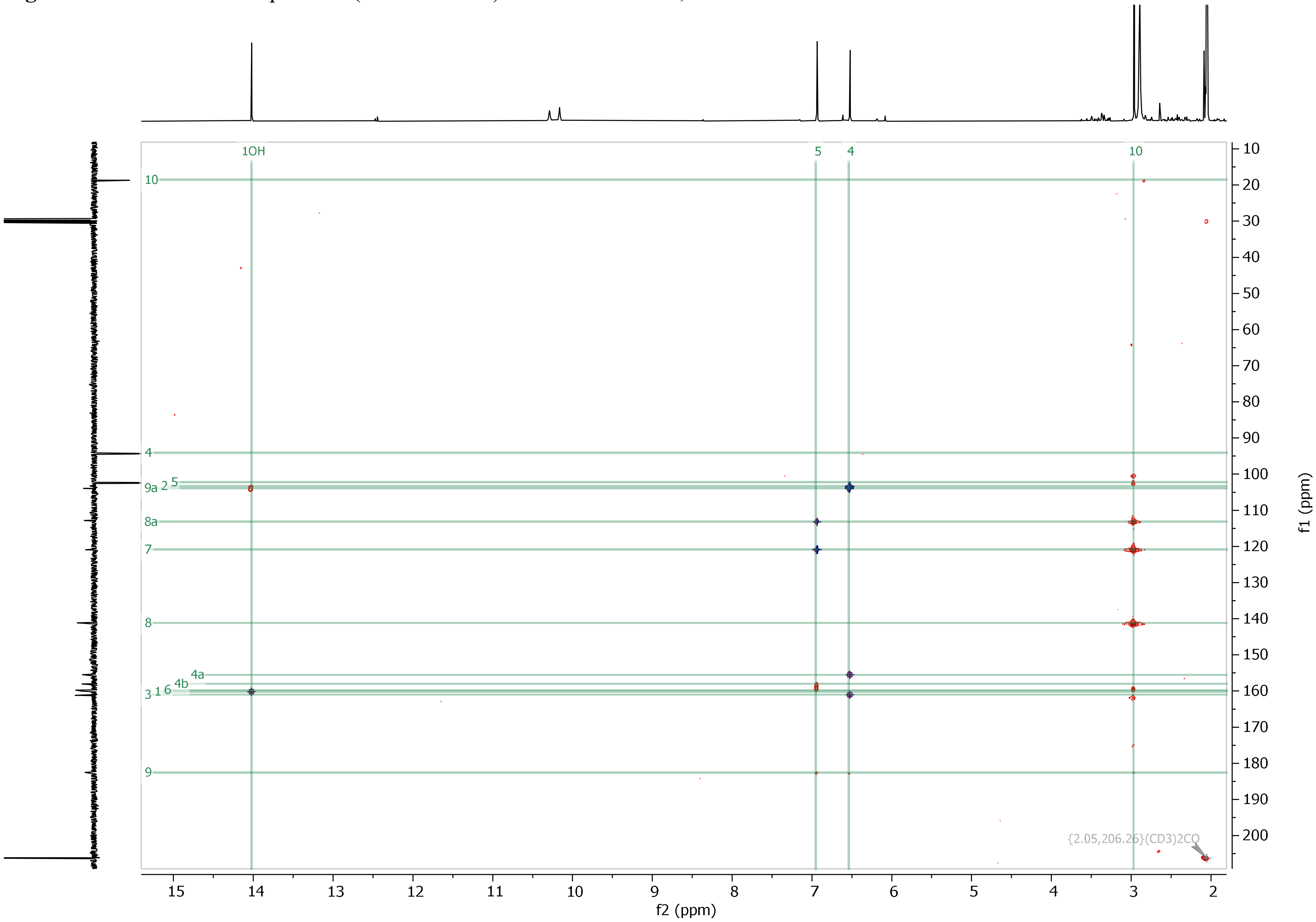

Figure S38. NOESY NMR spectrum (500 MHz) in acetone- d_6 of 2,7-dichloronorlichexanthone **8**

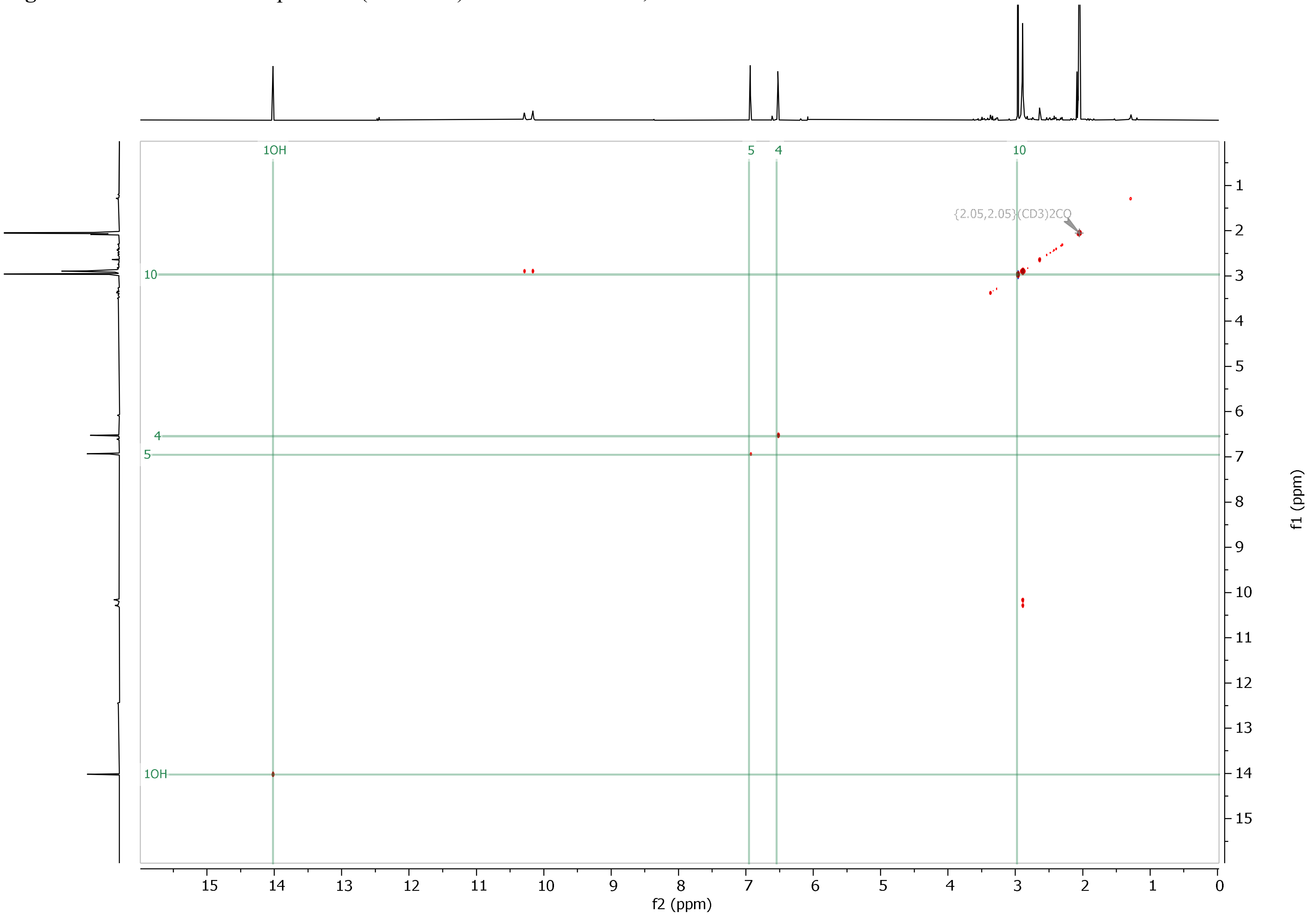

Figure S39. ^{1}H NMR spectrum (500 MHz) in acetone- d_6 of 4,5-dichloronorlichexanthone **9**

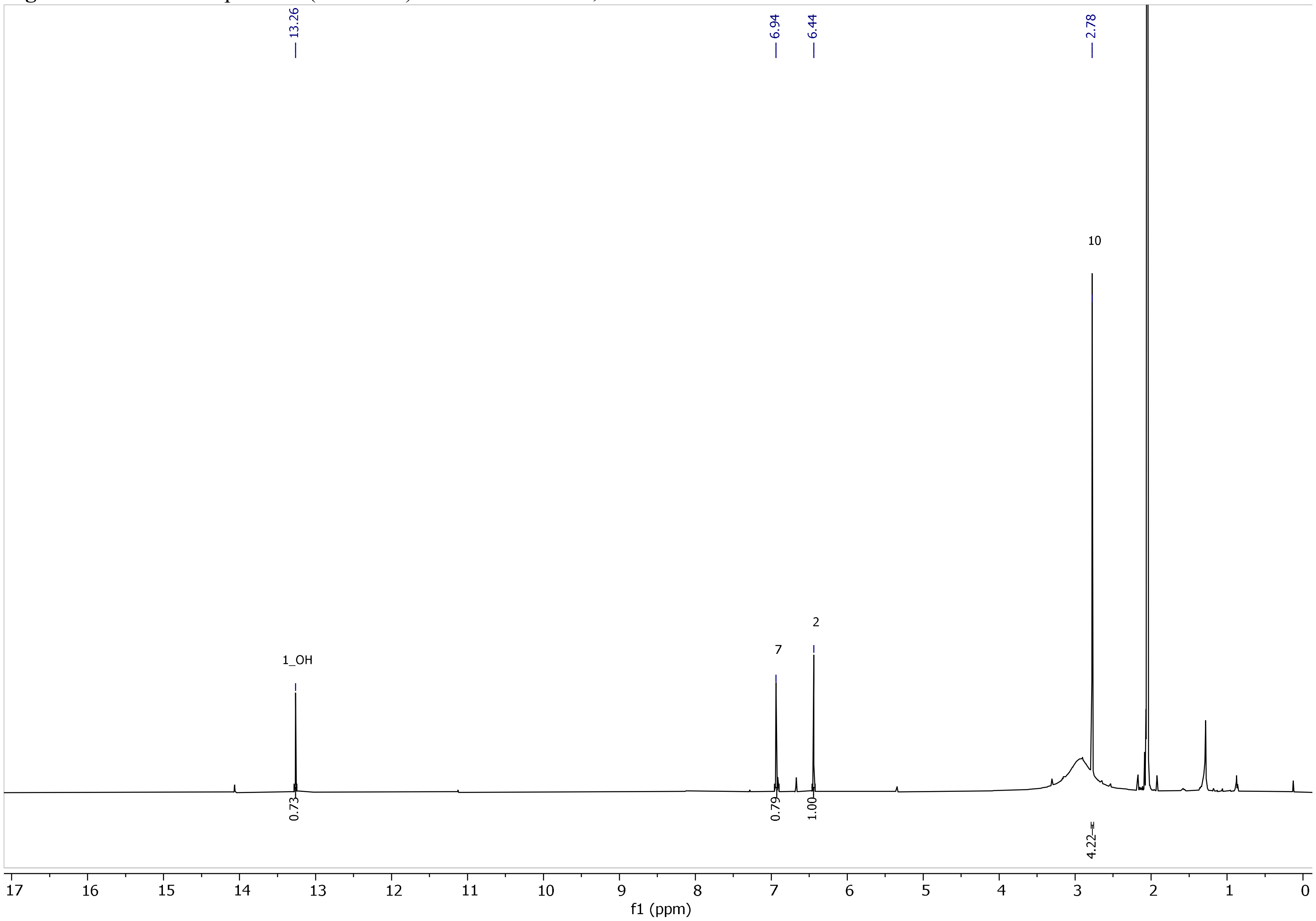

Figure S40. Jmod NMR spectrum (125 MHz) in acetone- d_6 of 4,5-dichloronorlichexanthone **9**

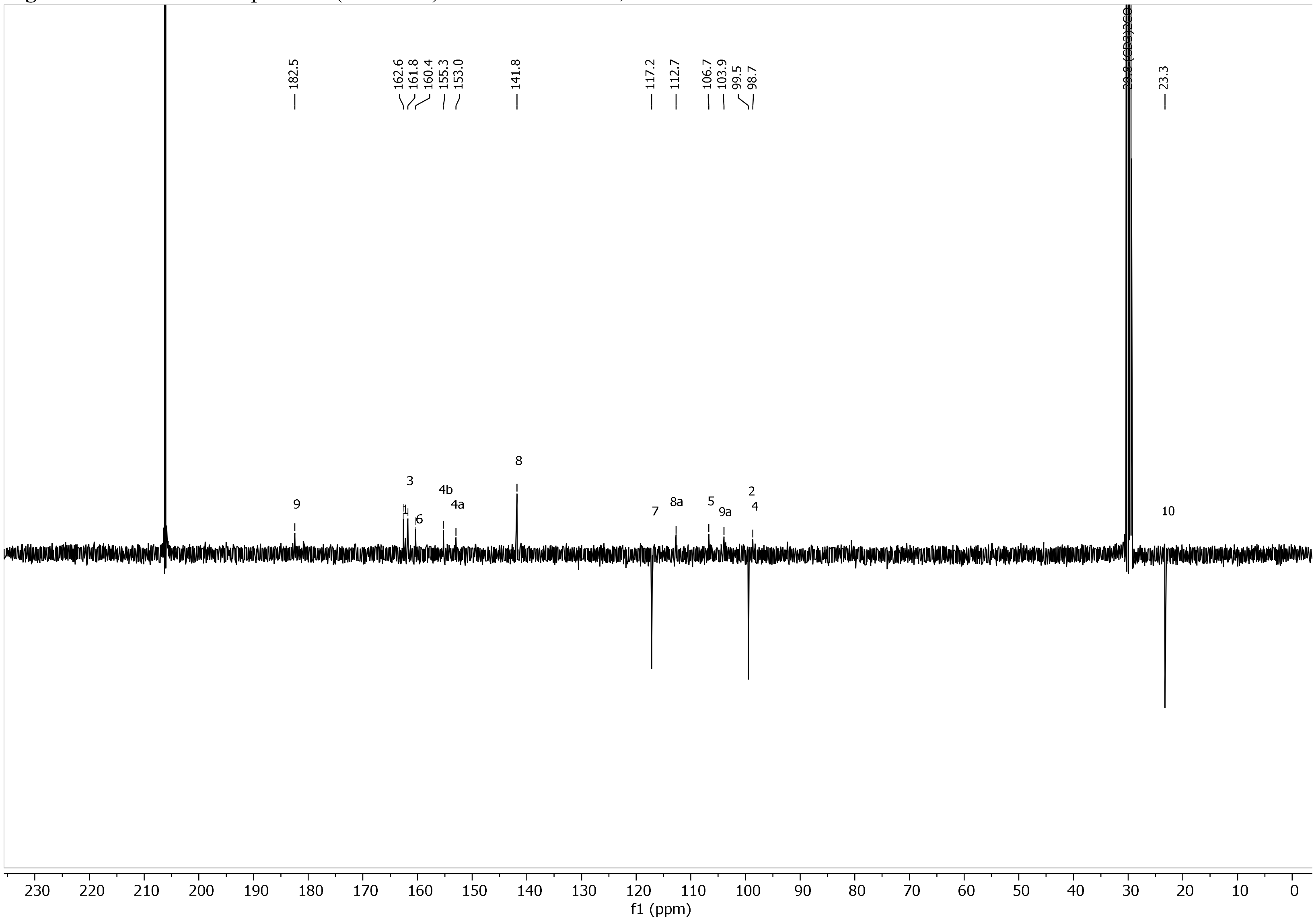

Figure S41. HSQC NMR spectrum (500/125 MHz) in acetone- d_6 of 4,5-dichloronorlichexanthone **9**

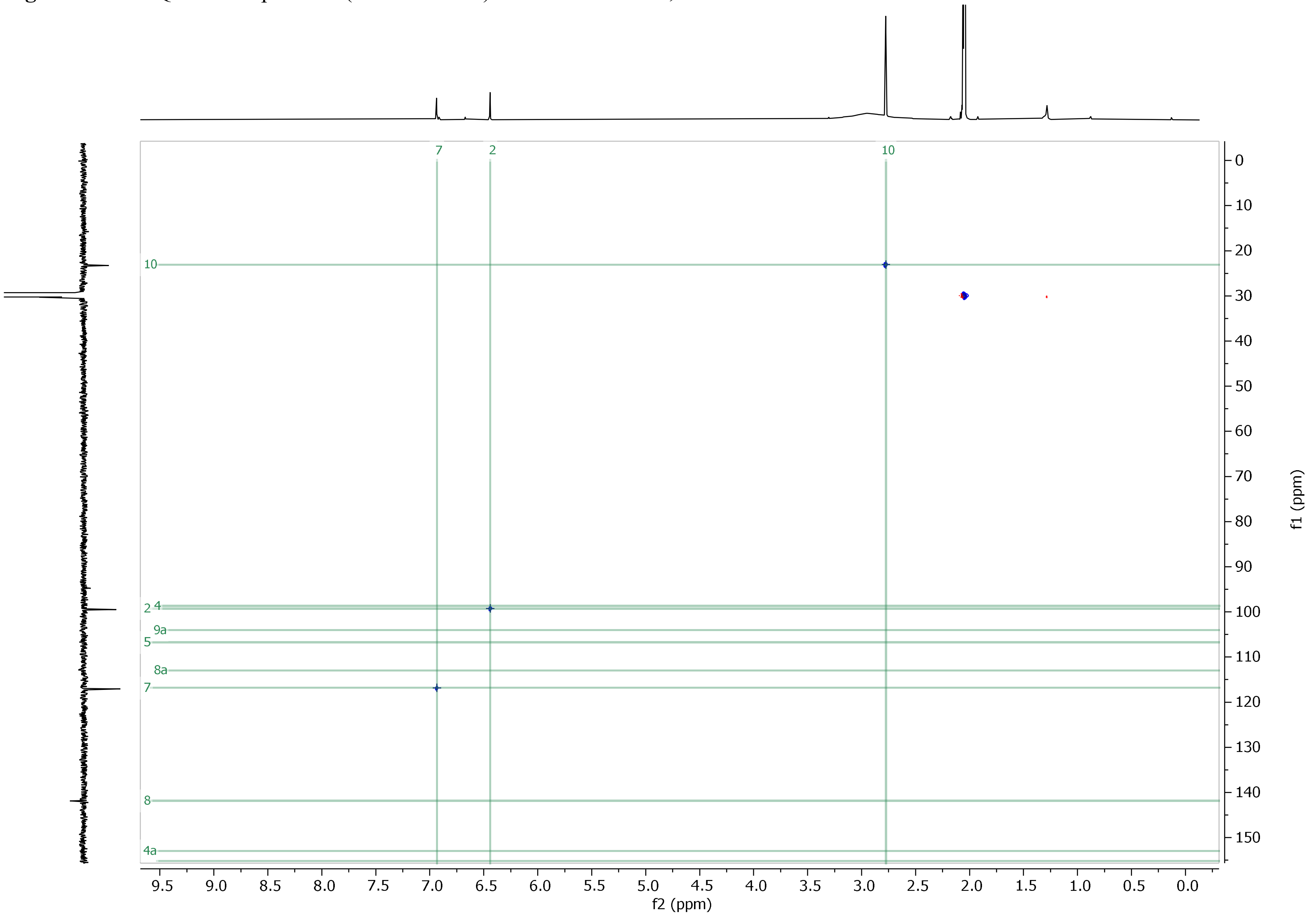

Figure S42. HMBC NMR spectrum (500/125 MHz) in acetone- d_6 of 4,5-dichloronorlichexanthone **9**

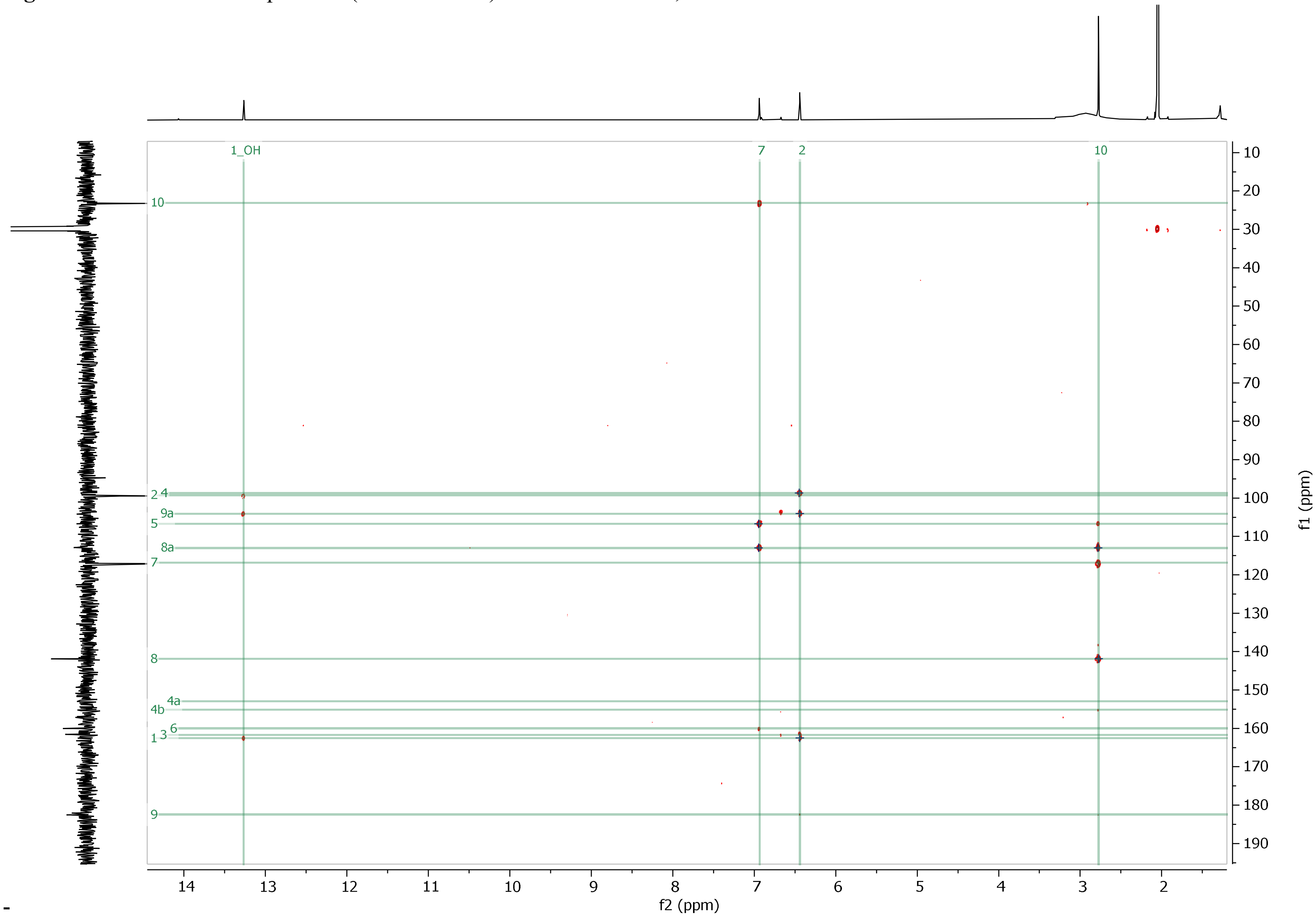

Figure S43. NOESY NMR spectrum (500 MHz) in acetone- d_6 of 4,5-dichloronorlichexanthone **9**

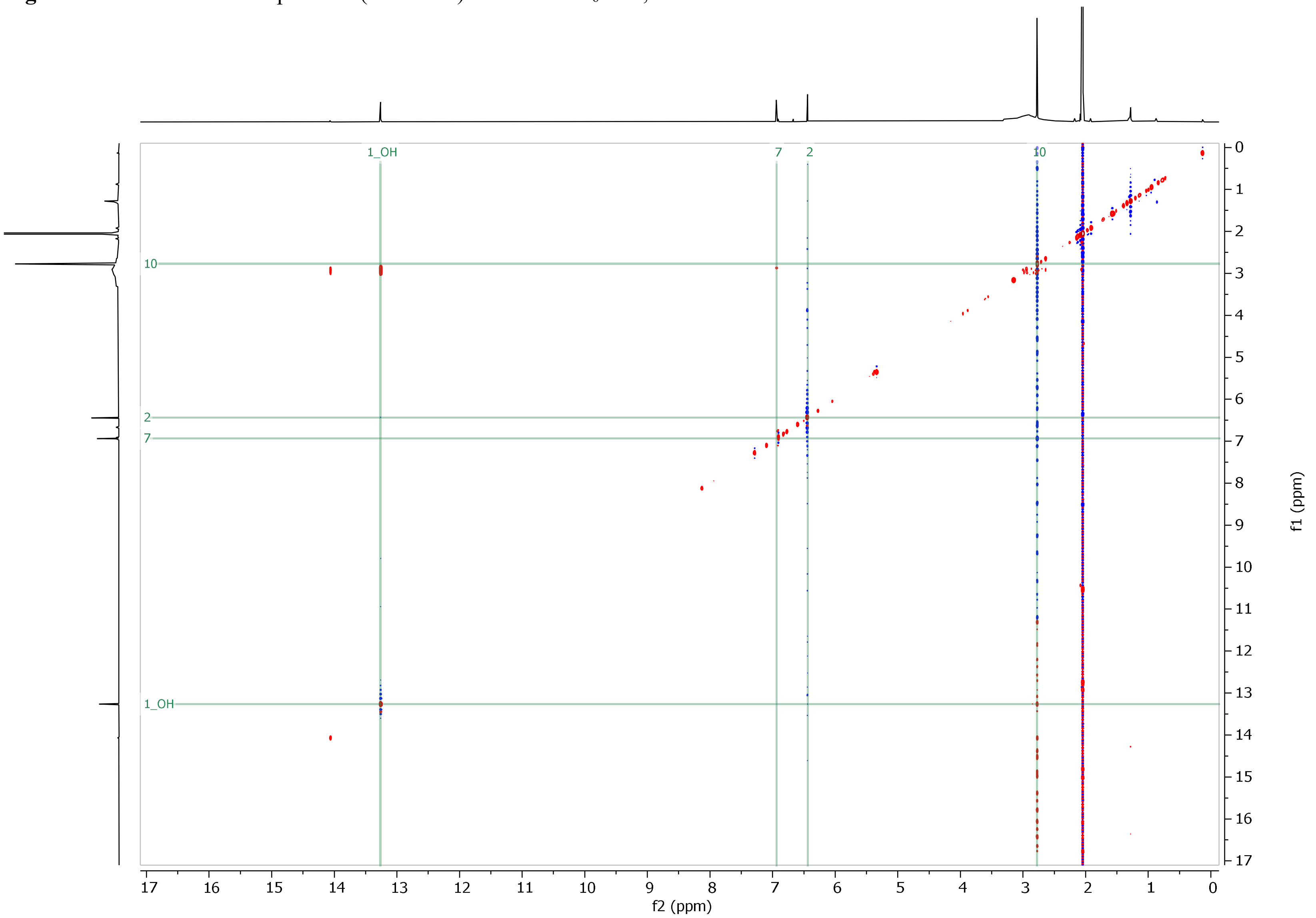

Figure S44. ^{1}H NMR spectrum (500 MHz) in acetone- d_6 of 4,7-dichloronorlichexanthone **10**

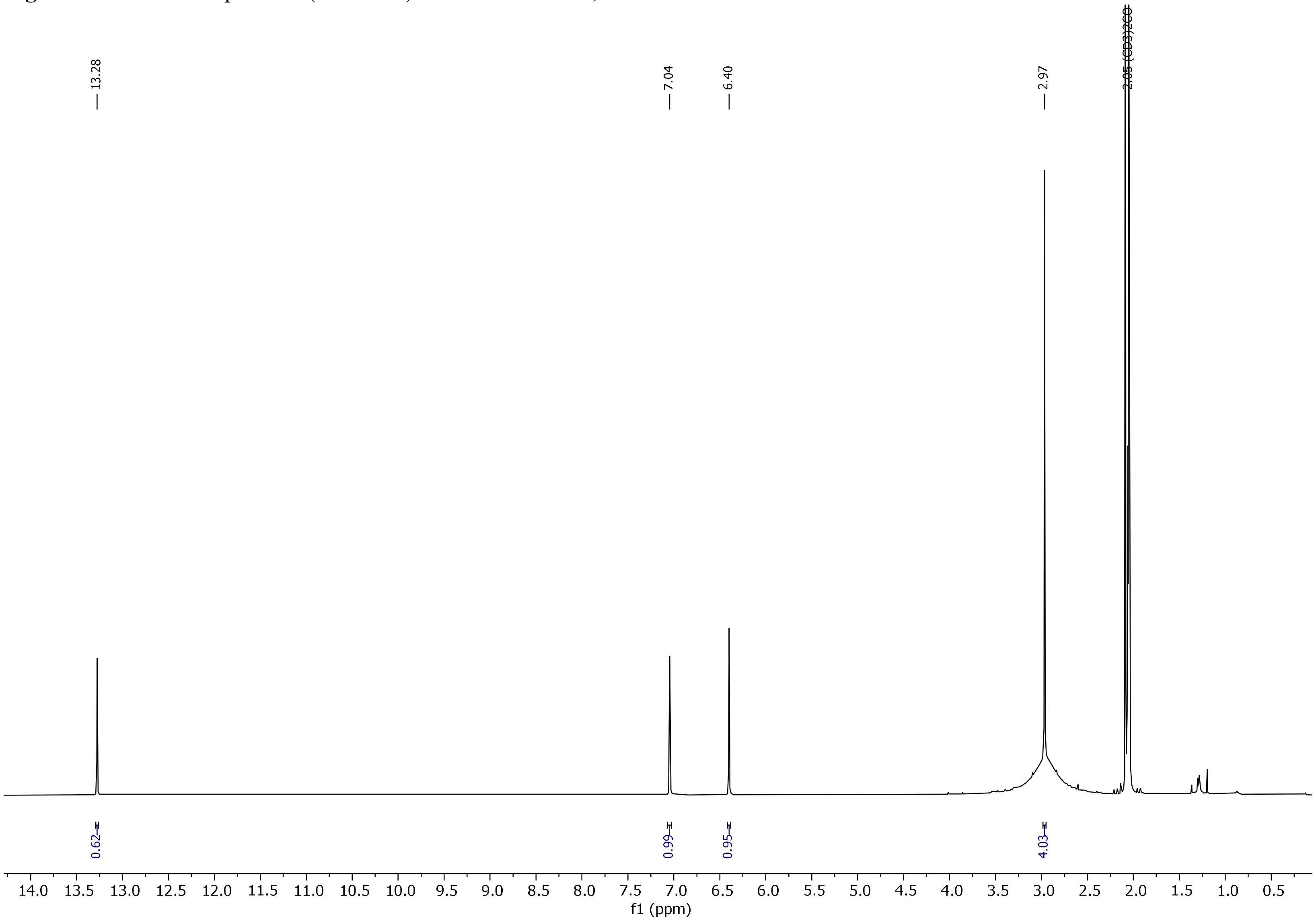

Figure S45. Jmod NMR spectrum (125 MHz) in acetone- d_6 of 4,7-dichloronorlichexanthone **10**

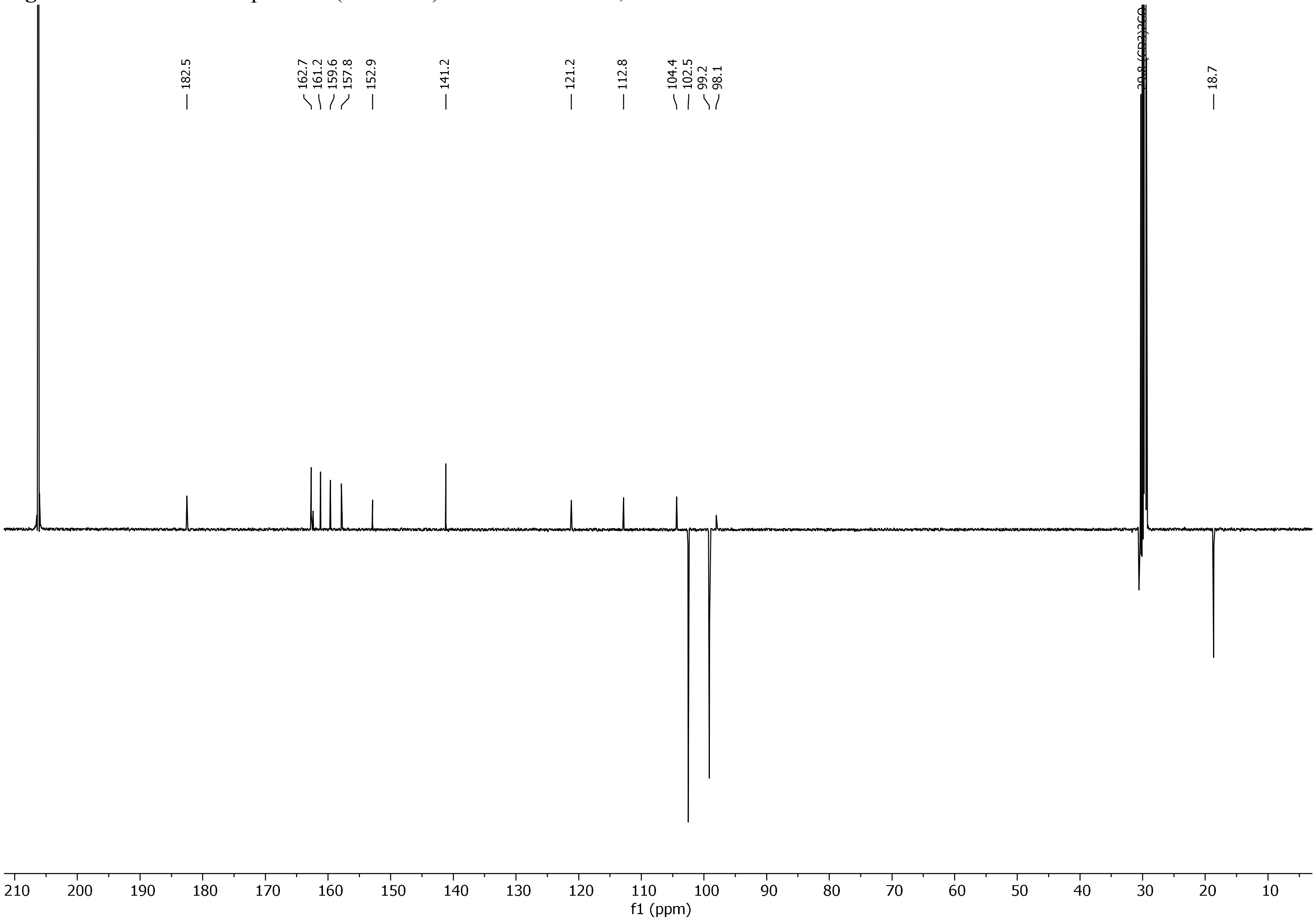

Figure S46. HSQC NMR spectrum (500/125 MHz) in acetone- d_6 of 4,7-dichloronorlichexanthone **10**

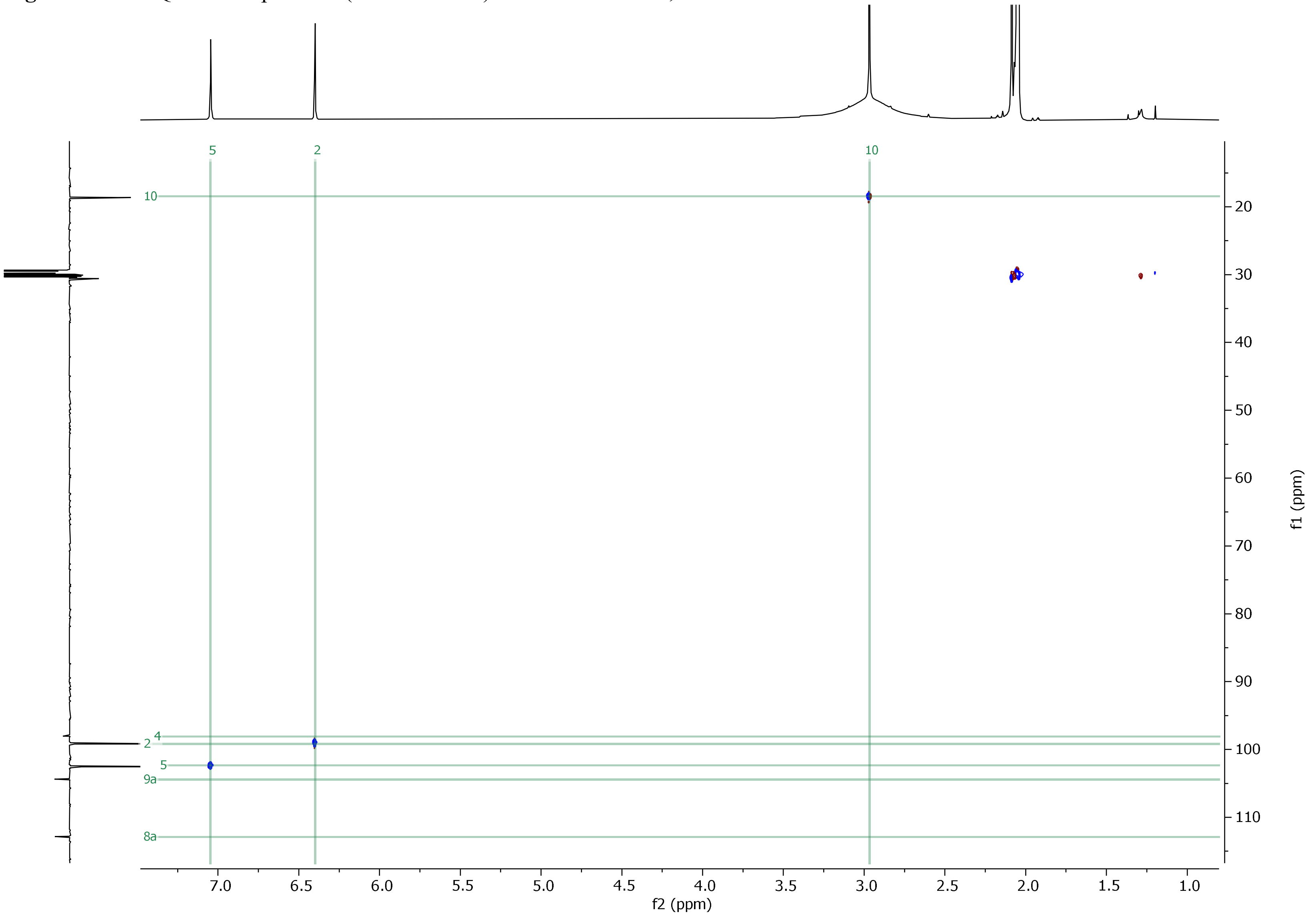

Figure S47. HMBC NMR spectrum (500/125 MHz) in acetone- d_6 of 4,7-dichloronorlichexanthone **10**

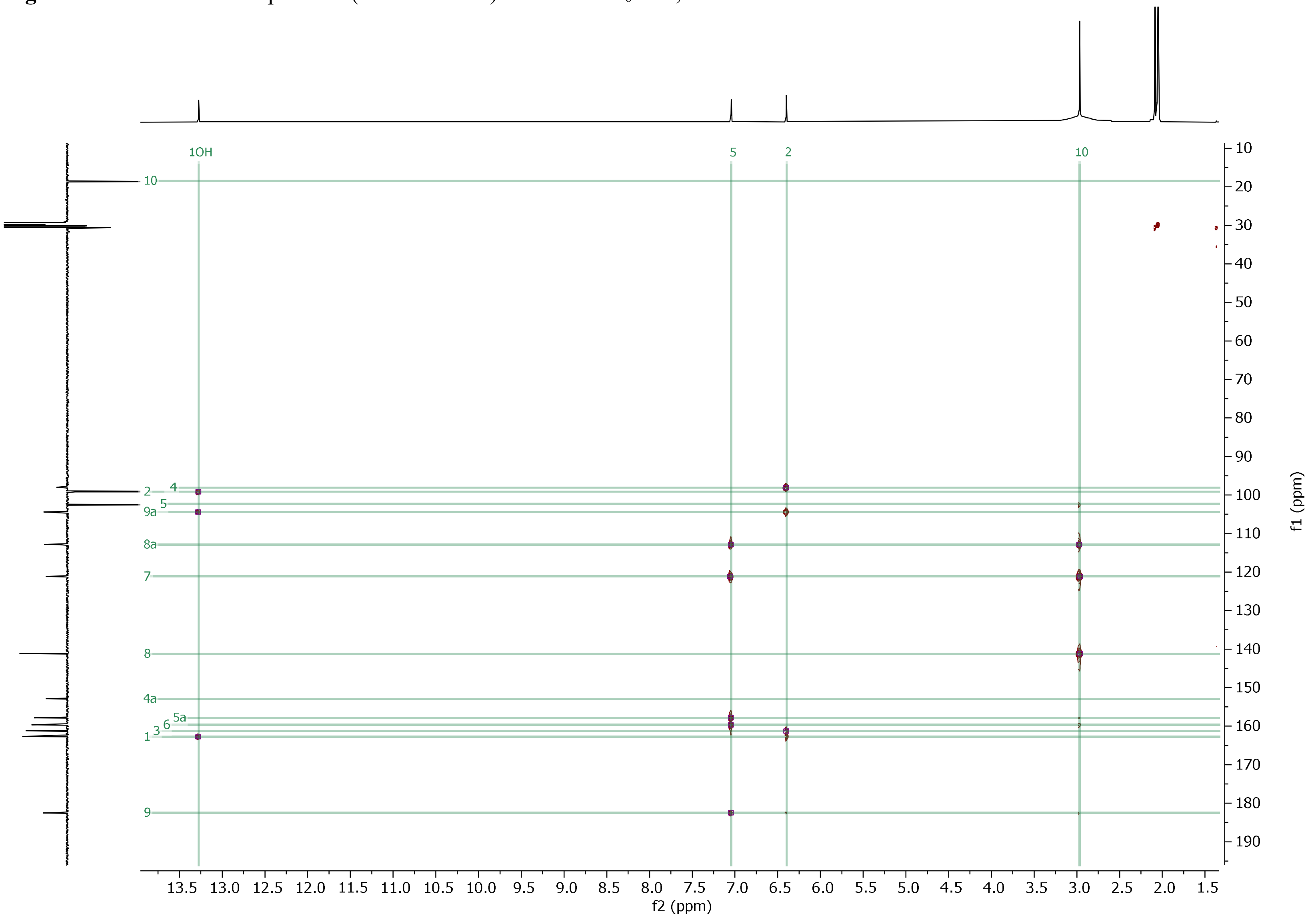

Figure S48. NOESY NMR spectrum (500 MHz) in acetone- d_6 of 4,7-dichloronorlichexanthone **10**

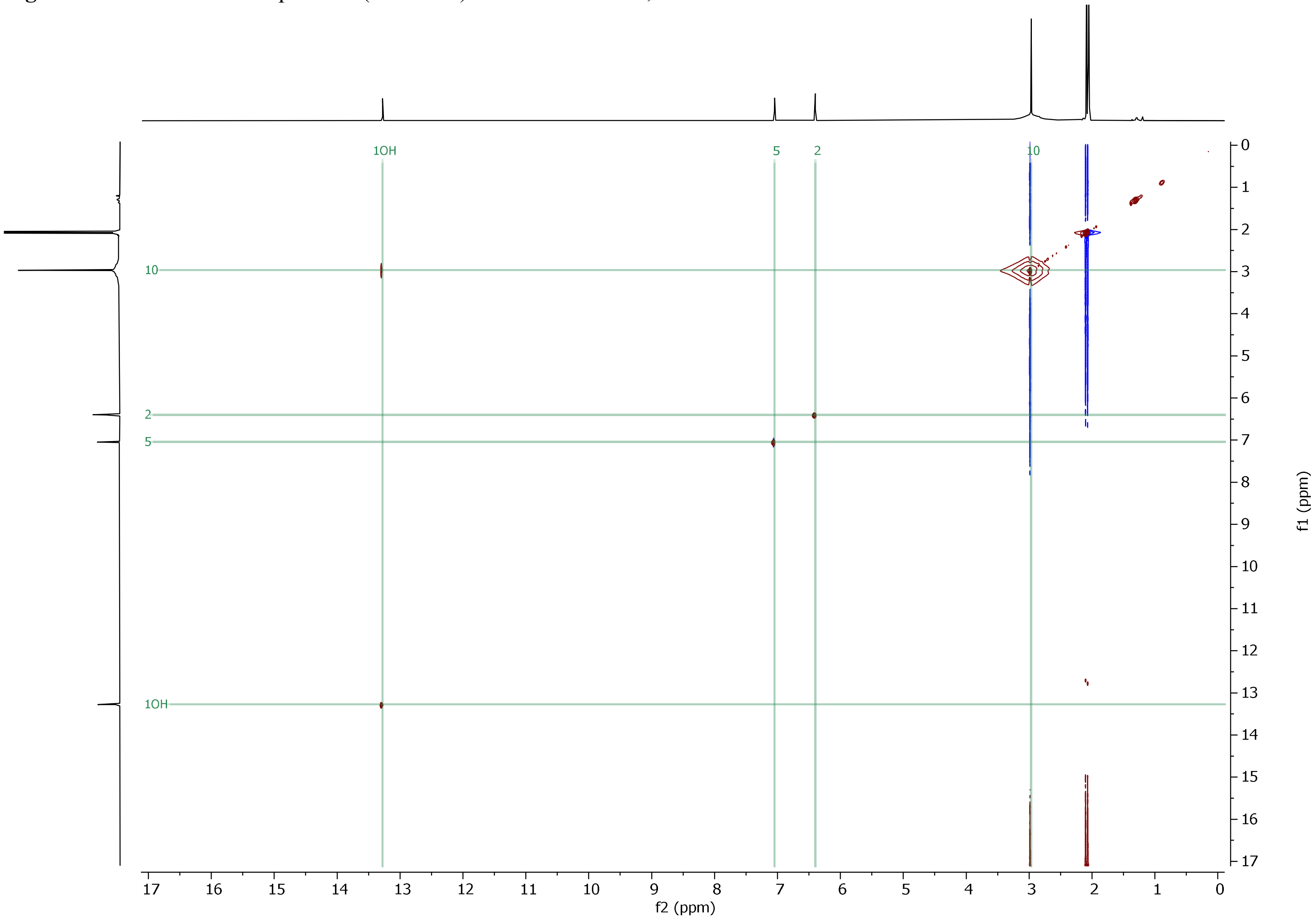

Figure S49. ^{1}H NMR spectrum (500 MHz) in acetone- d_6 of 5,7-dichloronorlichexanthone **11**

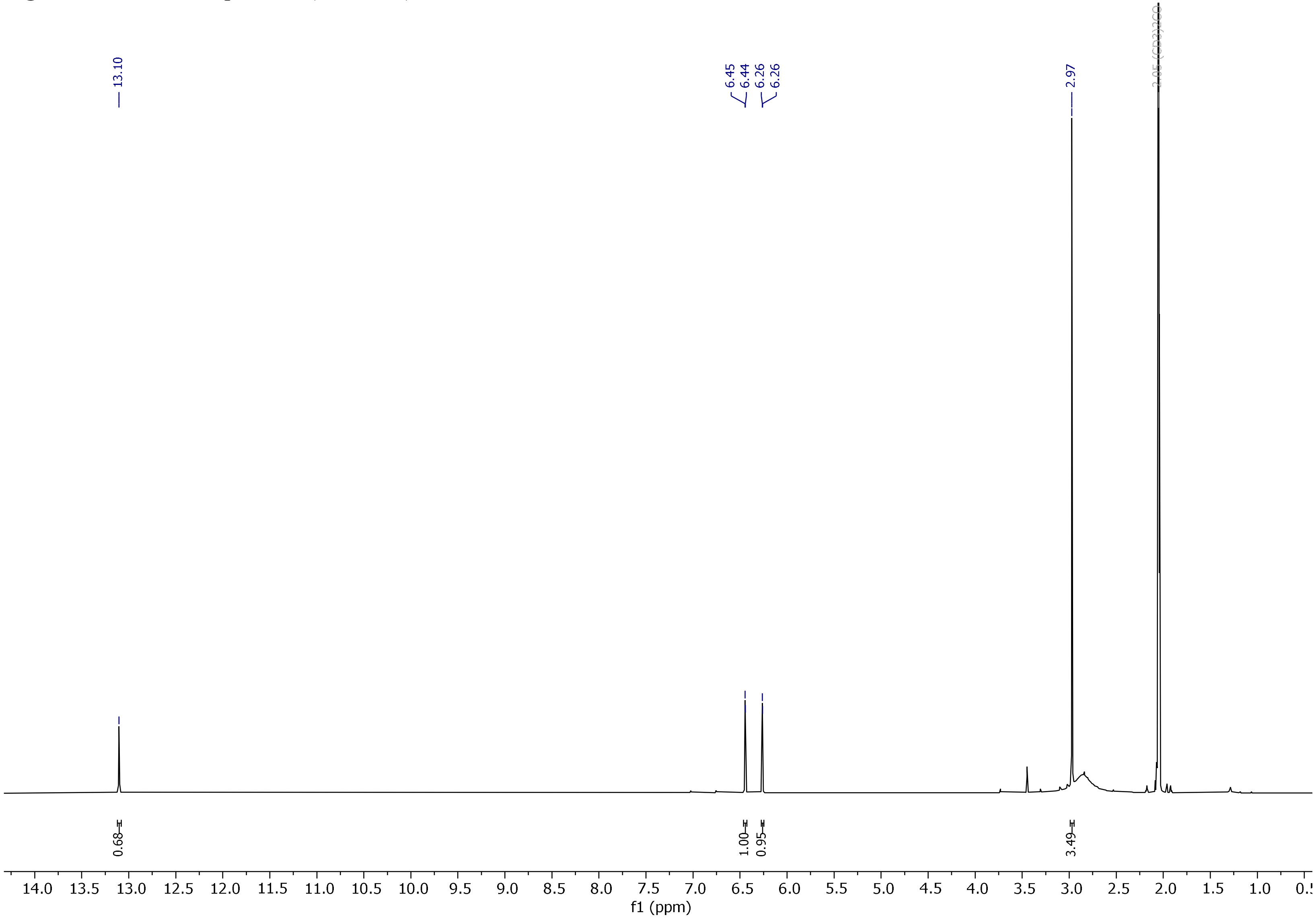

Figure S50. Jmod NMR spectrum (125 MHz) in acetone- d_6 of 5,7-dichloronorlichexanthone **11**

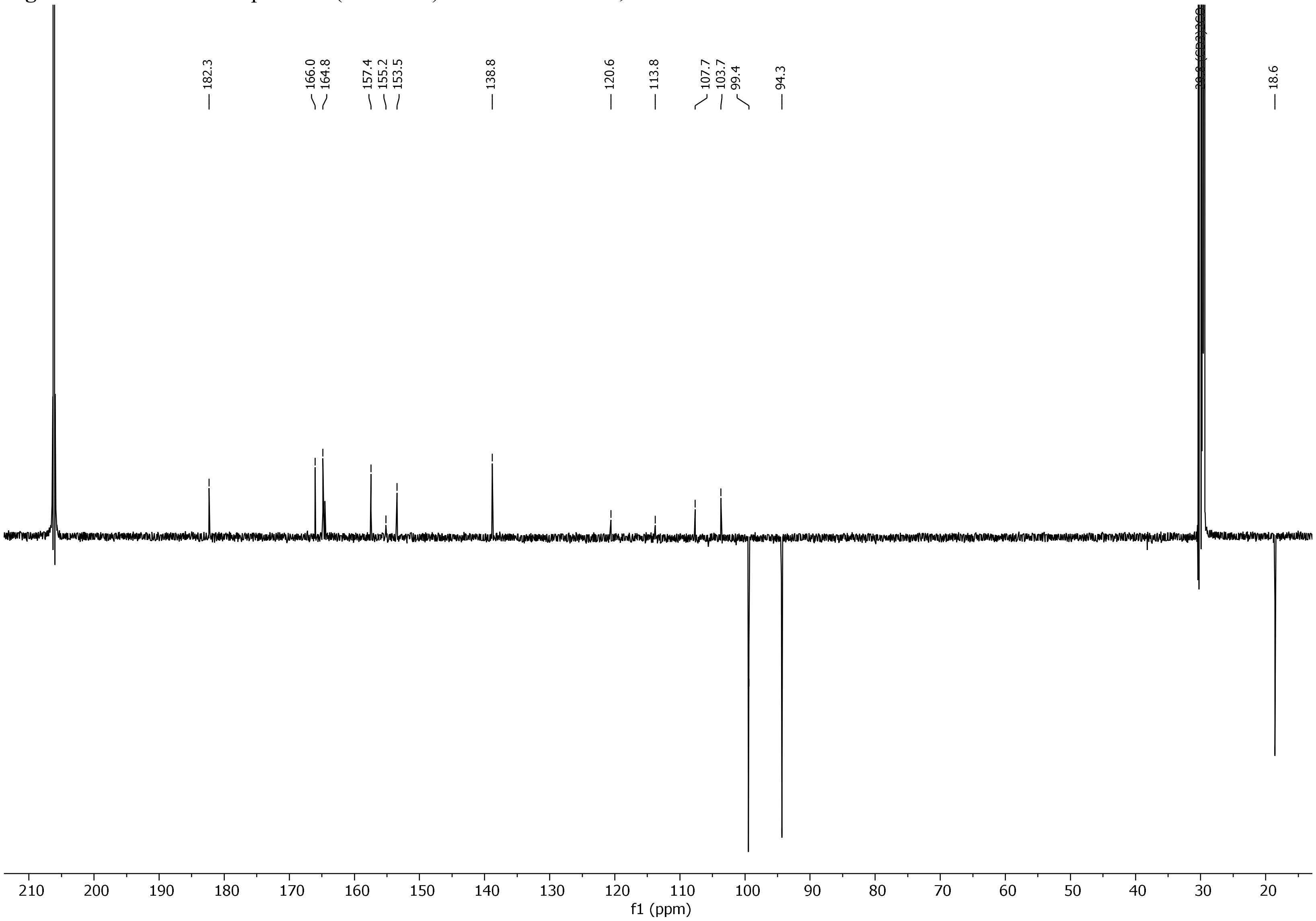

Figure S51. HSQC NMR spectrum (500/125 MHz) in acetone- d_6 of 5,7-dichloronorlichexanthone **11**

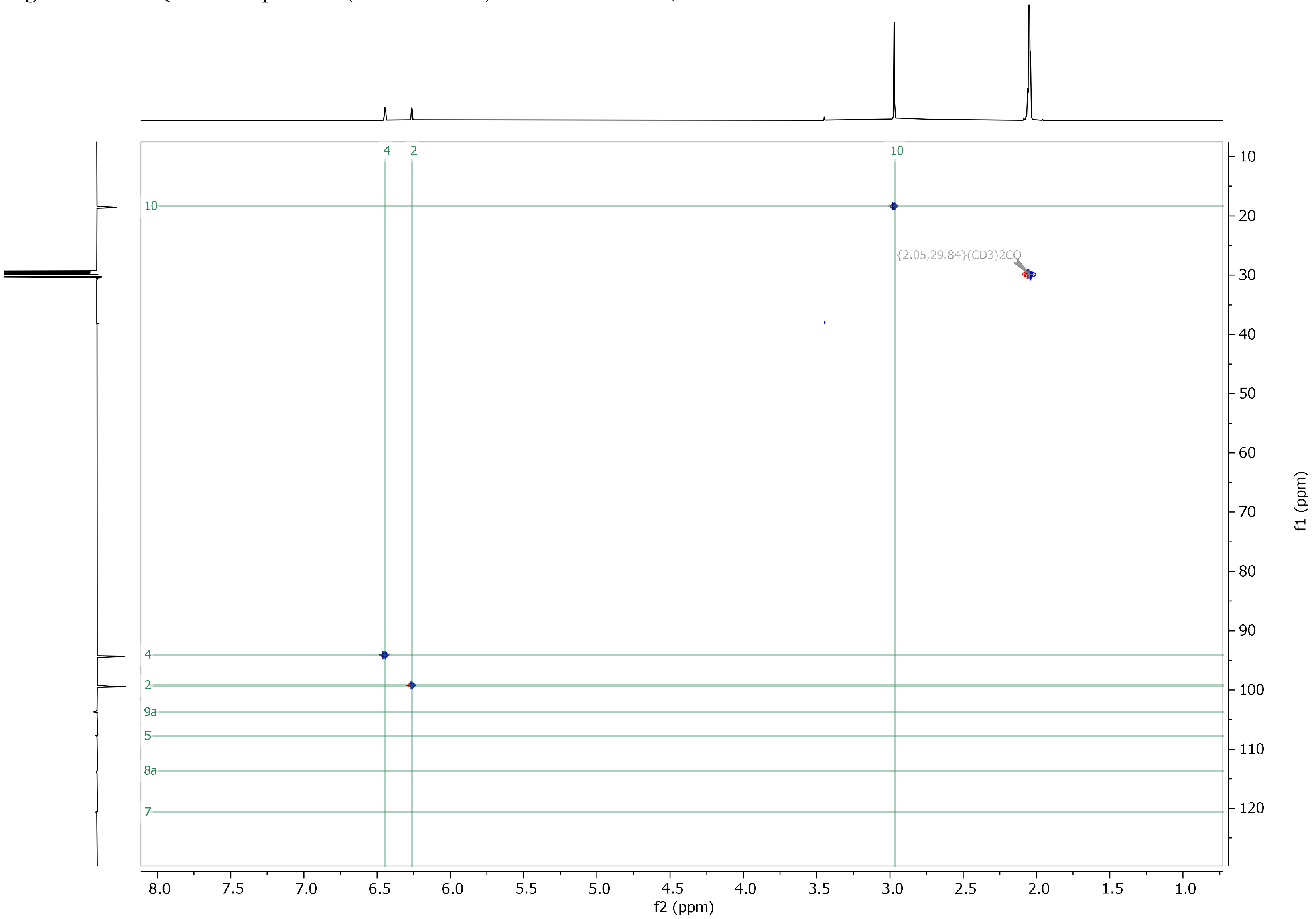

Figure S52. HMBC NMR spectrum (500/125 MHz) in acetone- d_6 of 5,7-dichloronorlichexanthone **11**

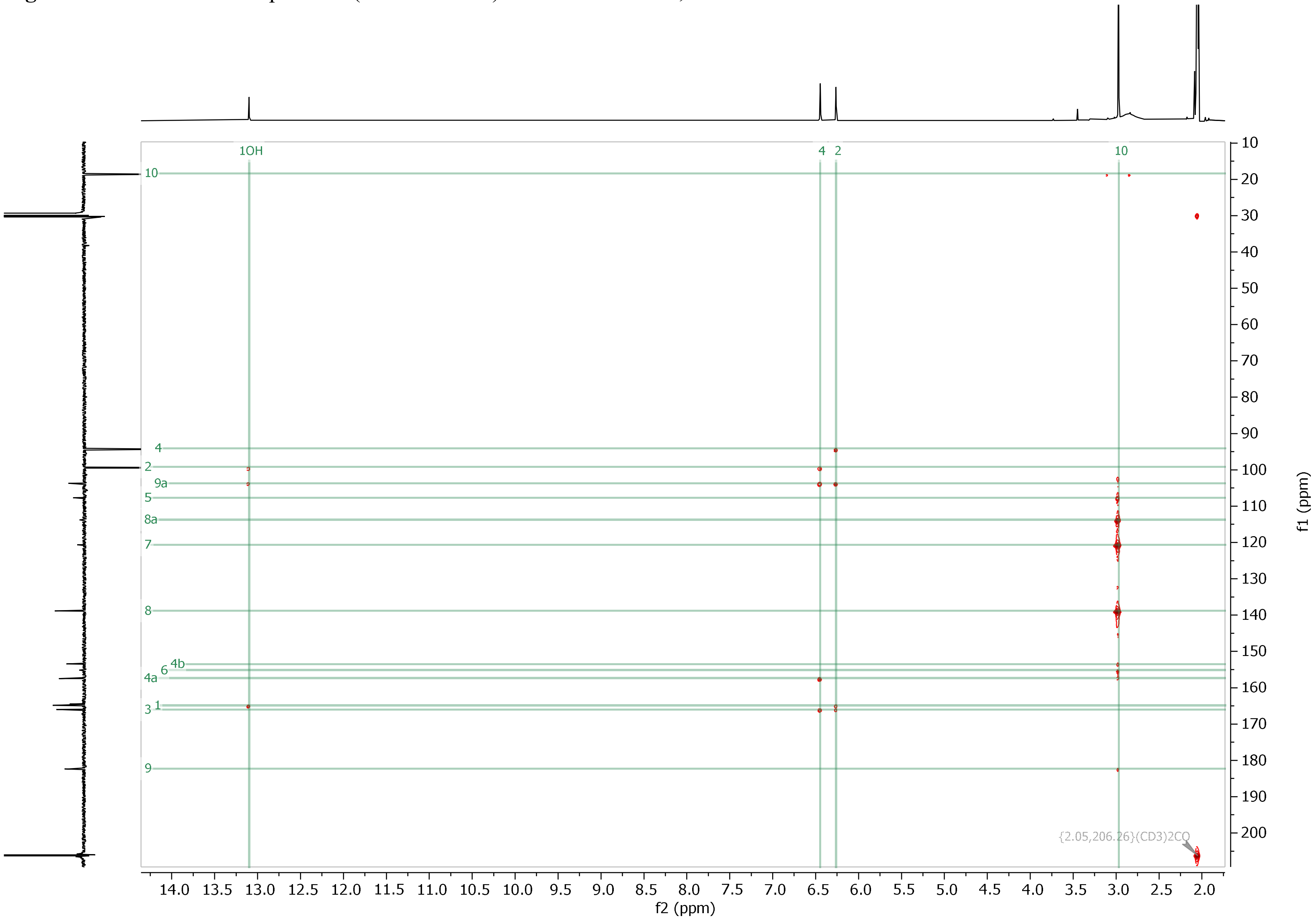

Figure S53. NOESY NMR spectrum (500 MHz) in acetone- d_6 of 5,7-dichloronorlichexanthone **11**

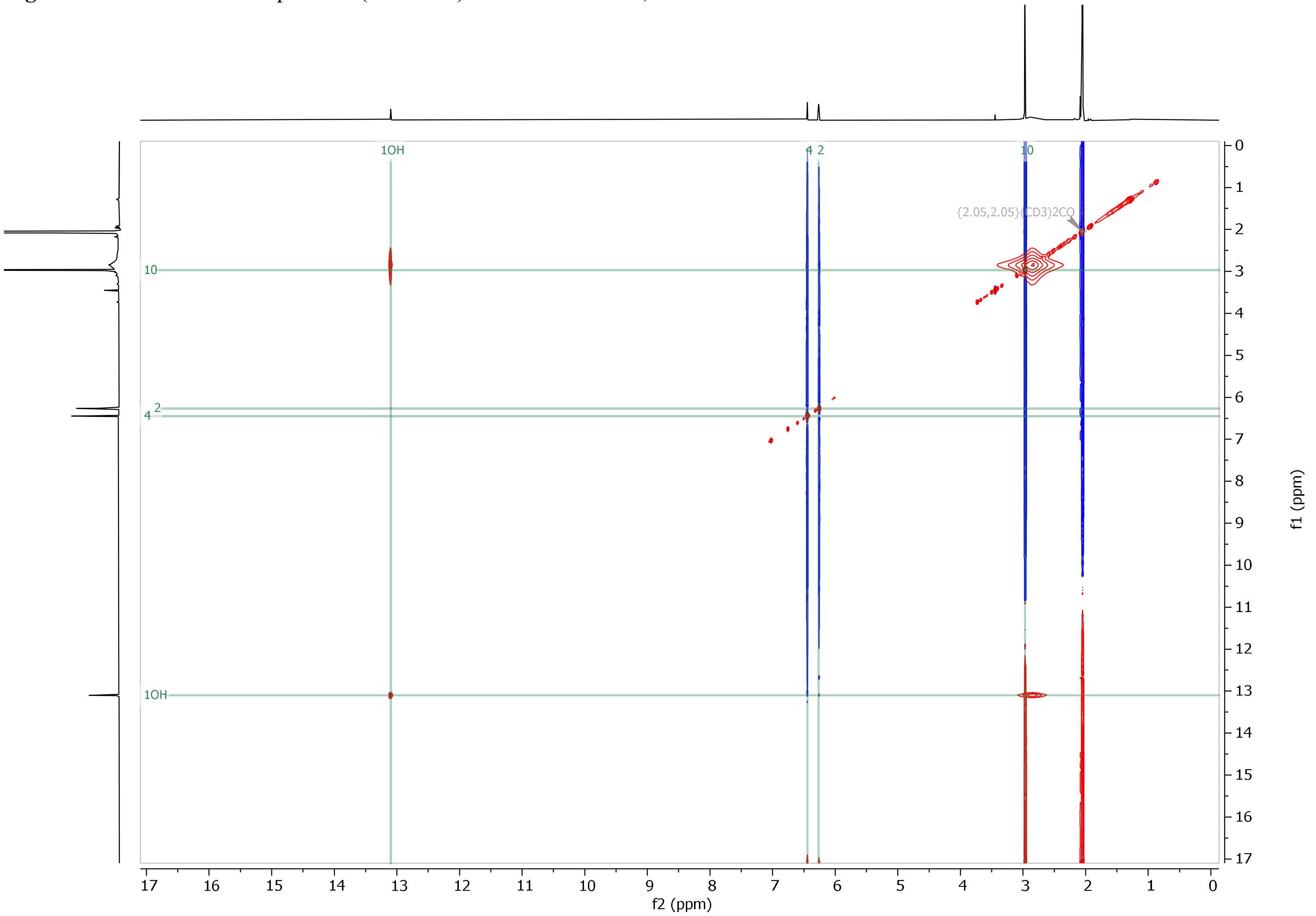

Figure S54. ^{1}H NMR spectrum (500 MHz) in acetone- d_6 of 2,4,5-trichloronorlichexanthone **12**

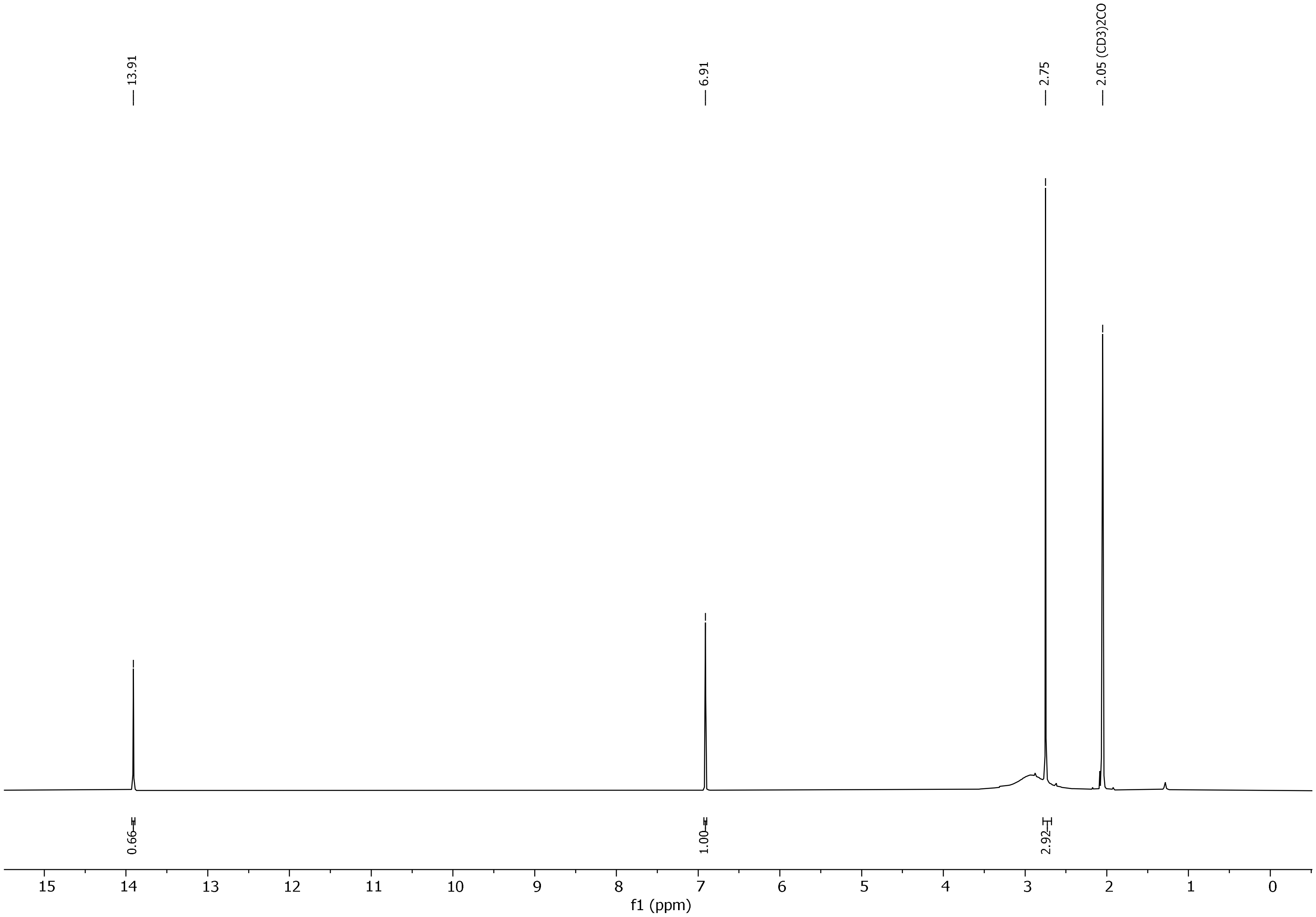

Figure S55. Jmod NMR spectrum (125 MHz) in acetone- d_6 of 2,4,5-trichloronorlichexanthone **12**

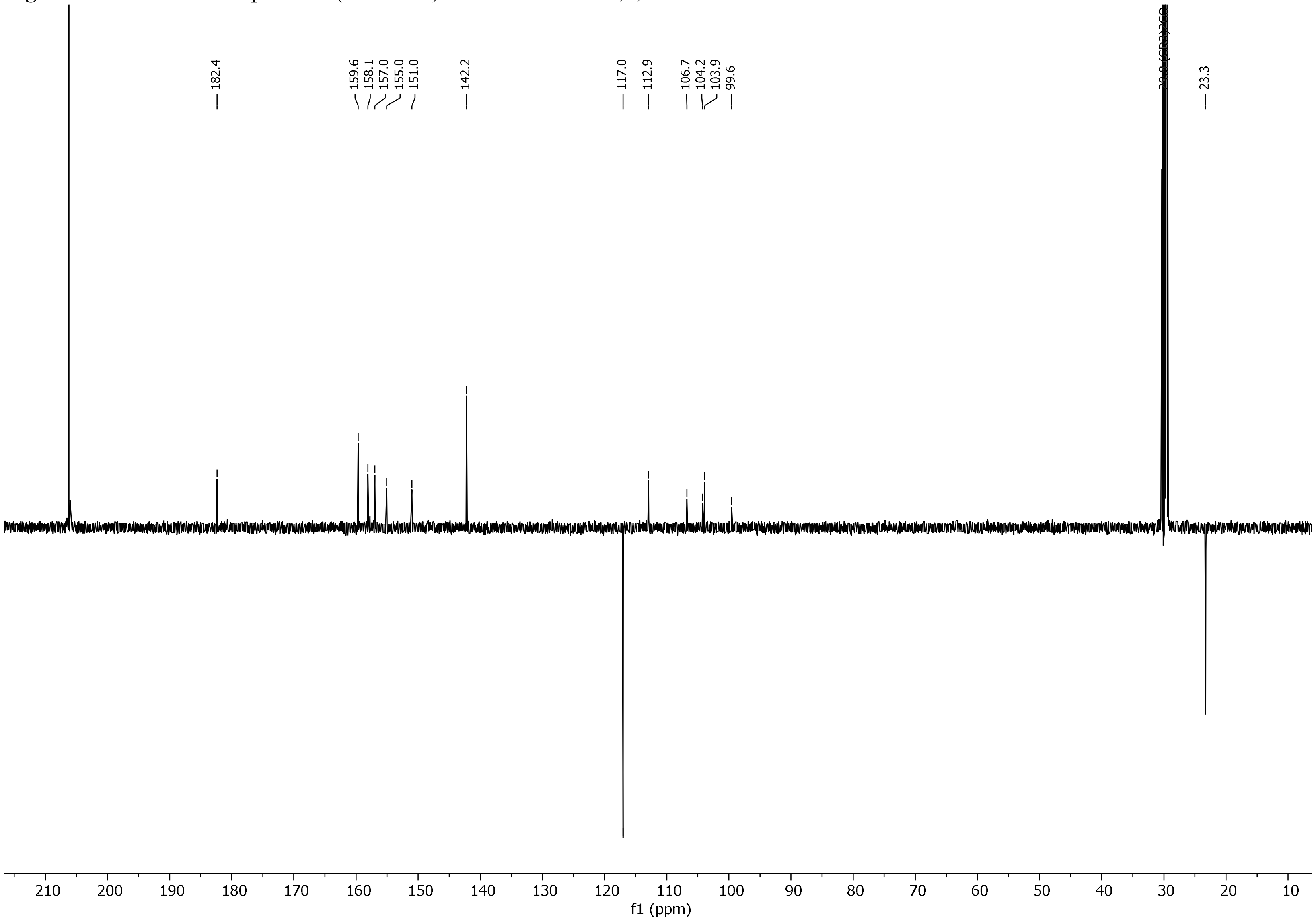

Figure S56. HSQC NMR spectrum (500/125 MHz) in acetone- d_6 of 2,4,5-trichloronorlichexanthone **12**

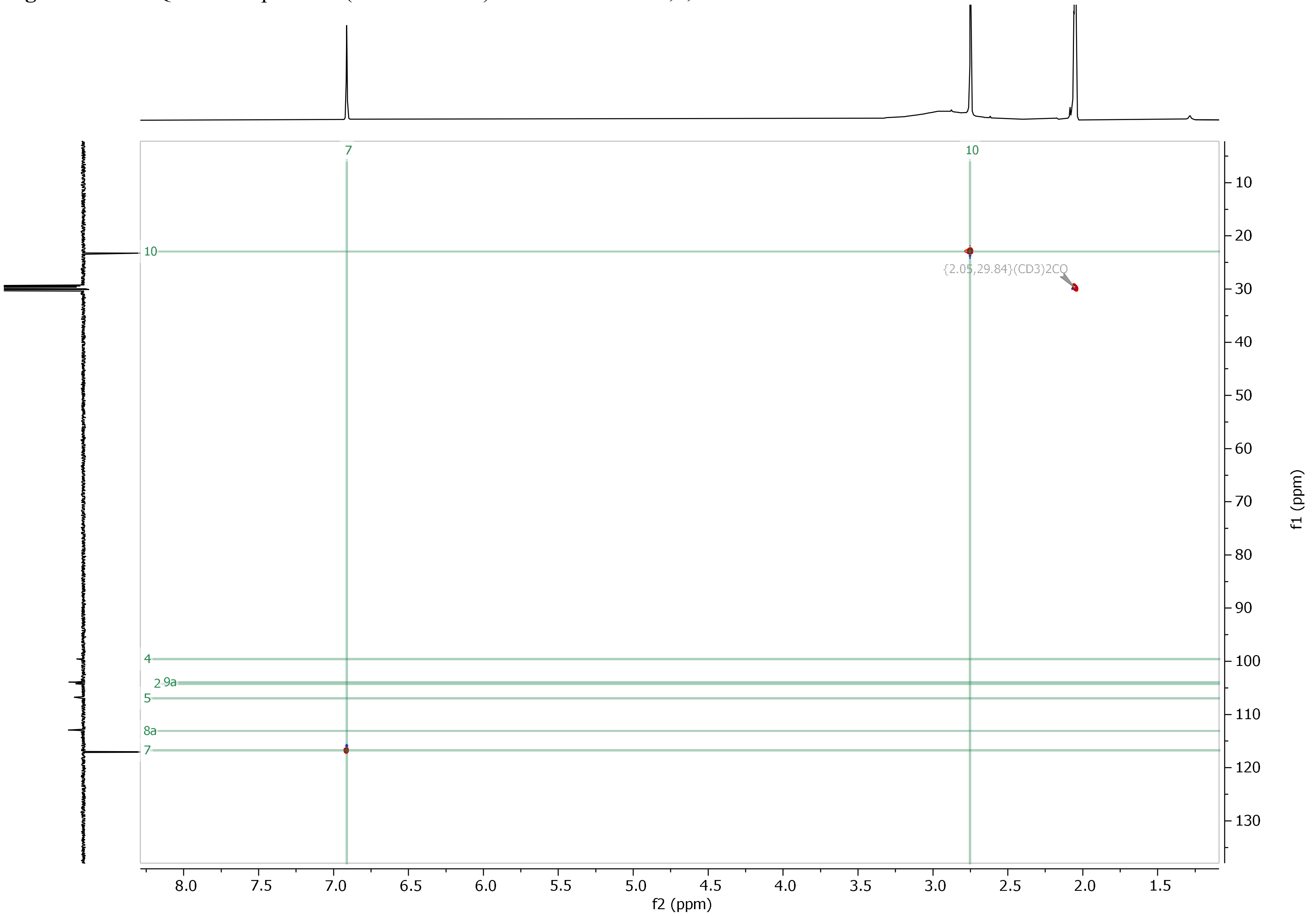

Figure S57. HMBC NMR spectrum (500/125 MHz) in acetone- d_6 of 2,4,5-trichloronorlichexanthone **12**

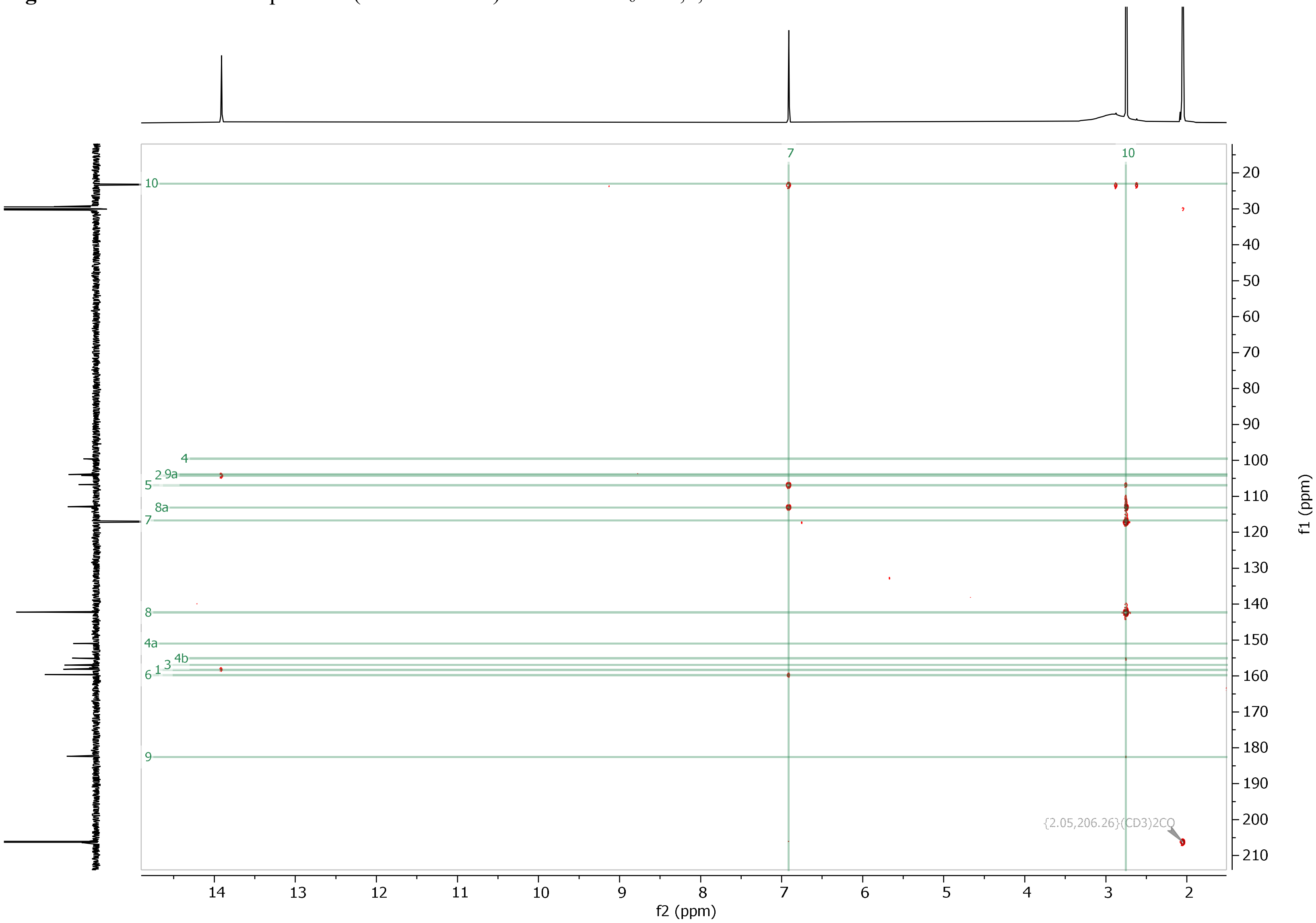

Figure S58. NOESY NMR spectrum (500 MHz) in acetone- d_6 of 2,4,5-trichloronorlichexanthone **12**

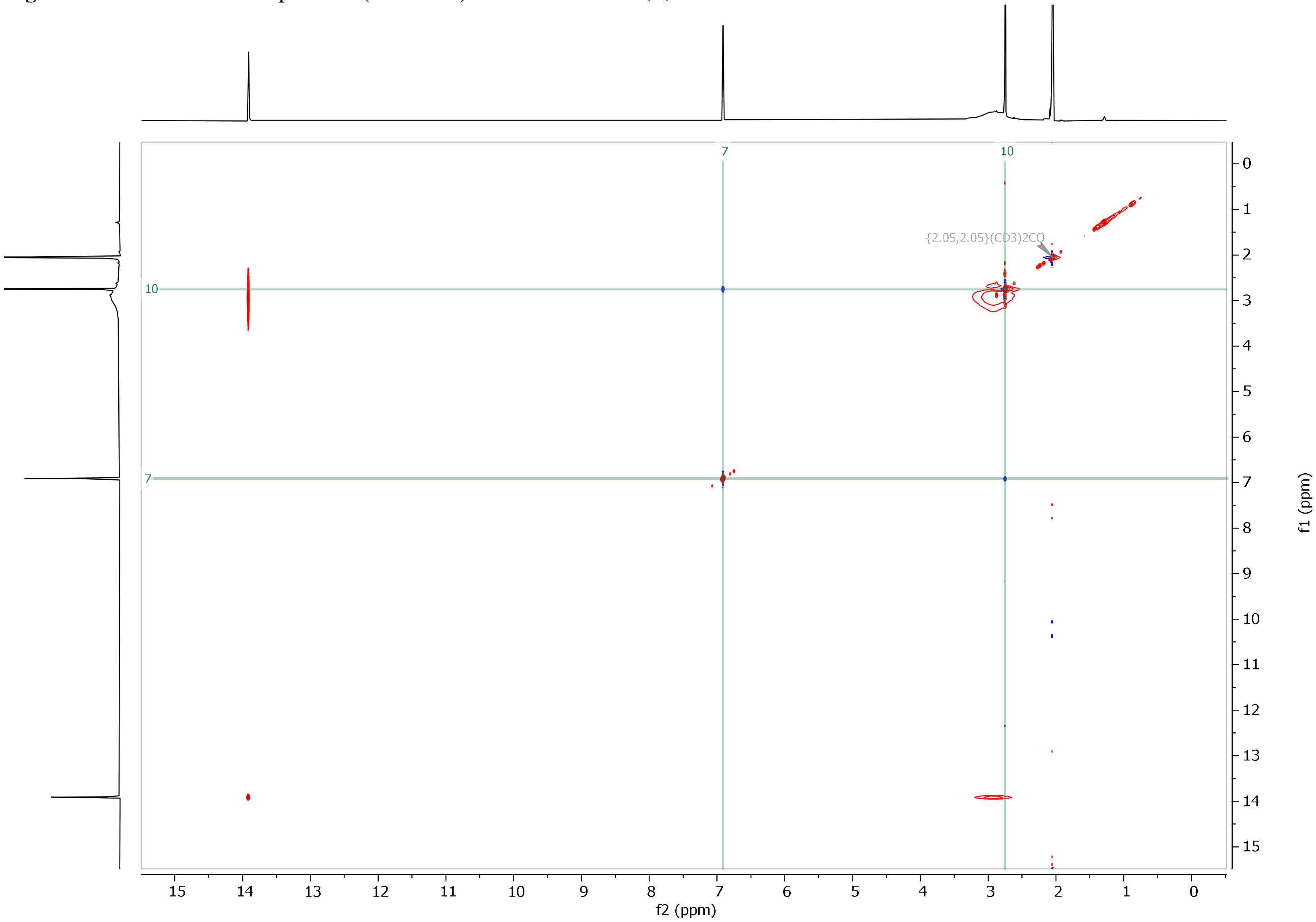

Figure S59. ^{1}H NMR spectrum (500 MHz) in acetone- d_6 of 2,4,7-trichloronorlichexanthone **13**

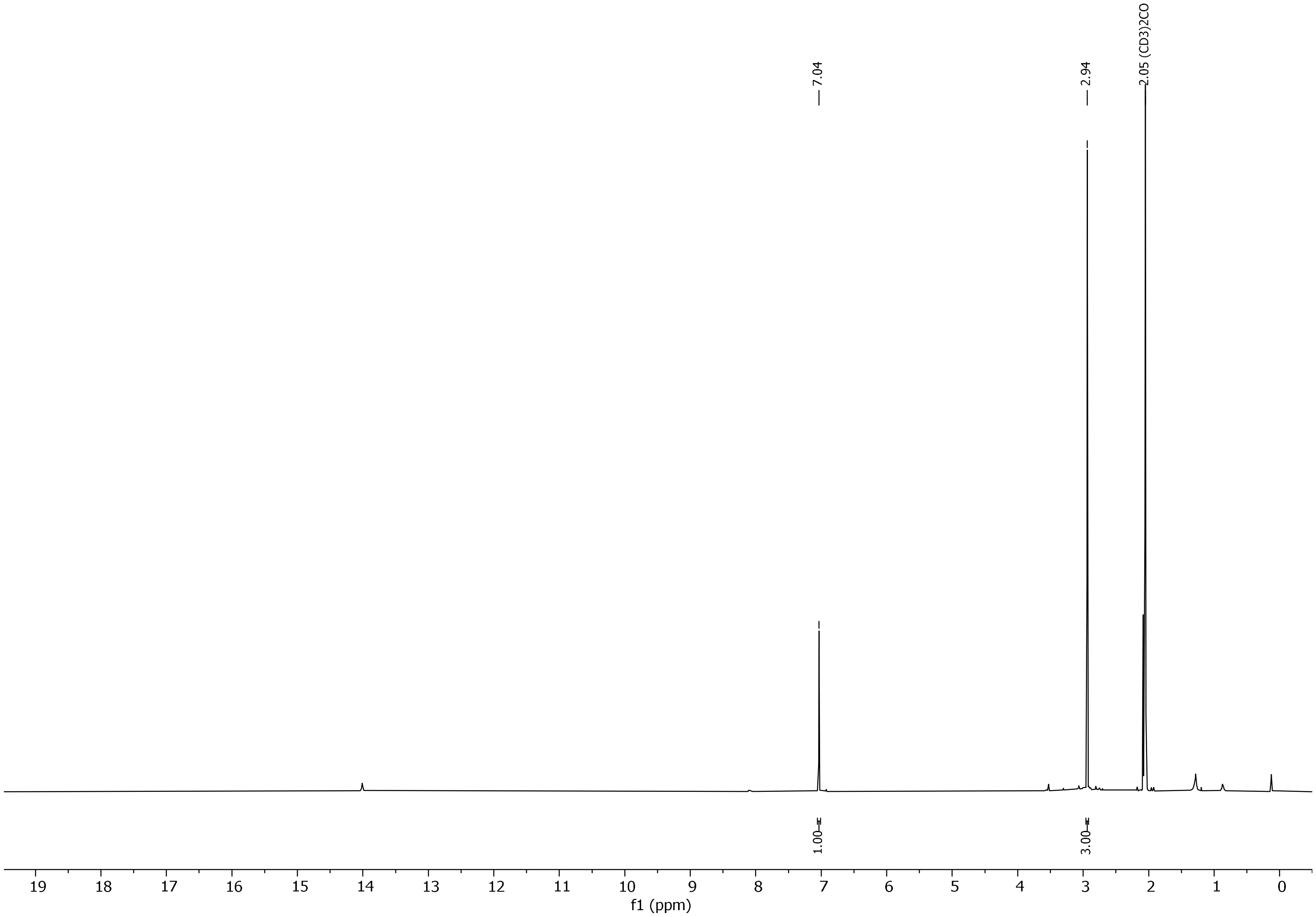

Figure S60. Jmod NMR spectrum (125 MHz) in acetone- d_6 of 2,4,7-trichloronorlichexanthone **13**

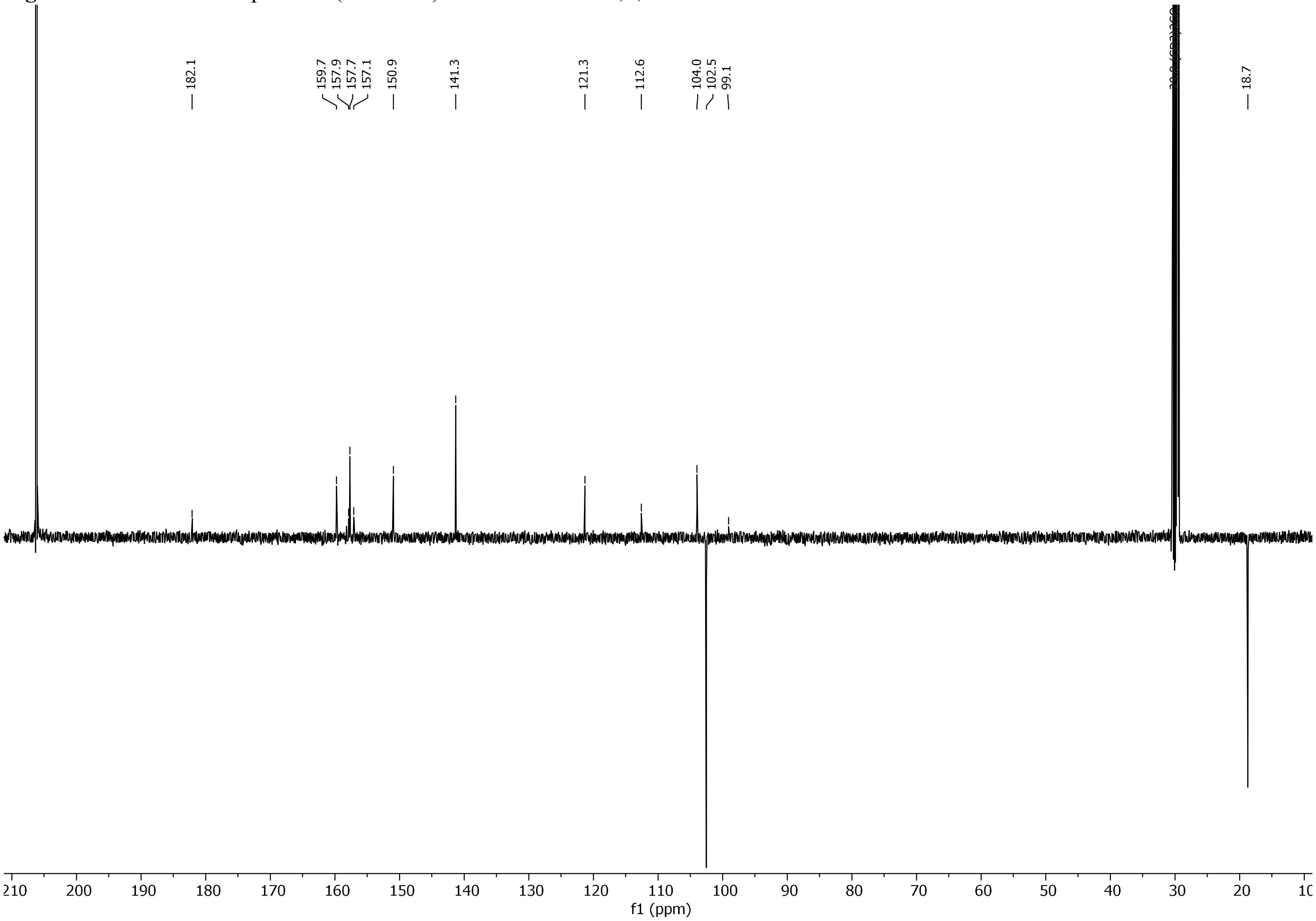

Figure S61. HSQC NMR spectrum (500/125 MHz) in acetone- d_6 of 2,4,7-trichloronorlichexanthone **13**

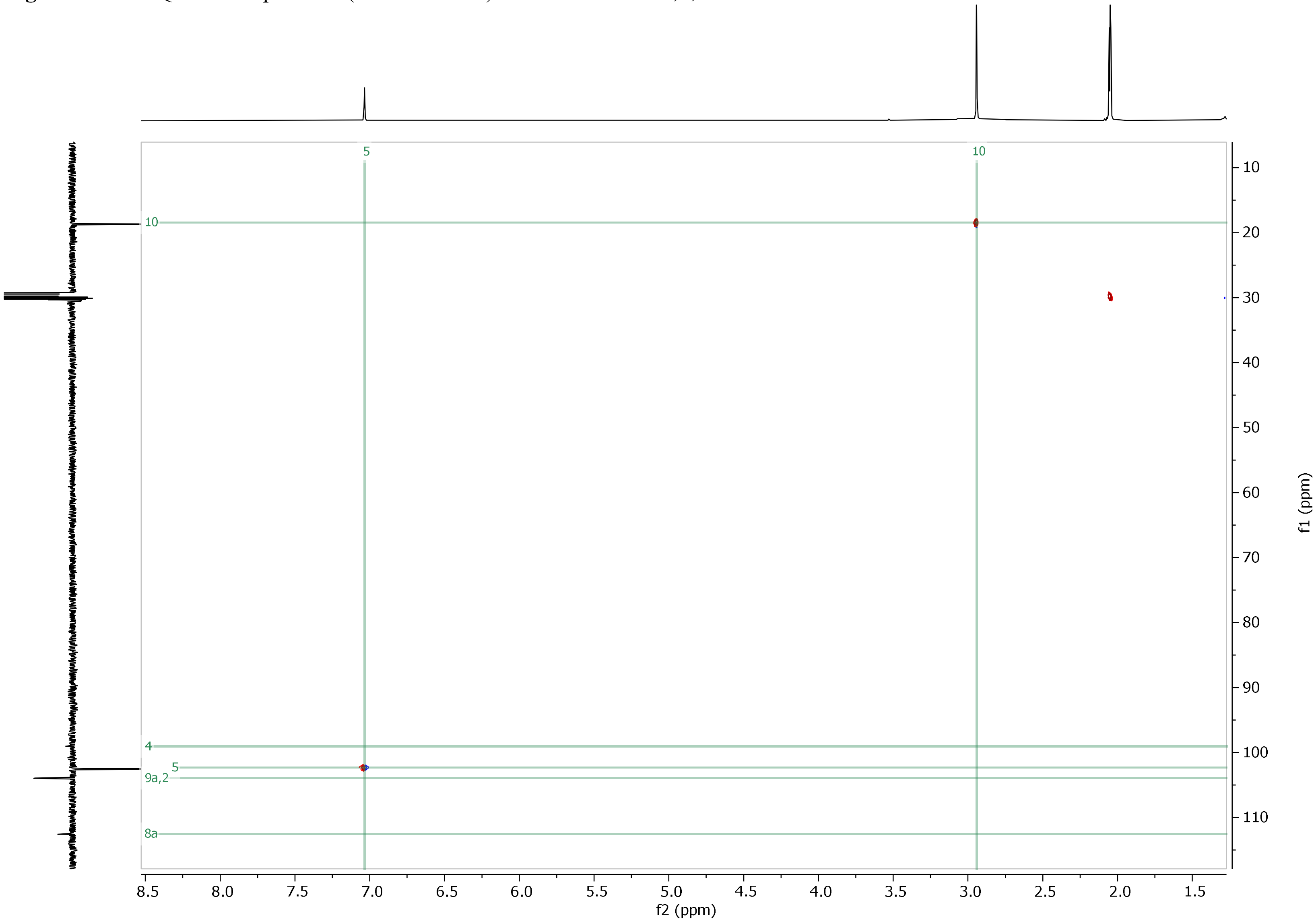

Figure S62. HMBC NMR spectrum (500/125 MHz) in acetone- d_6 of 2,4,7-trichloronorlichexanthone **13**

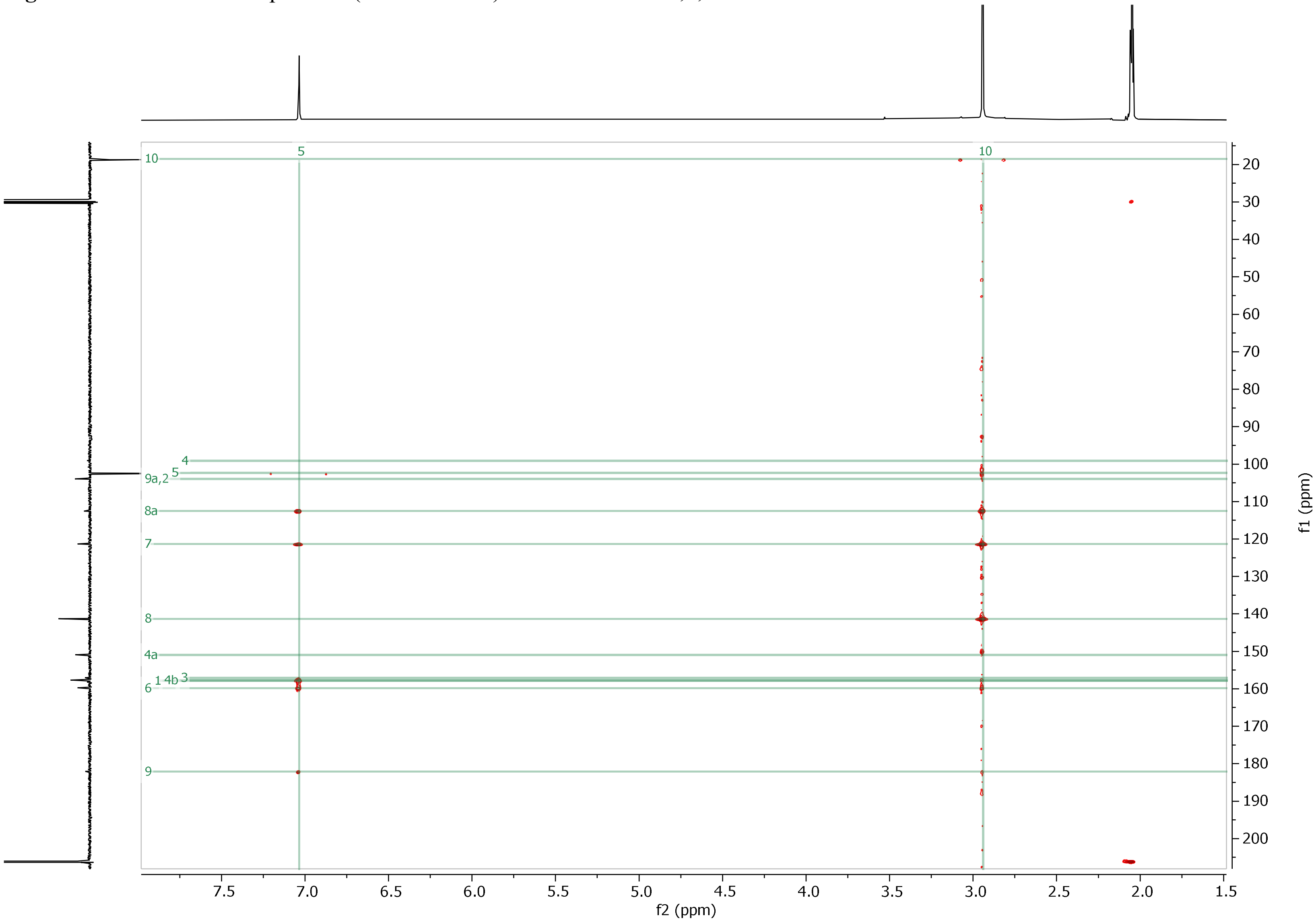

Figure S63. ^{1}H NMR spectrum (500 MHz) in acetone- d_6 of 2,5,7-trichloronorlichexanthone **14**

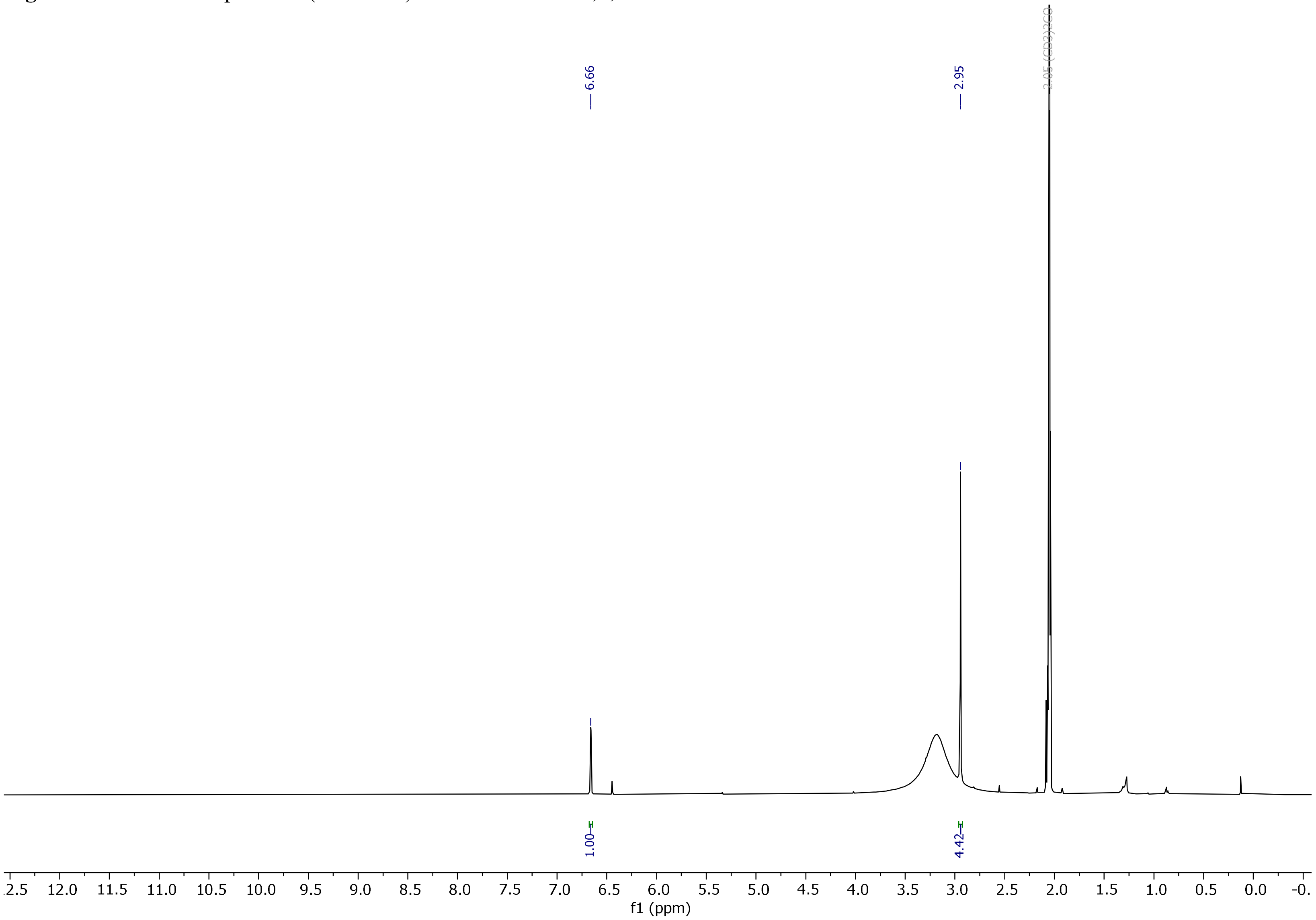

Figure S64. Jmod NMR spectrum (125 MHz) in acetone- d_6 of 2,5,7-trichloronorlichexanthone **14**

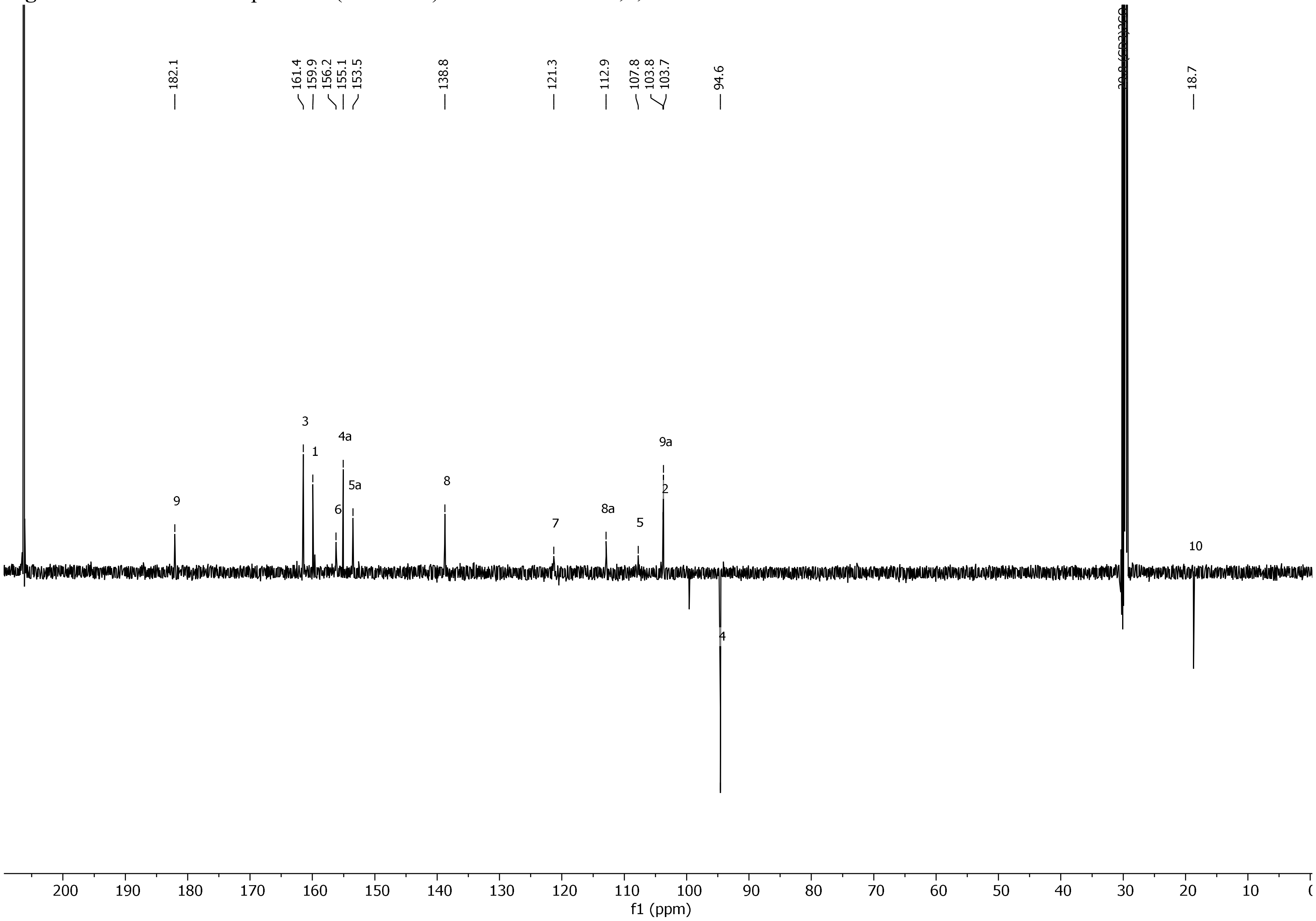

Figure S65. HSQC NMR spectrum (500/125 MHz) in acetone- d_6 of 2,5,7-trichloronorlichexanthone **14**

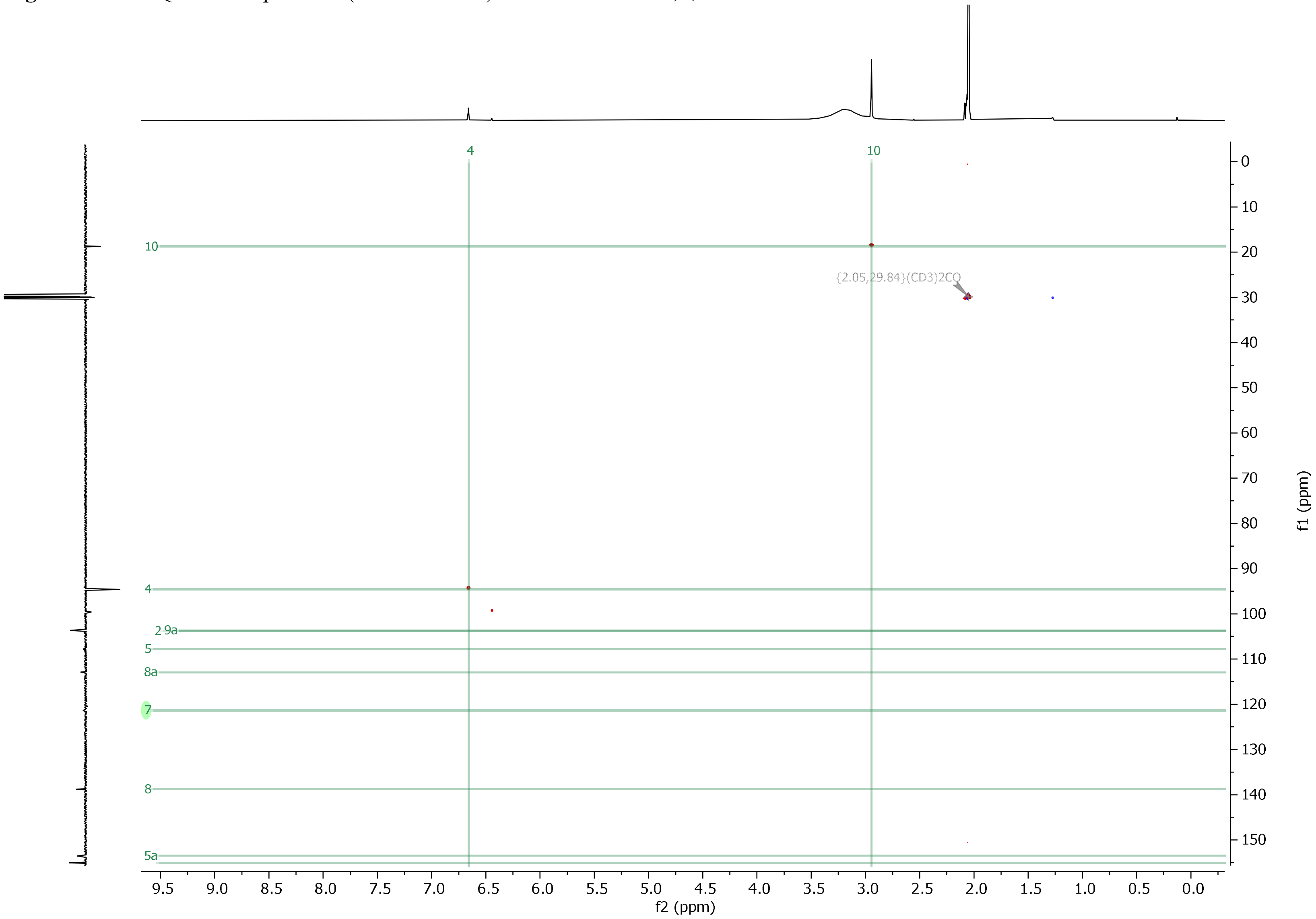

Figure S66. HMBC NMR spectrum (500/125 MHz) in acetone- d_6 of 2,5,7-trichloronorlichexanthone **14**

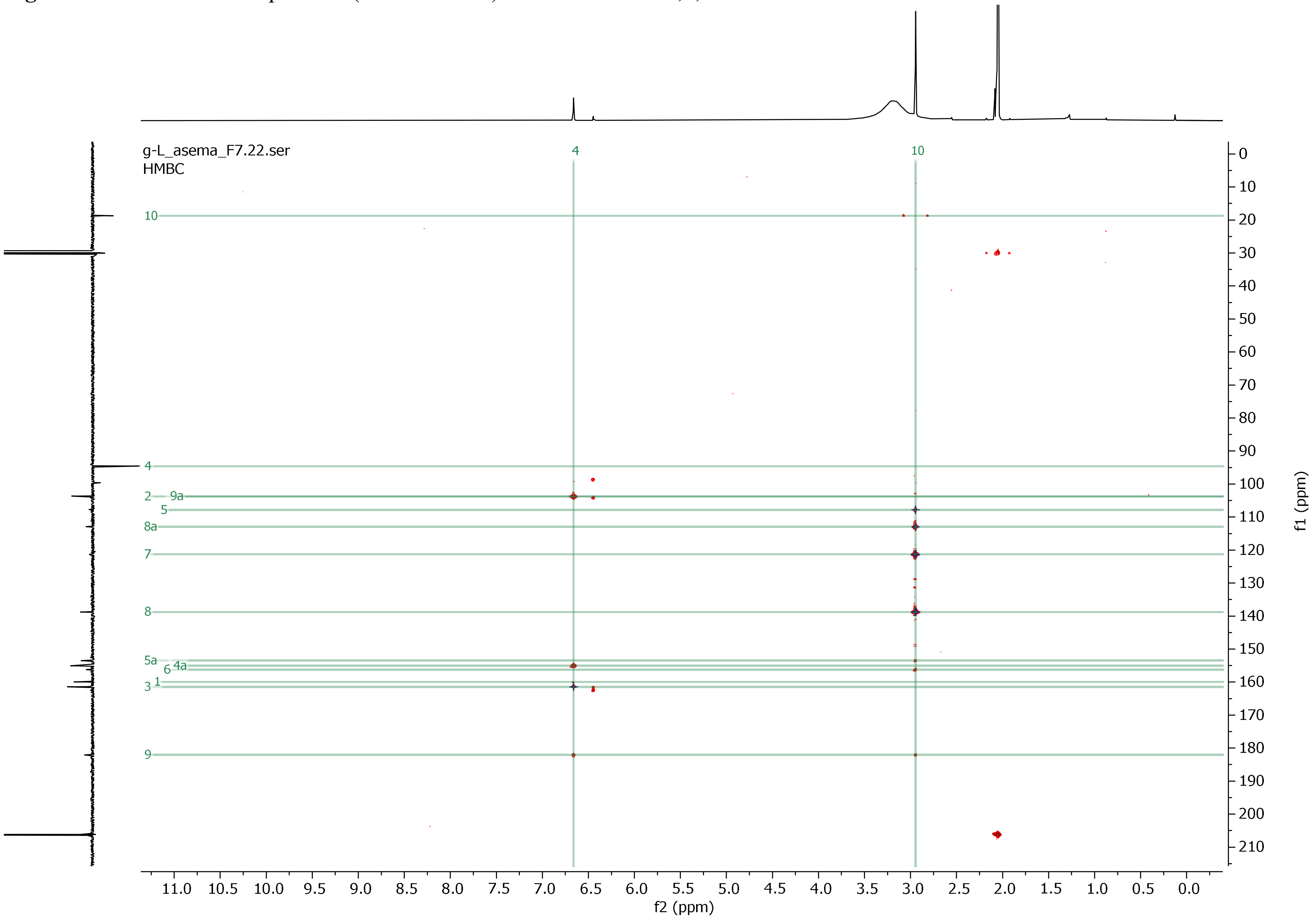

Figure S67. NOESY NMR spectrum (500 MHz) in acetone- d_6 of 2,5,7-trichloronorlichexanthone **14**

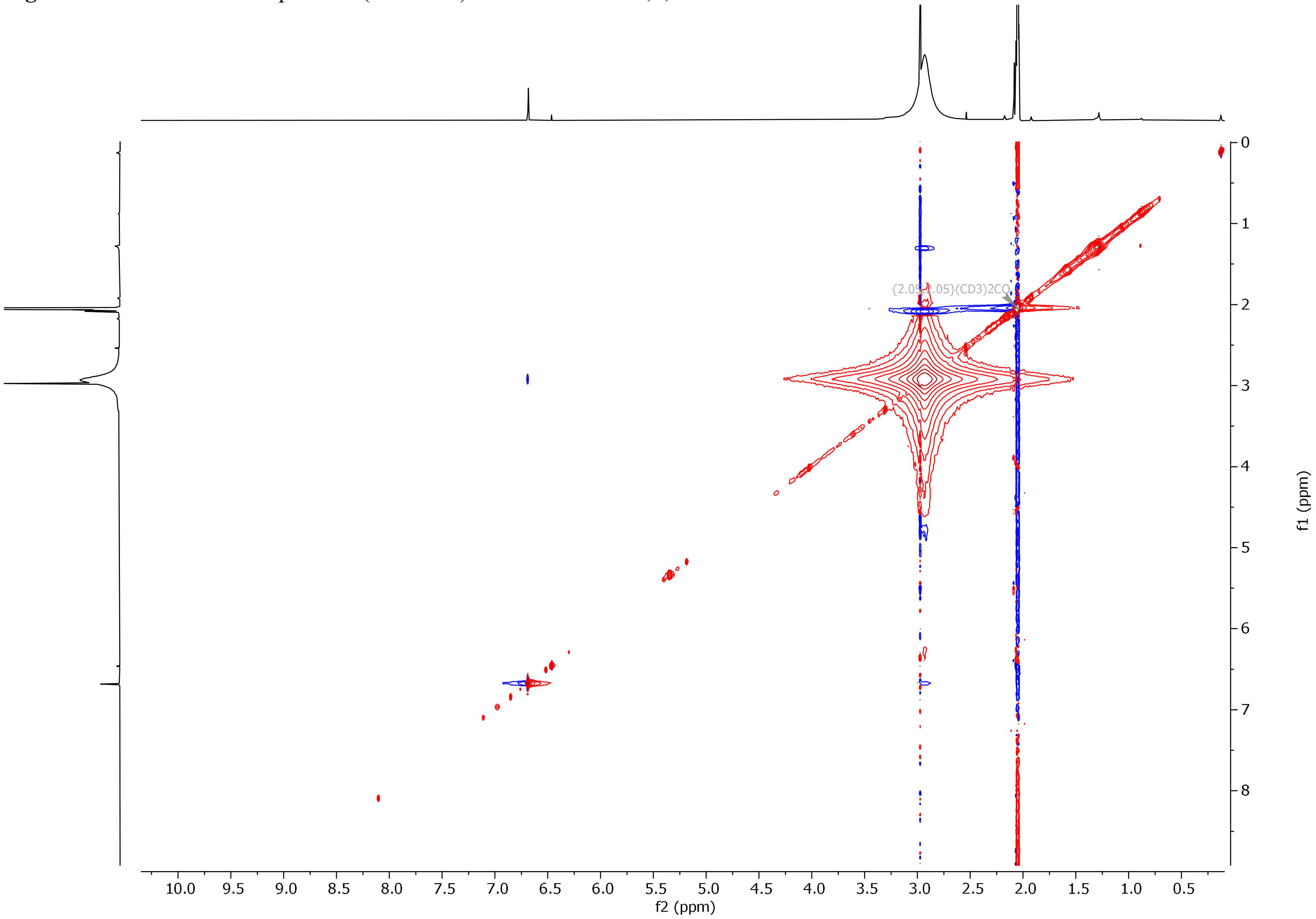

Figure S68. ^{1}H NMR spectrum (500 MHz) in acetone- d_6 of 4,5,7-trichloronorlichexanthone **15**

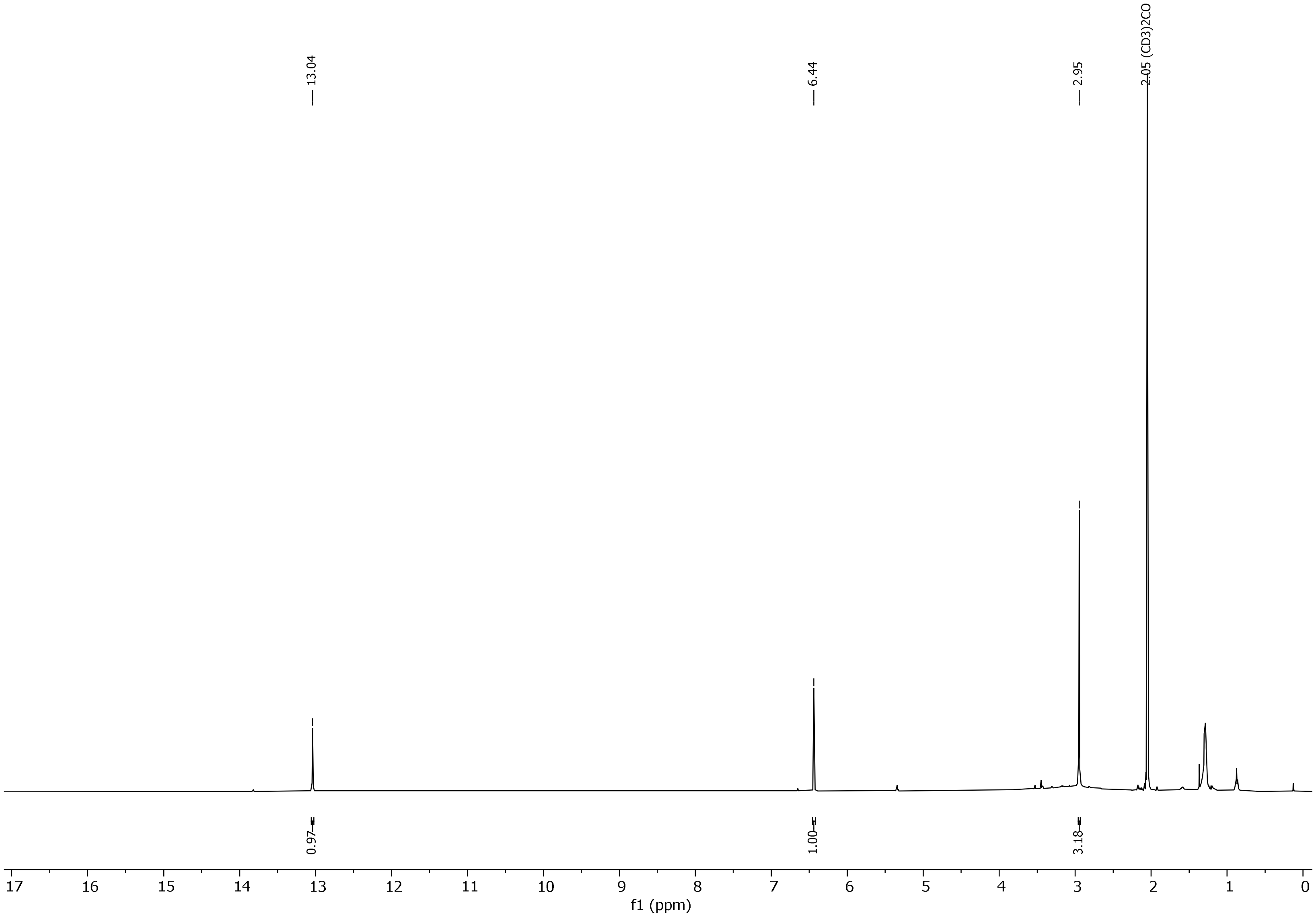

Figure S69. Jmod NMR spectrum (125 MHz) in acetone- d_6 of 4,5,7-trichloronorlichexanthone **15**

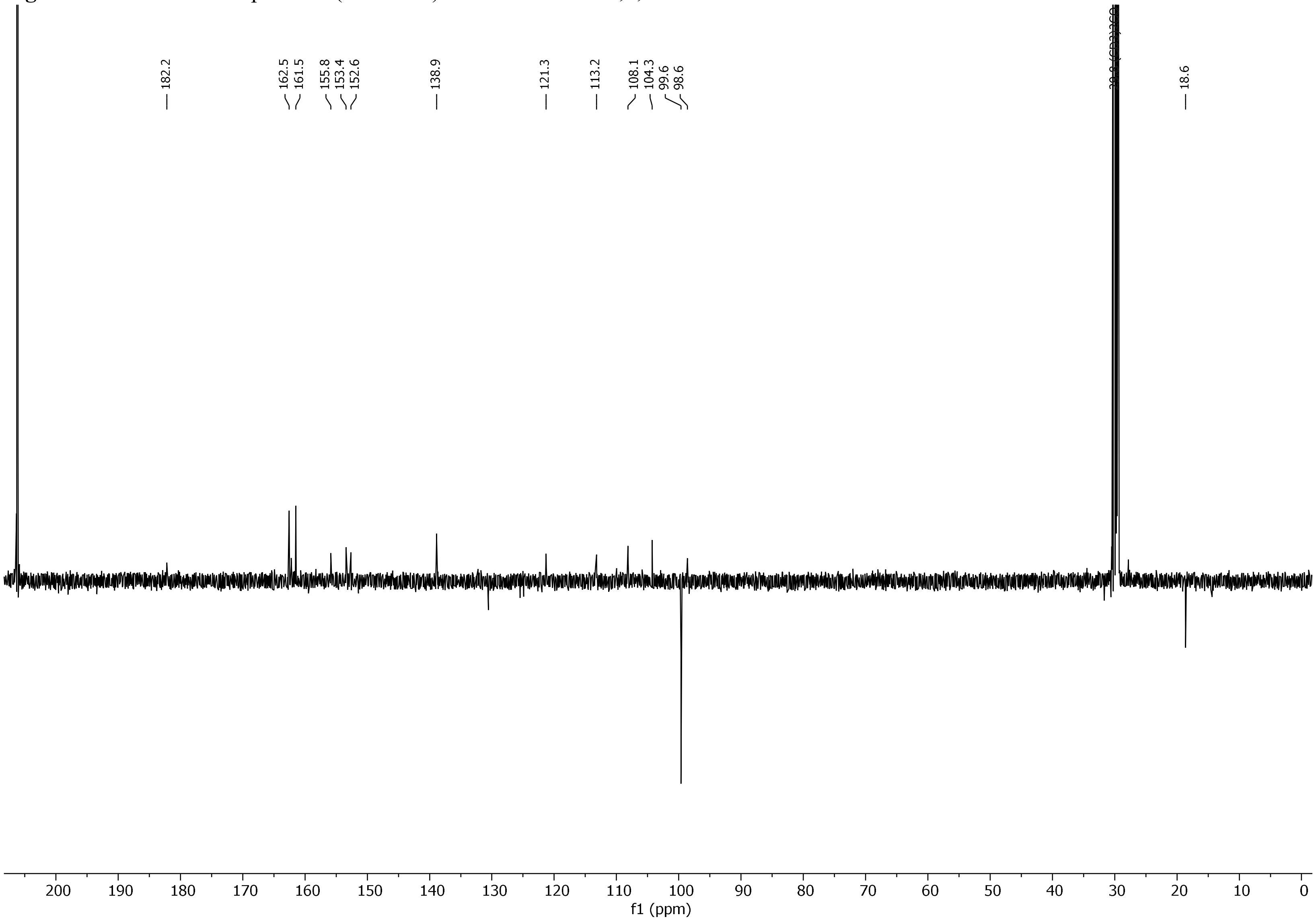

Figure S70. HSQC NMR spectrum (500/125 MHz) in acetone- d_6 of 4,5,7-trichloronorlichexanthone **15**

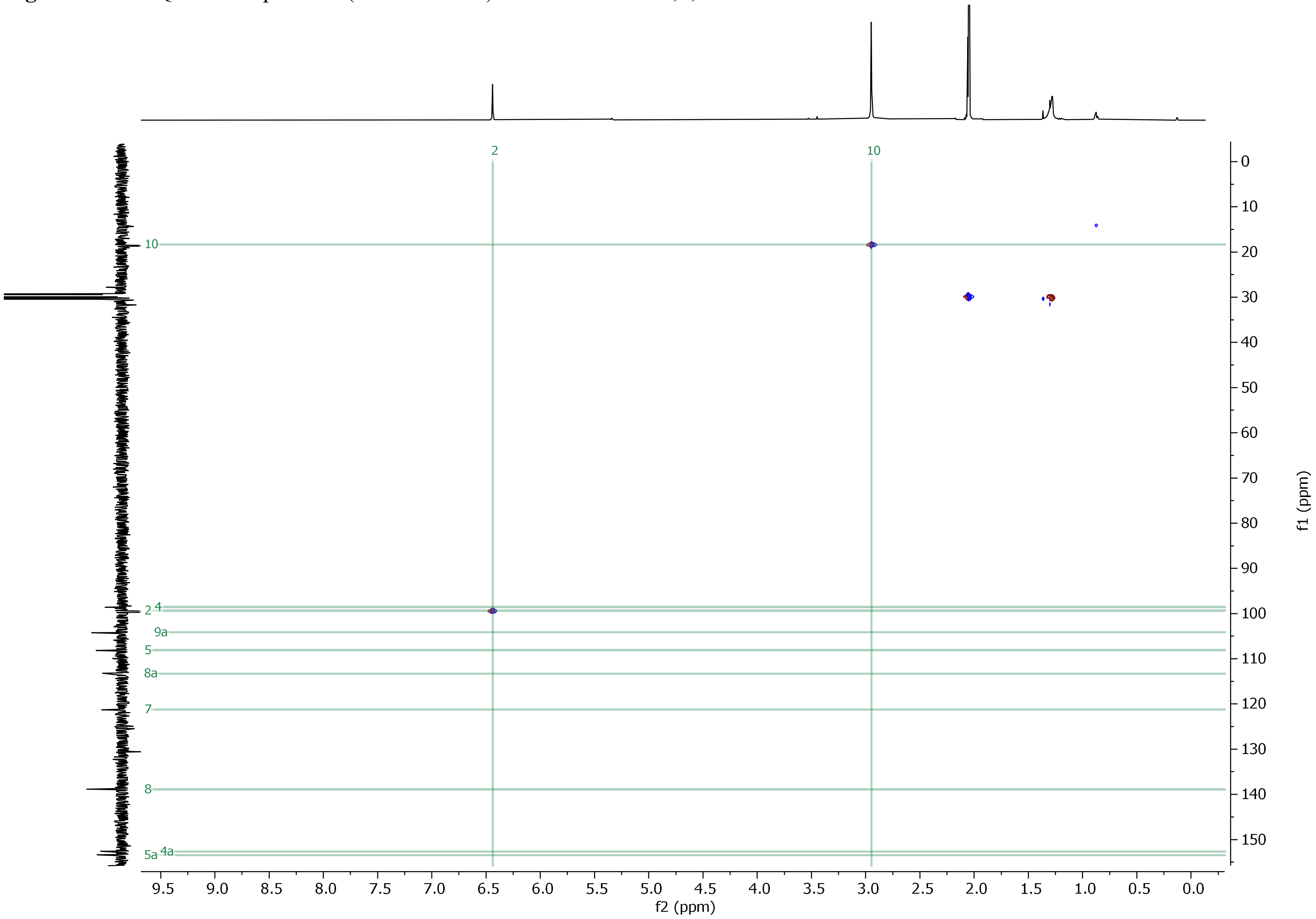

Figure S71. HMBC NMR spectrum (500/125 MHz) in acetone- d_6 of 4,5,7-trichloronorlichexanthone **15**

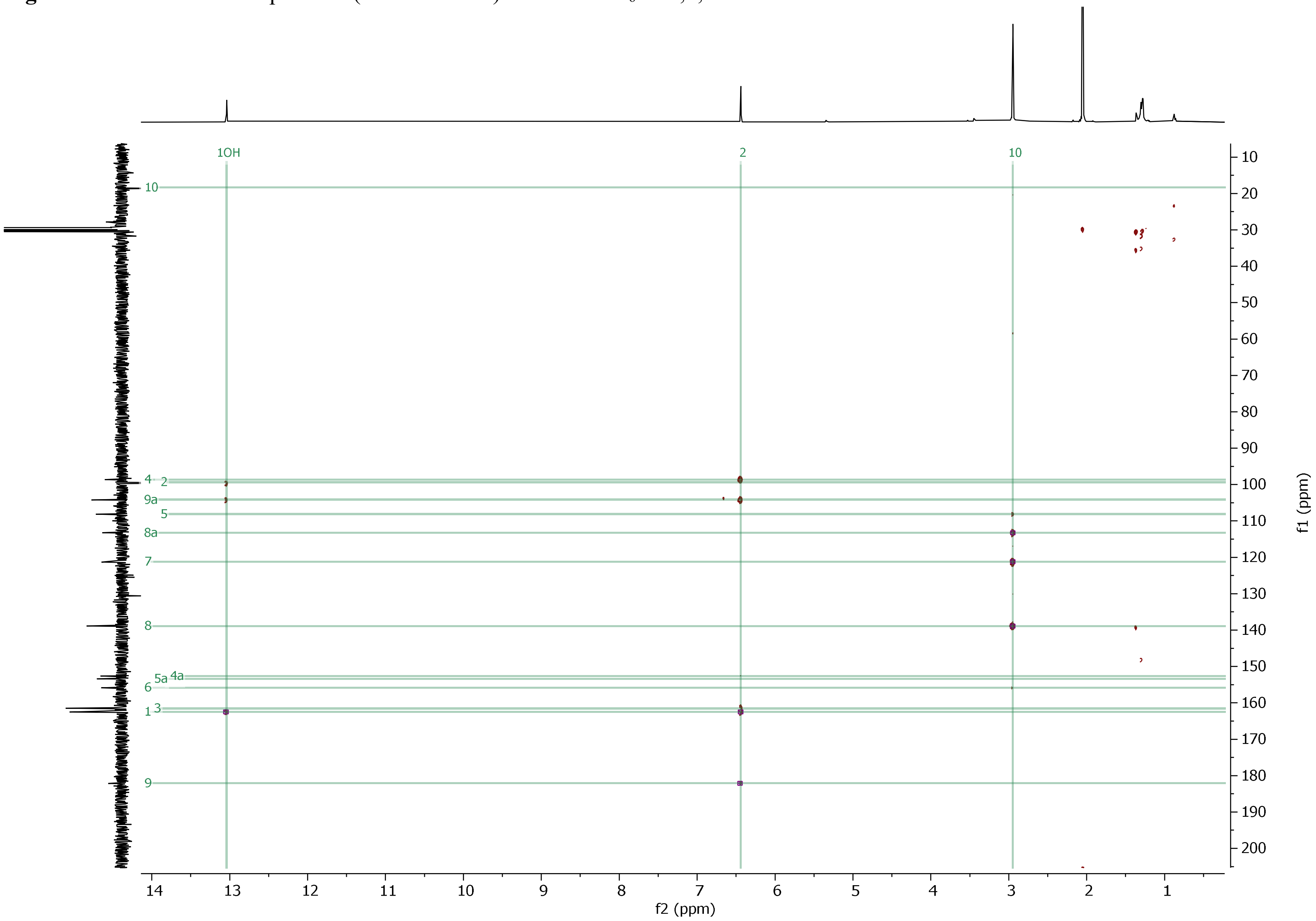

Figure S72. NOESY NMR spectrum (500 MHz) in acetone- d_6 of 4,5,7-trichloronorlichexanthone **15**

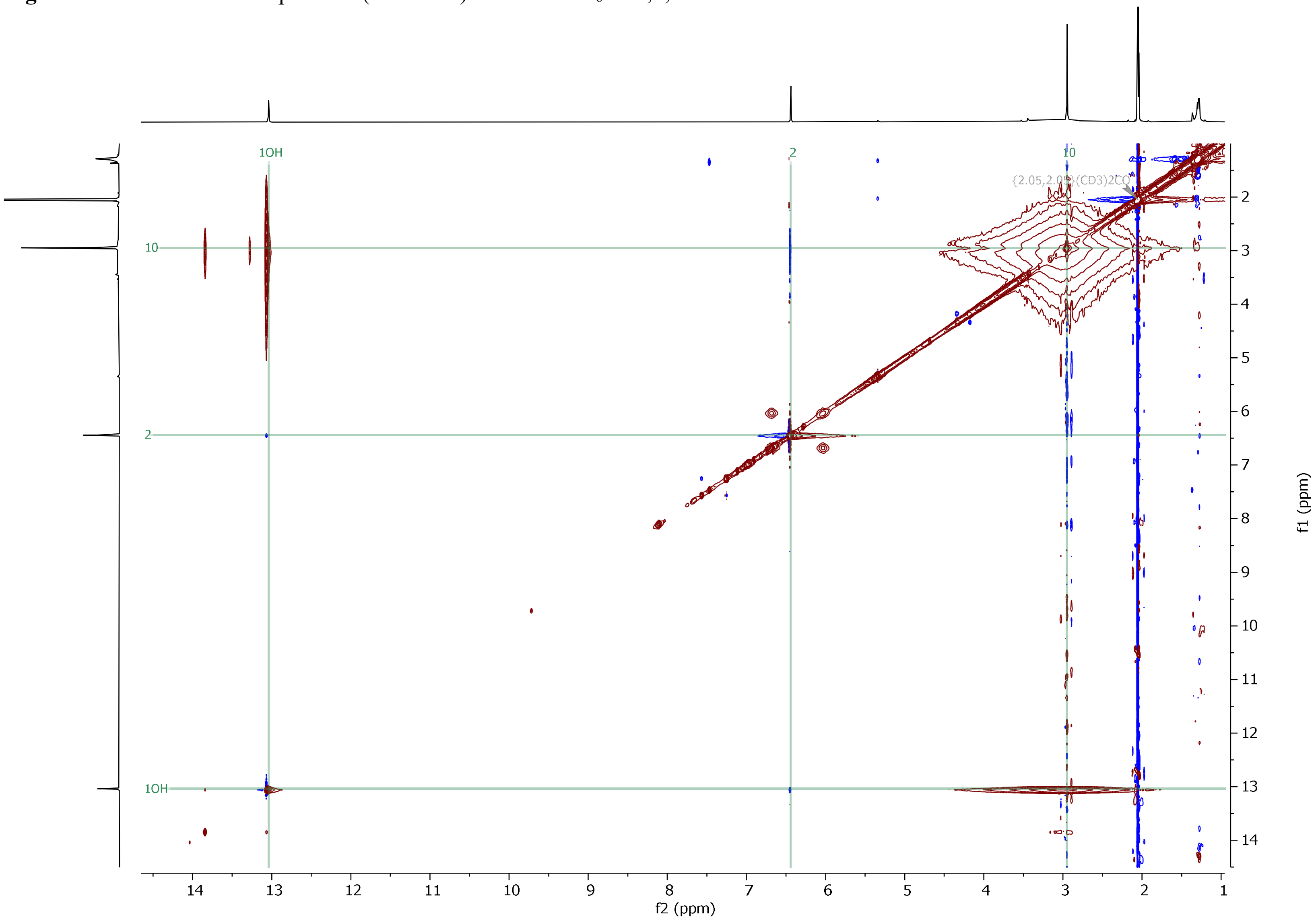

Figure S73. ^{1}H NMR spectrum (500 MHz) in DMSO- d_6 of 2,4,5,7-tetrachloronorlichexanthone **16**

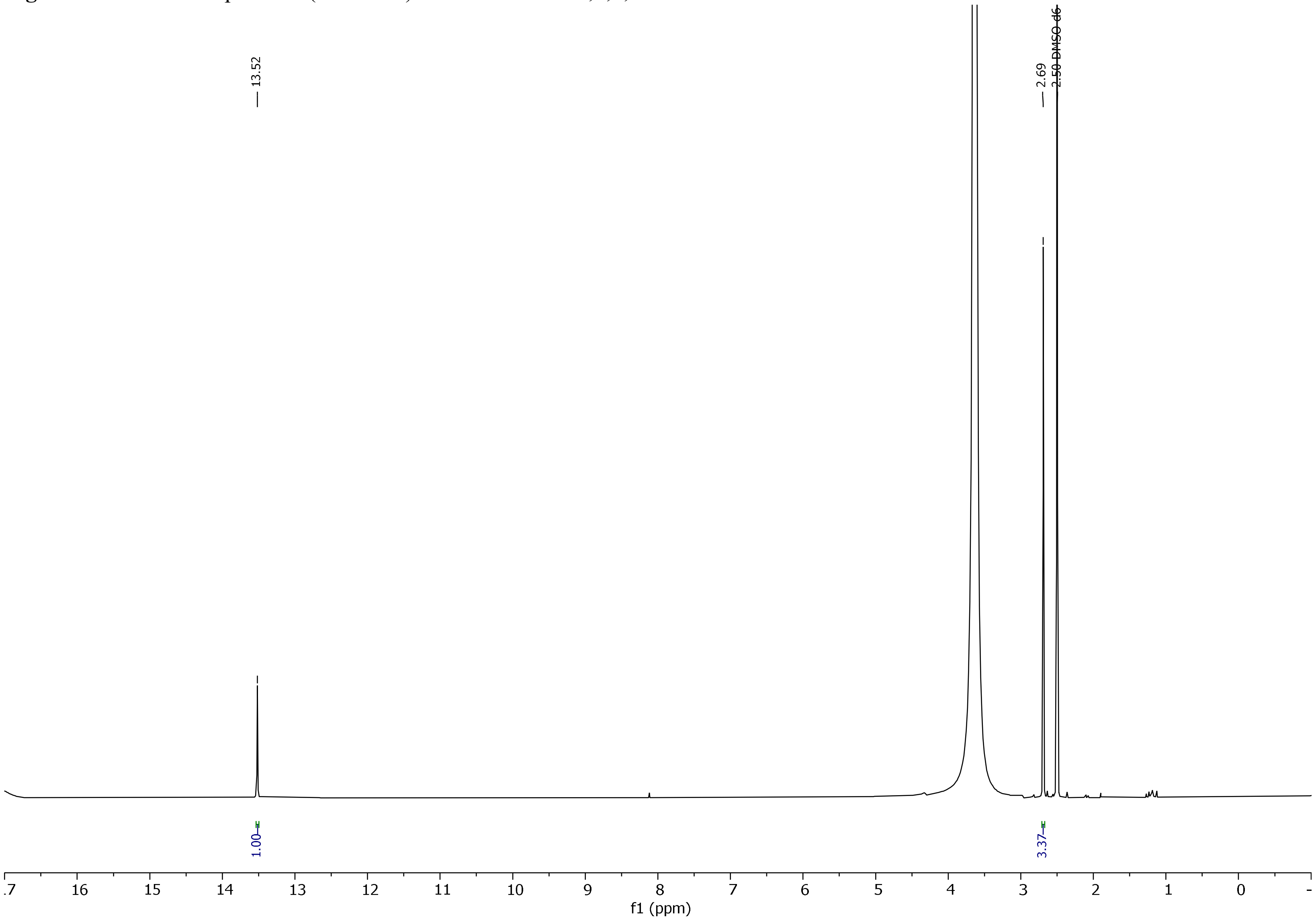

Figure S74. Jmod NMR spectrum (125 MHz) in DMSO - d_6 of 2,4,5,7-tetrachloronorlichexanthone **16**

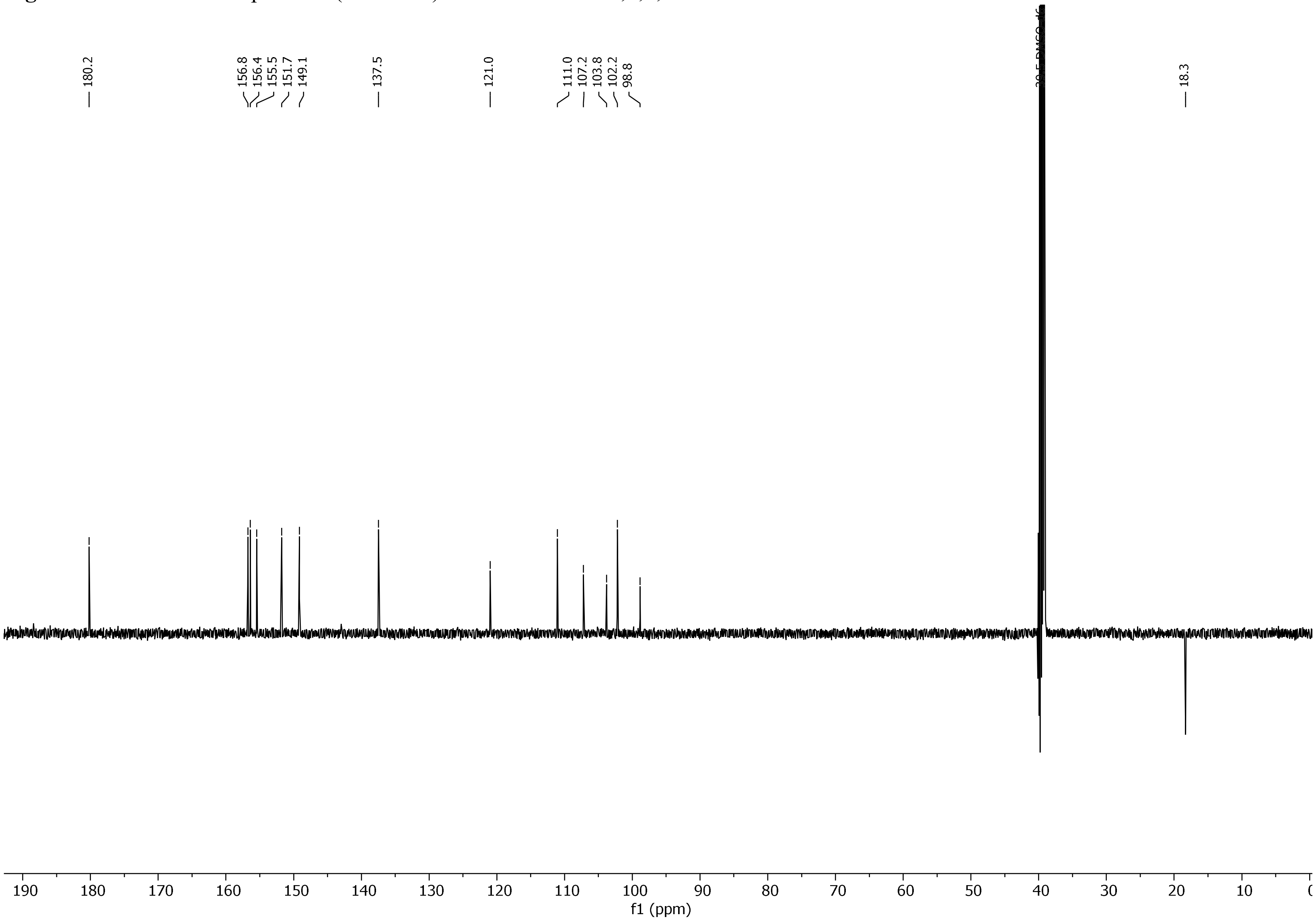

Figure S75. HSQC NMR spectrum (500/125 MHz) in DMSO - d_6 of 2,4,5,7-tetrachloronorlichexanthone **16**

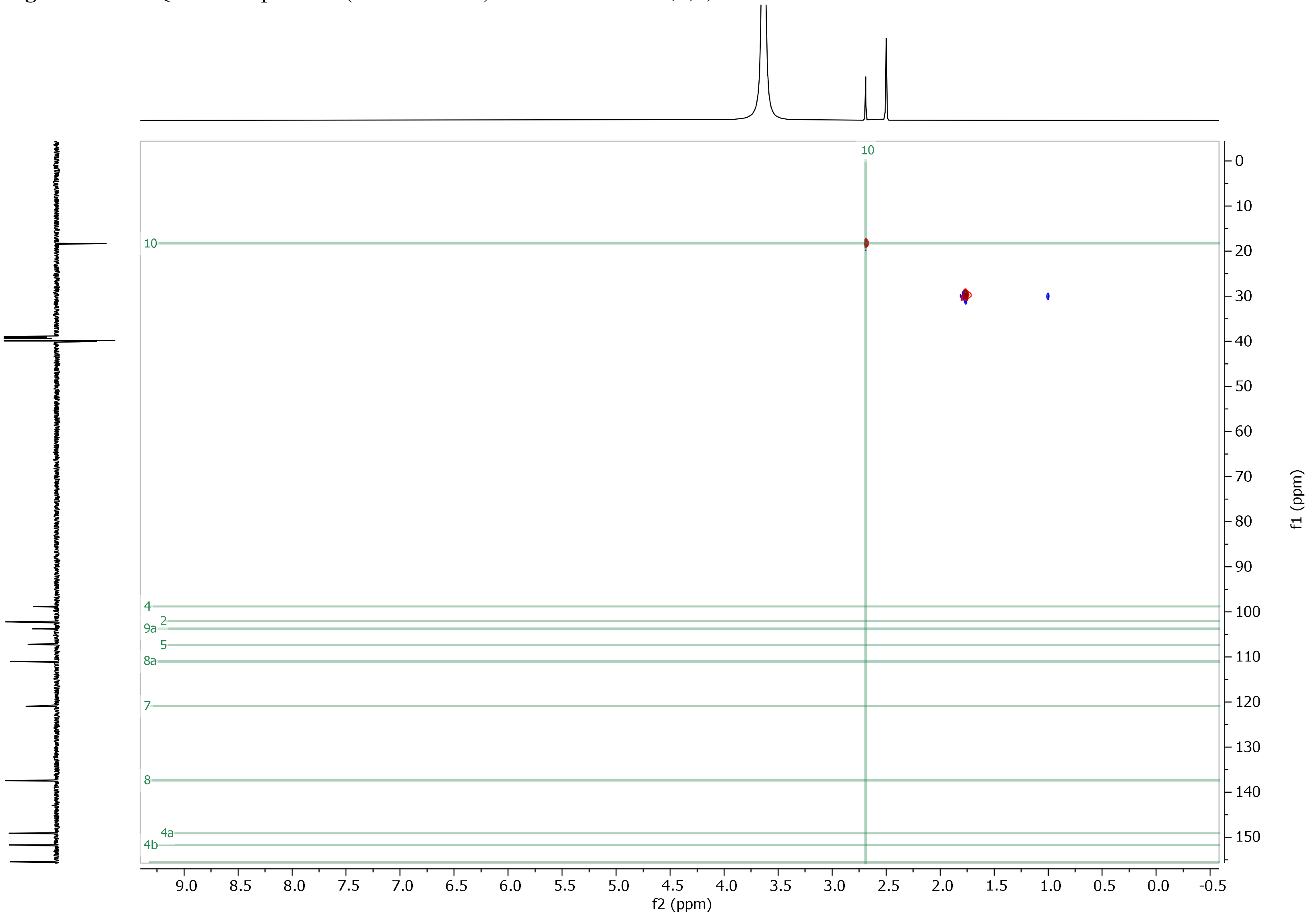

Figure S76. HMBC NMR spectrum (500/125 MHz) in DMSO - d_6 of 2,4,5,7-tetrachloronorlichexanthone **16**

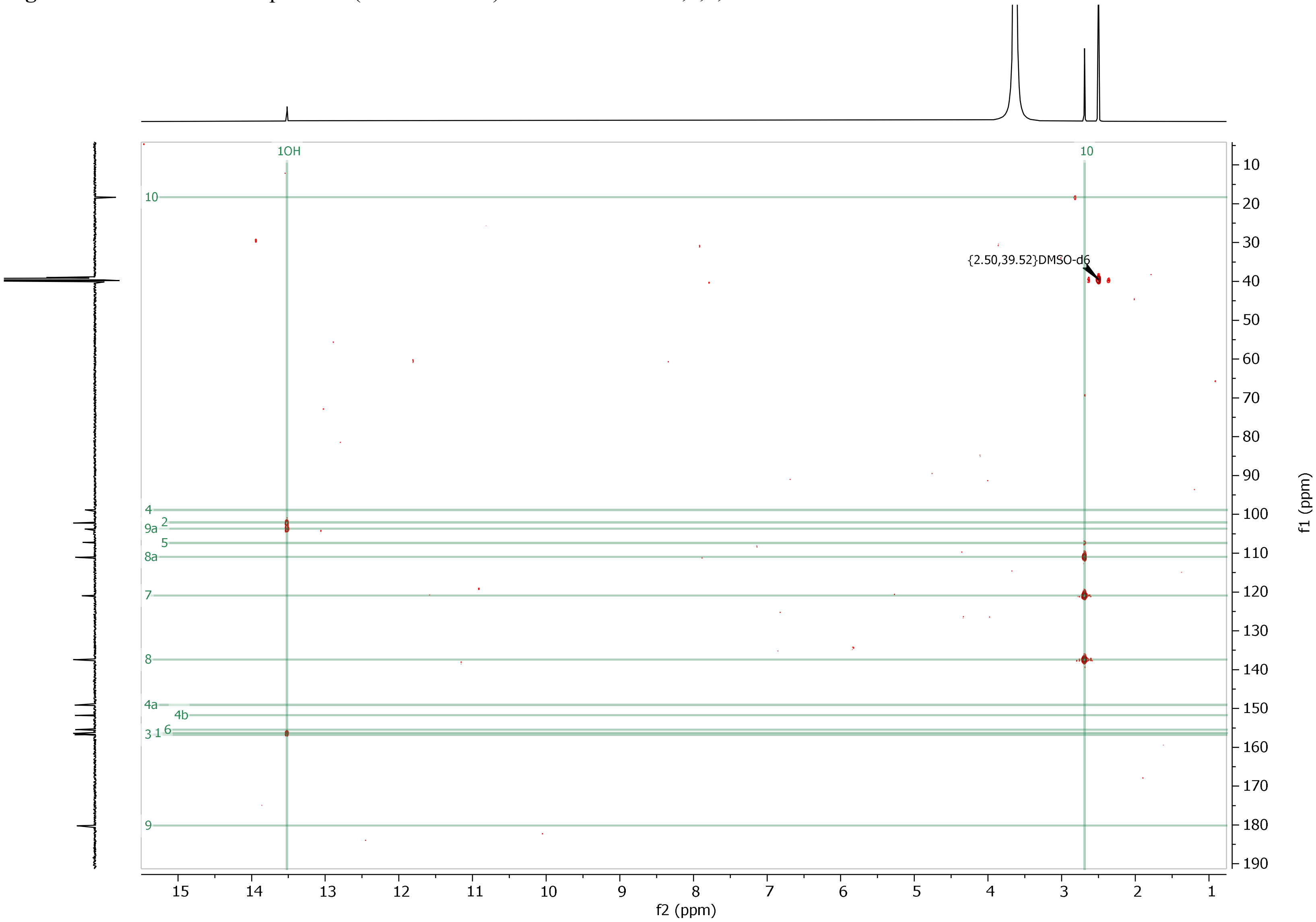

Figure S77. ^{1}H NMR spectrum (500 MHz) in acetone- d_6 of 5-chloroorsellinic acid **17**

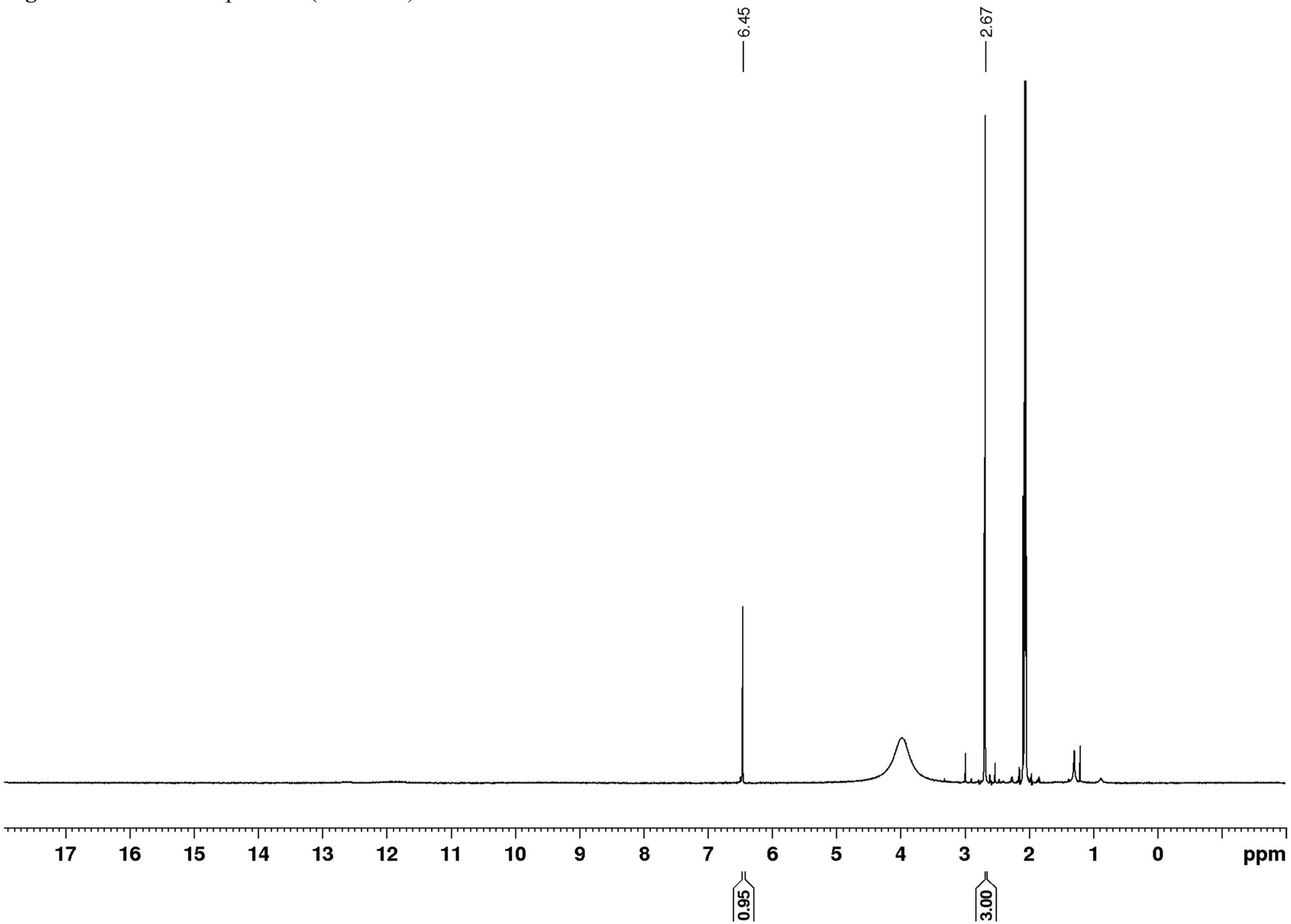

Figure S78. ^{1}H NMR spectrum (500 MHz) in acetone- d_6 of 3-chloroorsellinic acid **18**

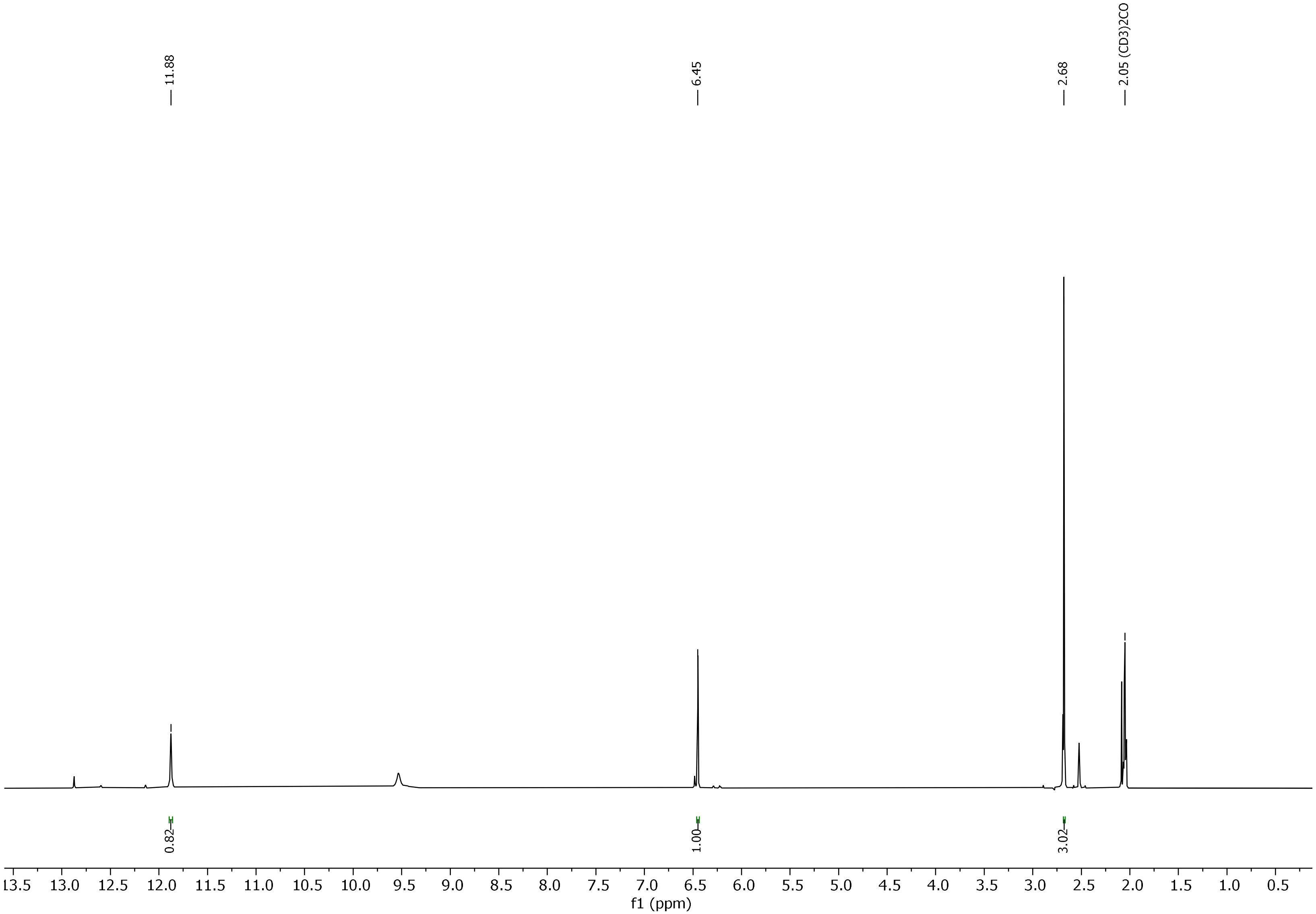

Figure S79. ^{1}H NMR spectrum (500 MHz) in acetone- d_6 of 3,5-dichloroorsellinic acid **19**

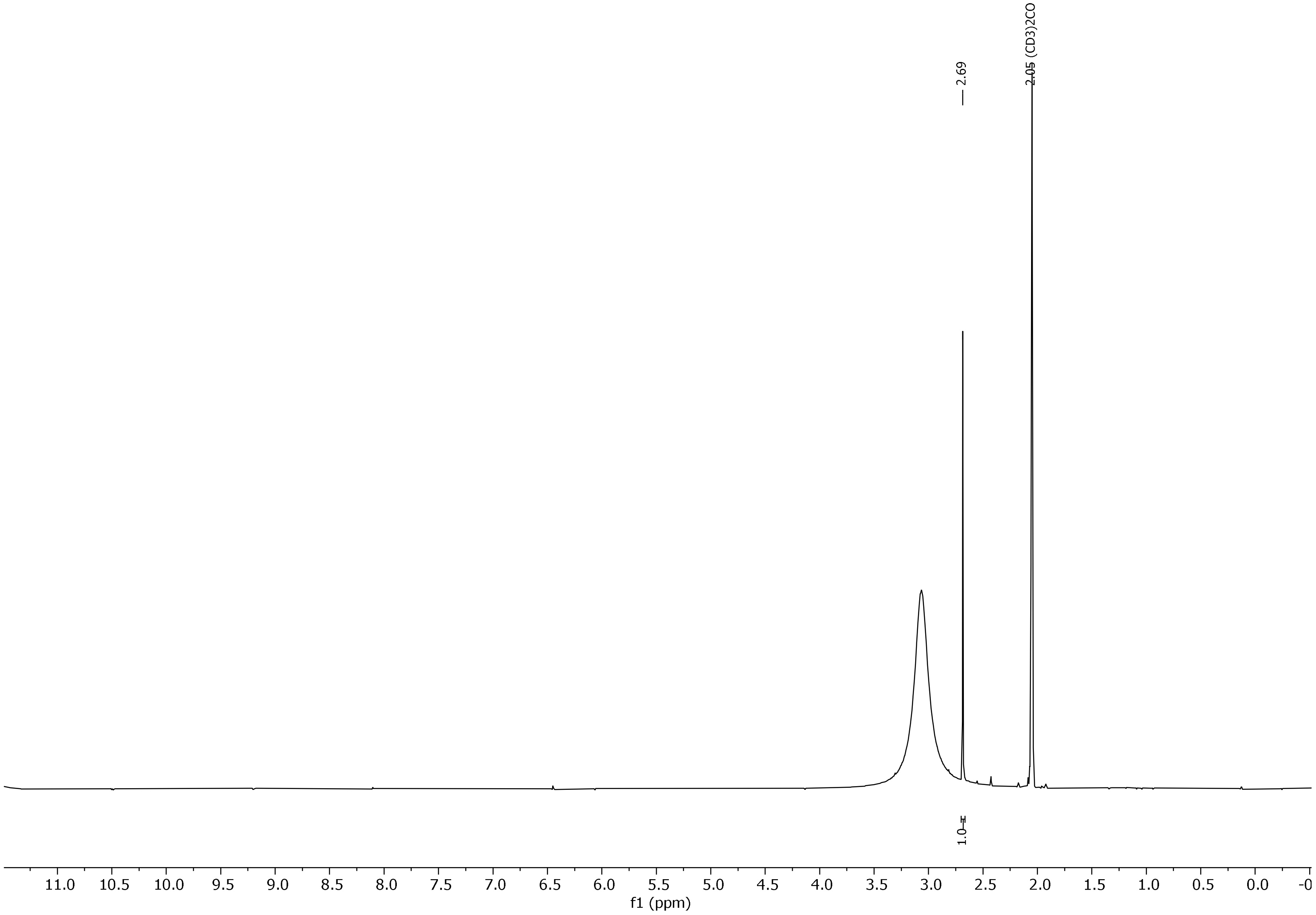

Figure S80. ^{1}H NMR spectrum (500 MHz) in acetone- d_6 of chlorophloroglucinol **20**

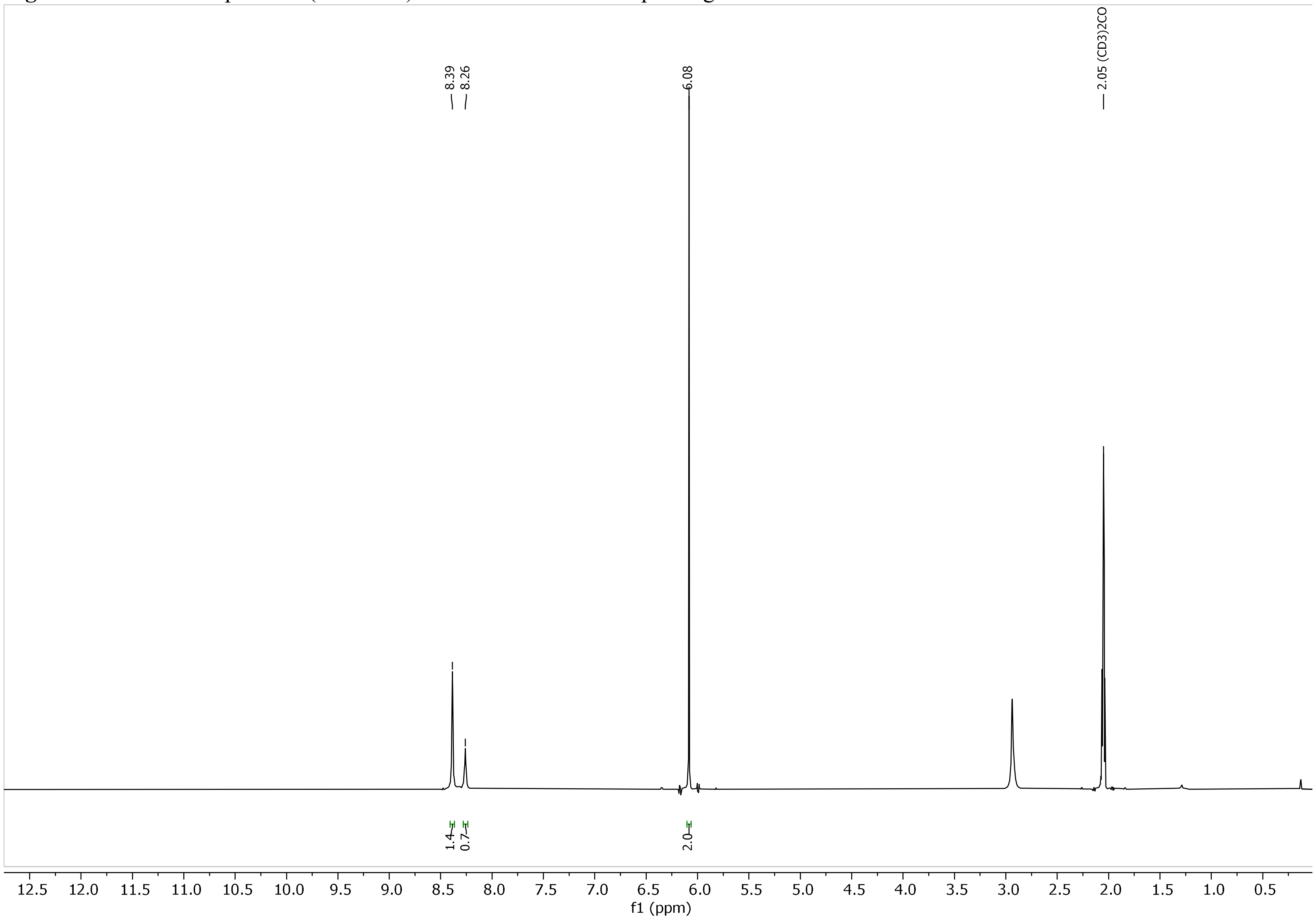

2. NMR analysis of lichens extracts

Figure S81. ^{1}H NMR spectrum (500 MHz) in acetone- d_6 of *Lecanora alboflavida* extract

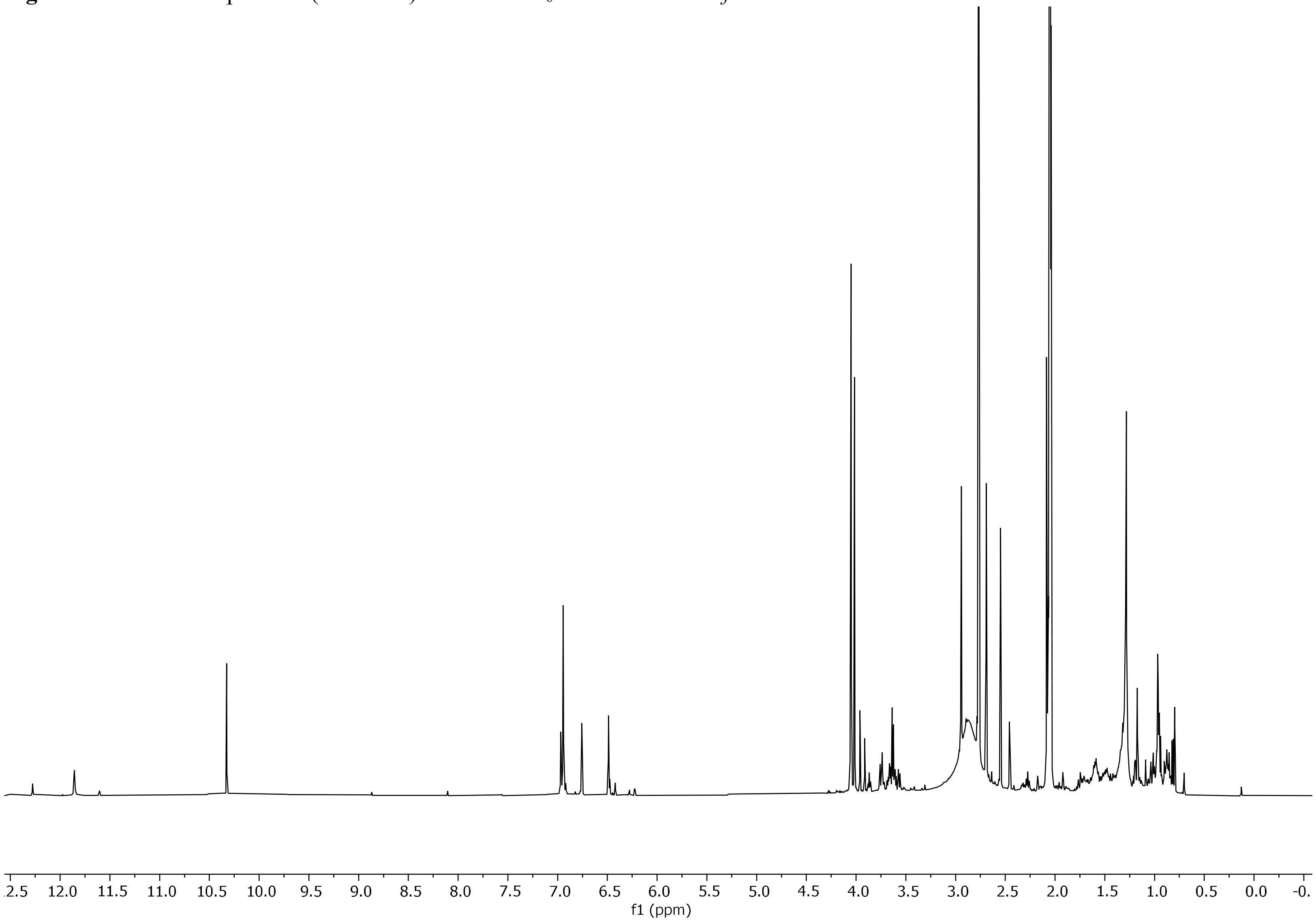

Figure S82. ^{13}C NMR spectrum (125 MHz) in acetone-d_6 of *Lecanora alboflavida* extract

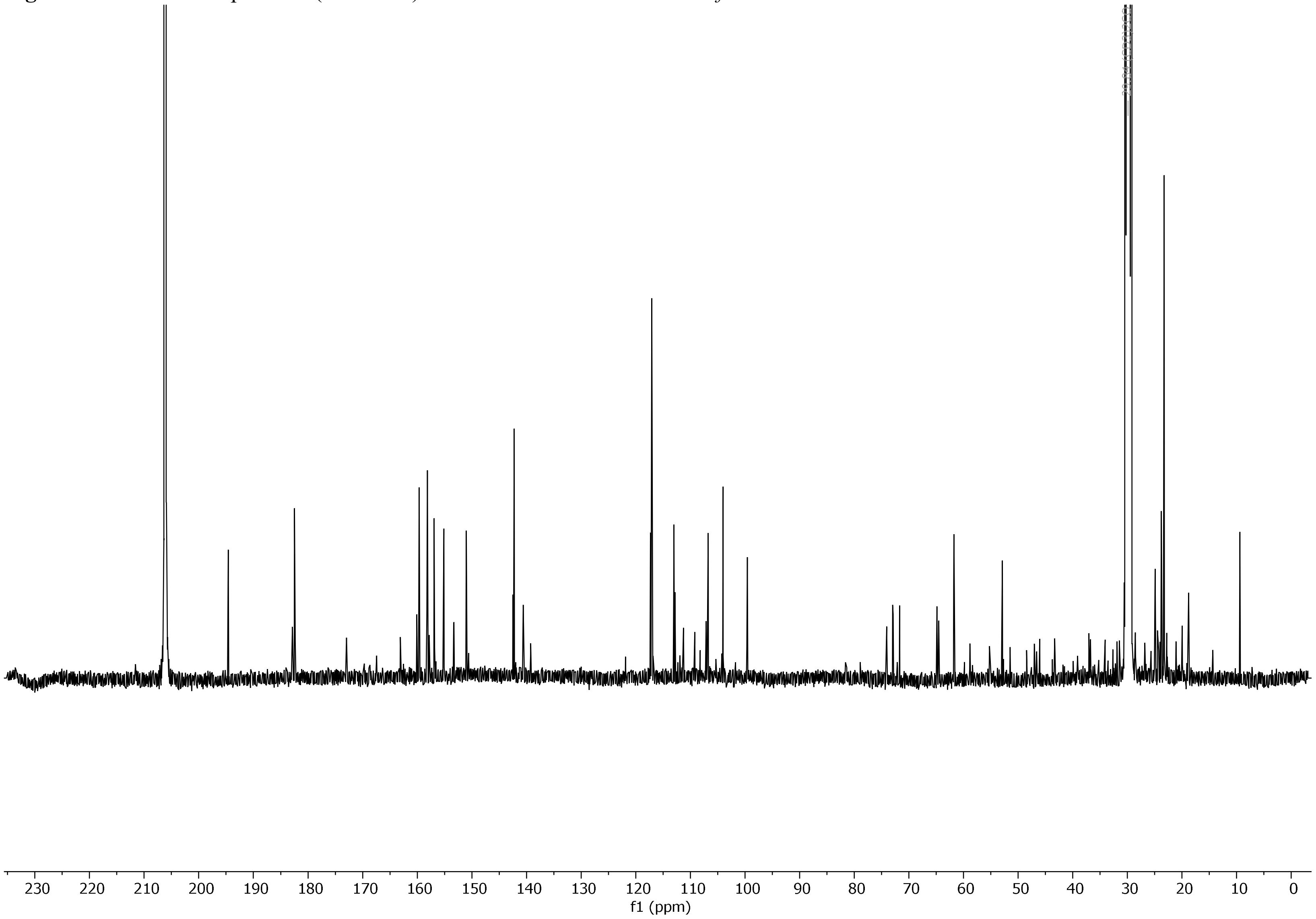

Figure S83. HSQC NMR spectrum (500/125 MHz) in acetone- d_6 of *Lecanora alboflavida* extract

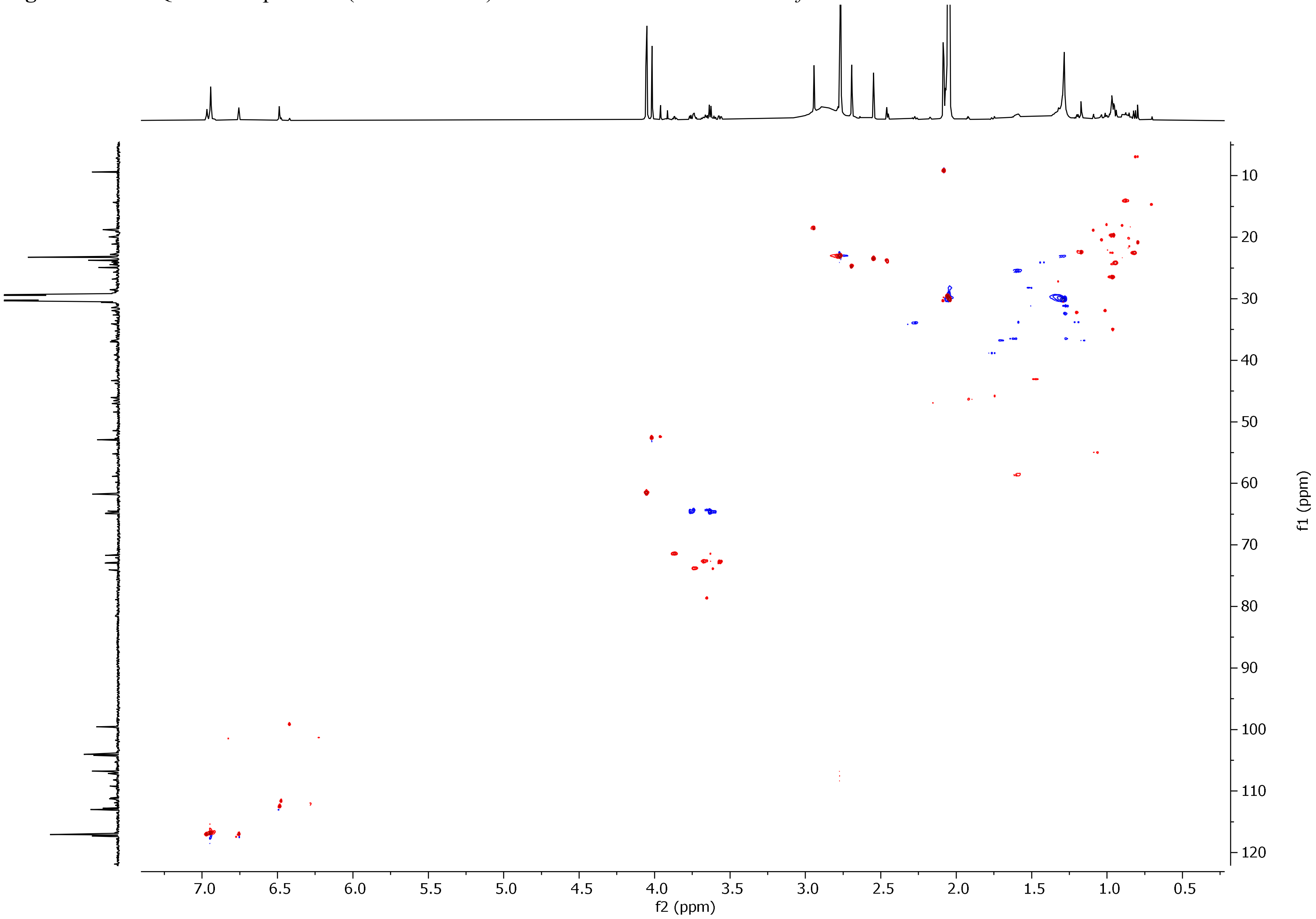

Figure S84. HSQC spectrum of *L. alboflavida* extract (^{1}H between 6 and 7.2 ppm; ^{13}C between 90 and 120 ppm).

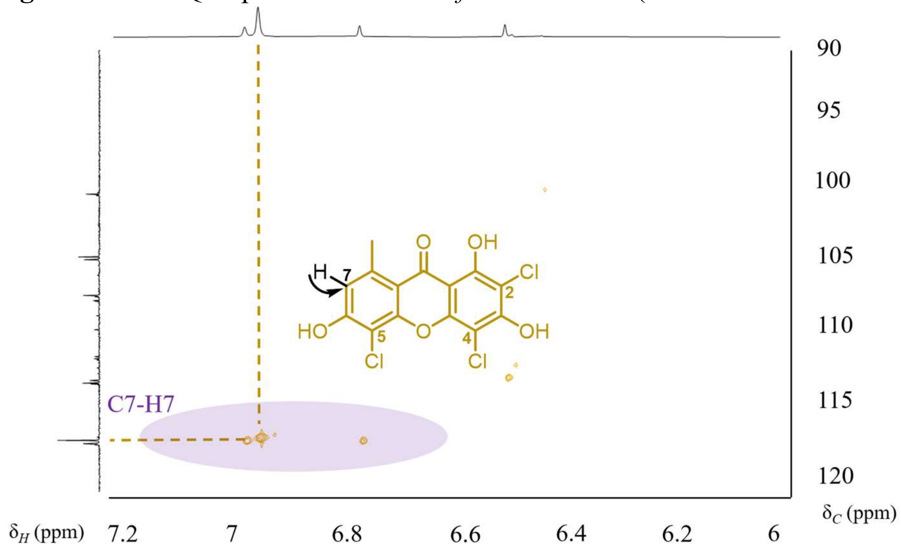

Figure S85. NOESY NMR spectrum (500 MHz) in acetone- d_6 of *Lecanora alboflavida* extract

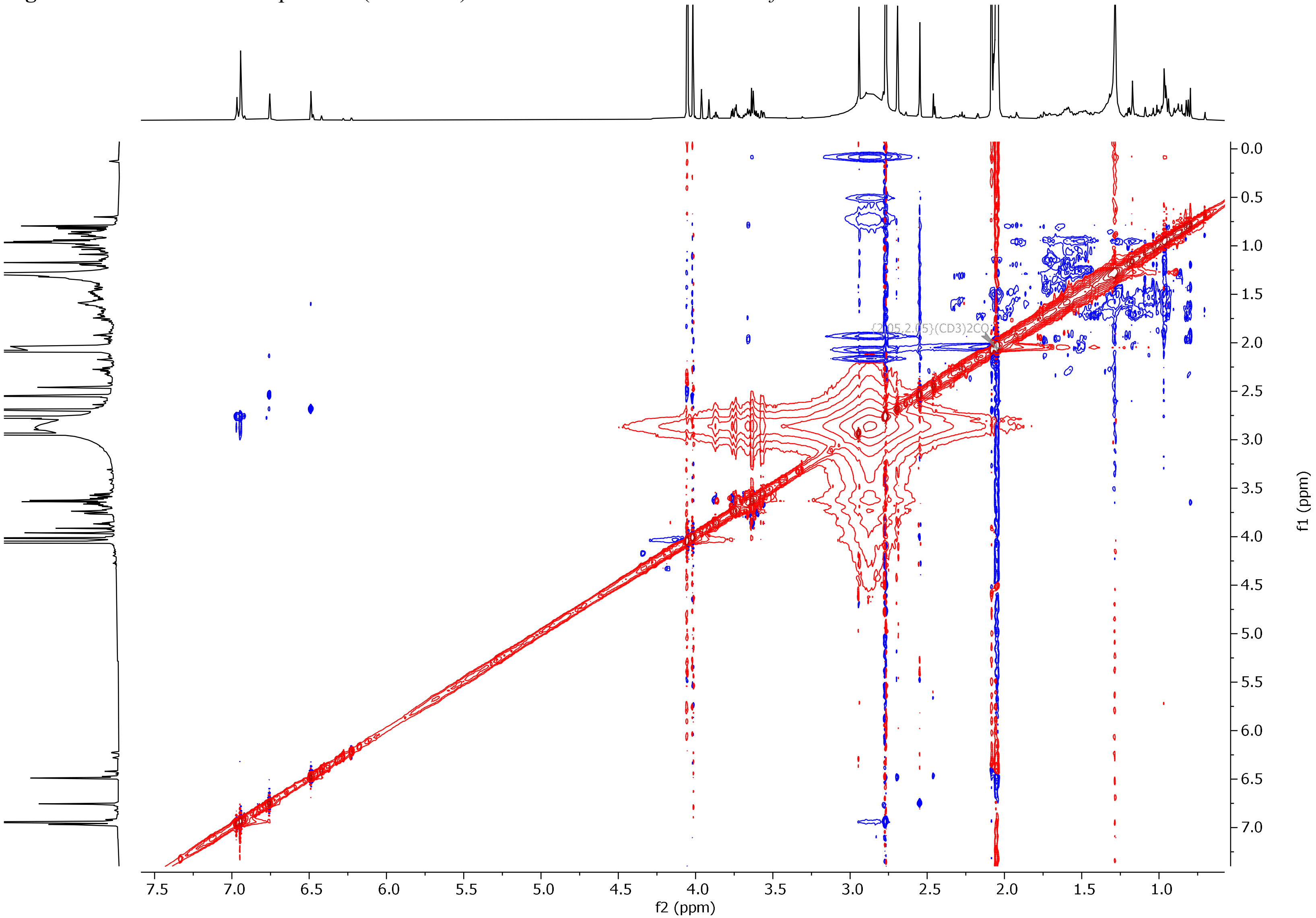

Figure S86. ^{1}H NMR spectrum (500 MHz) in acetone- d_6 of *Myriolecis antiqua* extract

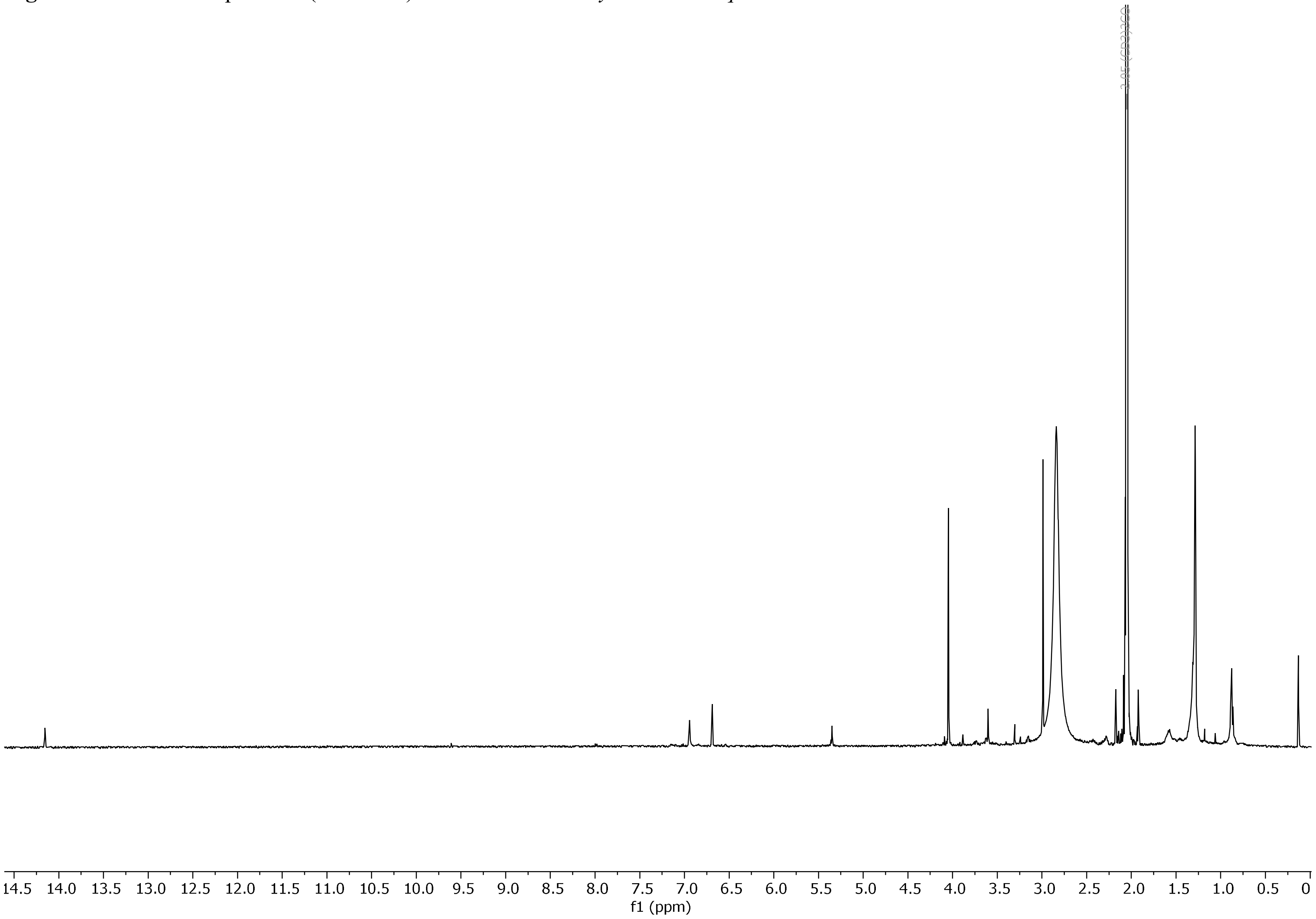

Figure S87. HSQC NMR spectrum (500/125 MHz) in acetone- d_6 of *Myriolecis antiqua* extract

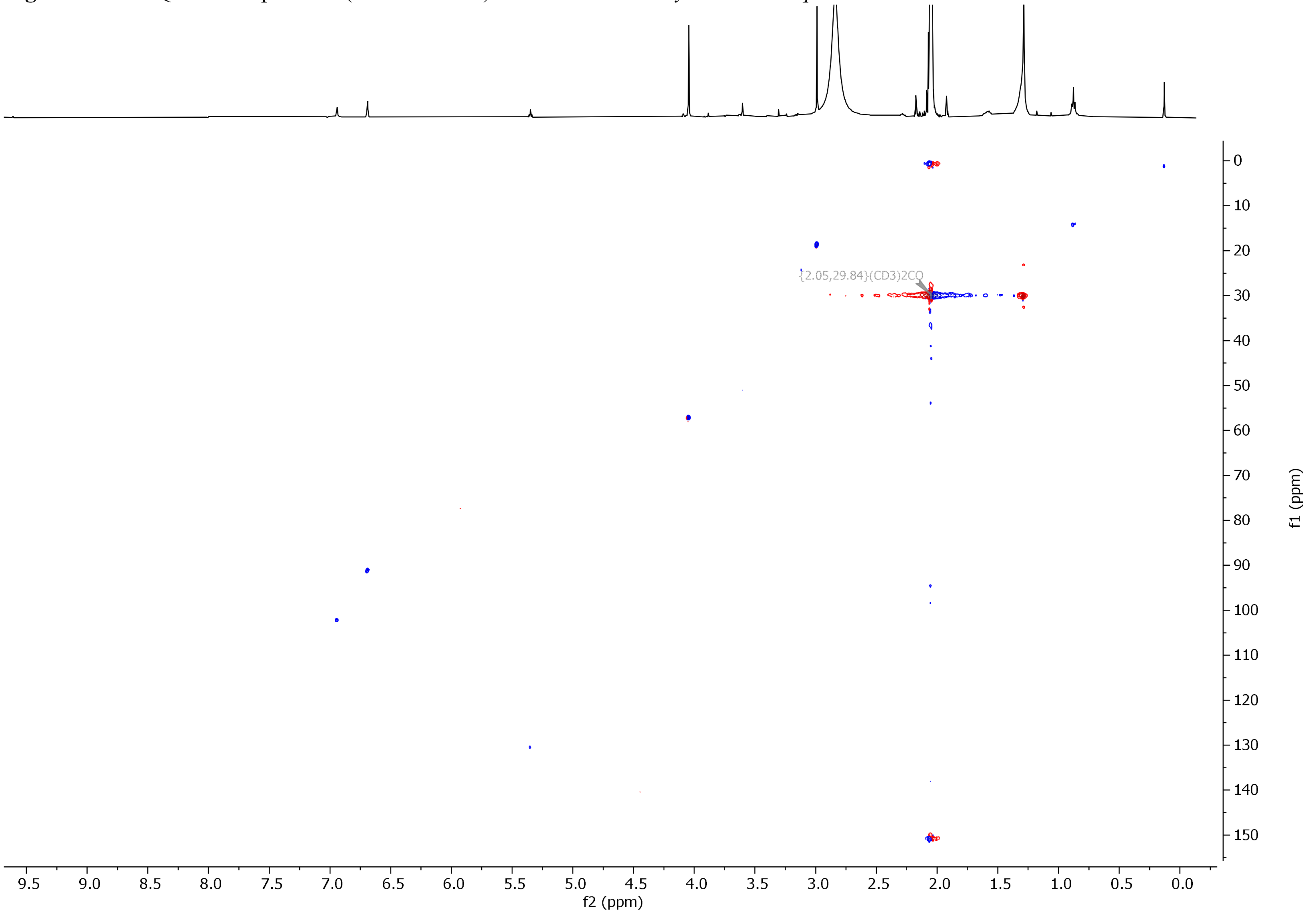

Figure S88. NOESY NMR spectrum (500 MHz) in acetone- d_6 of *Myriolecis antiqua* extract

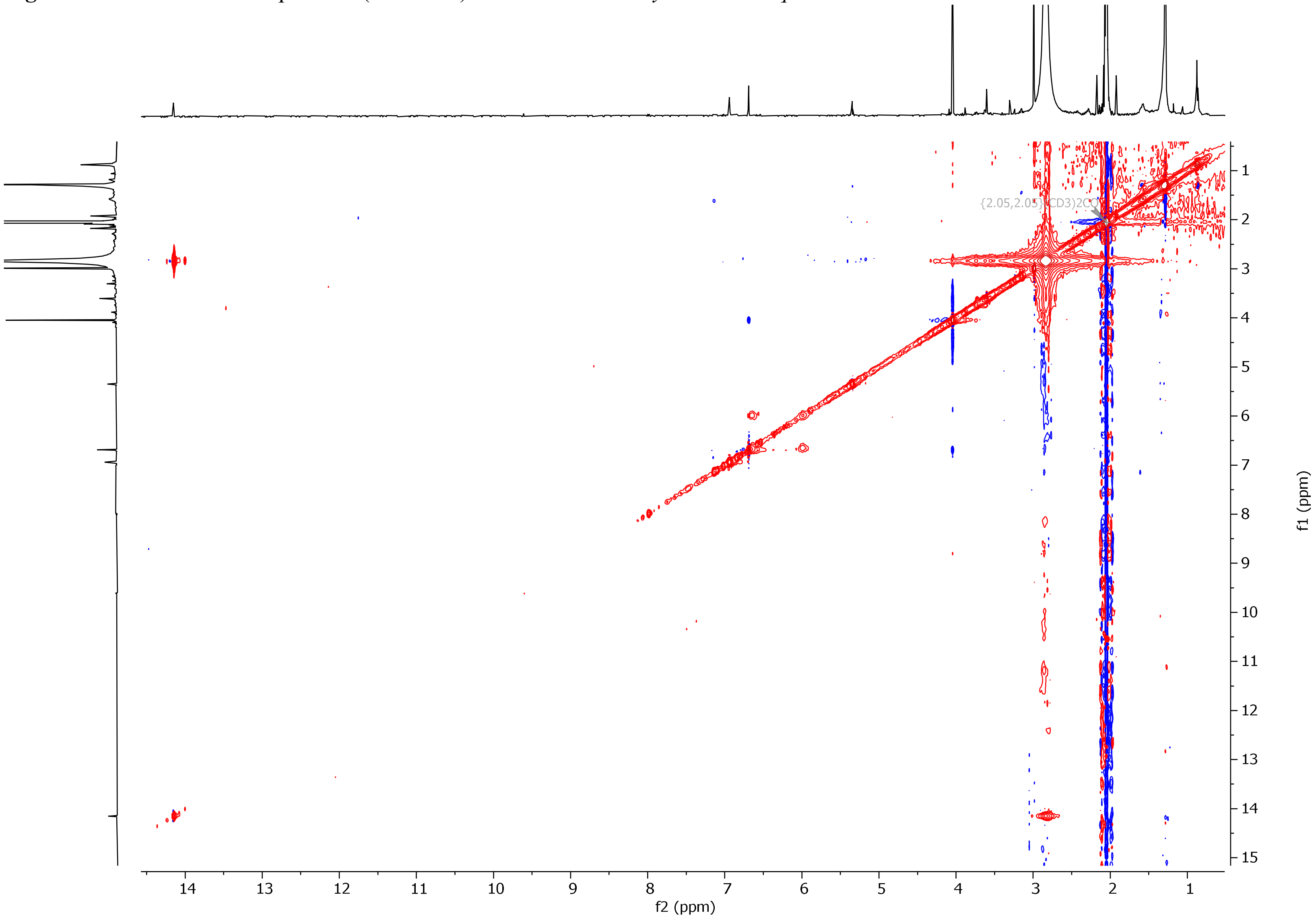

Figure S89. ^{1}H NMR spectrum (500 MHz) in acetone- d_6 of *Pyrrhospora quernea* extract

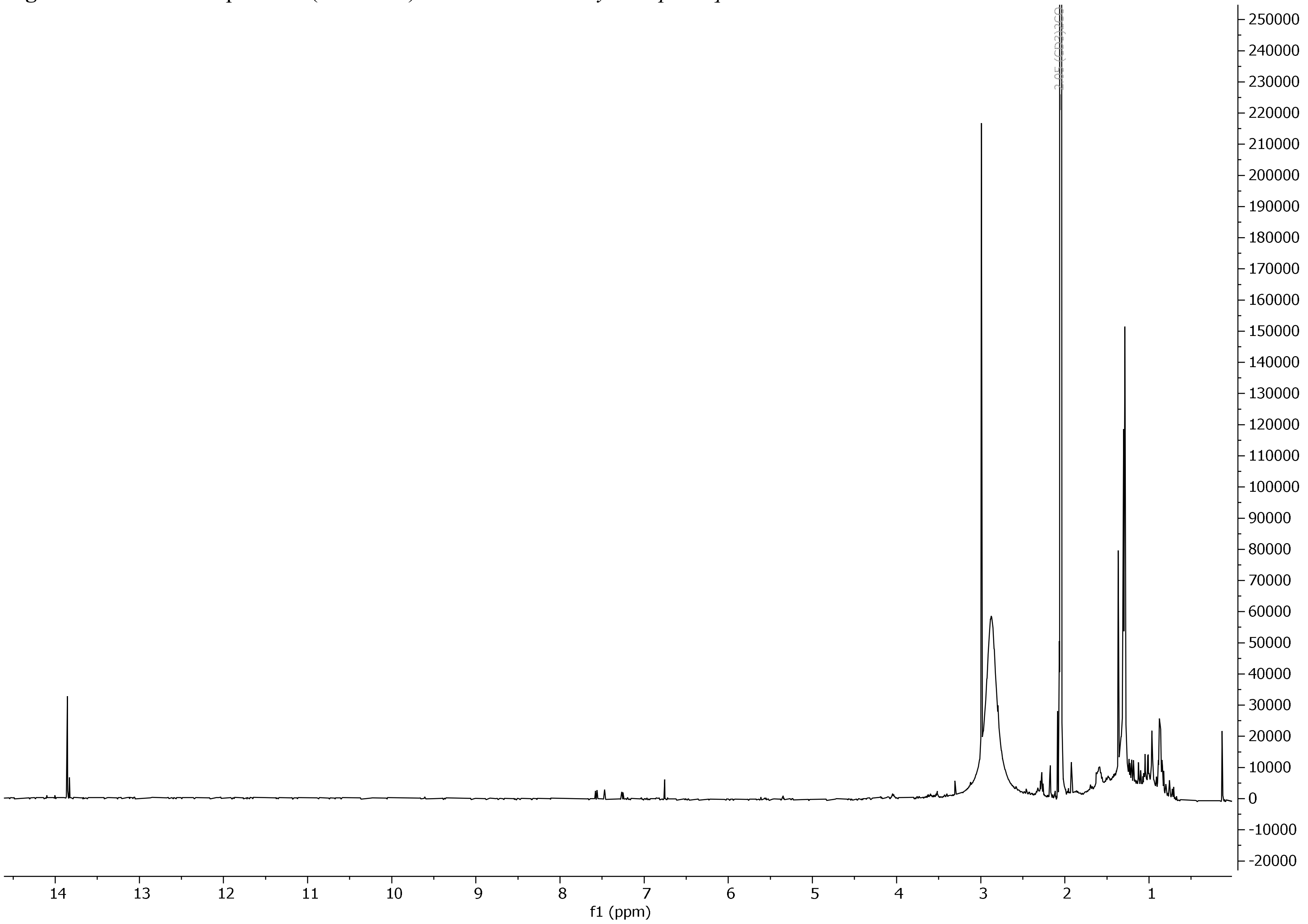

Figure S90. HSQC NMR spectrum (500/125 MHz) in acetone- d_6 of *Pyrrhospora quernea* extract

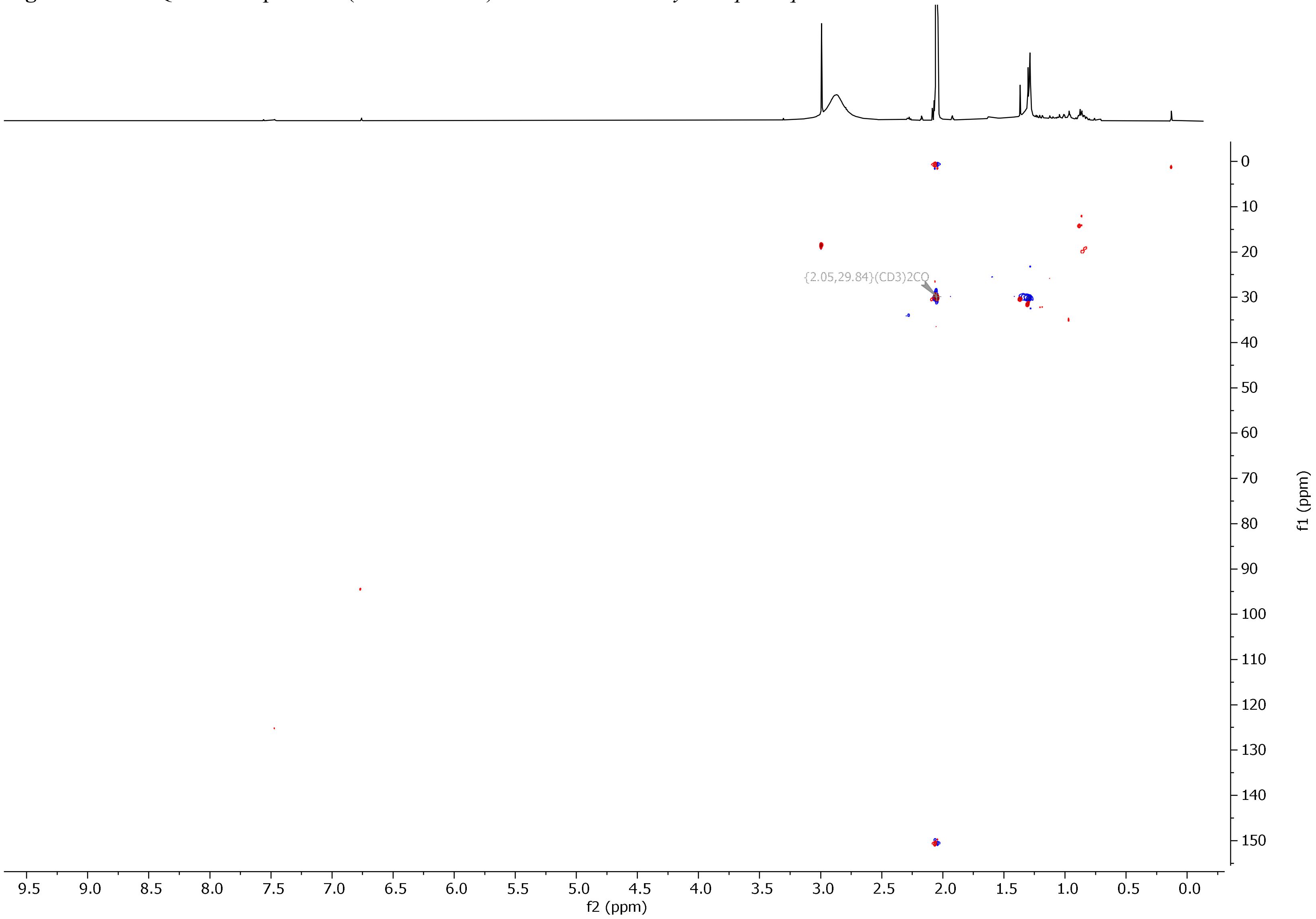

Figure S91. NOESY NMR spectrum (500 MHz) in acetone- d_6 of *Pyrrhospora quernea* extract

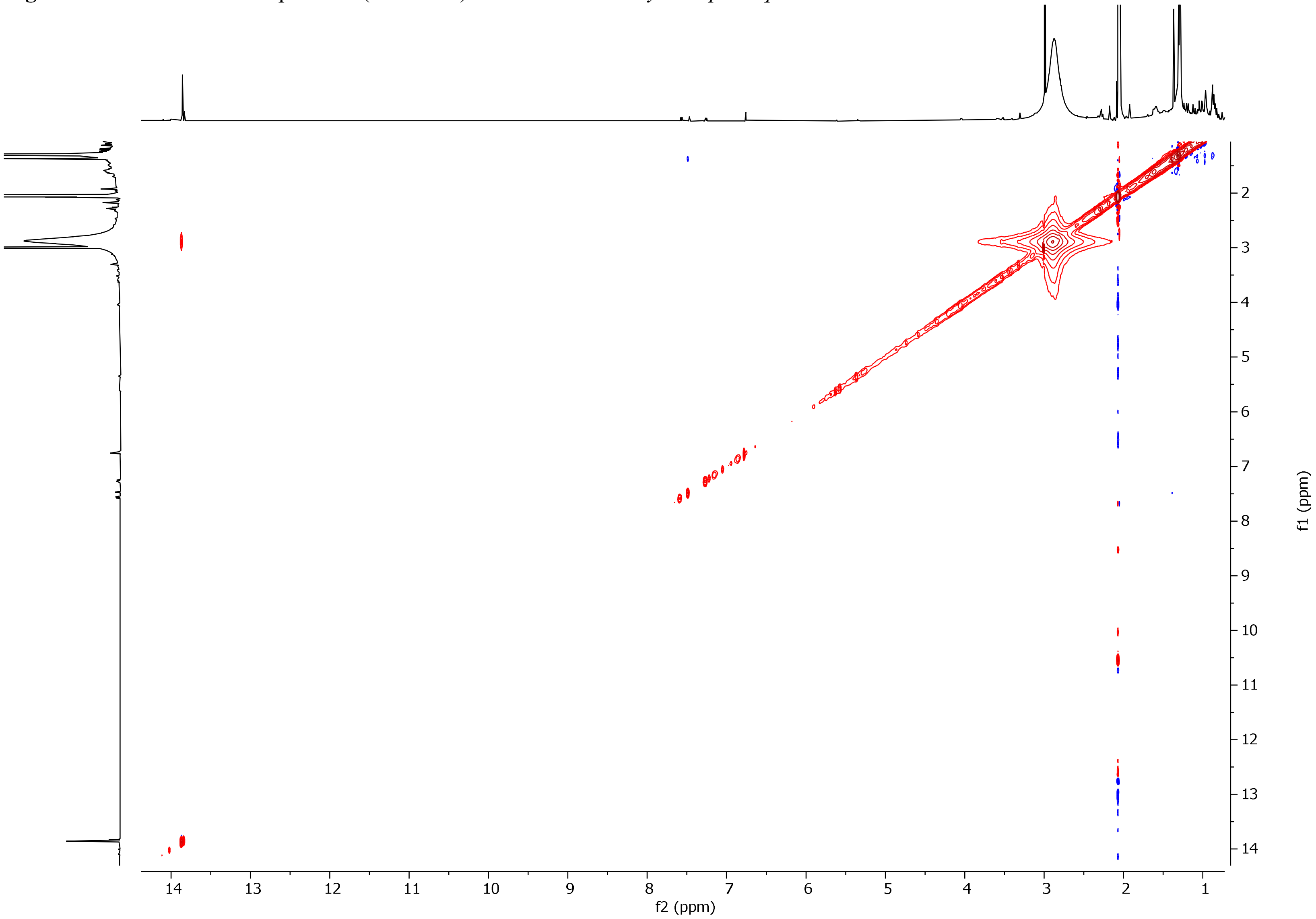

Figure S92. ^{1}H NMR spectrum (500 MHz) in acetone- d_6 of *Lecidella elaeochroma* extract

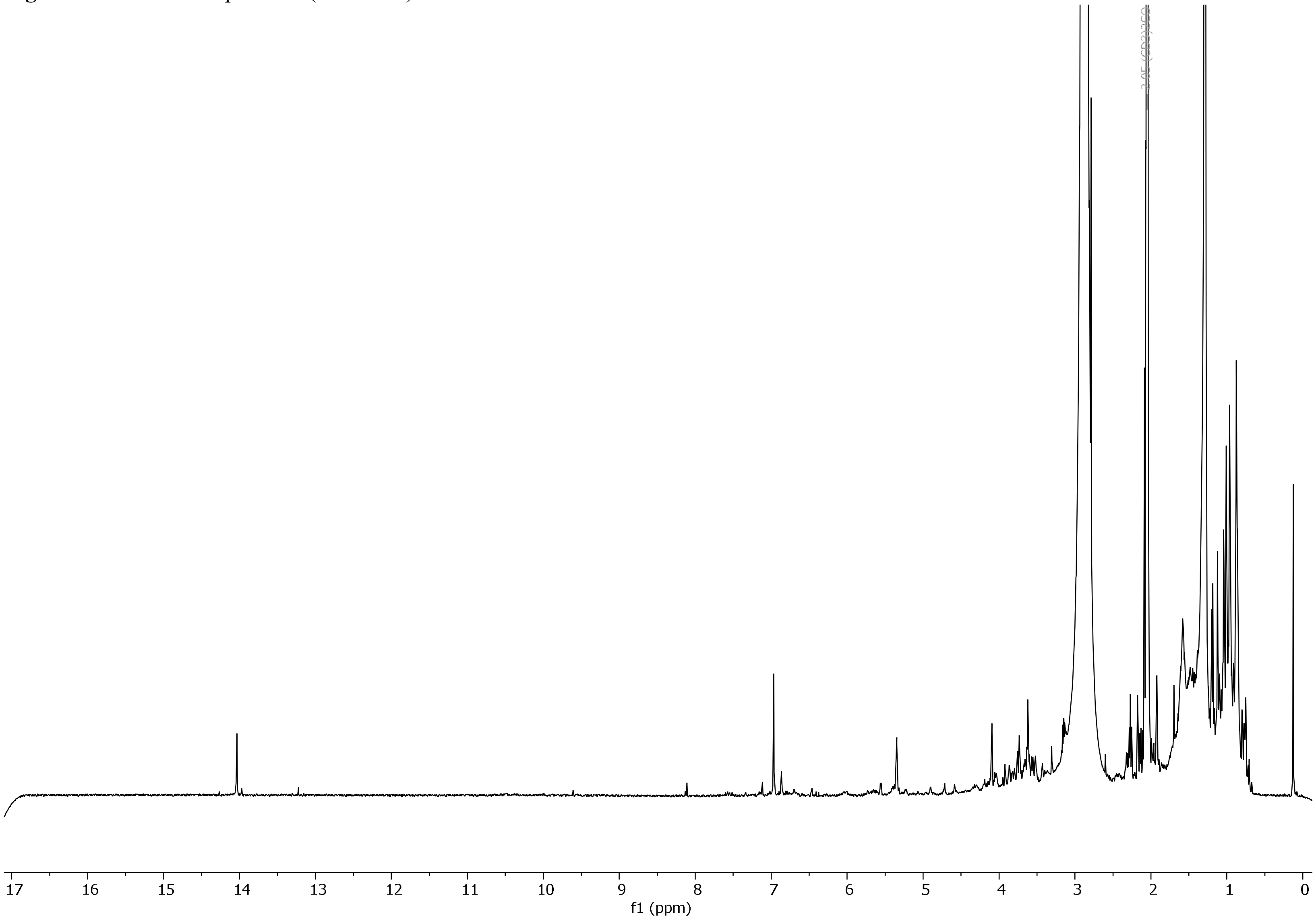

Figure S93. HSQC NMR spectrum (500/125 MHz) in acetone- d_6 of *Lecidella elaeochroma* extract

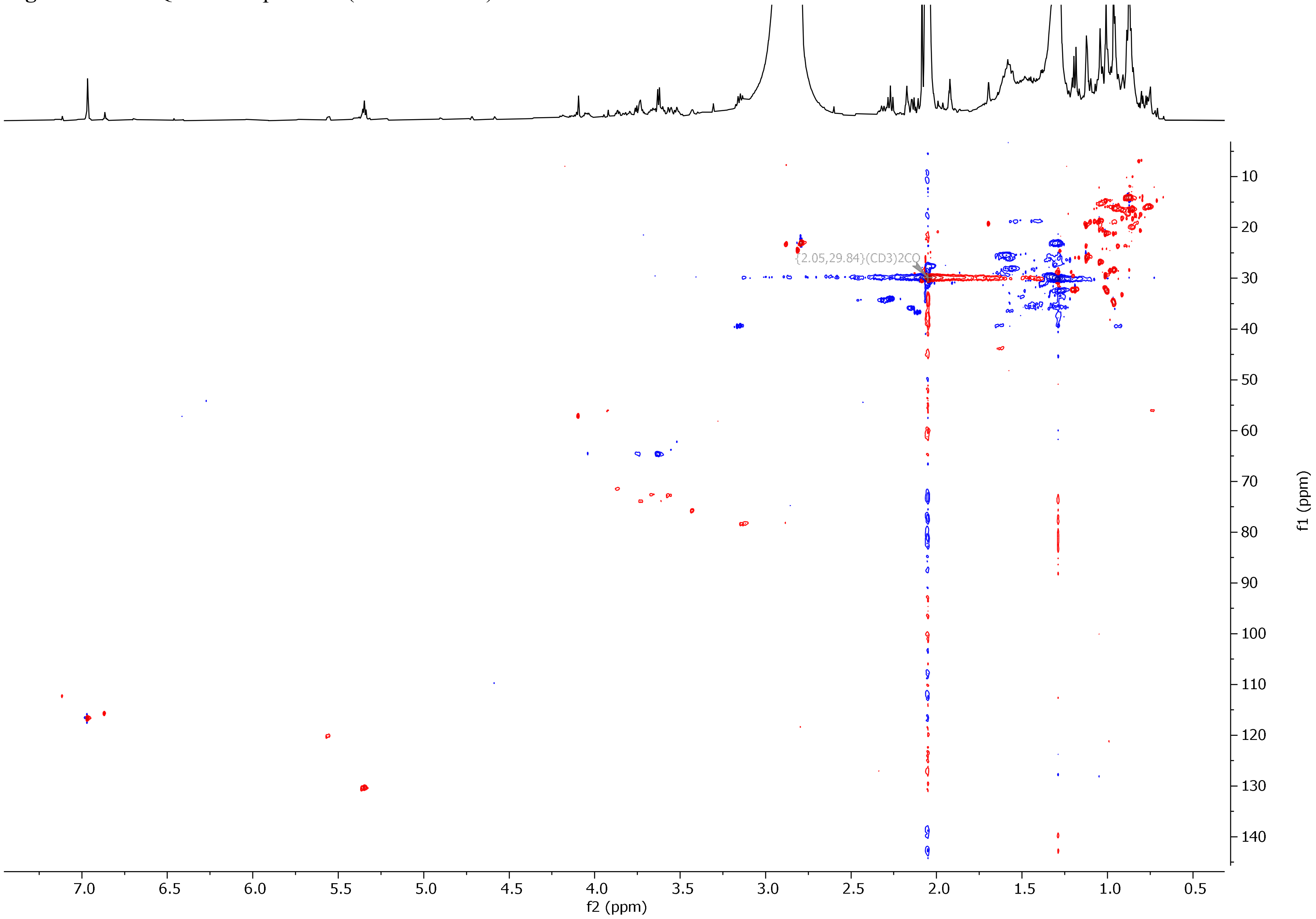

Figure S94. NOESY NMR spectrum (500 MHz) in acetone- d_6 of *Lecidella elaeochroma* extract

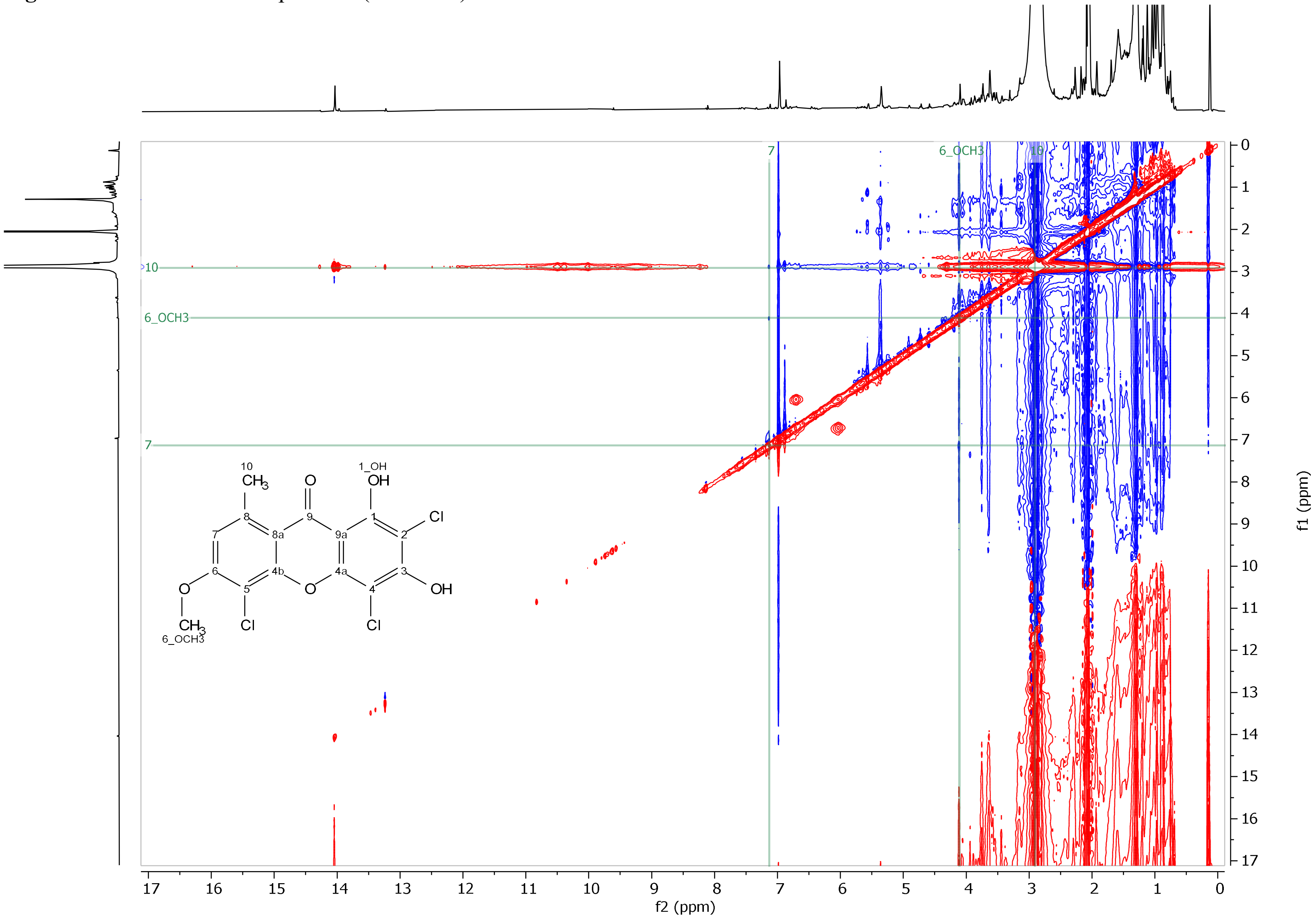

Figure S95. Key NOESY correlation discriminating 3L245 from 6L245

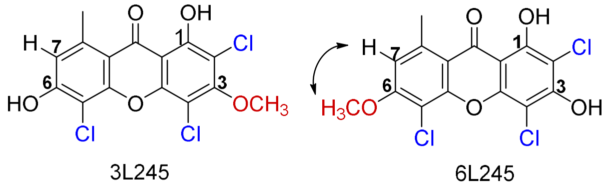

Figure S96. HMBC correlations (H→ C) that distinguish between 3L2457 and 6L2457

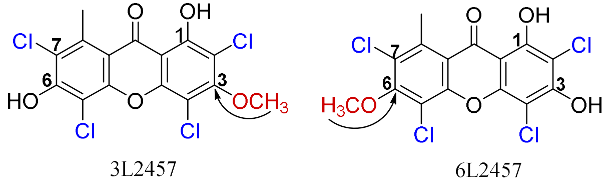

3. Chromatographic data of compounds **1-16**

Figure S97. HPLC/DAD chromatogram and extracted MS spectrum of norlichexanthone **1** (*Rt*= 6.6 min)

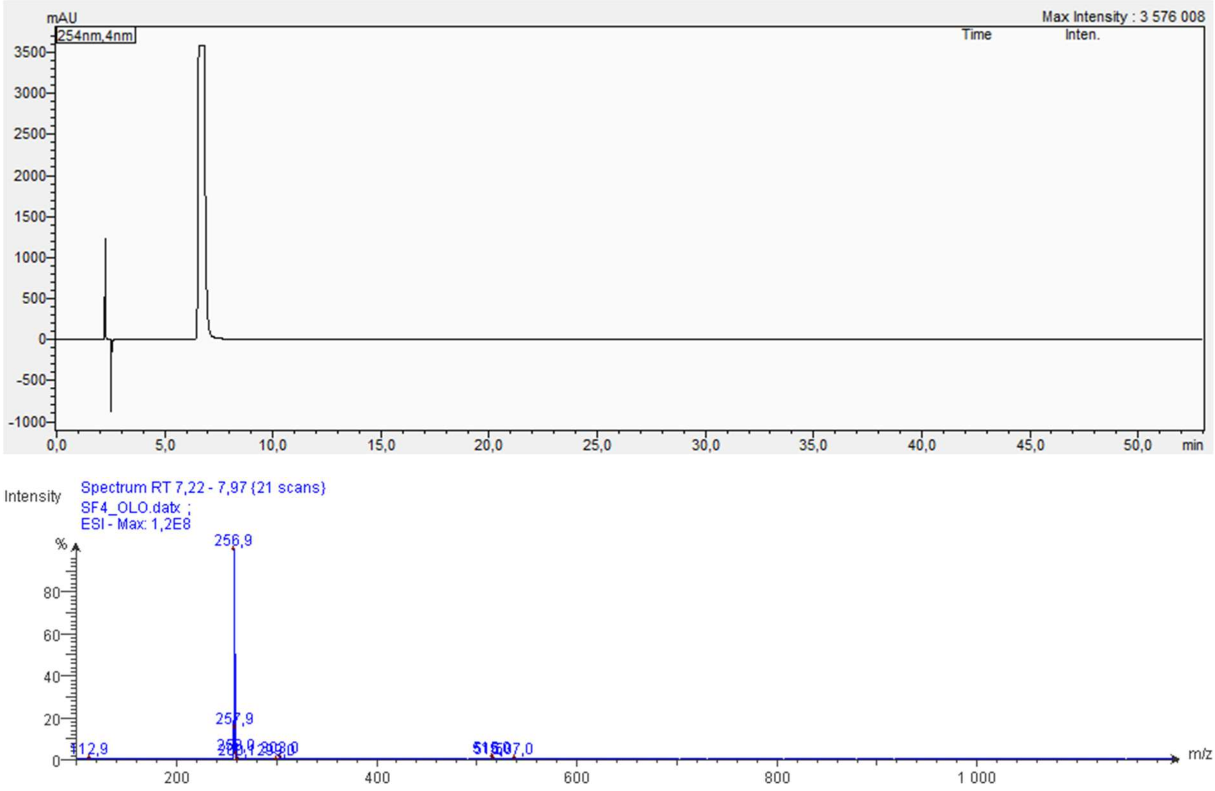

Figure S98. HPLC/DAD chromatogram and extracted MS spectrum of 2-chloronorlichexanthone **2** (*Rt* = 10.5 min)

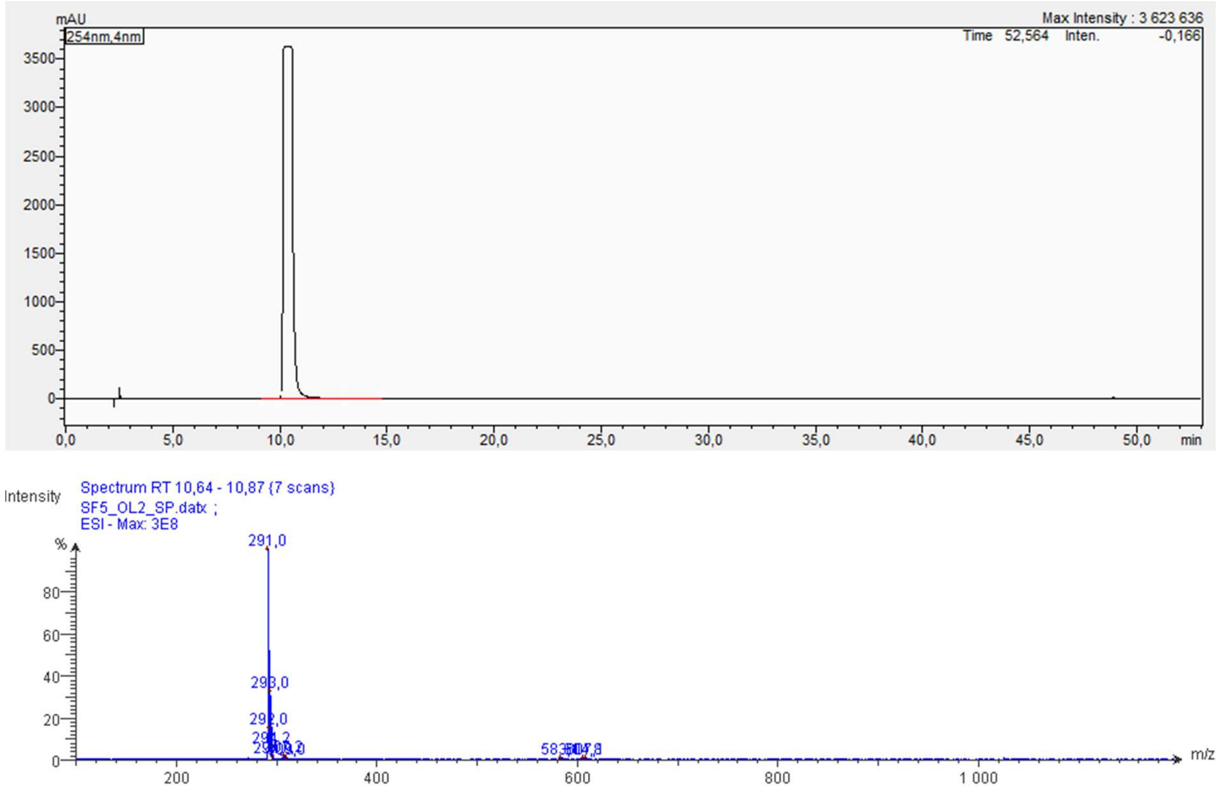

Figure S99. HPLC/DAD chromatogram and extracted MS spectrum of 4-chloronorlichexanthone **3** (*Rt* = 9.4 min)

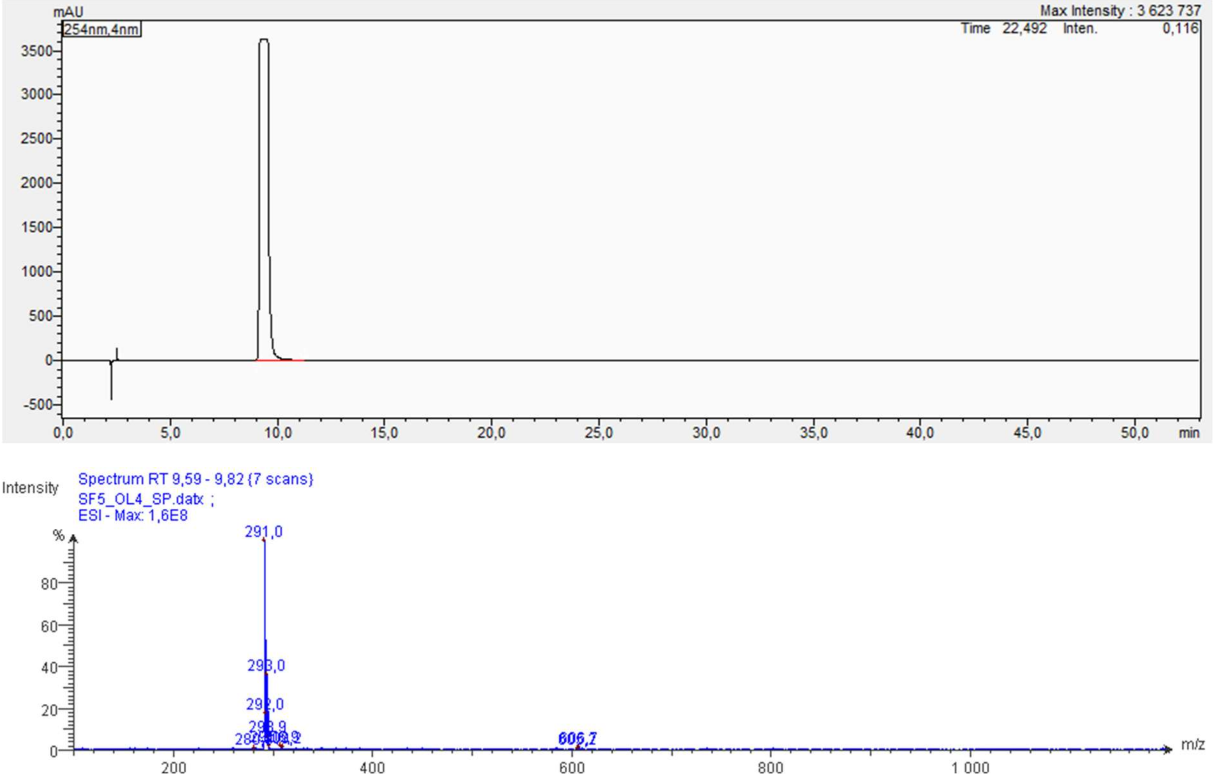

Figure S100. HPLC/DAD chromatogram and extracted MS spectrum of 5-chloronorlichexanthone **4** (*Rt* = 8.6 min)

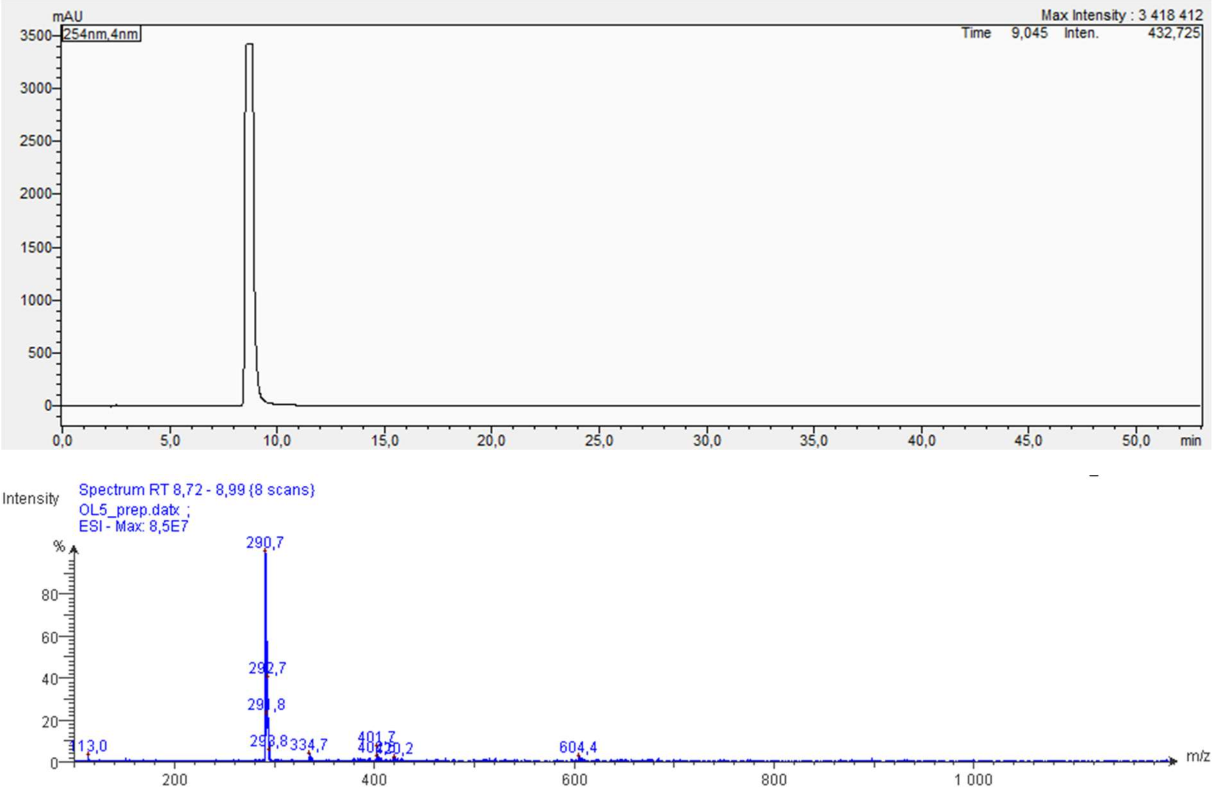

Figure S101. HPLC/DAD chromatogram and extracted MS spectrum of 7-chloronorlichexanthone **5** (*Rt* = 12.8 min)

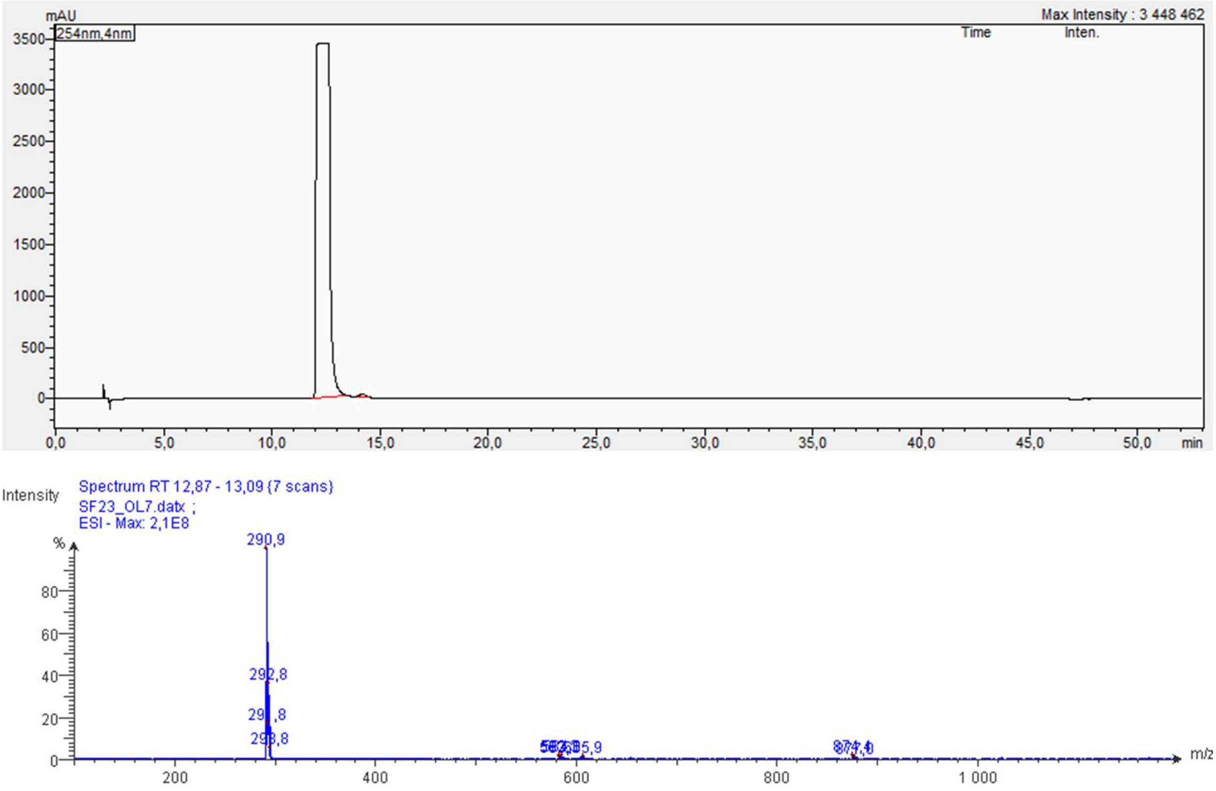

Figure S102. HPLC/DAD chromatogram and extracted MS spectrum of 2,4-dichloronorlichexanthone **6** (*Rt* = 15.6 min)

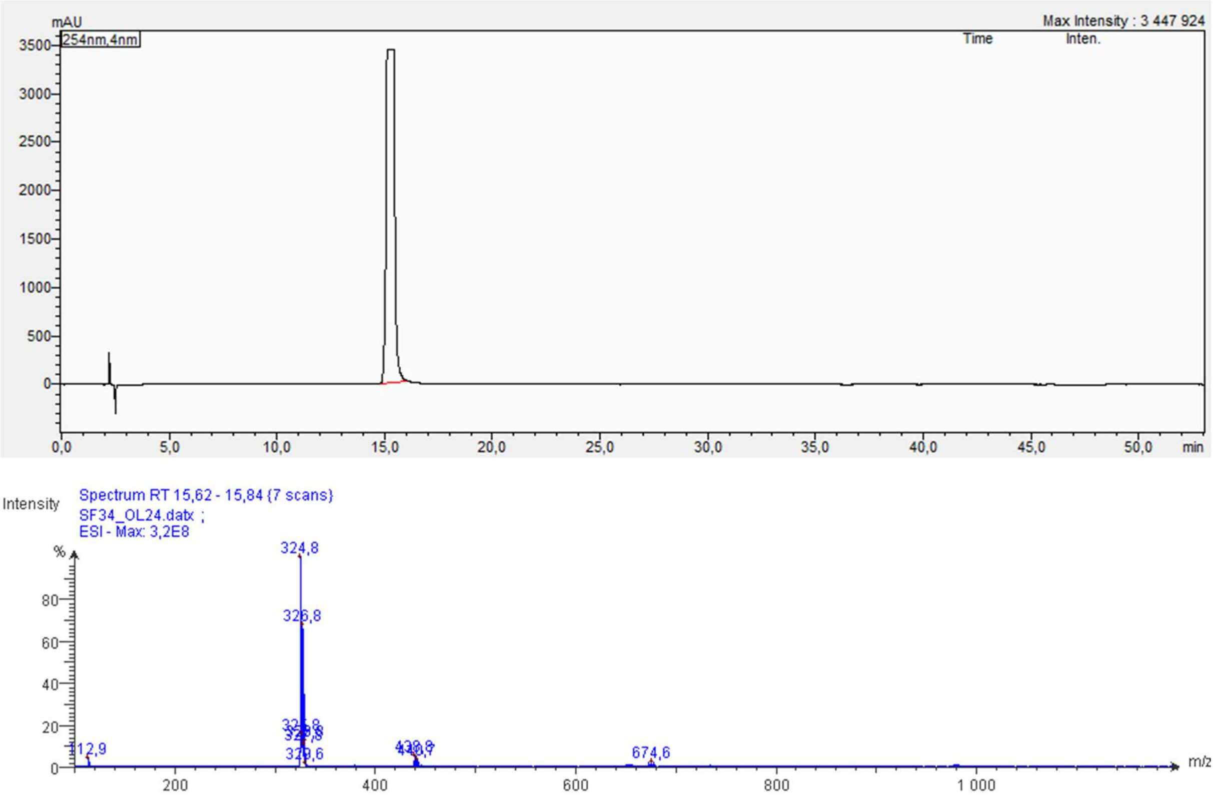

Figure S103. HPLC/DAD chromatogram and extracted MS spectrum of 2,5-dichloronorlichexanthone **7** (*Rt* = 13.4 min)

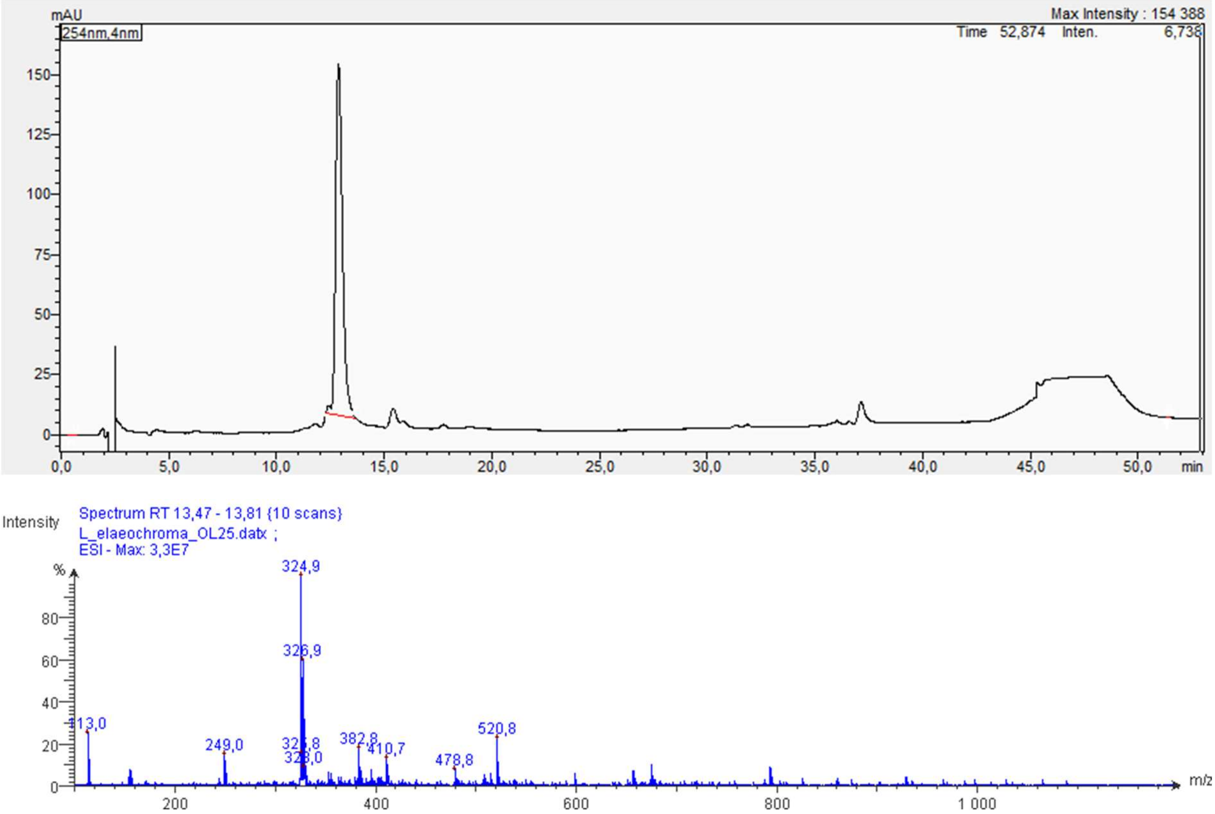

Figure S104. HPLC/DAD chromatogram and extracted MS spectrum of 2,7-dichloronorlichexanthone **8** (*Rt* = 19.0 min)

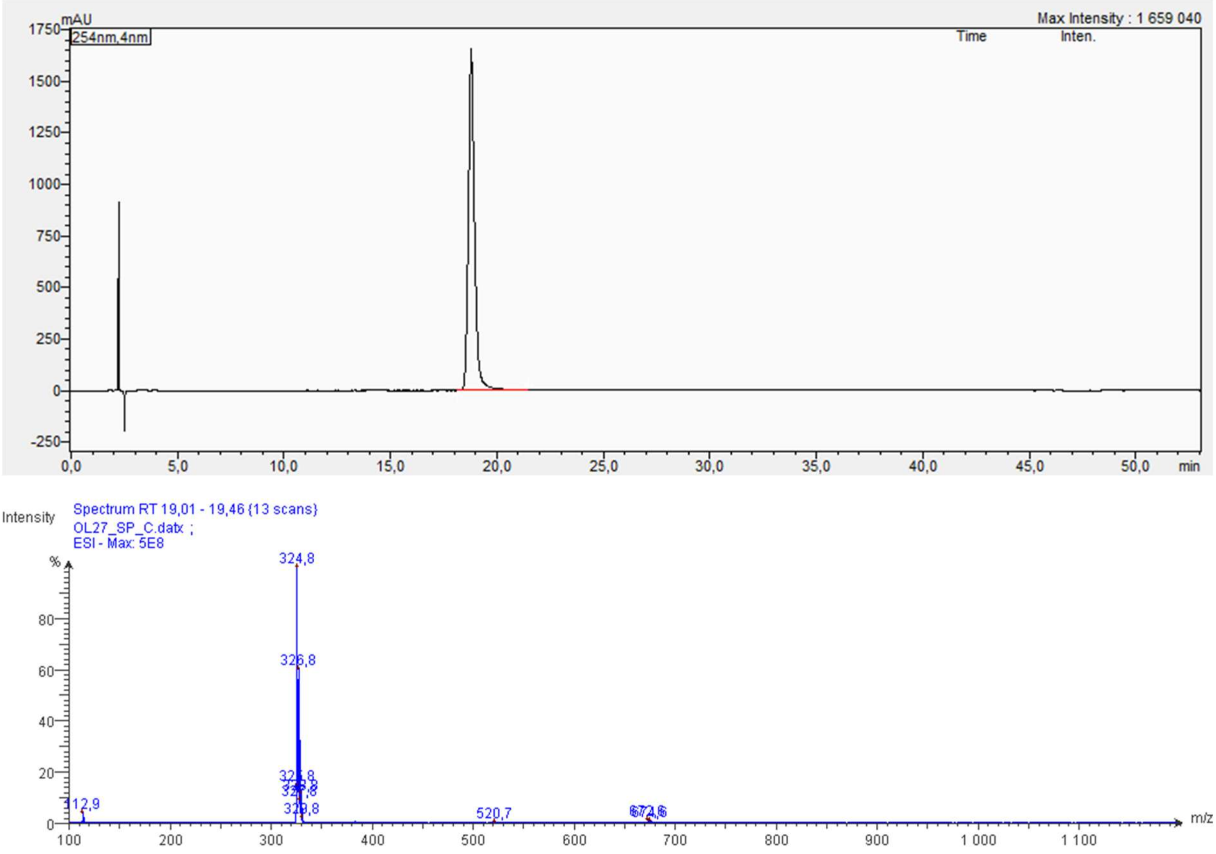

Figure S105. HPLC/DAD chromatogram and extracted MS spectrum of 4,5-dichloronorlichexanthone **9** (*Rt* = 12.1 min)

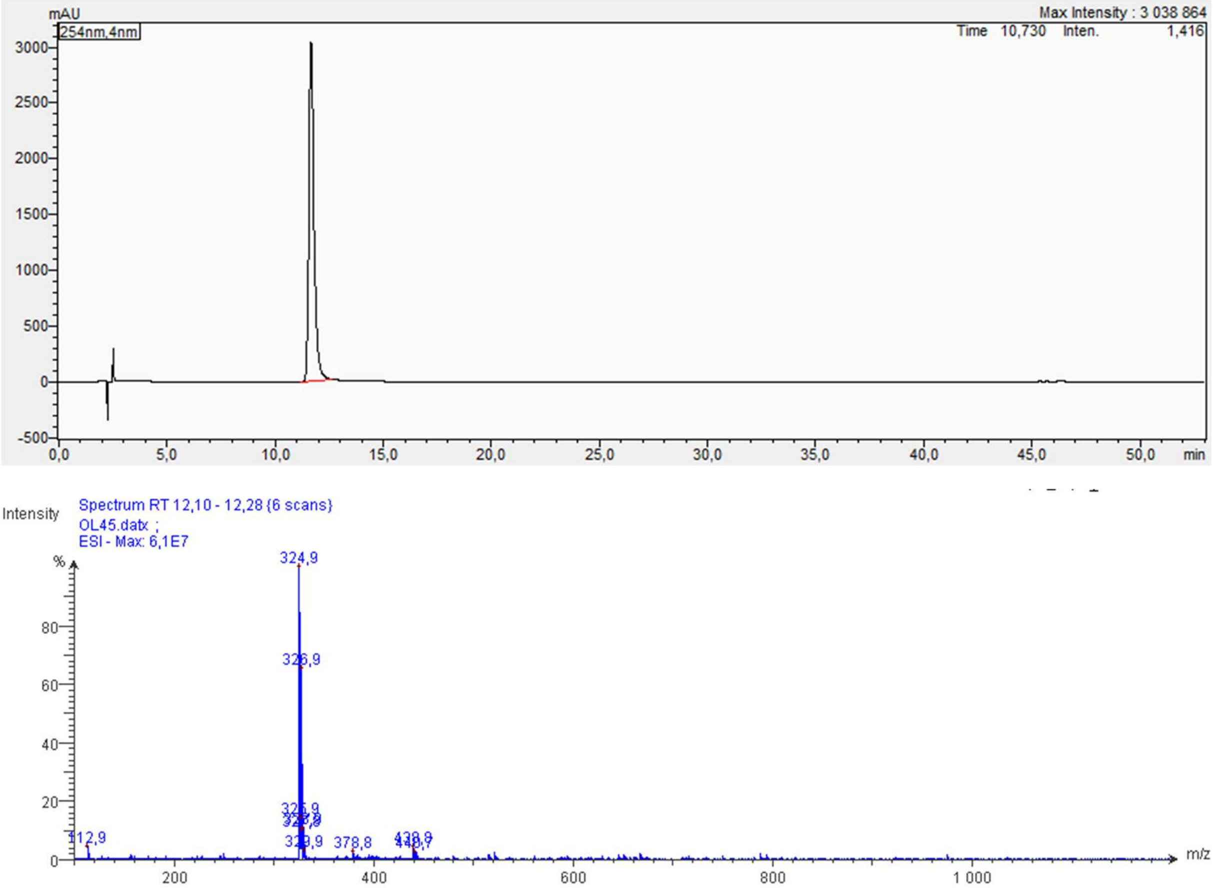

Figure S106. HPLC/DAD chromatogram and extracted MS spectrum of 4,7-dichloronorlichexanthone **10** (*Rt* = 16.8 min)

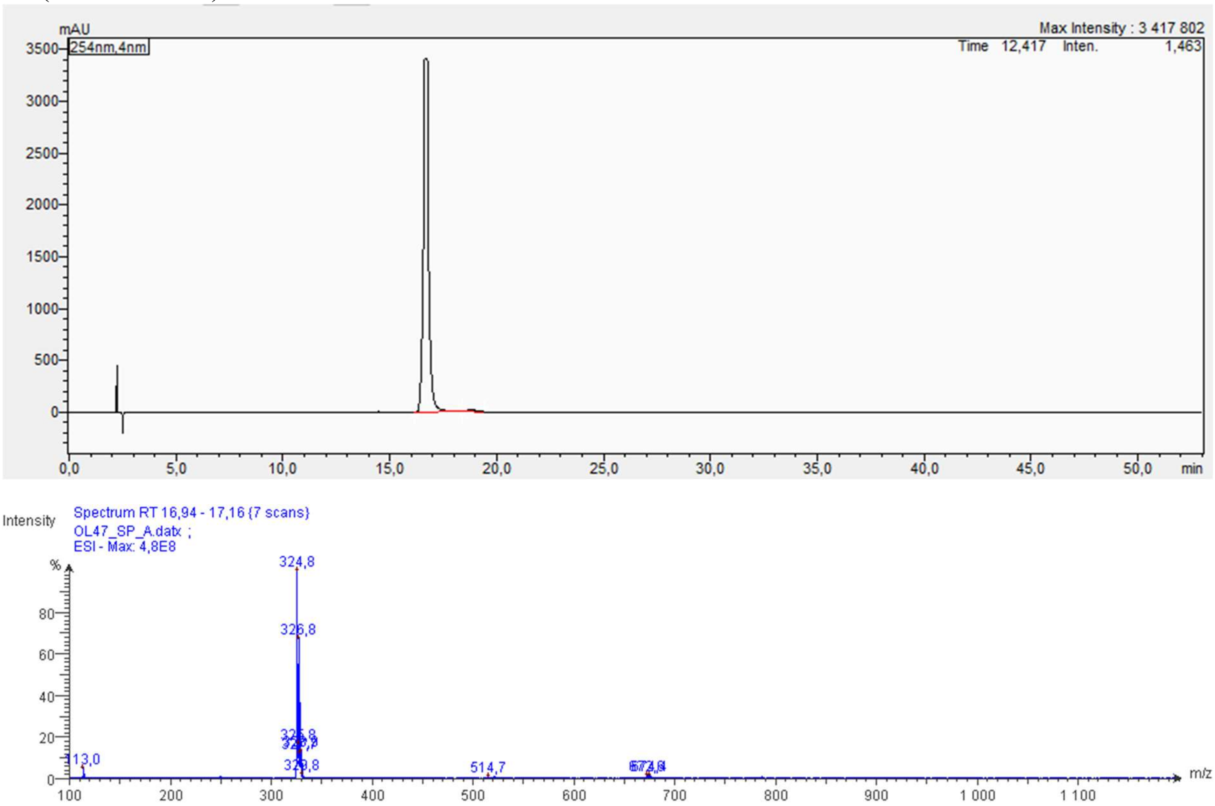

Figure S107. HPLC/DAD chromatogram and extracted MS spectrum of 5,7-dichloronorlichexanthone **11** (*Rt* = 17.4 min)

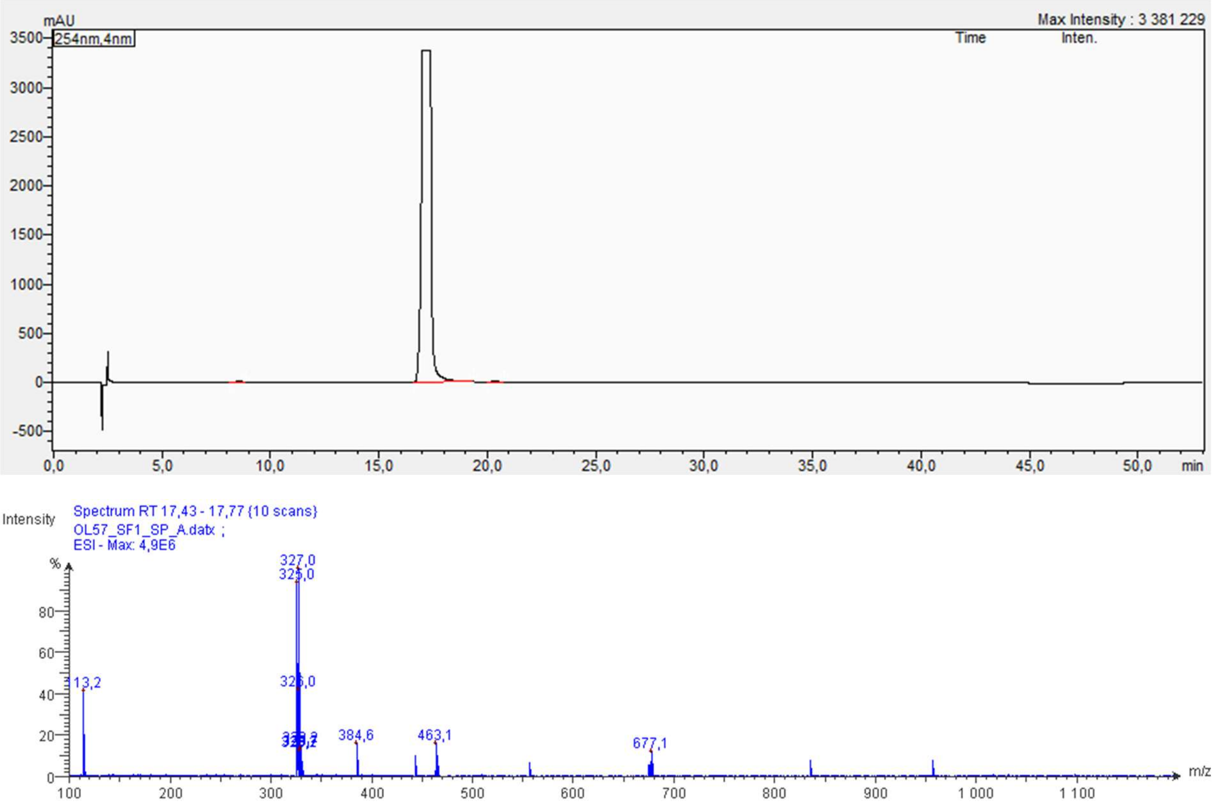

Figure S108. HPLC/DAD chromatogram and extracted MS spectrum of 2,4,5-trichloronorlichexanthone **12** (*Rt* = 18.8 min)

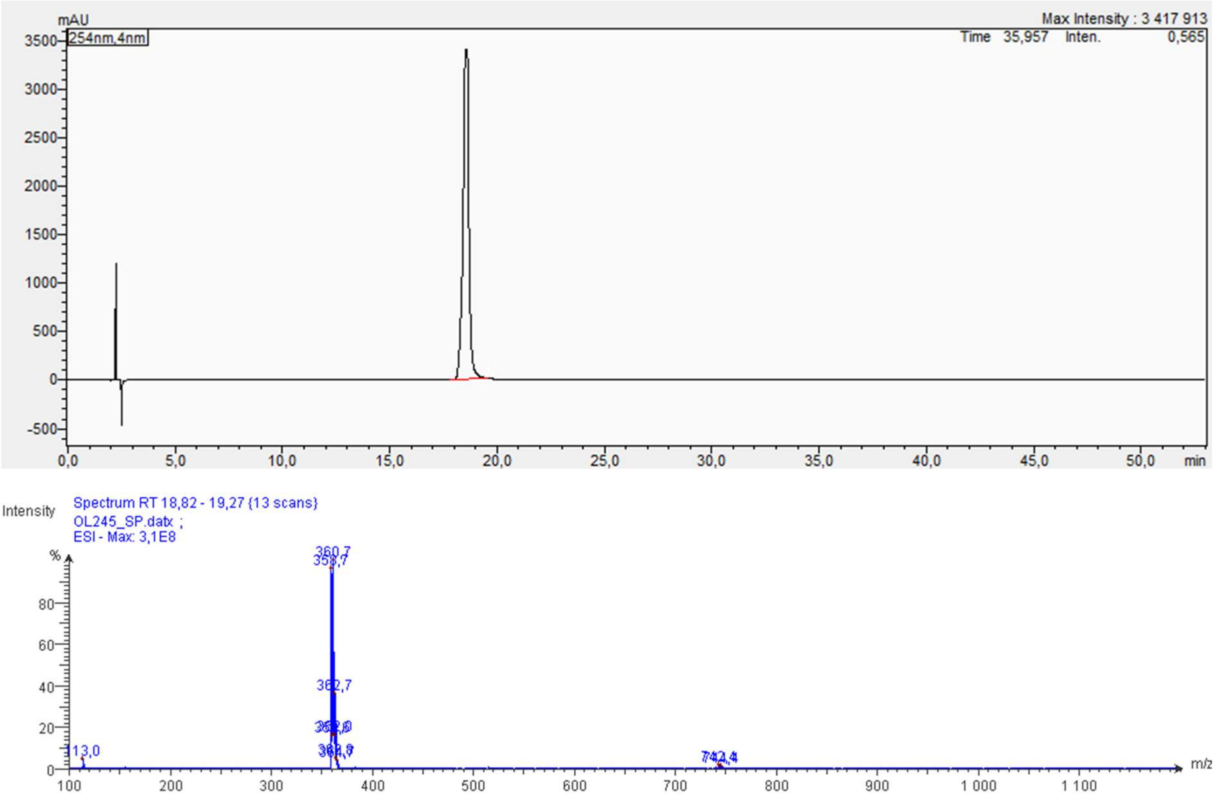

Figure S109. HPLC/DAD chromatogram and extracted MS spectrum of 2,4,7-trichloronorlichexanthone **13** (*Rt* = 26.1 min)

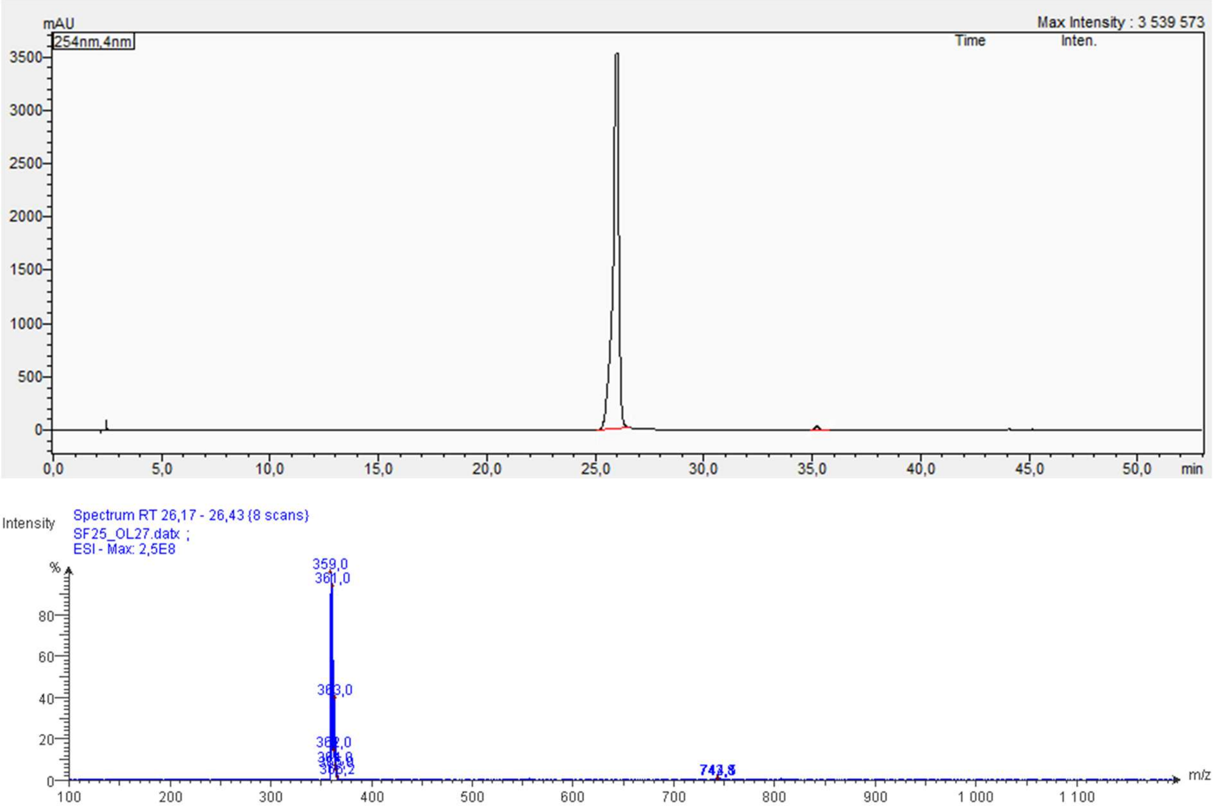

Figure S110. HPLC/DAD chromatogram and extracted MS spectrum of 2,5,7-trichloronorlichexanthone **14** (*Rt* = 24.4 min)

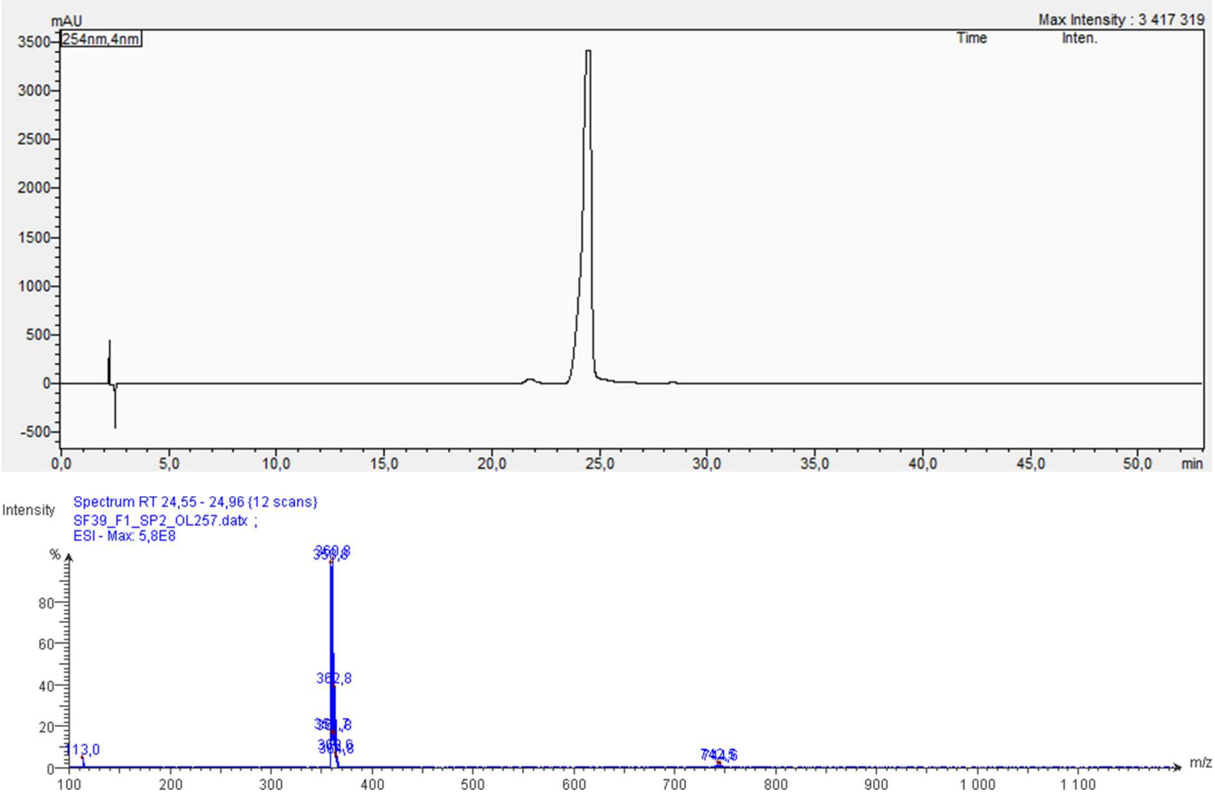

Figure S111. HPLC/DAD chromatogram and extracted MS spectrum of 4,5,7-trichloronorlichexanthone **15** (*Rt* = 22 min)

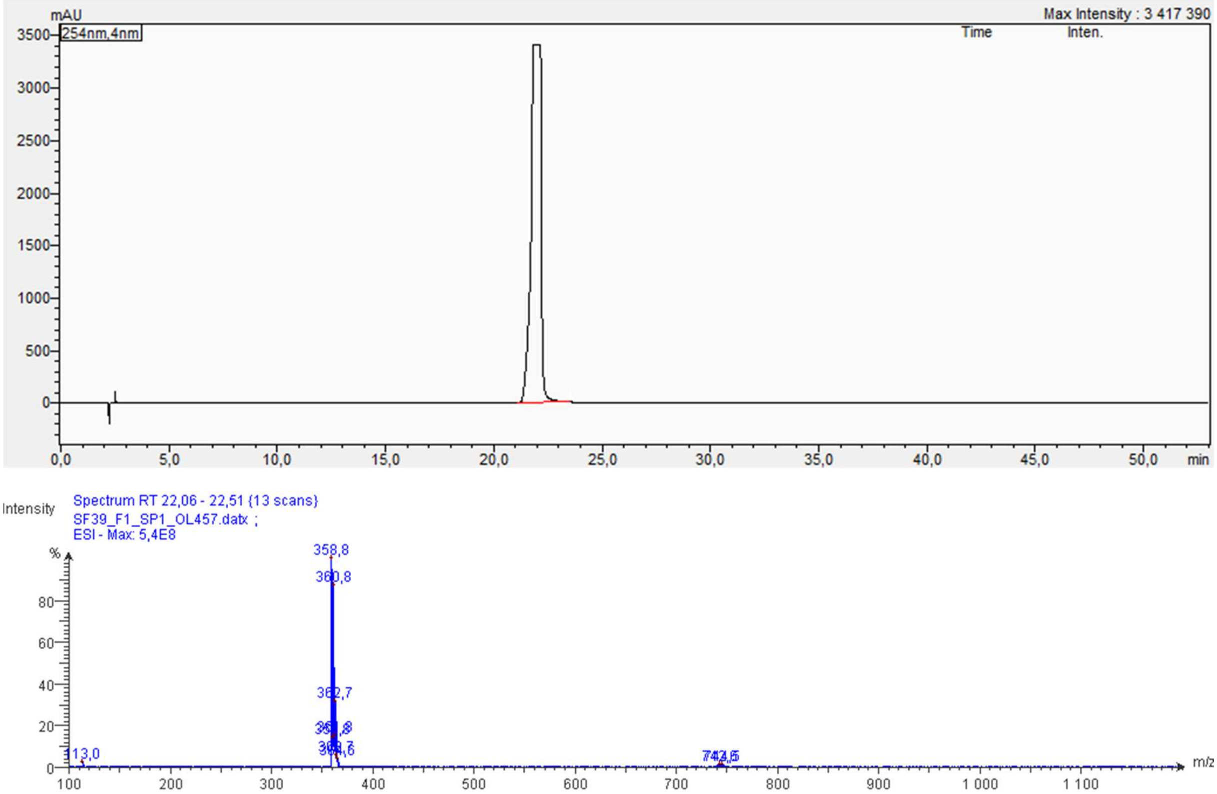

Figure S112. HPLC/DAD chromatogram and extracted MS spectrum of 2,4,5,7-tetrachloronorlichexanthone **16** (*Rt* = 30.8 min)

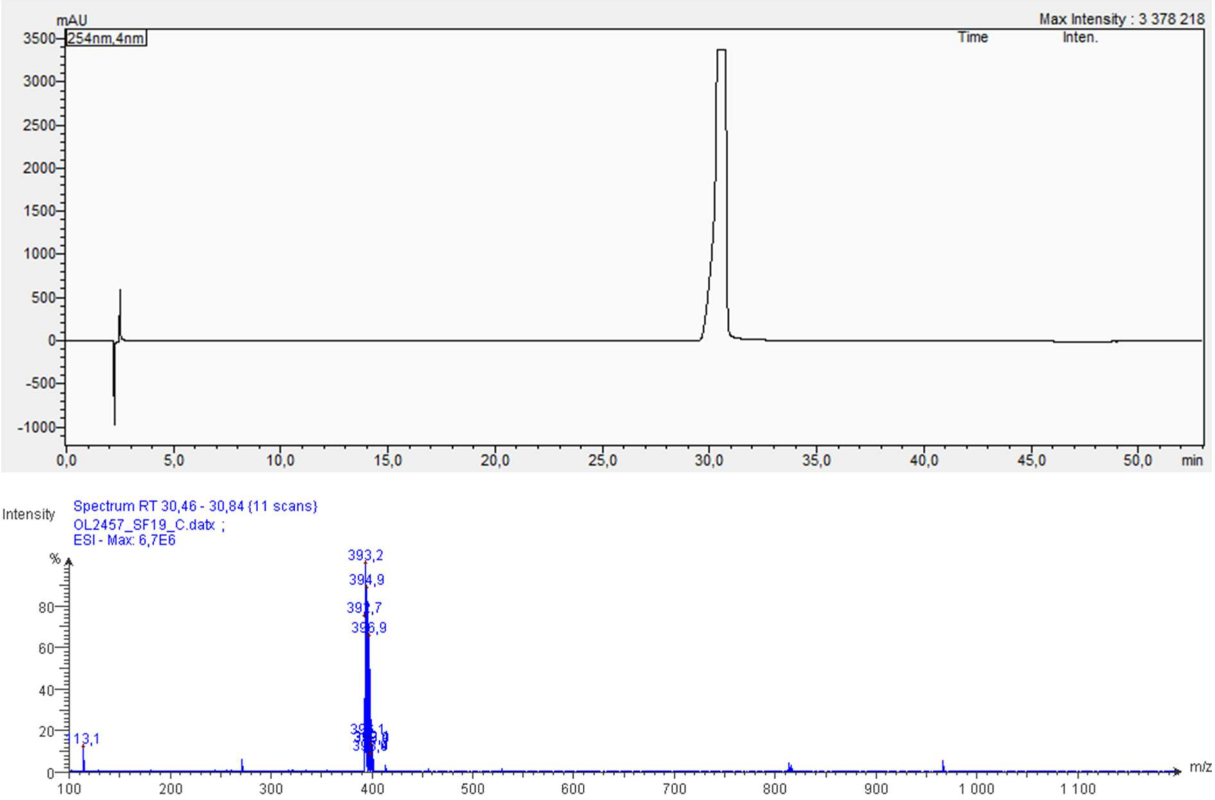

Figure S113. Superimposition of the chromatograms of the four monochlorinated norlichexanthones (compounds **2-5**)

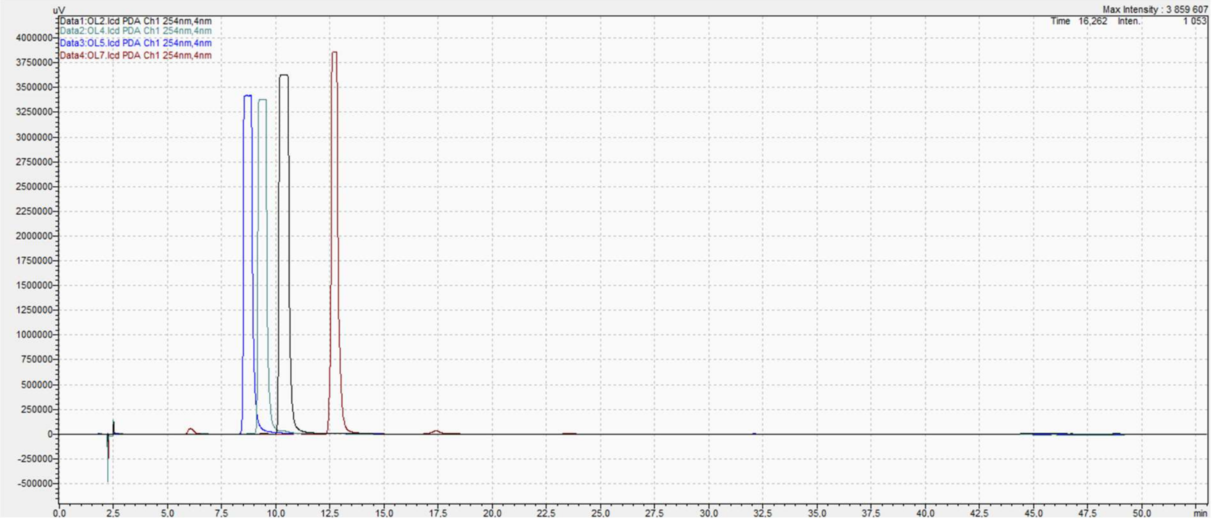

Figure S114. Superimposition of the chromatograms of the six dichlorinated norlichexanthones (compounds **6-11**)

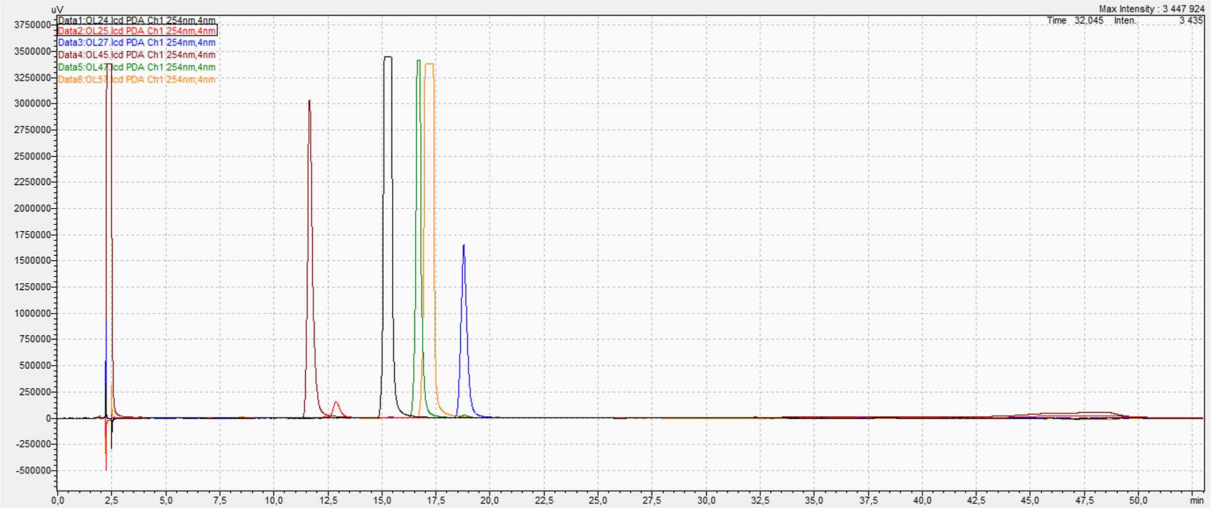

Figure S115. Superimposition of the chromatograms of the four trichlorinated norlichexanthones (compounds **12-15**)

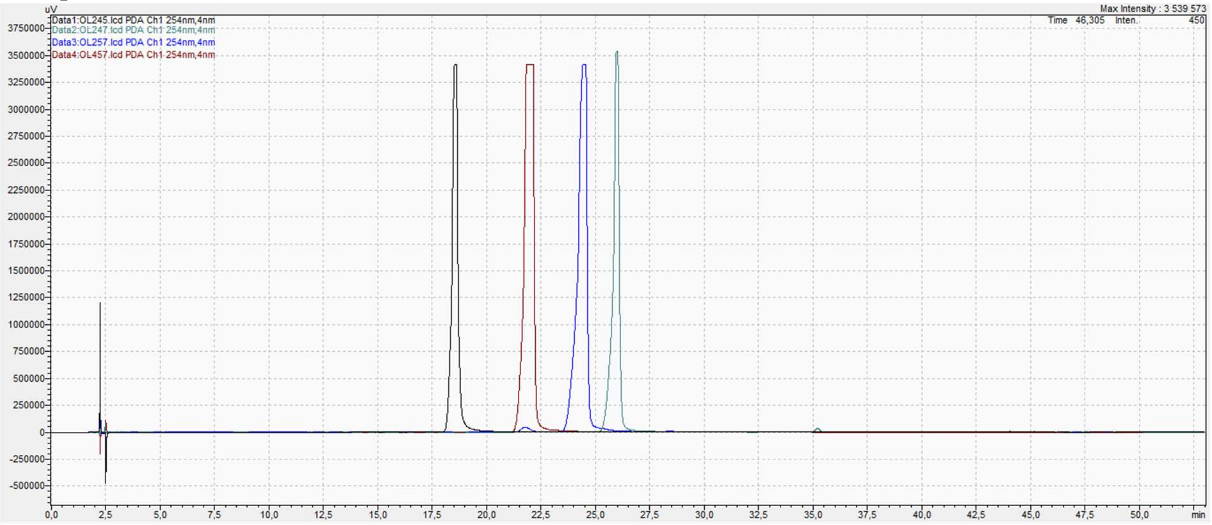

Figure S116. Separation of norlichexanthones along the lowest m/z ratio and retention time axes (Rt and m/z).
The colours of the dots represent the number of chlorine atoms of the molecule (red: 0, m/z= 257; green: 1, m/z= 291; blue: 2, m/z= 325; yellow: 3, m/z= 359 and purple: 4, m/z= 393).

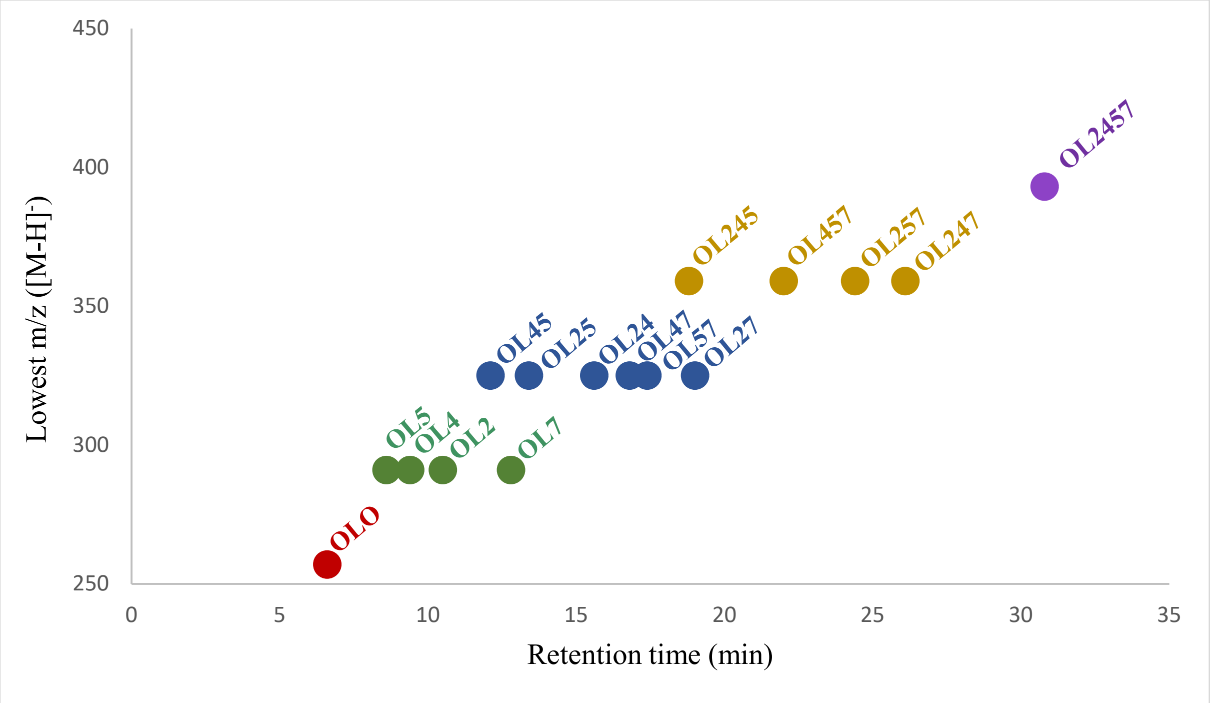

4. HPLC/DAD chromatograms of lichens extracts

Figure S117. HPLC/DAD chromatogram of *L. asema* var. *elaeochromoides*

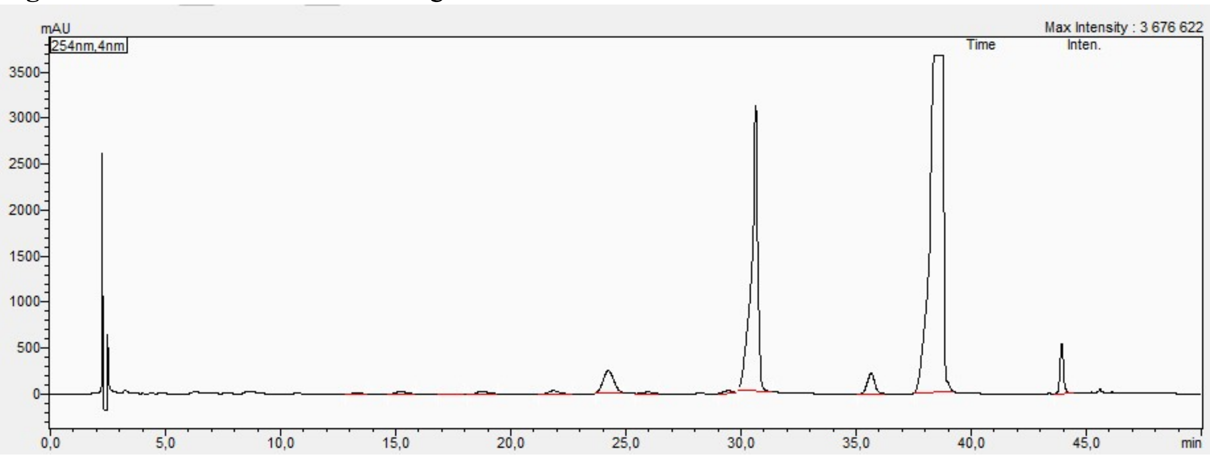

Figure S118. HPLC/DAD chromatogram of *M. antiqua*

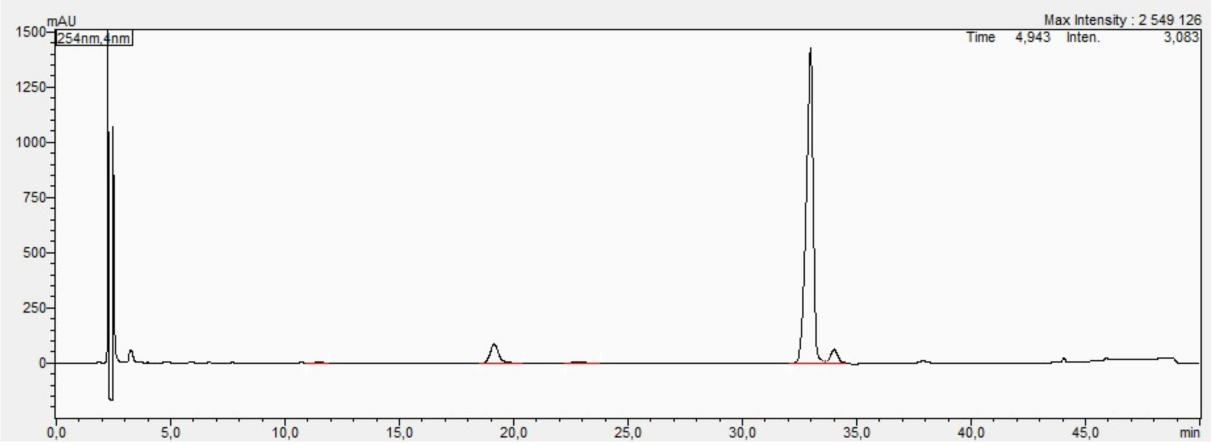

Figure S119. HPLC/DAD chromatogram of *L. alboflavida*

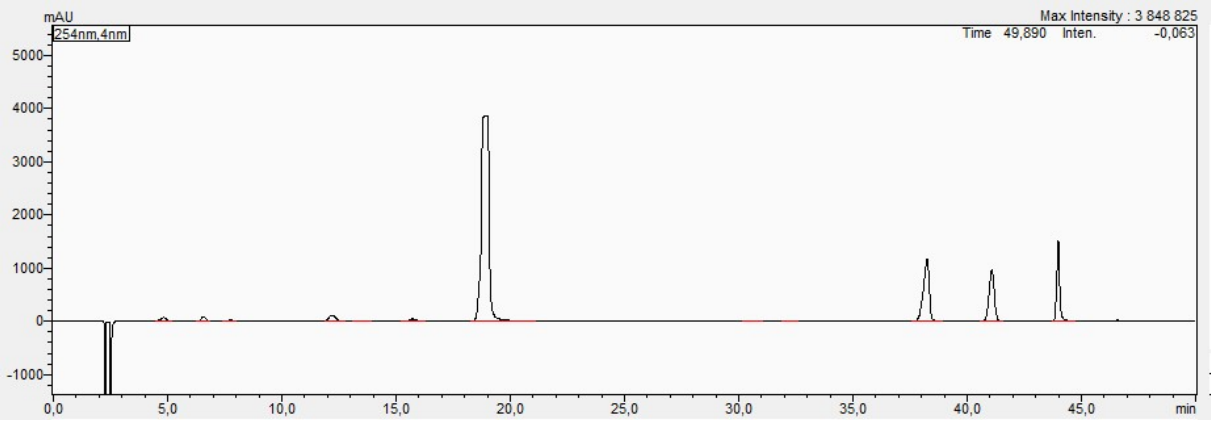

Figure S120. HPLC/DAD chromatogram of *P. quernea*

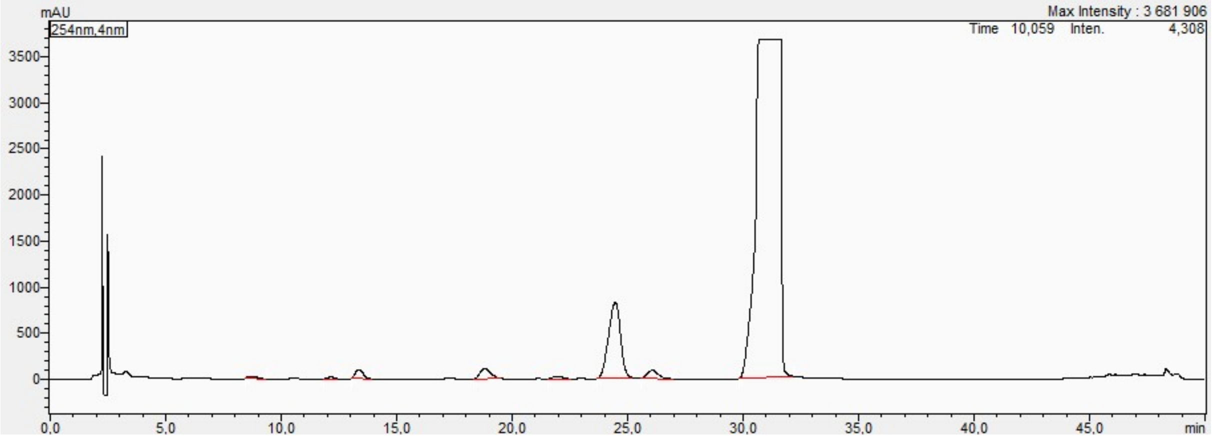

Figure S121. HPLC/DAD chromatogram of *L. elaeochroma*

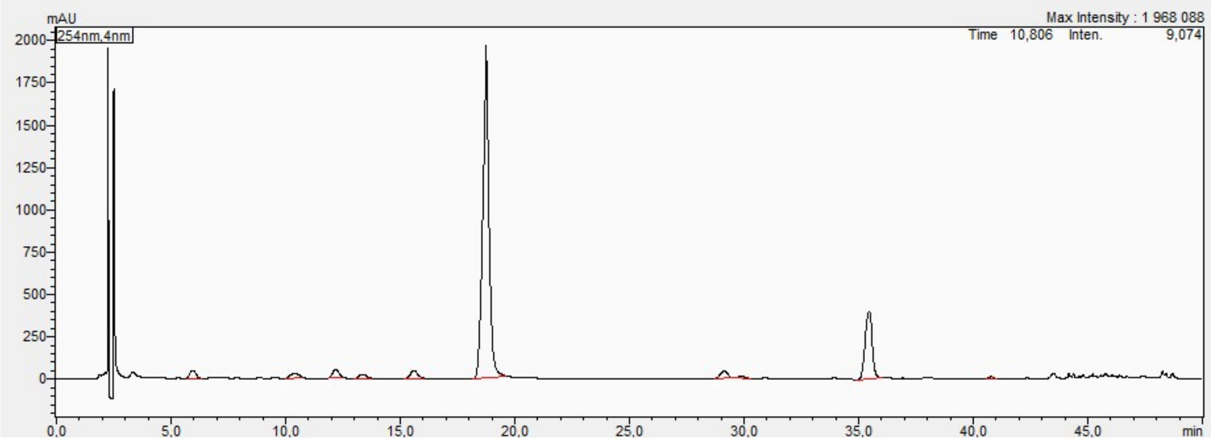

Supplement: Supplementary file 1 — Figure S1: pca70043‐sup‐0001‐Supplementary_Material.pdf. 1H NMR spectrum (500 MHz) in acetone‐d 6 of norlichexanthone 1. Figure S2: Jmod NMR spectrum (125 MHz) in acetone‐d 6 of norlichexanthone 1. Figure S3: HSQC NMR spectrum (500/125 MHz) in acetone‐d 6 of norlichexanthone 1. Figure S4: HMBC NMR spectrum (500/125 MHz) in acetone‐d 6 of norlichexanthone 1. Figure S5: NOESY NMR spectrum (500 MHz) in acetone‐d 6 of norlichexanthone 1. Figure S6: pca70043‐sup‐0001‐Supplementary_Material.pdf. 1H NMR spectrum (500 MHz) in acetone‐d 6 of 2‐chloronorlichexanthone 2. Figure S7: Jmod NMR spectrum (125 MHz) in acetone‐d 6 of 2‐chloronorlichexanthone 2. Figure S8: HSQC NMR spectrum (500/125 MHz) in acetone‐d 6 of 2‐chloronorlichexanthone 2. Figure S9: HMBC NMR spectrum (500/125 MHz) in acetone‐d 6 of 2‐chloronorlichexanthone 2. Figure S10: NOESY NMR spectrum (500 MHz) in acetone‐d 6 of 2‐chloronorlichexanthone 2. Figure S11: pca70043‐sup‐0001‐Supplementary_Material.pdf. 1H NMR spectrum (500 MHz) in acetone‐d 6 of 4‐chloronorlichexanthone 3. Figure S12: Jmod NMR spectrum (125 MHz) in acetone‐d 6 of 4‐chloronorlichexanthone 3. Figure S13: HSQC NMR spectrum (500/125 MHz) in acetone‐d 6 of 4‐chloronorlichexanthone 3. Figure S14: HMBC NMR spectrum (500/125 MHz) in acetone‐d 6 of 4‐chloronorlichexanthone 3. Figure S15: NOESY NMR spectrum (500 MHz) in acetone‐d 6 of 4‐chloronorlichexanthone 3. Figure S16: pca70043‐sup‐0001‐Supplementary_Material.pdf. 1H NMR spectrum (500 MHz) in acetone‐d 6 of 5‐chloronorlichexanthone 4. Figure S17: Jmod NMR spectrum (125 MHz) in acetone‐d 6 of 5‐chloronorlichexanthone 4. Figure S18: HSQC NMR spectrum (500/125 MHz) in acetone‐d 6 of 5‐chloronorlichexanthone 4. Figure S19: HMBC NMR spectrum (500/125 MHz) in acetone‐d 6 of 5‐chloronorlichexanthone 4. Figure S20: NOESY NMR spectrum (500 MHz) in acetone‐d 6 of 5‐chloronorlichexanthone 4. Figure S21: pca70043‐sup‐0001‐Supplementary_Material.pdf. 1H NMR spectrum (500 MHz) in acetone‐d 6 of 7‐chloronorlich [file PCA-37-330-s001.pdf]
